# Nickel-Mediated Radical Capture: Evidence for a Concerted Inner-Sphere Mechanism

**DOI:** 10.1021/jacs.5c01554

**Published:** 2025-05-30

**Authors:** Ethan H. Spielvogel, Jonathan Yuan, Norah M. Hoffmann, Tianning Diao

**Affiliations:** Department of Chemistry, 5894 New York University, 100 Washington Square East, New York, New York 10003, United States

## Abstract

Nickel catalysis enables cross-coupling of a broad scope of C­(sp^3^) moieties by mediating carbon–carbon bond formation from carbon-centered radicals. A widely proposed mechanism involves stepwise radical capture by a nickel­(II) complex that forms a nickel­(III) intermediate. The alternative pathway, a concerted radical capture and carbon–carbon bond formation, has been largely overlooked. This study investigates the ligand effect and kinetics of nickel-mediated radical capture and reductive elimination, which provide evidence to distinguish between stepwise and concerted pathways. Through radical clock experiments, spectroscopic investigation, electrochemical studies, and multivariate linear regression analysis of a series of [(pybox)­Ni­(Ar)]­BAr^F^_4_ complexes, we established a strong correlation between the rate of radical capture and HOMO and LUMO energies, along with positive charge stabilization at nickel and the aryl actor ligand. These data rule out the stepwise formation of a nickel­(III) intermediate and support a concerted pathway. Redox-active *nitrogen* ligands and nonredox-active *phosphine* ligands exhibit contrasting reactivity, with only redox-active ligands facilitating radical capture and carbon–carbon bond formation. This critical role of ligand redox activity can be attributed to the participation of the LUMO in bond cleavage and formation. Among redox-active ligands, bidentate and tridentate ligands exhibit similar rates, suggesting a consistent mechanism with relatively minimal ancillary ligand effect. Our results highlight the critical interplay between ligand electronics, sterics, and orbital contributions, offering valuable design principles for nickel-catalyzed cross-coupling reactions involving radical intermediates.

## Graphical abstract

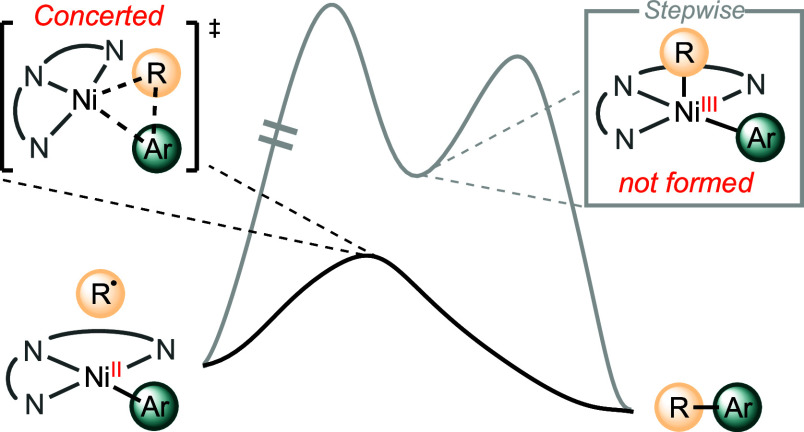

## Introduction

Synthesis of C­(sp^3^)-rich molecules is crucial for drug discovery.^1^ Nickel catalysis has emerged as powerful tools for cross-coupling C­(sp^3^) fragments in the form of carbon radicals,^2,3^ expanding the substrate scope to a wide variety of radical precursors activated through chemical,^4^ photochemical,^5^ and electrochemical redox methods.^6^ In addition, nickel-mediated coupling of carbon radicals enables stereoconvergent synthesis of chiral molecules.^7–9^ The success of nickel catalysts stem from their reactivity with carbon-centered radicals, facilitating subsequent carbon–carbon bond formation (Scheme 1A).^10,11^ Typical mechanistic proposals involve the capture of carbon radicals by a nickel­(II)-aryl or -alkyl intermediate to form a nickel­(III) species, followed by reductive elimination to generate the desired carbon–carbon bond.^12,13^ Catalytic screening and optimization have identified bidentate and tridentate redox-active π-acceptor *nitrogen* ligands, such as bipyridine (bpy), 1,10-phenanthroline (phen), and pyridine-bis-oxazoline (pybox), as the key to promote these reactions. Understanding the mechanisms of the radical–nickel interaction and the role of redox active ligands is crucial for advancing nickel-catalyzed cross-coupling reactions involving radical intermediates.

**Scheme 1:**
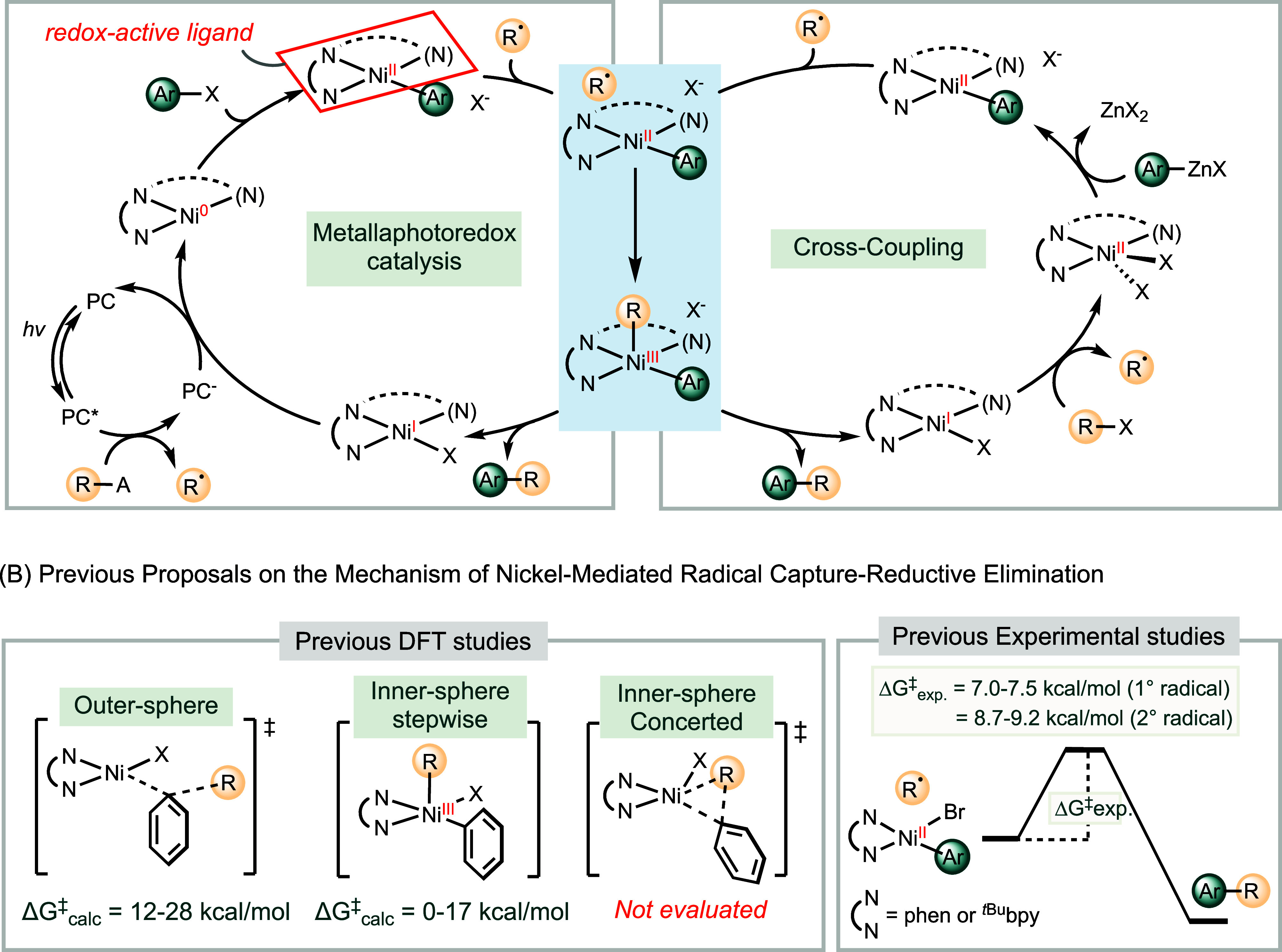
Mechanisms of Nickel-Catalyzed Carbon–Carbon Bond Formation Reactions Converging at a Radical Capture Step (A) and Possible Pathways for Nickel-Mediated Radical Capture and Bond Formation (B)

Previous studies on nickel-mediated radical capture have primarily relied on density functional theory (DFT) calculations. These studies have considered the outer-sphere carbon–carbon bond formation pathway^14^ and a stepwise inner-sphere mechanism involving the formation of a nickel­(III) intermediate, followed by reductive elimination (Scheme 1B).^15–20^ The latter has been the most widely proposed, with calculated activation energies for radical capture ranging from 0 to 17 kcal/mol, where the subsequent reductive elimination is typically identified as the rate-determining step. Additionally, light-induced isomerization of the nickel­(II) complex from square planar to tetrahedral geometry has been calculated to facilitate radical capture under photoredox conditions.^21,22^ An alternative mechanism involves a concerted inner-sphere process, where radical capture and carbon–carbon bond formation occur simultaneously, bypassing the formation of a discrete nickel­(III) intermediate. To the best of our knowledge, this mechanism has not been explicitly proposed or evaluated, either computationally or experimentally.

Experimental characterization of nickel-mediated radical capture and subsequent carbon–carbon bond formation remains limited.^23–25^ Recently, we investigated radical capture at catalytically relevant nickel­(II) complexes.^26^ Our study revealed a striking difference in reactivity between nickel-aryl and nickel–alkyl complexes. For nickel–alkyl complexes, EPR spectroscopy confirmed the formation of nickel­(III) intermediates, from which slow C­(sp^3^)–C­(sp^3^) bond formation precluded product formation.^26,27^ In contrast, nickel-aryl complexes showed no EPR signal that corresponds to nickel­(III) species but rapidly formed C­(sp^2^)–C­(sp^3^) coupling products in high yields. Via radical clock experiments, we benchmarked the overall rates of radical capture and subsequent C­(sp^2^)–C­(sp^3^) bond formation, ranging from 10^7^ M^–1^ s^–1^ for primary radicals to 10^6^ M^–1^ s^–1^ for secondary radicals (Scheme 1B). Notably, the activation energy observed in this study deviated from previous estimations by computational methods.

Previous experimental and computational studies suggested an inner-sphere mechanism for nickel-aryl-mediated radical capture and C­(sp^2^)–C­(sp^3^) bond formation. However, these studies have not differentiated between a stepwise pathway involving the formation of a nickel­(III) intermediate and a concerted pathway. Additionally, the effect of redox-active ligands in nickel-catalyzed cross-coupling, particularly during the radical capture step, remains unexplored. Herein, we present a detailed investigation into the steric and electronic effect of both supporting and actor ligands. Through statistical modeling,^28^ we provide evidence to distinguish between stepwise and concerted inner-sphere pathways. Furthermore, we contextualize the catalytic success of redox-active ligands, offering valuable insights for ligand design in catalytic reactions with radical capture as an intermediate step.

## Results and Discussion

### Effect of the Redox Activity of Supporting Ligands

We prepared a series of nickel-aryl complexes and evaluated their stoichiometric reactivity in capturing benzyl radical, generated by near-UV irradiation of Hantzsch ester **1**,^29^ and mediating the formation of benzyl-arene **2** (Scheme 2). Complexes **3**-**6**, featuring bidentate and tridentate *pyridine* and *oxazoline-*ligands, produced cross-coupling product **2** in high yields,^26^ whereas complexes **7**-**10**, supported by *phosphine* and *amine-*ligands, resulted in little to no product.

**Scheme 2:**
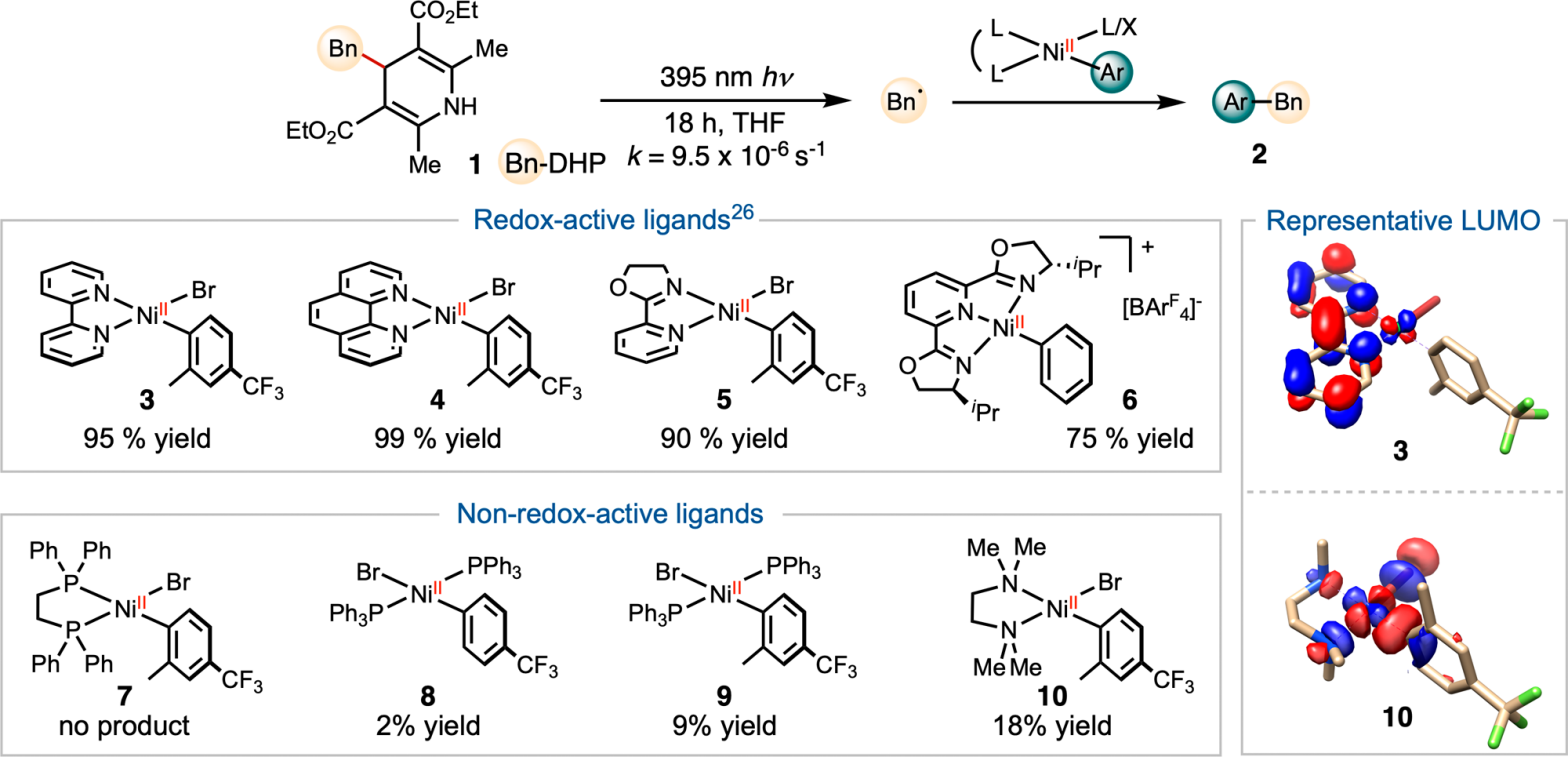
Stoichiometric Radical Capture with Redox-Active and Non-Redox-Active Nickel Complexes

DFT calculations using the ORCA package^30^ with BP86/def2-SVP level of theory revealed the electronic structures of **3–10**. Complexes **3**–**6** feature LUMOs delocalized over the π* orbitals of the supporting ligands, a key criterion for ligand redox-activity (Scheme 2).^31^ In contrast, the LUMOs of complexes **7**–**10** primarily exhibit metal-centered d-orbital character. The superior reactivity of ligands **3**-**6** correlates with their redox-active nature—a property critical for initiating radical formation and stabilizing metalloradical intermediates.^32^ While this initial observation suggested that the LUMO is involved in the bond forming process, the connection between the canonical stepwise radical capture mechanism and the LUMO remained unclear. To gain deeper mechanistic insight into the role of ligand redox activity, we investigated the influence of ligand steric and electronic effects on radical capture rates.

### Methodology for Determining the Rates of Radical Capture at [(Pybox)­Ni­(II)­Ar]­BAr^F^_4_

Given the photoinstability of bpy and phen-coordinated nickel­(II)–aryl complexes,^33–35^ we selected [(pybox)­Ni­(Ar)]­BAr^F^_4_ complexes as a model system to investigate the effects of ligands on the rates of radical capture and subsequent reductive elimination (Scheme 3).^36^ Pybox ligands promote stereoconvergent coupling^37^ and cross-electrophile coupling reactions^38,39^ while offering improved photostability. The constrained square planar geometry of [(pybox)­Ni­(Ar)]­BAr^F^_4_ complexes prevents complications arising from adopting the tetrahedral conformation required for photoinduced aryl radical ejection^34^ and biaryl formation.^40^ Moreover, the absence of inner-sphere halide coordination in these complexes precludes the possibility of photoinduced protodemetalation and halogen photoelimination.^33,35^

**Scheme 3:**
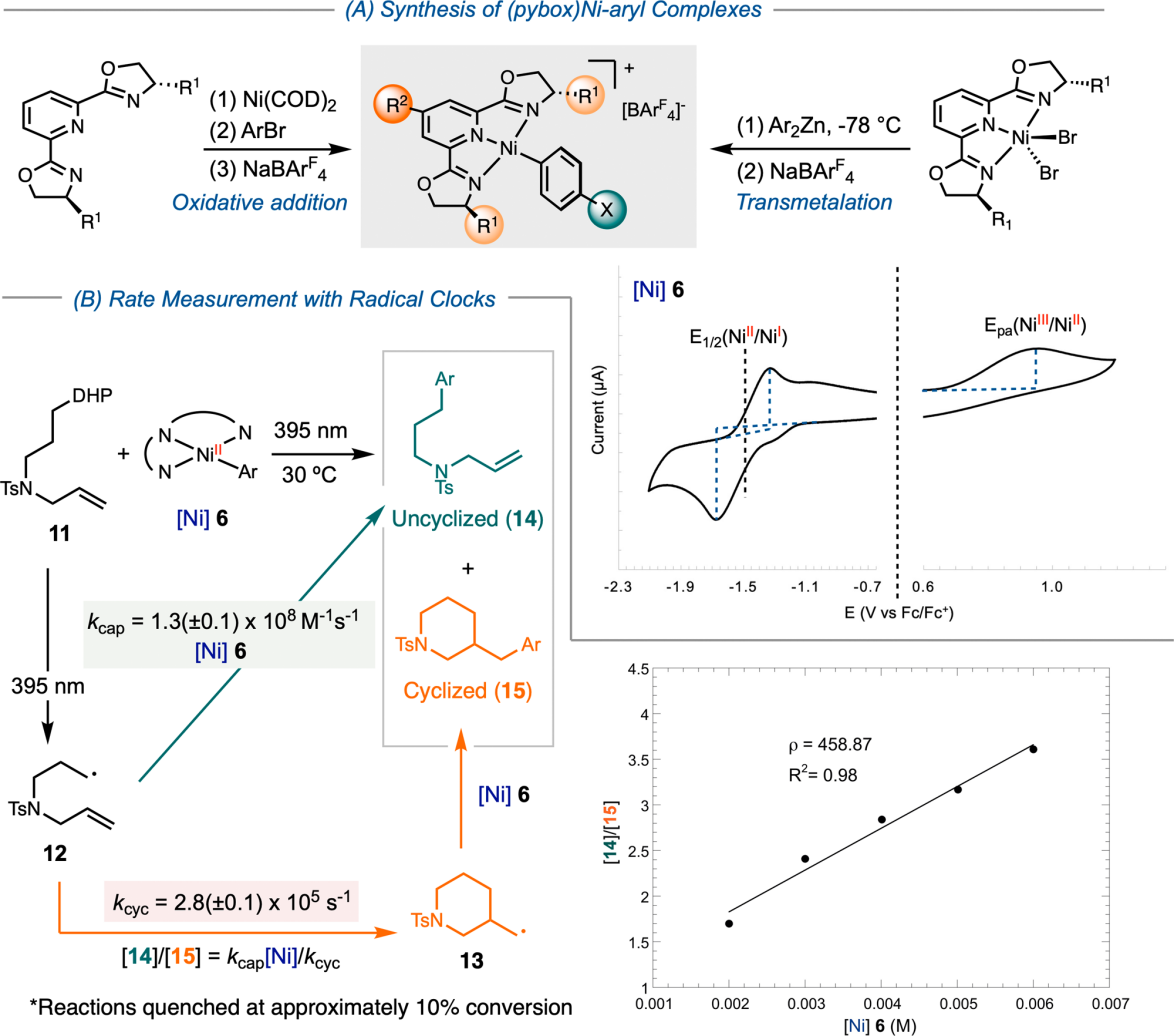
Methodology for Investigating Rates of Radical Capture by [(^*i*Pr^pybox)­Ni­(Ar)]­BAr^F^_4_ Complexes: Synthesis, CV Studies (A) and Rates Determination by Radical Clock Experiments (B)

We synthesized a series of [(^*i*Pr^pybox)­Ni­(Ar)]­BAr^F^_4_ complexes with varying substituents at the 4-position of the pyridine ring in ^*i*Pr^pybox, as well as different substituents on the oxazoline and aryl groups coordinated to nickel (Scheme 3A). For electron-rich or neutral derivatives, we modified the previously established transmetalation protocol involving (^*i*Pr^pybox)­NiBr_2_ with diarylzinc reagents to obtain the desired complexes **6, 21–28** and **31**.^36^ However, for [(^*i*Pr^pybox)­Ni­(Ar)]­BAr^F^_4_ complexes bearing electron-withdrawing groups, such as acetyl, aldehyde, or cyano, the corresponding aryl halide reagents were incompatible with diarylzinc generation. Instead, we employed an oxidative addition strategy using *in situ* generated (^*i*Pr^pybox)­Ni­(COD) (COD = 1,5-cyclooctadiene) to synthesize **29**, **30**, and **32**. For the bulky [(^*i*Pr^pybox)­Ni­(Mes)]­BAr^F^_4_ **16** (Mes = 2,4,6-mesityl), we adopted a different route, utilizing transmetalation of mesityl Grignard to in situ generated (^*i*Pr^pybox)­Ni­(acac)_2_ (acac = acetylacetonate).

We recorded cyclic voltammograms (CVs) for each [(^*i*Pr^pybox)­Ni­(Ar)]­BAr^F^_4_ compound (Scheme 3A). The reduction waves were quasi-reversible for the parent and electron-deficient complexes, as well as for electron-rich variants at fast scan rates. Oxidation, however, was irreversible across all [(^*i*Pr^pybox)­Ni­(Ar)]­BAr^F^_4_ complexes. The reduction potentials *E*_1/2_(Ni^II^/Ni^I^) and oxidation potentials *E*_pa_(Ni^III^/Ni^II^) for each compound follow a general trend: increasing electron-donating substituents on the pybox ligand inhibits reduction while promoting oxidation (Scheme 5). However, the electronic effects of substituents on the aryl actor ligand were less straightforward. This observation may stem from the perpendicular geometry of the aryl group relative to the pybox plane and the d_z2_ orbital of nickel. Notably, complex [(^*i*Pr^pybox)­Ni­(*p*-CN-C_6_H_4_)]­BAr^F^_4_ **32** displayed a distinct CV profile, characterized by multiple reversible reduction features and a diffusional peak following oxidation (Figure S47). We attribute these features to the redox reactions occurring directly on the cyanoarene moiety.

We applied radical clock substrate **11** to determine the overall rates of radical capture and reductive elimination at [(^*i*Pr^pybox)­Ni­(Ar)]­BAr^F^_4_ complexes **6** and **21–33** (Scheme 3B). Upon irradiation with a 395 nm LED, substrate **11** generates a radical intermediate **12** that can proceed via two competing pathways to yield either the uncyclized product **14** or the cyclized product **15**. The selectivity between these products, expressed as the ratio of [**14**]/[**15**], is proportional to [Ni] if both pathways are irreversible, as the formation of **14** is a bimolecular process, while the formation of **15** depends on the rate of unimolecular cyclization (cf. Supporting Information for expanded discussion).

For each reaction, we quenched the reaction at approximately 10% conversion of the nickel complex to accurately capture initial rates. At later stages, changes in [Ni] and the formation of nickel­(I) species from reductive elimination could compromise the accuracy of kinetic analysis. By varying [Ni], we observed a linear relationship between [**14**]/[**15**] and [Ni], with the slope corresponding to the ratio of *k*_cap_/*k*_cyc_, where *k*_cap_ is radical capture rate by nickel and *k*_cyc_ is the rate of 6-exo radical cyclization. Using the previously calibrated value of *k*_cyc_ = 2.8(±0.1) × 10^5^ s^–1^,^26^ we determined the rates of primary radical capture and reductive elimination at nickel complexes **6** and **21**-**33** (Scheme 5).

### Steric Effect on Radical Capture

Following the protocol outlined above, we evaluated the steric effects of the actor ligand, supporting ligand, and incoming radical (Scheme 4). [(^*i*Pr^pybox)­Ni­(Mes)]­BAr^F^_4_ **16** failed to trap the benzyl radical and generate any cross-coupling product (Scheme 4A), in contrast to the excellent reactivity previously observed with [(^*i*Pr^pybox)­Ni­(Ph)]­BAr^F^_4_ **6** (Scheme 4A).^26^ When using [(^Me^pybox)­Ni­(Ph)]­BAr^F^_4_ **19**, the reduced steric hindrance of ^Me^pybox resulted in a slightly faster rate of radical capture compared to [(^*i*Pr^pybox)­Ni­(Ph)]­BAr^F^_4_ **6** (Scheme 4B). To further investigate the effect of radical identity, we employed radical clock substrates **11** and **20** to compare the rates of primary and secondary alkyl radicals with [(^*i*Pr^pybox)­Ni­(Ph)]­BAr^F^_4_ **6** (Scheme 4C). The data revealed that trapping of primary radicals was approximately 2 orders of magnitude faster than that of secondary radicals, consistent with our previous findings for bidentate complexes.^26^ These results highlight a significant steric effect on radical trapping and reductive elimination, demonstrating that less sterically hindered substrates and nickel complexes enable faster reactivity.

**Scheme 4:**
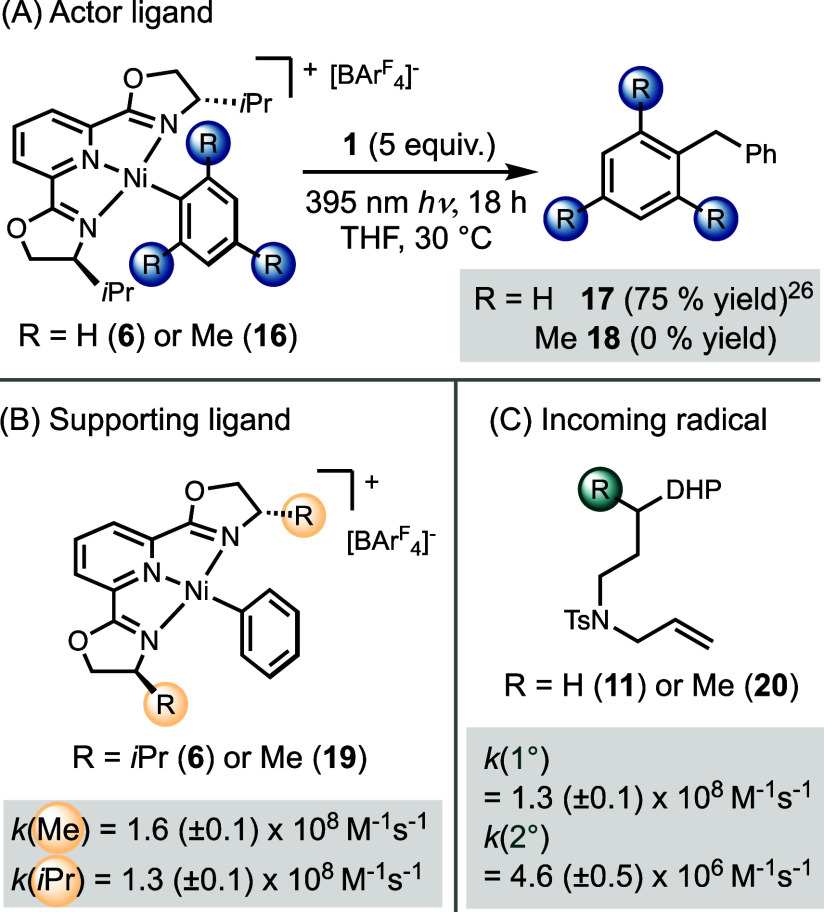
Steric Effect of the Actor Ligand (A), Supporting Ligand (B), and the Incoming Radical (C)

### Electronic Effect on Radical Capture

Subsequently, we conducted radical clock experiments on a series of [(^*i*Pr^pybox)­Ni­(Ar)]­BAr^F^_4_ complexes **21**-**33** with various substituents at the 4-position of the pybox ligand and the *para*-position of the aryl group on nickel (Scheme 5). Plotting the reaction rates as a function of the substituents’ Hammett parameter σ_p_ revealed a volcano-shaped trend, where both electron-donating and electron-withdrawing substituents on the pybox and aryl ligands led to a decrease in the rate of radical capture (Figure 1A). The slopes for the pybox ligands are relatively shallow compared to those of the aryl actor ligands, suggesting that pybox exerts a minimal electronic effect on the rates. Analogous nonlinear Hammett trends have been observed previously in the oxidative addition of aryl and alkyl halides to nickel(0) and nickel­(I) complexes.^41^ Such trends may indicate either a mechanistic shift or a transition state influenced by multiple factors, which cannot be fully explained by a straightforward single-parameter linear correlation.

**Scheme 5:**
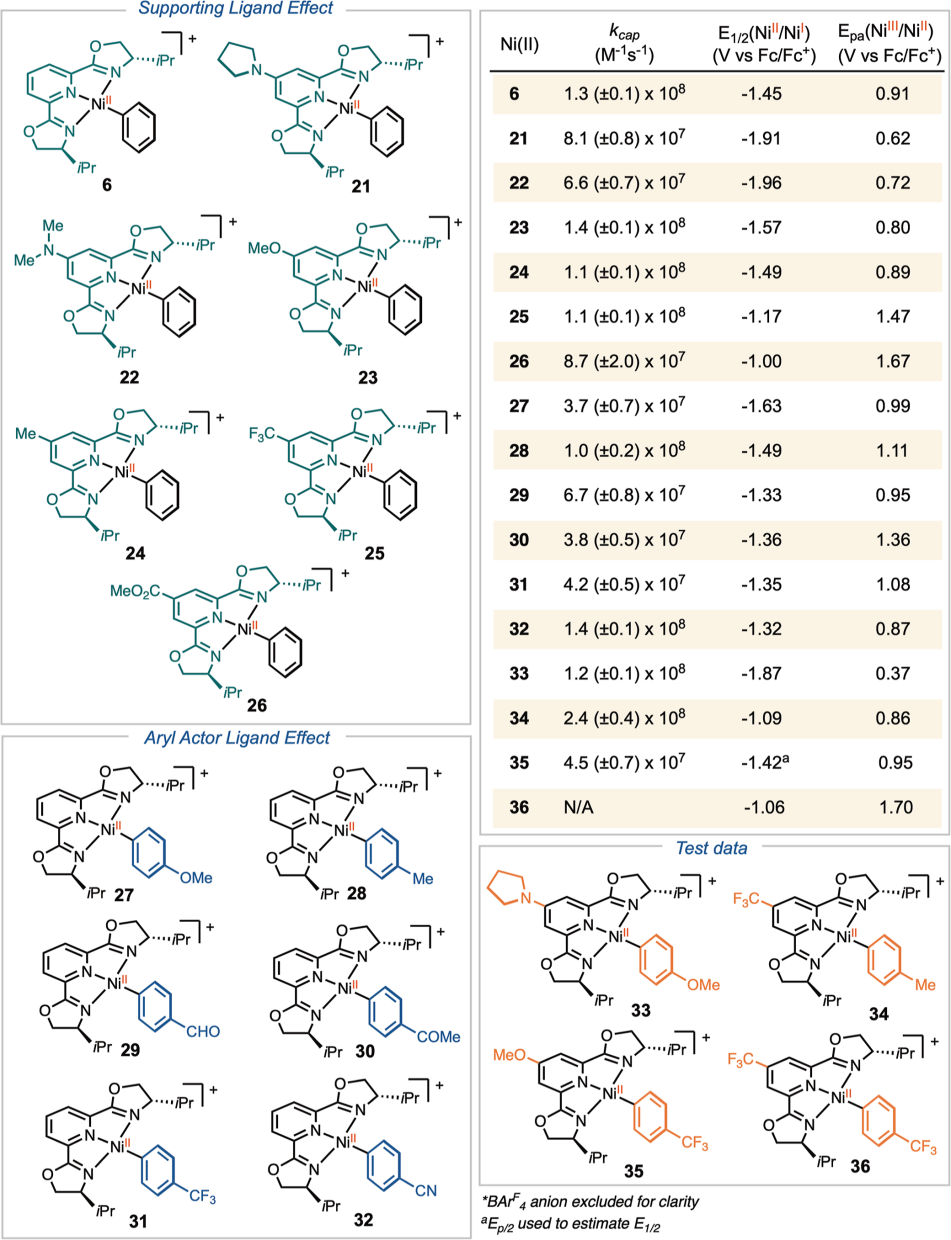
Radical Capture Rate and Electrochemical Data for Electronically Substituted [(^*i*Pr^pybox)­Ni­(Ar)]­BAr^F^_4_ Complexes

**Figure 1:**
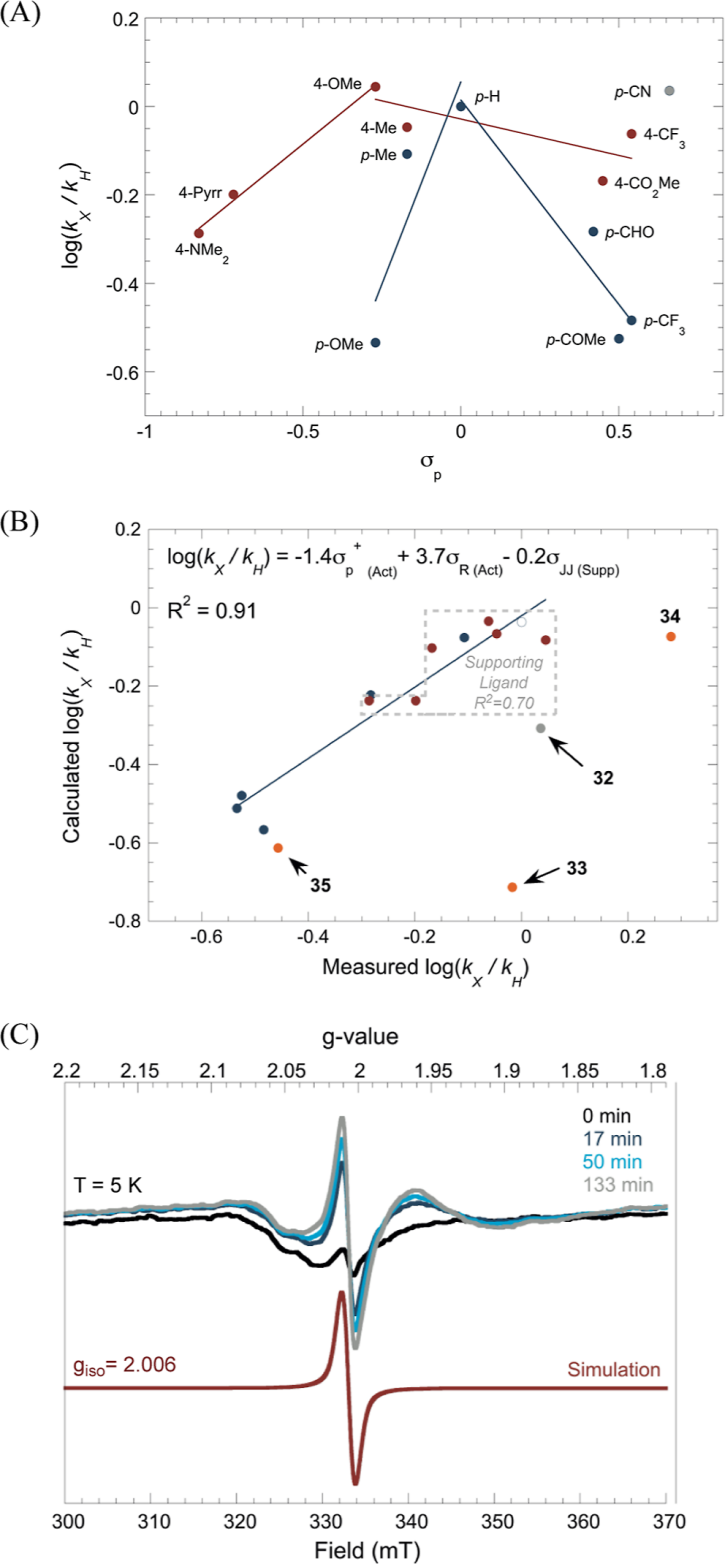
(A) Hammett plot of supporting (red) and actor (blue) ligand versus a single Hammett parameter; (B) fitting of kinetic data using multiple Hammett parameters as descriptors, with [(^*i*Pr^pybox)­Ni­(*p*-CN-C_6_H_4_)]­BAr^F^_4_ **32** shown in gray and [(4-pyrrolidinyl-^*i*Pr^pybox)­Ni­(*p*-MeO-C_6_H_4_)]­BAr^F^_4_ **33**, [(4-CF_3_-^*i*Pr^pybox)­Ni­(*p*-tol)]­BAr^F^_4_ **34**, and [(4-MeO-^*i*Pr^pybox)­Ni­(*p*-CF_3_–C_6_H_4_)]­BAr^F^_4_ **35** shown in orange; (C) X-Band EPR spectrum recorded at 5 K in THF under 390 nm irradiation of 20 mM [(4-pyrrolidinyl-^iPr^pybox)­Ni­(*p*-MeO-C_6_H_4_)]­BAr^F^_4_ **33** in the presence of 5 equiv of **1**. The EPR spectrum was collected with the following settings: modulation frequency = 100.00 kHz, modulation amplitude = 10.00 G, power = 0.4743 mW.

Complex **32**, bearing a *para-*cyano group on the aryl ring, deviated from the observed trend, prompting additional control experiments. Upon light irradiation for 12 h, complex **32** underwent photodecomposition to form the corresponding biaryl product in 40% yield, whereas **6** remained stable. We therefore attribute the faster product formation from **32** to an alternative, faster radical–radical coupling pathway, likely involving photoexcited ejection of a stable *para-*cyanophenyl radical.^42,43^

Fitting the data using multiple Hammett parameters—including the pure-resonance constant σ_R_ [σ_R (Act)_],^44^ the positive charge stabilization constant σ_p_^+^ [σ_p_^+^_(Act)_] for the actor ligand,^44^ and the spin-delocalization constant σ_JJ_ for the supporting ligand [σ_JJ (Supp)_][Bibr ref45] —yielded a reasonable fit with an *R* ^2^ value of 0.91 (Figure 1B), with the exception of complex **32**, whose fast reaction rate remained an outlier. The data indicate that reaction rates are enhanced by stronger resonance stabilization and positive charge stabilization on the actor aryl ligand, as well as by radical stabilization through the supporting ligand. However, when we analyzed the effect of the supporting ligand alone, the fit was less robust, yielding an *R* ^2^ value of 0.70.

To further validate the Hammett model, we synthesized [(4-pyrrolidinyl-^*i*Pr^pybox)­Ni­(*p*-MeO-C_6_H_4_)]­BAr^F^_4_ **33**, [(4-CF_3_-^*i*Pr^pybox)­Ni­(*p*-tol)]­BAr^F^_4_ **34**, [(4-MeO-^*i*Pr^pybox)­Ni­(*p*-CF_3_–C_6_H_4_)]­BAr^F^_4_ **35**, and [(4-CF_3_-^*i*Pr^pybox)­Ni­(*p*-CF_3_–C_6_H_4_)]­BAr^F^_4_ **36**, which incorporate substituents on both the actor and supporting ligands. While both complexes **33** and **34** exhibited high rates of radical capture, complex **35** was comparatively slower. Among the series, complex **36** was the slowest, showing negligible nickel consumption and minimal product formation, preventing us from obtaining rate data. Upon irradiation of complex **33** in the presence of **1**, photo-EPR analysis revealed the buildup of benzyl radical without any detectable signals corresponding to metalloradical species (Figure 1C). While the radical capture rate of **35** is consistent, the rates for **33** and **34** do not align with the multi-Hammett model. This discrepancy suggests that a straightforward analysis of inductive and resonance effects is insufficient to fully capture the nuanced and potentially competing ligand electronic effects at play.

Recognizing the limitations of the Hammett parameters, we adopted a more comprehensive approach using a multivariate linear regression (MLR) model with computational descriptors to capture the complex free energy relationship and substitution patterns.^46^ To implement this tool, we performed DFT calculations to obtain descriptors that reflect the molecular properties of each [(^*i*Pr^pybox)­Ni­(Ar)]­BAr^F^_4_ complex and their corresponding pybox ligands. We first optimized the gas-phase geometry of each complex, from which a screen of basis sets and functionals identified BP86/def2-SVP as providing structures which best reflected the reported crystallographic data for [(^*i*Pr^pybox)­Ni­(Ph)]­BAr^F^_4_ **6** (Table S3).^36^ Analysis of the electronic structure of **6** revealed that the HOMO is nickel-centered, primarily composed of the d_*z*2_ orbital, consistent with previous studies of (bpy)­Ni­(Ar)­X complexes (Figure 2A).^47^ The LUMO consists of the π* orbital of pybox, consistent with previous findings that the reduction of complex **6** results in a ligand-centered radical.^36,48^

**Figure 2:**
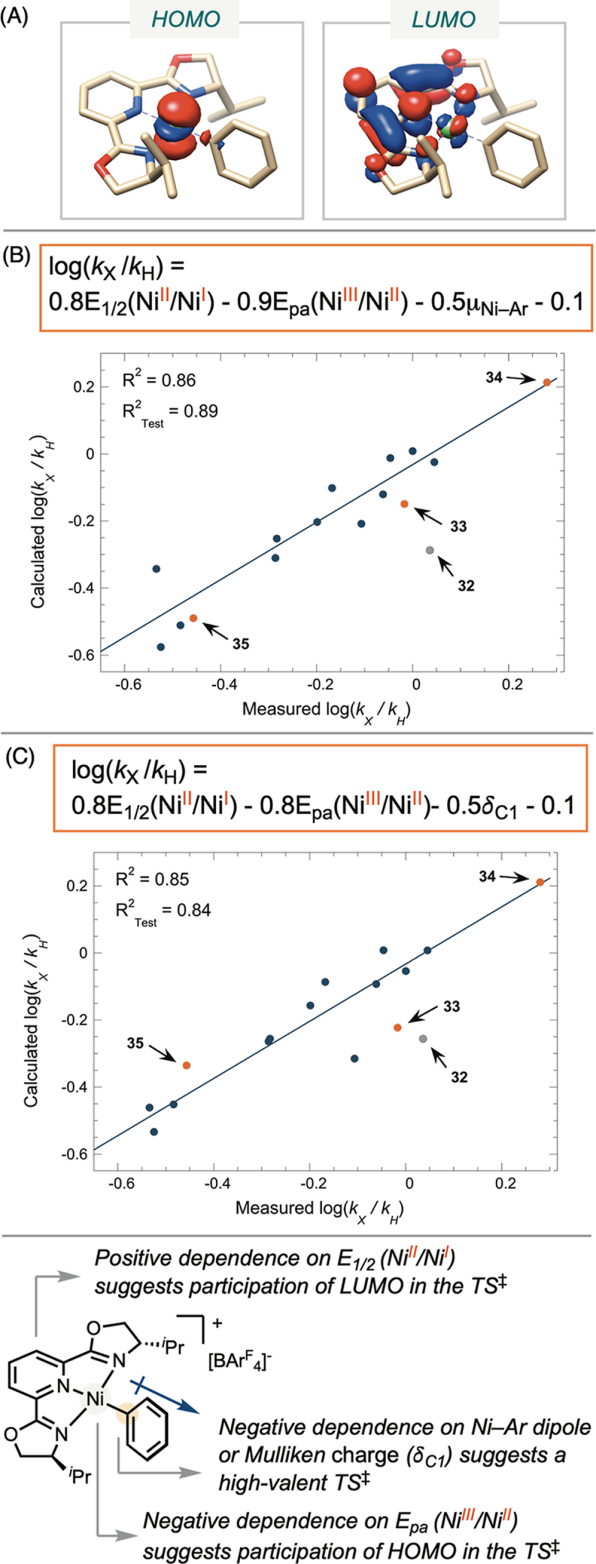
(A) Frontier molecular orbitals of complex **6** calculated using BP86/def2-SVP level of theory. (B) Multiparameter regression of radical capture rates at [(^iPr^pybox)­Ni­(Ar)]­BAr^F^_4_ complexes using redox potentials and the dipole moment (μ_Ni–Ar_) of the isolated nickel–aryl fragment calculated at B3LYP-D3/def2-TZVPP/CPCM­(THF) level of theory. (C) Multiparameter regression analysis using redox potentials and the Mulliken charge on the *ipso*-carbon calculated at B3LYP-D3/def2-SVP level of theory.

With the optimized structures we then generated computational descriptors with various levels of theory, incorporating dispersion corrections and a solvent model to obtain the most accurate descriptors (Tables S7–S9). We performed MLR analysis using the descriptors, including orbital energies, atomic charges, and bond orders, as well as experimental parameters, to fit the rate data (Figures S59–S62). Ultimately, we identified a robust fit using three parameters: the reduction potential *E*_1/2_(Ni^II^/Ni^I^), the oxidation potential *E*_pa_(Ni^III^/Ni^II^), and dipole moment (μ_Ni–Ar_) of the nickel-aryl fragment of the complex (*R* ^2^ = 0.86) (Figure 2B) or Mulliken charge (δ_C1_)­of the *ipso*-carbon attached to nickel (*R* ^2^ = 0.85) (Figure 2C).^49^ Further validation of the model using complexes **33**-**35** showed strong agreement with *R*_Test_^2^ values of 0.89 and 0.84, respectively. Notably, the data point for complex **34** falls within the extrapolation region of the fit, yet still exhibits excellent agreement with the model. To further confirm the model’s robustness, we performed cross-validation with a ∼70:30 random split of the data into train and validation sets. Repeating this process four times showed no significant variation in the coefficients or the *R* ^2^ value, further supporting the reliability of the fit (Figures S65 and S66).

The reduction potential *E*_1/2_(Ni^II^/Ni^I^) shows a strong correlation with the calculated LUMO energies of each complex (Figures S57–S58). The positive correlation between the radical capture rate and the ease of nickel­(II) reduction suggests that the LUMO plays a role in the transition state, with a more accessible LUMO leading to a faster reaction rate. In contrast, *E*_pa_(Ni^III^/Ni^II^) exhibited poor correlation with computationally derived HOMO energies (Figures S57 and S58). We attribute this to challenges in accurately modeling metal-centered orbitals computationally. The negative coefficient of *E*_pa_(Ni^III^/Ni^II^) in the linear fit suggests that a higher HOMO energy and the ease to remove an electron correlate with a faster reaction rate.

The negative coefficients for dipole moment (Figure 2B) and Mulliken charge (Figure 2C) both suggest that positive charge accumulates on nickel and the *ipso-*carbon bonded to nickel in the transition state. Qualitatively, this is reflected in the slow reaction rates observed for complexes **35** and **36** in the test data set, both of which feature electron-withdrawing groups on the reacting aryl group, consistent with transition state destabilization by the trifluoromethyl group. Collectively, the MLR analysis suggests involvement of both the HOMO and LUMO in the transition state, with partial positive charge developing at the active site.

The parameters identified in our fittings align with a recent study by Weix, Sigman, and co-workers on the overall selectivity of cross-electrophile coupling reactions.^40^ In that study, statistical modeling revealed a correlation between the formation of the target cross-coupling product and the chemical potential (average of HOMO and LUMO energies), as well as positive charge stabilization at the *ipso*-carbon. The nuanced electronic effect observed in our study indicates that radical capture is not simply accelerated by uniformly shifting electronic effects in one direction. Rather, it reflects a balance among the stabilization of LUMO, destabilization of HOMO, and the stabilization of partial positive charge buildup at the transition state.

### Mechanism of Nickel-Mediated Radical Capture and Carbon–Carbon Bond Formation

The data in this study enables us to differentiate among possible mechanisms for nickel-mediated radical capture and reductive elimination (Scheme 6). An outer-sphere concerted pathway, analogous to an S_H_2 mechanism,^50^ is considered unlikely. S_H_2 pathways are typically promoted by increased nucleophilicity of the incoming radical and the electrophilicity of the metal-bound carbon (Scheme 6A).^51^ However, we found that reactions involving more nucleophilic secondary alkyl radicals led to slower rates compared to those with primary alkyl radicals (Scheme 4C). Additionally, reactions involving electron-deficient nickel centers with electron-withdrawing substituents on the supporting ligands exhibited slower rates (Scheme 5A). These results rule out an outer-sphere concerted pathway.

**Scheme 6:**
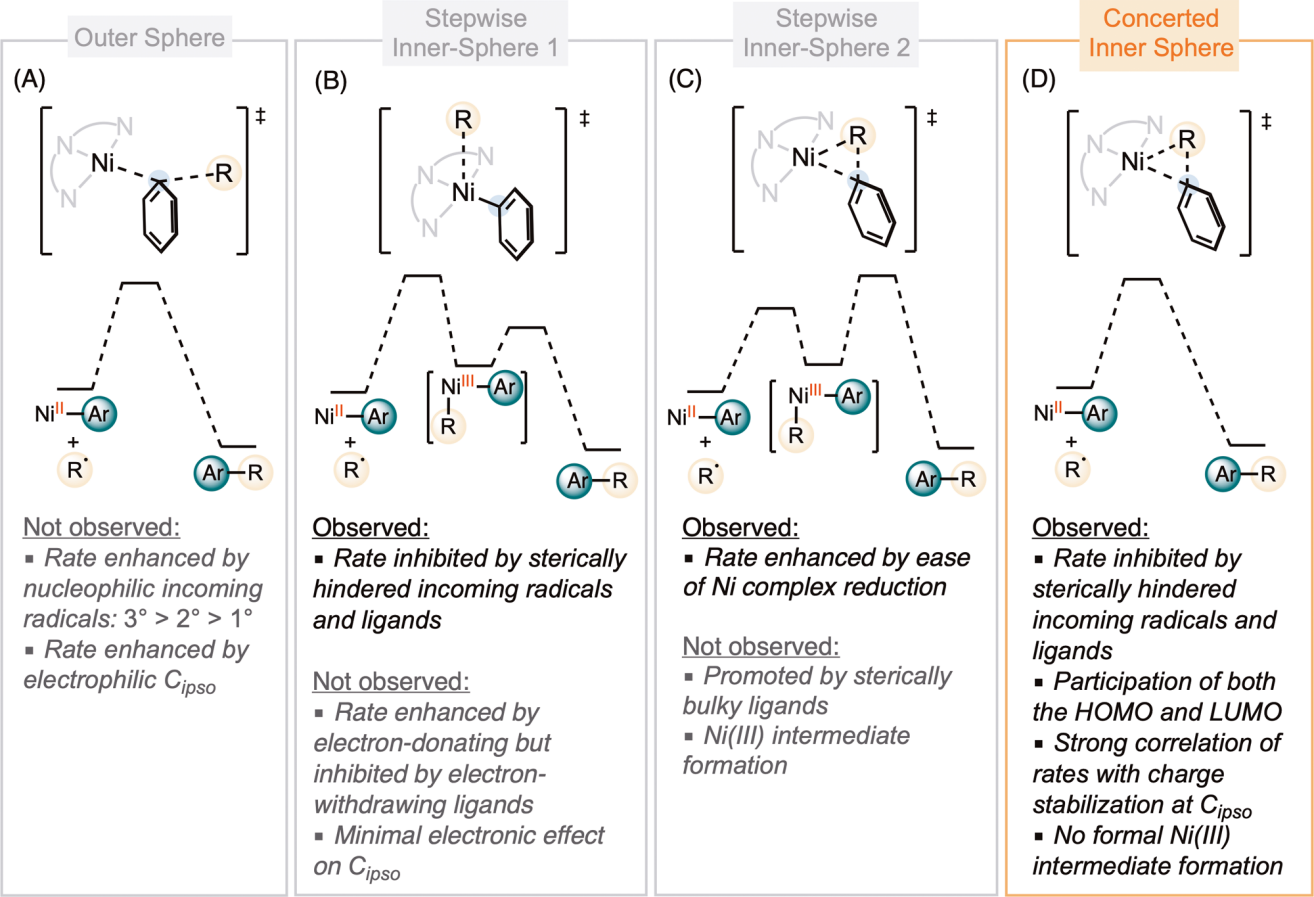
Mechanism of Nickel-Mediated Radical Capture and Carbon–Carbon Bond Formation and Expected Ligand Effect for Each Pathway

For a stepwise mechanism involving radical trapping followed by reductive elimination, two scenarios are possible. In the first scenario, the formation of a nickel­(III) intermediate is the rate-determining step, followed by rapid reductive elimination (Scheme 6B). This model predicts that radical capture would be sterically hindered by bulky aryl groups and incoming radicals, while electronically promoted by electron-donating ligands capable of stabilizing a high-valent nickel­(III) intermediate. Although the observed steric effects align with this model, the mixed electronic effects, particularly the positive dependence on the reduction potential of the complex E_1/2_(Ni^II^/Ni^I^), are inconsistent with this pathway. Furthermore, the rate-determining step does not involve bond formation or cleavage at the aryl ligand, which contradicts the strong correlation observed between the reaction rate and charge stabilization at the *ipso*-carbon bound to nickel.

Another possible scenario involves the rapid and reversible formation of nickel­(III) intermediate, followed by rate-determining reductive elimination (Scheme 6C). This pre-equilibrium pathway is partially consistent with the linear regression data, which suggest that a more positive *E*_1/2_(Ni^II^/Ni^I^) enhances the reaction rate. However, this mechanism does not account for the observed inhibition by steric hindrance, the negative dependence on *E*_pa_(Ni^III^/Ni^II^), and the stabilization of positive charge at the *ipso*-carbon of the aryl group. Moreover, photo-EPR spectroscopy did not detect any nickel­(III) intermediate, even for the most electron-rich derivative **33** (Figure 1C), providing additional evidence against this pathway. In comparison, in previous cases where reductive elimination was the rate-determining step for nickel­(III)-dialkyl complexes, the nickel­(III) intermediates were directly observable by EPR spectroscopy.^26,27^ Ruling out this pathway also verifies our assumption that radical trapping at nickel is irreversible.

The mechanism that best aligns with all observations is a concerted inner-sphere radical capture and carbon–carbon bond formation pathway (Scheme 6D). In this mechanism, the SOMO of the incoming radical interacts with both the LUMO and HOMO of the nickel complex, facilitating cleavage of the nickel–carbon bond and formation of the new carbon–carbon bond. This pathway is unique to radical reactions, as the SOMO can simultaneously donate electron density and accept electrons. This model predicts a balanced contribution from both the HOMO and LUMO, while demonstrating high steric sensitivity to both the incoming radical and the actor ligand. The modest steric effect of the pybox ligand also likely reflects this balance—reduced steric hindrance facilitates radical capture, while some degree of steric bulk may promote carbon–carbon bond formation. Moreover, the strong correlation between the reaction rate and positive charge stabilization at nickel and the aryl ligand’s *ipso*-carbon suggests direct involvement of the aryl group in the transition state, where the nickel center exhibits high-valent character.

This concerted mechanism accounts for the critical effect of redox-active ligands identified in prior methodology development. A survey of various ligand types reveals that only redox active ligands effectively promote radical capture and carbon–carbon bond formation (Scheme 2). This effect is more clearly visualized by plotting product yield against the LUMO energy of the nickel­(II) complexes, revealing two distinct regimes: complexes with high-energy LUMOs show low product yields, while those with low-energy LUMOs exhibit high yields of radical capture (Figure 3A). The rates for pybox are comparable to those observed for ^*t*Bu^bpy and phen (Figure 3B),^26^ suggesting that the mechanisms for bidentate and tridentate ligands may be analogous. The relatively shallow slopes for pybox in the Hammett plot (Figure 1A) indicate that substituents exert minimal electronic effects. These findings are consistent with the small electronic effects of ligands on the redox potentials of nickel complexes.^52^

**Figure 3:**
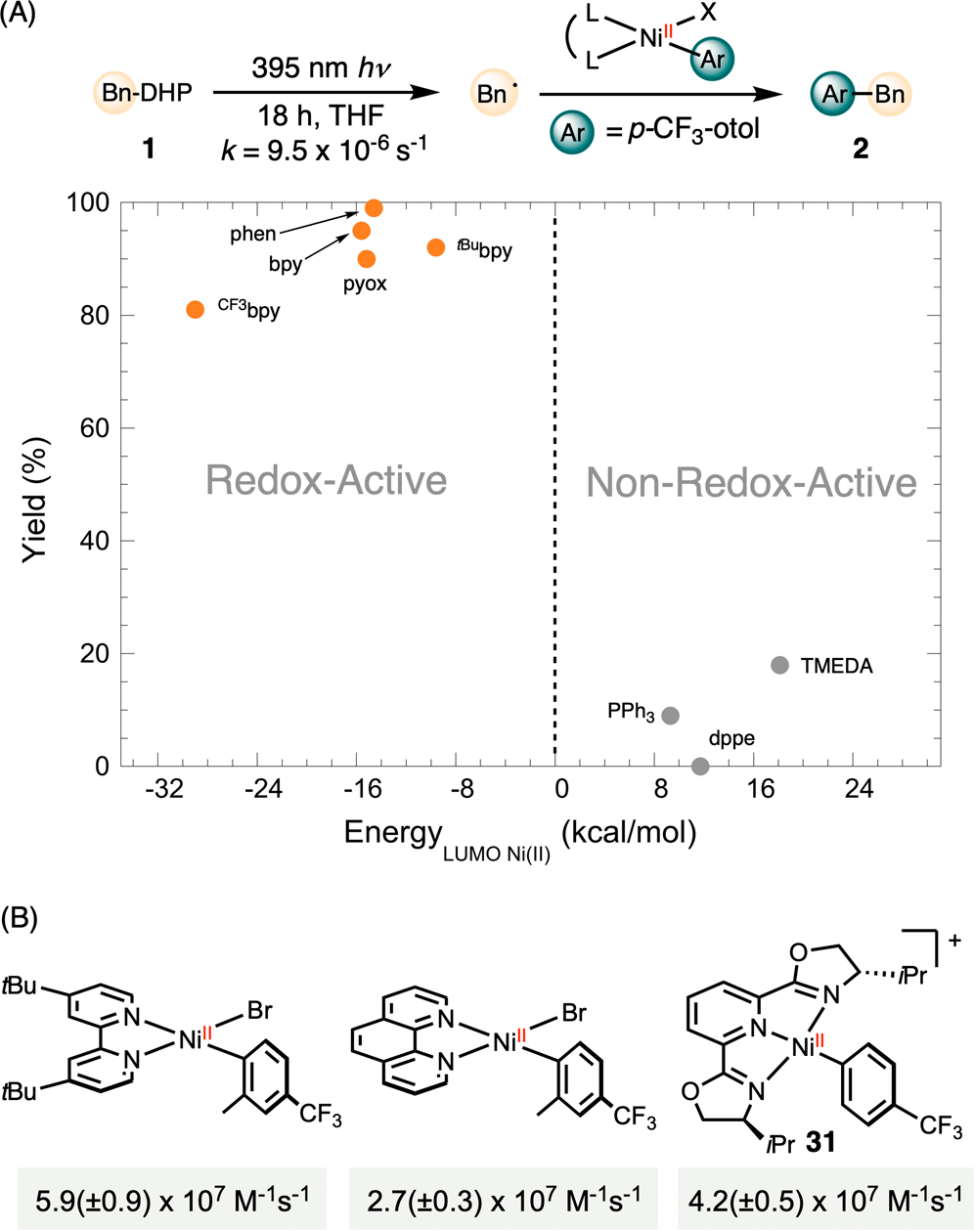
Comparison of redox-active and nonredox-active ligands with −65 kcal/mol Energy_LUMO Ni(II)_ as the zero-point (A) and bidentate and tridentate ligands (B).

## Conclusion

In conclusion, our study on nickel-mediated radical capture and carbon–carbon bond formation provides valuable insights into nickel-catalyzed cross-coupling. While phosphine and amine ligands are ineffective, bidentate and tridentate nitrogen π-acceptor ligands promote efficient radical capture and subsequent carbon–carbon bond formation. The observed steric effect and nonlinear Hammett trends contradict outer-sphere and stepwise inner-sphere pathways. MLR analysis and photo-EPR spectroscopy reveal a concerted inner-sphere mechanism that proceeds without the formation of a discrete nickel­(III) intermediate. The concerted transition state involves a nickel-centered HOMO [oxidation potential *E*_pa_(Ni^III^/Ni^II^)], a supporting ligand-centered LUMO [reduction potential *E*_1/2_(Ni^II^/Ni^I^)], and positive charge buildup at nickel and the *ipso*-carbon of the aryl actor ligand. The participation of the LUMO accounts for the success of redox-active ligands, which aligns with discoveries from catalytic reaction optimization. This mechanistic insight provides a foundation for the rational design of ligands to enhance the efficiency and selectivity of nickel-catalyzed cross-coupling reactions involving carbon radical intermediates.

## Supplementary Material



## Notes

Detailed experimental procedures, characterization data, statistical models, and details of DFT calculations (PDF)
