# Supplementary material for: Nickel-Mediated Radical Capture: Evidence for a Concerted Inner-Sphere Mechanism
Source: J Am Chem Soc. 2025 May 30;147(23):19632–42. doi: 10.1021/jacs.5c01554 (PMC12164335; doi:10.1021/jacs.5c01554)
Supplement: Supplementary file 1 [file ja5c01554_si_001.pdf]

Supporting Information

Nickel-Mediated Radical Capture: Evidence for a Concerted Inner-Sphere Mechanism

Ethan H. Spielvogel, Jonathan Yuan, Norah M. Hoffmann* and Tianning Diao*

Department of Chemistry, New York University, 100 Washington Square East, New York, NY 10003, United States

E-Mail: diao@nyu.edu; norah.hoffmann@nyu.edu

Table of Contents

1. General information 1
2. Stoichiometric Radical Capture 2
3. Radical Clock Model 2
4. Radical Clock Measurement and Error Propagation 4
5. Radical Clock Control Experiment with Differing DHP Equivalents 15
6. In-situ Photo EPR Experiments 17
7. Synthesis and Characterization Data 18
8. Electrochemical Characterization 60
9. Tabulated Hammett Parameters and Relative Rate Data 79
10. Modeling Using Hammett Parameters 80
11. Computational Benchmarking and Workflow 81
12. Description of Computational Parameters 84
13. Tabulated Data on Computational Parameters 86
14. Evaluation of Computationally Determined MO Energies 91
15. Initial Data Modeling with Computational Parameters 92
16. Linear Regression Analysis with Mixed Experimental and Computational Descriptors 93
17. Test for Collinearity in Mixed Computational and Experimental Model 96
18. Cross Validation for Mixed Computational and Experimental Models 96
19. Complex LUMO Calculations for Redox Activity Series 100
20. Cartesian Coordinates of Optimized Structures 101
21. UV-Vis Spectra 149
22. FTIR Spectra 159
23. NMR Spectra 160
24. References 239

1. General information

General considerations. All air- and moisture-sensitive manipulations were carried out in a nitrogen-filled Vacuum Atmospheres glove box or under nitrogen atmosphere using standard Schlenk techniques. Reactions were monitored by thin-layer chromatography (TLC) on Merck TLC silica gel 60 F254 plates and compounds were visualized by UV light (254 nm and 365 nm) or staining with $KMnO_4$. Column chromatography was performed using silica gel (40-53 µm, 60 Å).

Materials. Commercial reagents were purchased from Oakwood Chemical, Sigma Aldrich, Strem Chemicals, Ambeed or Thermo Fischer scientific and used as received unless otherwise specified. Tetrahydrofuran (THF), toluene, diethyl ether (Et_2O), dichloromethane (DCM), acetonitrile (MeCN), and pentane were dried and deoxygenated by passing through alumina in a solvent purification system. Deuterated solvents ($CDCl_3$, CD_2Cl_2, C_6D_6, THF-d_8, acetone-d_6) were purchased from Cambridge Isotope Laboratories, Inc. either in ampoule vials and stored over activated 4 Å molecular sieves, or dried over sodium metal, distilled and freeze-pump-thawed prior to use.

Instrumentation. Nuclear magnetic resonance (NMR) spectra were recorded on Bruker 400 MHz, 500 MHz, or 600 MHz Avance. Chemical shifts for proton are reported in ppm relative to tetramethylsilane, with the residual solvent resonance ($CDCl_3$, δ = 7.26 ppm; CD_2Cl_2, δ = 5.32 ppm; C_6D_6, δ = 7.16 ppm; THF-d_8, δ = 1.72, 3.58 ppm; acetone-d_6, δ = 2.05) as the internal reference. Chemical shifts for carbon were reported in ppm relative to tetramethylsilane with the solvent resonance used as the internal reference ($CDCl_3$, δ = 77.16 ppm; CD_2Cl_2, δ = 53.84 ppm; C_6D_6, δ = 128.06 ppm; THF-d_8, δ = 25.31, 67.21 ppm; acetone-d_6, δ = 29.84, 206.26). Spectra are reported as follows: chemical shift (δ ppm), multiplicity (s = singlet, d = doublet, t = triplet, q = quartet, m = multiplet), coupling constant (Hz), and integration. High resolution mass spectra (HRMS) were recorded on an Agilent 6224 TOF LC/MS (ESI source). GC-MS data was obtained using a Shimadzu GCMS-TQ8040 with a Shimadzu SH-Rxi-5Sil MS column (L 30 m, ID 0.25, DF 0.25). GC data was obtained using a Shimadzu GC-2010 Plus with a Restek Rxi-5MS column (L 15 m, ID 0.25, DF 0.25). Fourier Transform Infrared (FTIR) spectra were recorded on a Nicolet iS50 FTIR spectrometer. UV-Vis spectra were collected using an Agilent Cary 3500 UV-Visible

Spectrophotometer. EPR (Electron Paramagnetic Resonance) measurements were conducted on a Bruker ELEXSYS E500 Spectrometer System in continuous-wave mode in the X-band frequency using a Bruker ER 4122SHQE resonator.

2. Stoichiometric Radical Capture

Stoichiometric evaluation of radical capture with diethyl 4-benzyl-1,4-dihydropyridine-3,5-dicarboxylate (BnDHP) **1** was carried out according to reported procedure.[1] In a nitrogen-filled glove box, a 2 mL crimp cap GC vial was charged with nickel complex **7-10** (10 μmol) and BnDHP **1** (5.0 equiv). After addition of THF (1.0 mL) and a magnetic stirring flea, the vial was crimped and removed from the glove box. The reaction mixture was irradiated for 18 h at 30 °C with 395 nm light using a Penn PhD photoreactor M2. n-Tetradecane (1.0 μL, 3.8 μmol) and/or hexafluorobenzene (1.0 μL, 8.7 μmol) was then added into the reaction mixture as an internal standard and the solution was diluted in DCM and filtered through a pad of celite, then analyzed by GCMS and/or ^{19}F-NMR.

Scheme S1. Stoichiometric radical capture experiment with nickel(II) and BnDHP **1**.

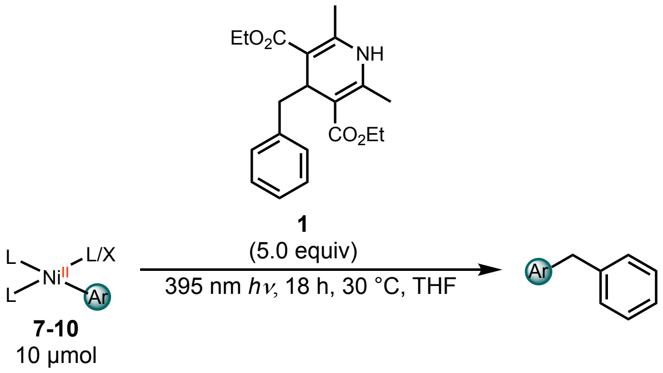

3. Radical Clock Model

We applied the radical clock substrate **11**[1] to determine radical trapping rates by analyzing its competition with radical cyclization.[2] Two scenarios are considered: in the first, where radical trapping by nickel is irreversible, the product ratio [**14**]/[**15**] is proportional to [k_{cap}]/[k_{cyc}]. In the second scenario, when radical trapping is reversible (Scheme S2), applying pre-equilibrium approximation (eq 1) allowed derivation of the product distribution (eq 3), which reflects the overall activation barrier, from the ground

state to the highest transition state (eq 4). In this case, the observed product ratio reflects changes in the barrier for reductive elimination.

Scheme S2. Mechanism and potential energy diagram for cyclized and uncyclized product formation.

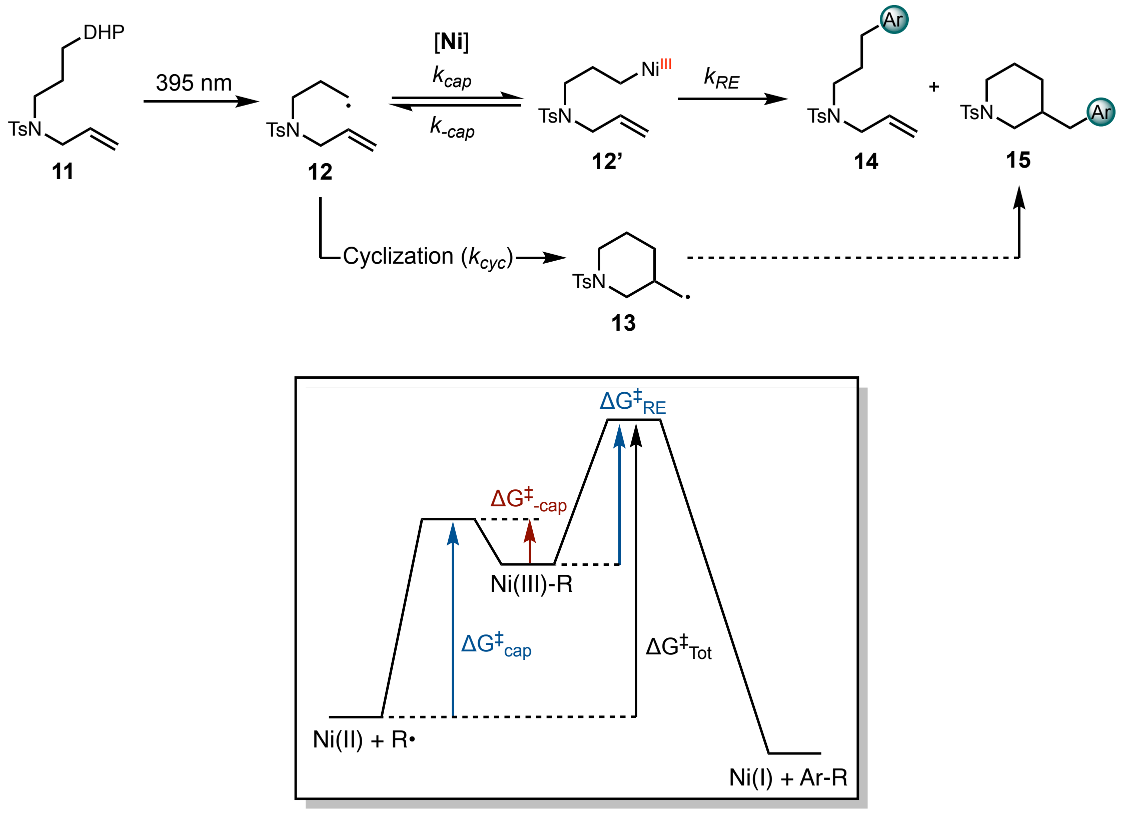

(1) $$\frac{d[\mathbf{14}]}{dt} = \frac{k_{cap} * k_{RE}}{k_{-cap}}[\boldsymbol{Ni}][\mathbf{12}]$$

(2) $$\frac{d[\mathbf{15}]}{dt} = k_{cyc}[\mathbf{12}]$$

(3) $$\frac{[\mathbf{14}]}{[\mathbf{15}]} = \frac{\frac{k_{cap} * k_{RE}}{k_{-cap}}[\boldsymbol{Ni}]}{k_{cyc}}$$

(4) $$k_{Tot} = \frac{k_{cap} * k_{RE}}{k_{-cap}}$$

(5) $$\Delta G^{\ddagger}_{Tot} = \Delta G^{\ddagger}_{cap} + \Delta G^{\ddagger}_{RE} - \Delta G^{\ddagger}_{-cap}$$

4. Radical Clock Measurement and Error Propagation

For radical clock experiments, nickel complexes were prepared fresh and used within 24 hours of synthesizing. Reaction mixtures were prepared by combining nickel and 2.3-5.2 equivalents of alkyl DHP **11** or **20** in a 2 mL crimp-cap vial, then diluting with THF to the desired concentration of nickel. The vials were sealed with crimp caps and placed into a Penn PHD photoreactor M2 and irradiated with 395 nm LED light at 30 °C. After 12 hours, the reaction mixture was opened to atmosphere, diluted with DCM, filtered through a pad of celite and analyzed by GC-FID and/or GCMS to obtain the ratio of **14** to **15**.

Scheme S3. Radical clock reaction scheme.

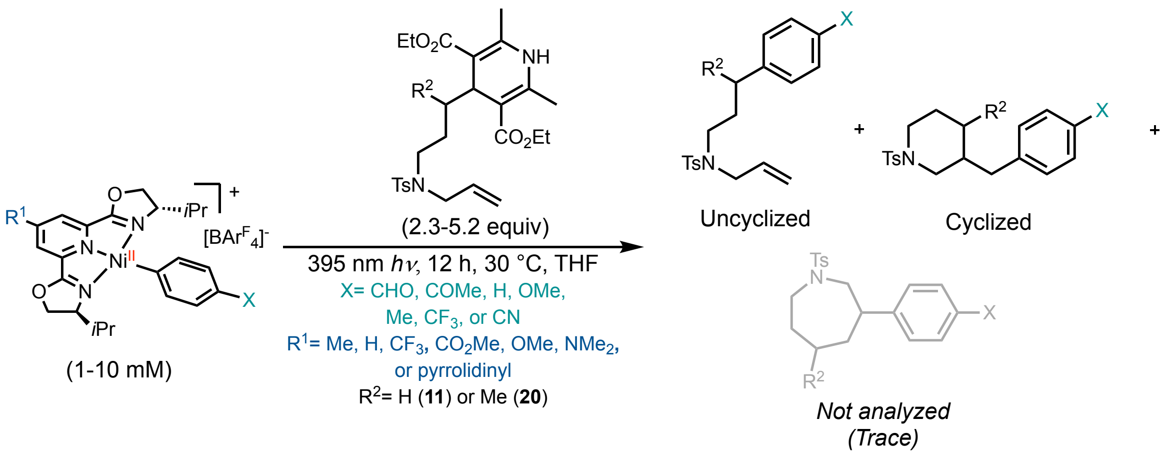

In the following section, the uncertainty in a calculated value "*x*" is denoted by u_x. The error in the slope (ρ) from the initial measurement of the uncyclized to cyclized product ratio across 4 or 5 different concentrations of nickel was propagated according to equations for calculating uncertainty.[3] The error for the slope of each linear regression was calculated using the LINEST function in Microsoft Excel. This error was then propagated with the uncertainty for the rate of cyclization, which was previously reported as k_{cyc}= 2.8 (±0.1) x 10^5 s^{-1} for primary and k_{cyc}= 3.8 (±0.3) x 10^5 s^{-1} for secondary alkyl radical,[1] to obtain the error in the radical capture rate.

Table S1. Tabulated data for radical clock plots and rates with uncertainties

Complex	Radical Clock	ρ	u_{ρ}	k_{cap} ($M^{-1}s^{-1}$)	u_{kcap}
**6**	Primary	458.87	38.21	1.28×10^{8}	0.12×10^{8}
**6**	Secondary	12.17	0.81	4.57×10^{6}	0.46×10^{6}
**21**	Primary	289.41	25.92	8.10×10^{7}	0.78×10^{7}
**22**	Primary	236.45	24.76	6.62×10^{7}	0.73×10^{7}
**23**	Primary	507.99	45.63	1.42×10^{8}	0.14×10^{8}
**24**	Primary	410.27	48.42	1.15×10^{8}	0.14×10^{8}
**25**	Primary	397.46	4.59	1.11×10^{8}	0.04×10^{8}
**26**	Primary	310.26	70.55	8.69×10^{7}	2.0×10^{7}
**27**	Primary	133.74	24.78	3.74×10^{7}	0.71×10^{7}
**28**	Primary	357.13	65.12	1.00×10^{8}	0.19×10^{8}
**29**	Primary	238.27	28.09	6.67×10^{7}	0.82×10^{7}
**30**	Primary	136.29	17.05	3.82×10^{7}	0.50×10^{7}
**31**	Primary	149.83	15.50	4.20×10^{7}	0.46×10^{7}
**32**	Primary	494.90	19.05	1.39×10^{8}	0.07×10^{8}
**33**	Primary	441.01	9.60	1.23×10^{8}	0.05×10^{8}
**34**	Primary	872.02	129.24	2.44×10^{8}	0.37×10^{8}
**35**	Primary	159.58	24.43	4.47×10^{7}	0.70×10^{7}
**19**	Primary	548.99	15.77	1.54×10^{8}	0.07×10^{8}

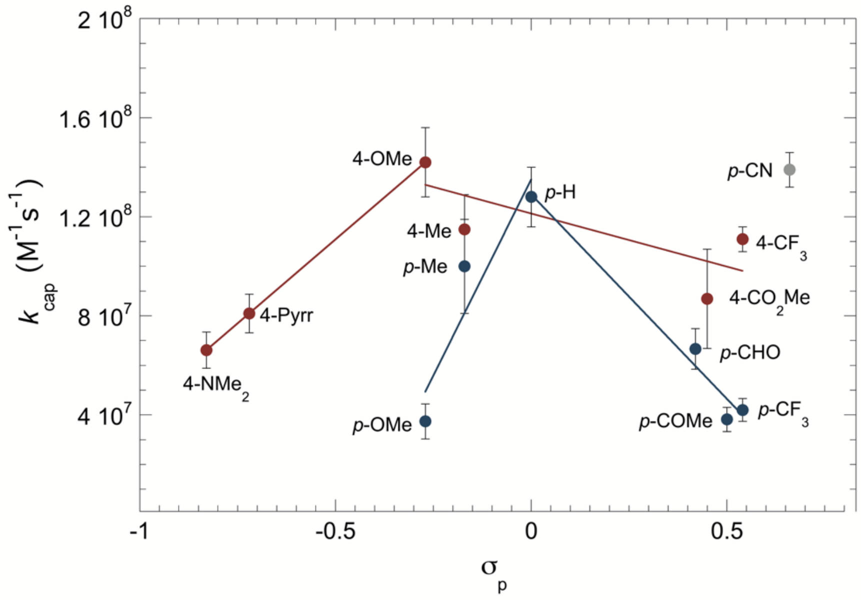

Figure S1. Plotted radical capture rates and uncertainties for complexes with varied electronics on the actor (blue) and supporting (red) ligand. Complex **32** is shown as an outlier with a grey marker.

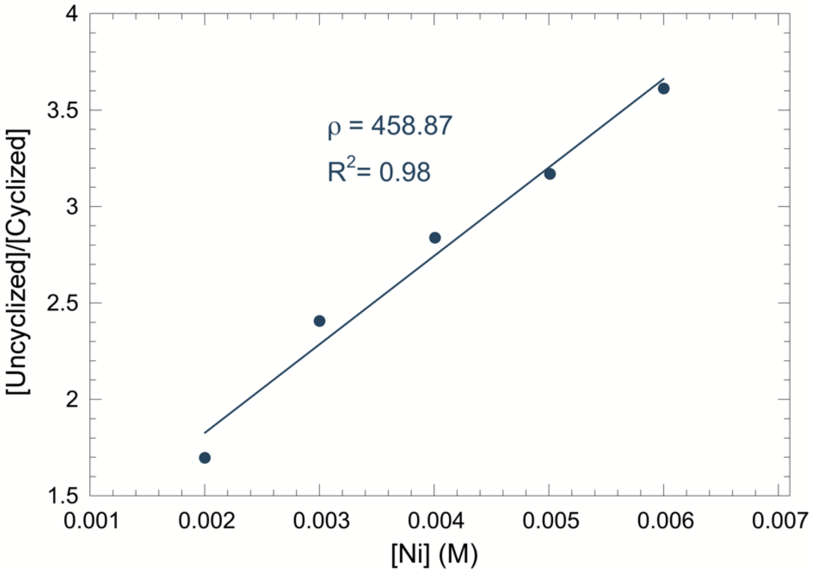

[Ni] (M)	[Uncyclized]/ [Cyclized]
0.00600	3.61
0.00501	3.17
0.00401	2.84
0.00300	2.41
0.00200	1.70

Figure S2. Primary radical clock plot for [($^{i\text{Pr}}$pybox)Ni(Ph)]BAr$^F_4$ **6** with 5.0 equivalents of alkyl-DHP **11**.

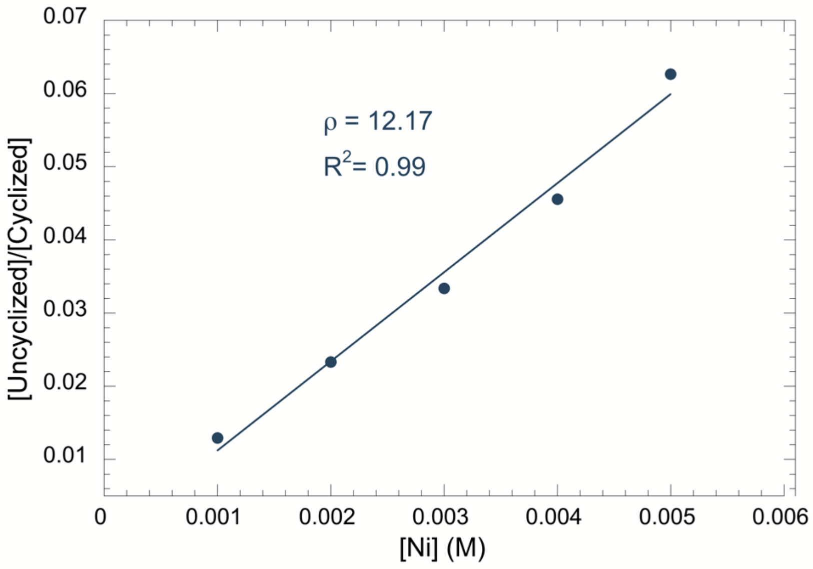

[Ni] (M)	[Uncyclized]/ [Cyclized]
0.00506	0.0626
0.00401	0.0456
0.00300	0.0334
0.00200	0.0233
0.00100	0.0129

Figure S3. Secondary radical clock plot for [(^{iPr}pybox)Ni(Ph)]BAr$^F{}_4$ **6** with 2.5 equivalents of alkyl-DHP **20**.

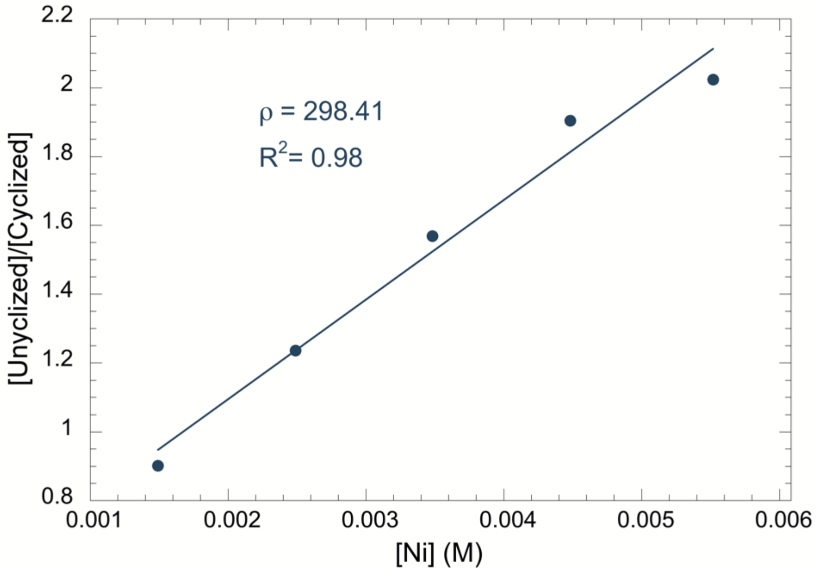

[Ni] (M)	[Uncyclized]/ [Cyclized]
0.00552	2.02
0.00448	1.90
0.00348	1.57
0.00249	1.24
0.00149	0.90

Figure S4. Primary radical clock plot for [(4-Pyrrolidinyl-^{iPr}pybox)Ni(Ph)]BAr$^F{}_4$ **21** with 2.5 equivalents of alkyl-DHP **11**.

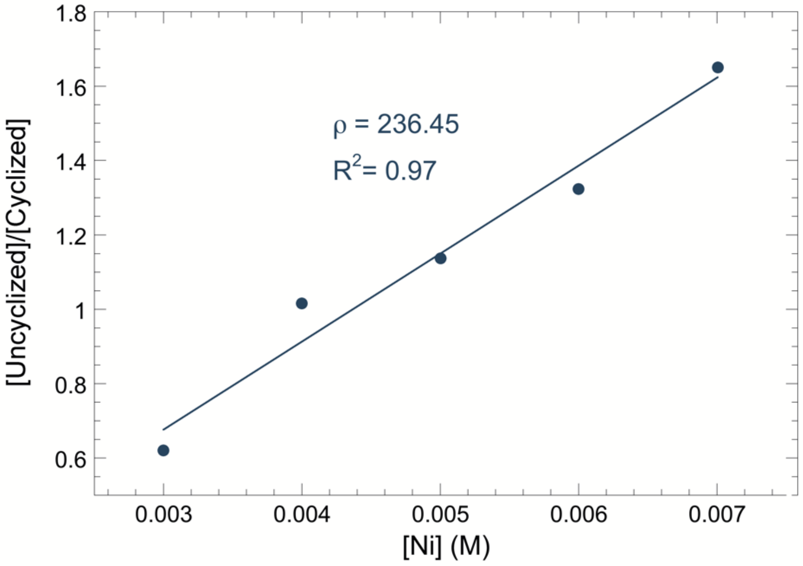

[Ni] (M)	[Uncyclized]/[Cyclized]
0.00701	1.65
0.00600	1.32
0.00500	1.14
0.00400	1.02
0.00300	0.62

Figure S5. Primary radical clock plot for [(4-NMe$_2$-^{iPr}pybox)Ni(Ph)]BAr$^F{}_4$ **22** with 2.5 equivalents of alkyl-DHP **11**.

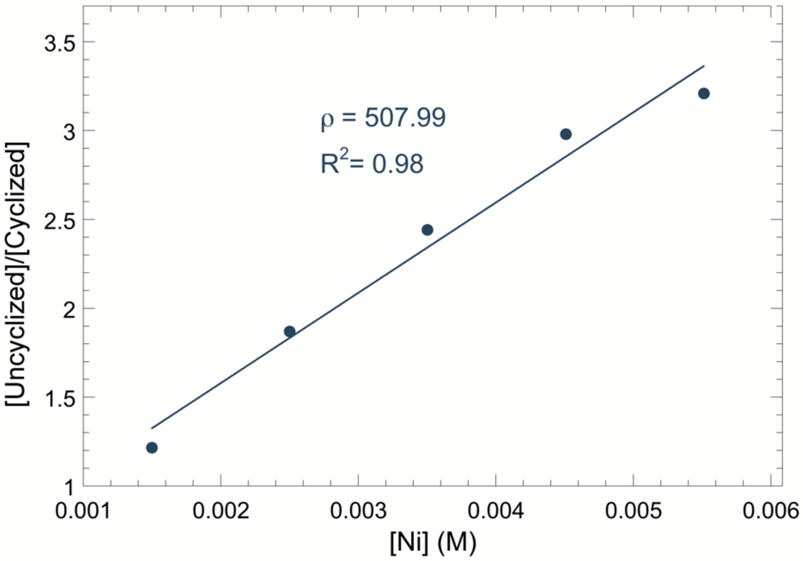

[Ni] (M)	[Uncyclized]/[Cyclized]
0.00551	3.21
0.00451	2.98
0.00350	2.44
0.00250	1.87
0.00150	1.22

Figure S6. Primary radical clock plot for [(4-OMe-^{iPr}pybox)Ni(Ph)]BAr$^F{}_4$ **23** with 5.0 equivalents of alkyl-DHP **11**.

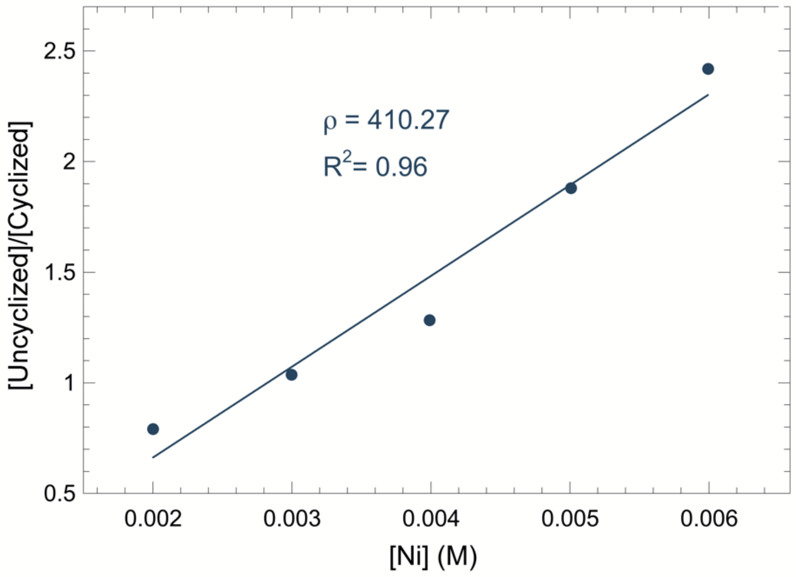

[Ni] (M)	[Uncyclized]/ [Cyclized]
0.00599	2.42
0.00501	1.88
0.00399	1.28
0.00300	1.04
0.00200	0.79

Figure S7. Primary radical clock plot for [(4-Me-$^{i\text{Pr}}$pybox)Ni(Ph)]BAr$^{F}_{4}$ **24** with 5.0 equivalents of alkyl-DHP **11**.

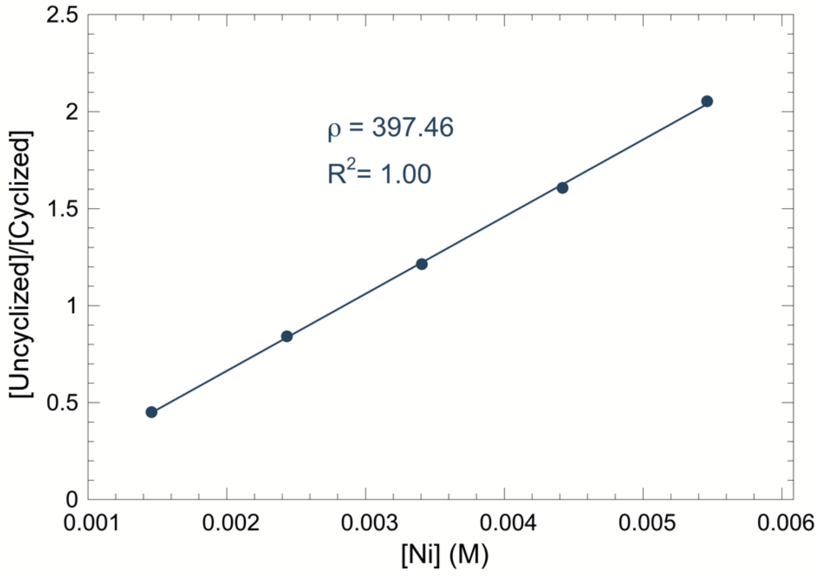

[Ni] (M)	[Uncyclized]/ [Cyclized]
0.00546	2.05
0.00442	1.61
0.00340	1.21
0.00243	0.84
0.00146	0.45

Figure S8. Primary radical clock plot for [(4-CF_3-$^{i\text{Pr}}$pybox)Ni(Ph)]BAr$^{F}_{4}$ **25** with 2.6 equivalents of alkyl-DHP **11**.

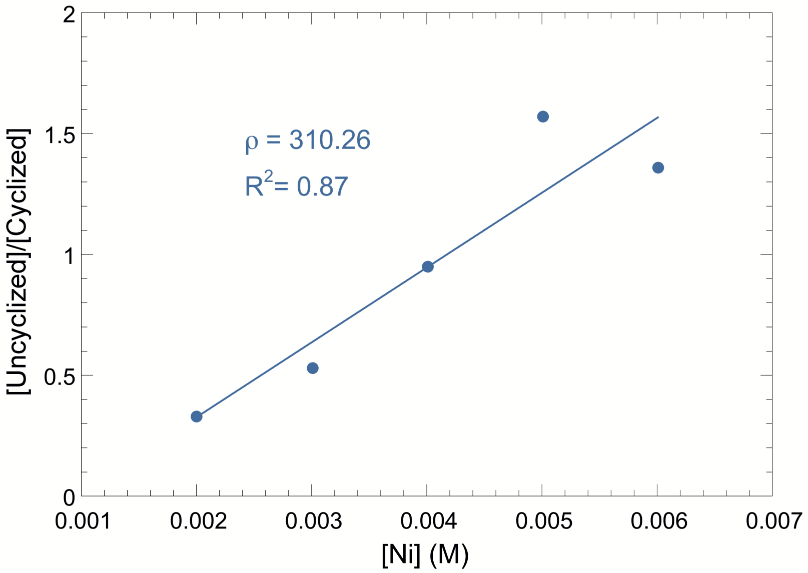

[Ni] (M)	[Uncyclized]/ [Cyclized]
0.00601	1.36
0.00501	1.57
0.00401	0.95
0.00301	0.53
0.00200	0.33

Figure S9. Primary radical clock plot for [(4-CO_2Me-^{iPr}pybox)Ni(Ph)]BAr^F_4 **26** with 5.1 equivalents of alkyl-DHP **11**.

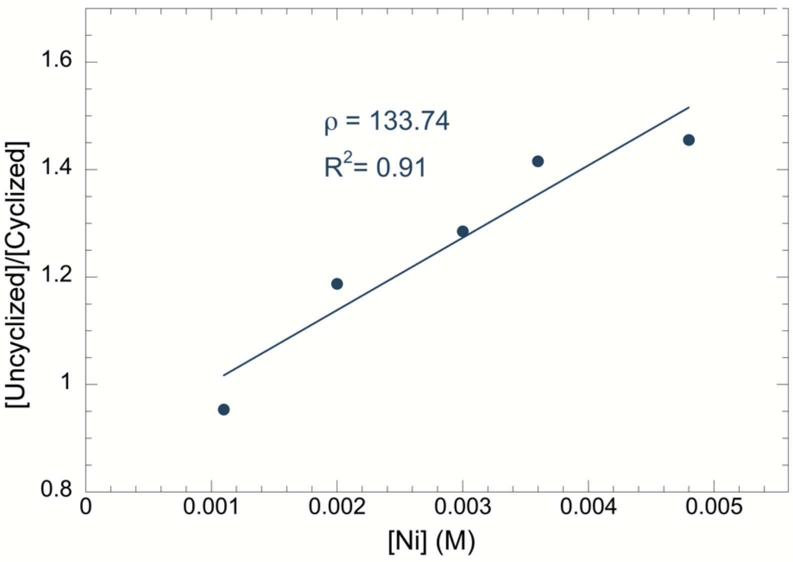

[Ni] (M)	[Uncyclized]/ [Cyclized]
0.00483	1.46
0.00356	1.42
0.00301	1.29
0.00200	1.19
0.00110	0.95

Figure S10. Primary radical clock plot for [(^{iPr}pybox)Ni(*p*-OMe-C_6H_4)]BAr^F_4 **27** with 5.0 equivalents of alkyl-DHP **11**.

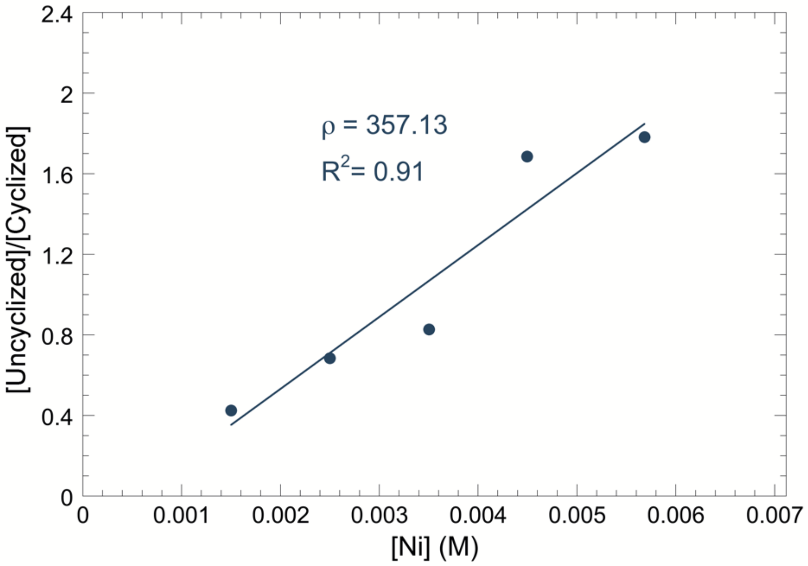

[Ni] (M)	[Uncyclized]/ [Cyclized]
0.00568	1.78
0.00450	1.69
0.00350	0.83
0.00250	0.68
0.00150	0.43

Figure S11. Primary radical clock plot for [($^{i\text{Pr}}$pybox)Ni(*p*-tol)]BAr$^{F}_{4}$ **28** with 2.5 equivalents of alkyl-DHP **11**.

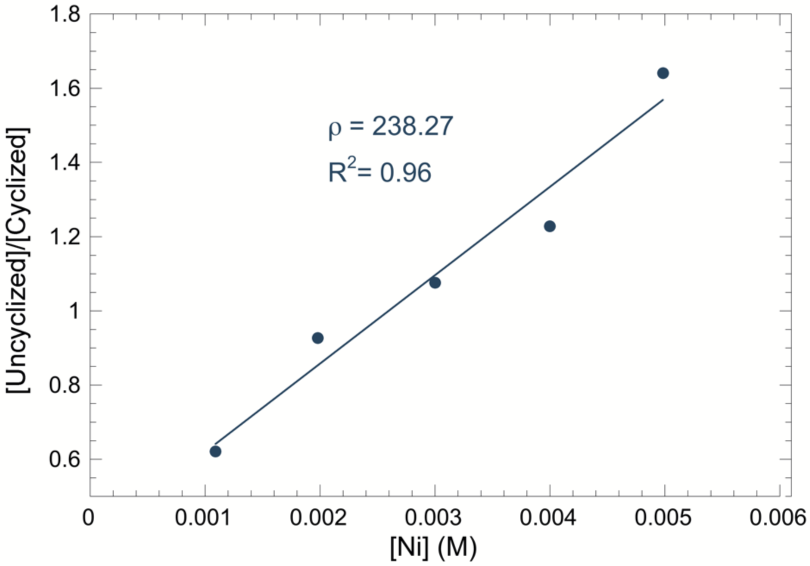

[Ni] (M)	[Uncyclized]/ [Cyclized]
0.00498	1.64
0.00400	1.23
0.00300	1.08
0.00198	0.93
0.00109	0.62

Figure S12. Primary radical clock plot for [($^{i\text{Pr}}$pybox)Ni(*p*-CHO-C_6H_4)]BAr$^{F}_{4}$ **29** with 5.0 equivalents of alkyl-DHP **11**.

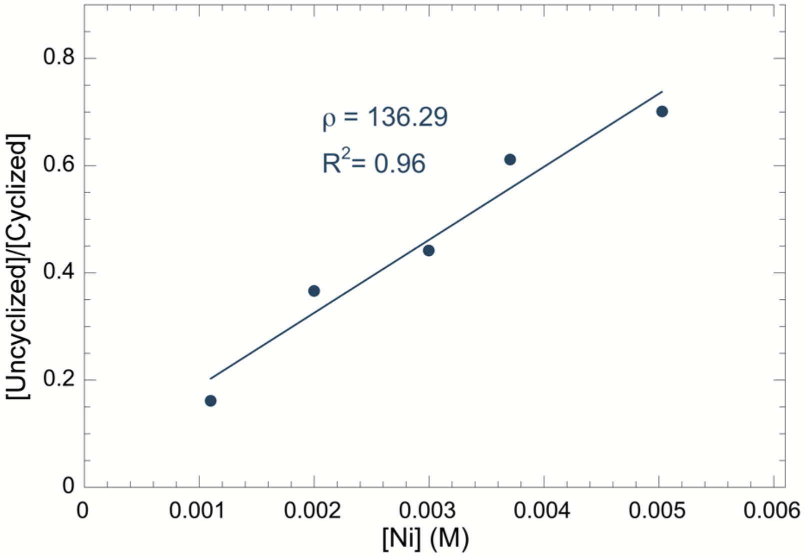

[Ni] (M)	[Uncyclized]/ [Cyclized]
0.00503	0.702
0.00370	0.612
0.00300	0.442
0.00200	0.367
0.00110	0.162

Figure S13. Primary radical clock plot for [($^{i\text{Pr}}$pybox)Ni(p-COMe-C_6H_4)]$BAr^F{}_4$ **30** with 5.0 equivalents of alkyl-DHP **11**.

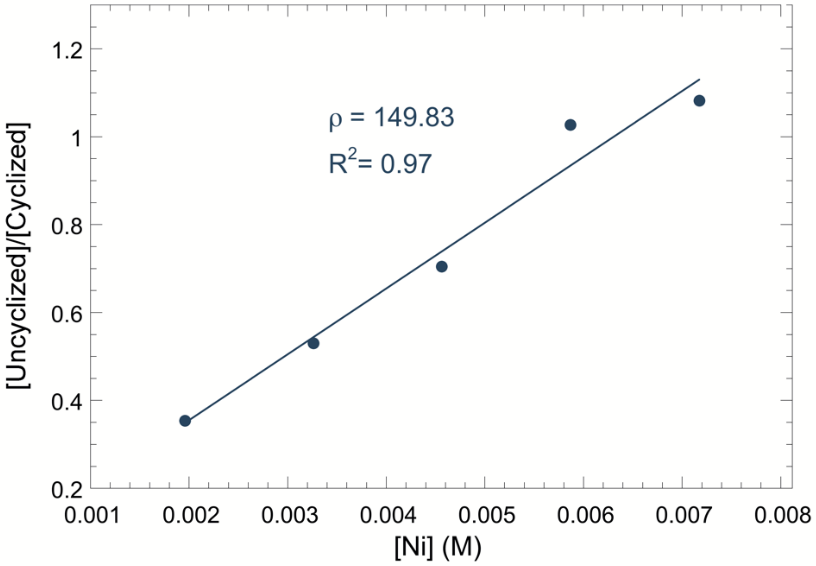

[Ni] (M)	[Uncyclized]/ [Cyclized]
0.00717	1.08
0.00587	1.03
0.00456	0.70
0.00326	0.53
0.00196	0.35

Figure S14. Primary radical clock plot for [($^{i\text{Pr}}$pybox)Ni(p-CF_3-C_6H_4)]$BAr^F{}_4$ **31** with 2.5 equivalents of alkyl-DHP **11**.

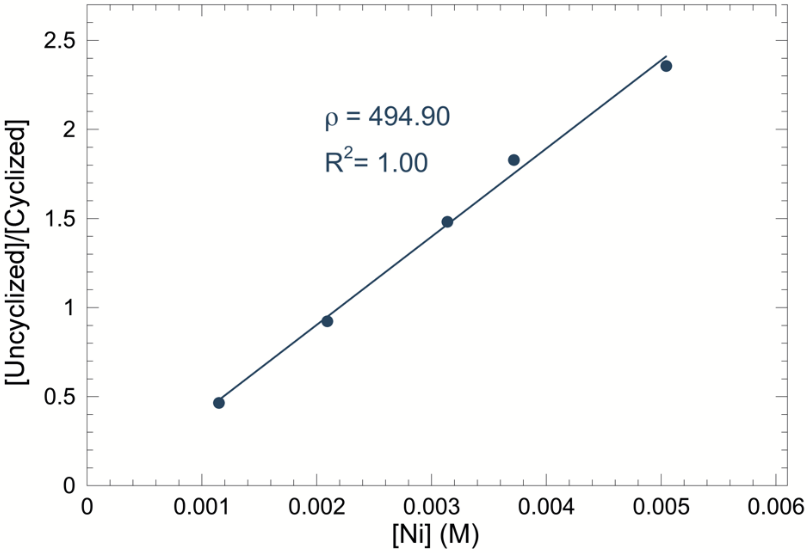

[Ni] (M)	[Uncyclized]/ [Cyclized]
0.00504	2.36
0.00372	1.83
0.00314	1.48
0.00209	0.92
0.00115	0.47

Figure S15. Primary radical clock plot for [($^{i\text{Pr}}$pybox)Ni(*p*-CN-C_6H_4)]$BAr^F{}_4$ **32** with 5.0 equivalents of alkyl-DHP **11**.

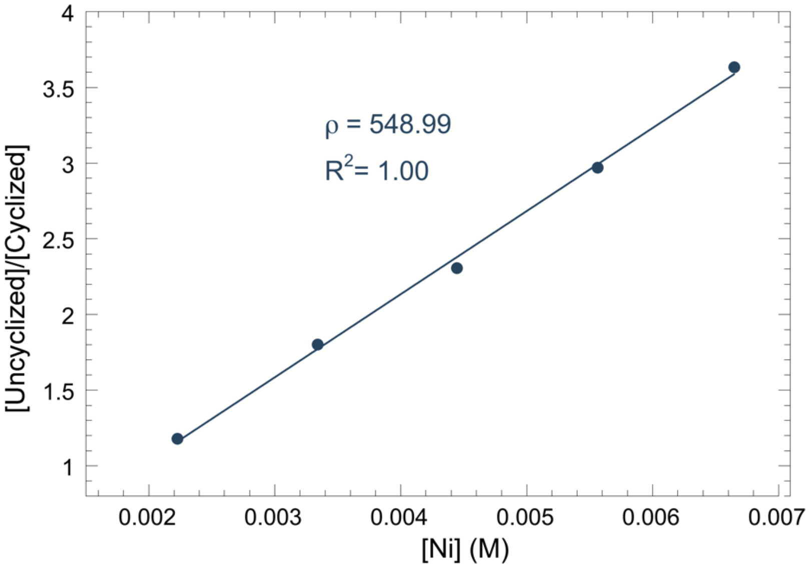

[Ni] (M)	[Uncyclized]/ [Cyclized]
0.00665	3.63
0.00556	2.97
0.00445	2.31
0.00334	1.80
0.00223	1.18

Figure S16. Primary radical clock plot for [(^{Me}pybox)Ni(Ph)]$BAr^F{}_4$ **19** with 2.3 equivalents of alkyl-DHP **11**.

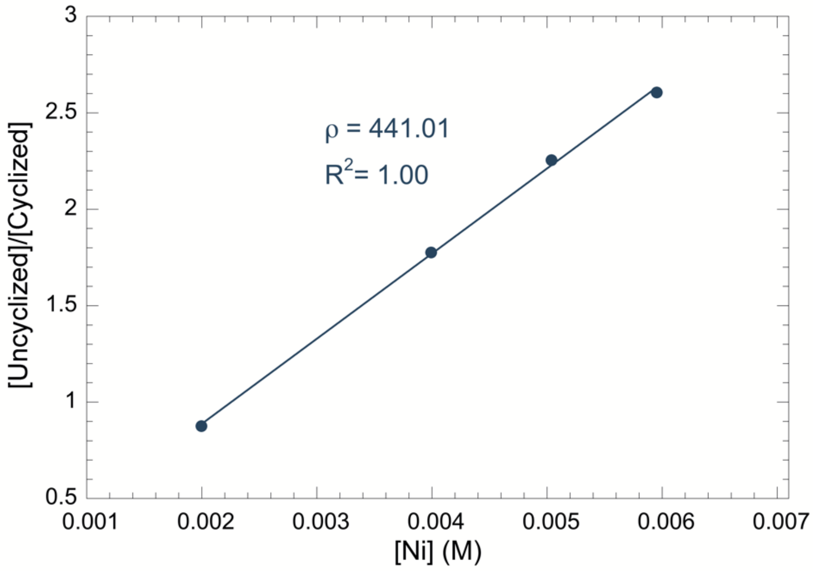

[Ni] (M)	[Uncyclized]/ [Cyclized]
0.00595	2.61
0.00504	2.25
0.00399	1.78
0.00200	0.88

Figure S17. Primary radical clock plot for [(4-Pyrrolidinyl-^{iPr}pybox)Ni(*p*-MeO-C_6H_4)]BAr^F_4 **33** with 2.5 equivalents of alkyl-DHP **11**.

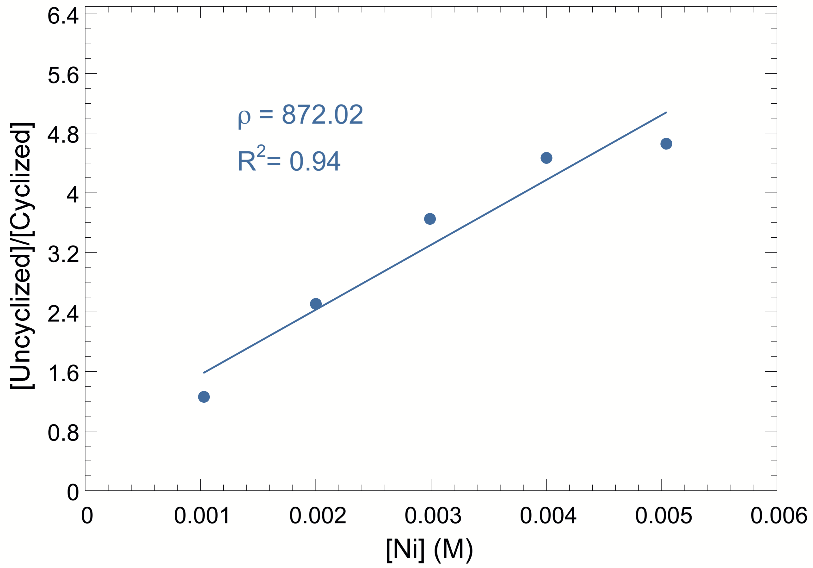

[Ni] (M)	[Uncyclized]/ [Cyclized]
0.00504	4.66
0.00400	4.47
0.00299	3.65
0.00200	2.51
0.00103	1.26

Figure S18. Primary radical clock plot for [(4-CF_3-^{iPr}pybox)Ni(*p*-tol)]BAr^F_4 **34** with 2.5 equivalents of alkyl-DHP **11**.

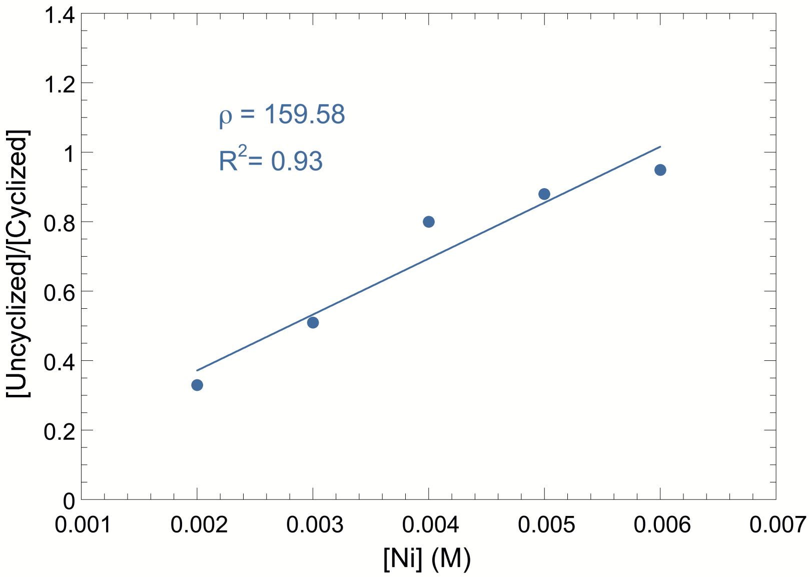

[Ni] (M)	[Uncyclized]/ [Cyclized]
0.00600	0.95
0.00500	0.88
0.00400	0.80
0.00300	0.51
0.00200	0.33

Figure S19. Primary radical clock plot for [(4-MeO-$^{i\text{Pr}}$pybox)Ni(*p*-CF_3-C_6H_4)]BAr^F_4 **35** with 5.2 equivalents of alkyl-DHP **11**.

5. Radical Clock Control Experiment with Differing DHP Equivalents

Due to differing rates of radical capture, 2.3-5.0 equivalents of alkyl DHP **11** or **20** were used. To ensure no difference in the measured rate constant with varying equivalents of alkyl DHP, a control experiment was carried out using ($^{t\text{Bu}}$bpy)Ni(2-methyl-4-(trifluoromethyl)phenyl)Br **S1** [($^{t\text{Bu}}$bpy)Ni(*p*-CF_3-*o*-tol)]. The rate of primary radical capture using 5.0 equiv **11** was previously determined to be 5.9 (±0.9) x 10^7 $M^{-1}s^{-1}$.[1] Using 2.5 equiv **11** resulted in a rate of 6.3 (±0.5) x 10^7 $M^{-1}s^{-1}$, which is within error of the previously measured value.

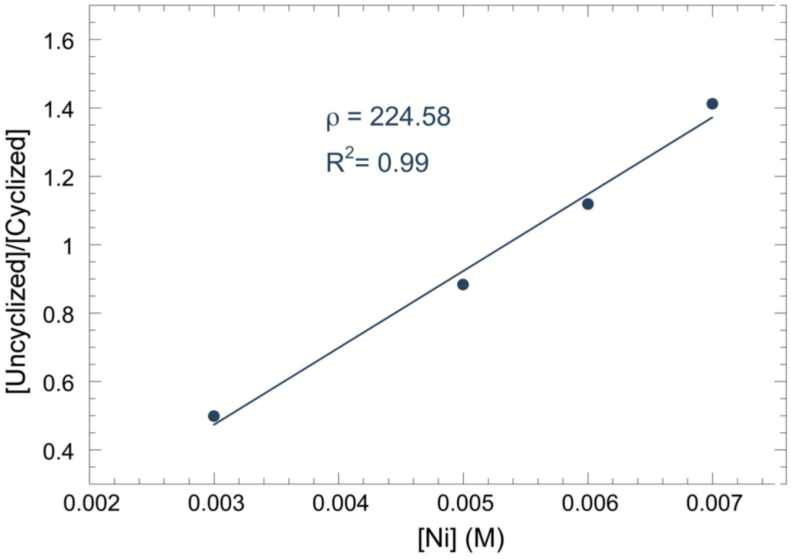

[Ni] (M)	[Uncyclized]/[Cyclized]
0.00700	1.41
0.00600	1.12
0.00500	0.88
0.00300	0.50

Figure S20. Primary radical clock plot for ($^{t\text{Bu}}$bpy)Ni(*p*-CF_3-*o*-tol) **S1** with 2.5 equivalents of alkyl-DHP **11.**

5. Complex 31 Photostability Test

Complex **32** was an outlier for rate measurements, with a faster than expected rate based on the Hammett plot and multivariate linear regression modeling. Bidentate nickel complexes with cyanoarene ligands have been shown to undergo aryl radical ejection readily under near-UV irradiation resulting in biaryl formation.[4] Cyanoarenes are also commonly employed in photoredox reactions as they readily form persistent radicals due to their low-lying LUMO.[5, 6] This is consistent with electrochemical data for **32**, which shows additional reversible reduction features not corresponding to Ni^{II}/Ni^{I} or Ni^{I}/Ni^{0} redox couple. We reasoned that due to the unusual stability of cyanoarene radical, complex **32** is capable of ejecting aryl radical and thus has access to a faster product formation pathway than canonical radical capture.

Scheme S4. Proposed additional pathway to product formation with complex **32**

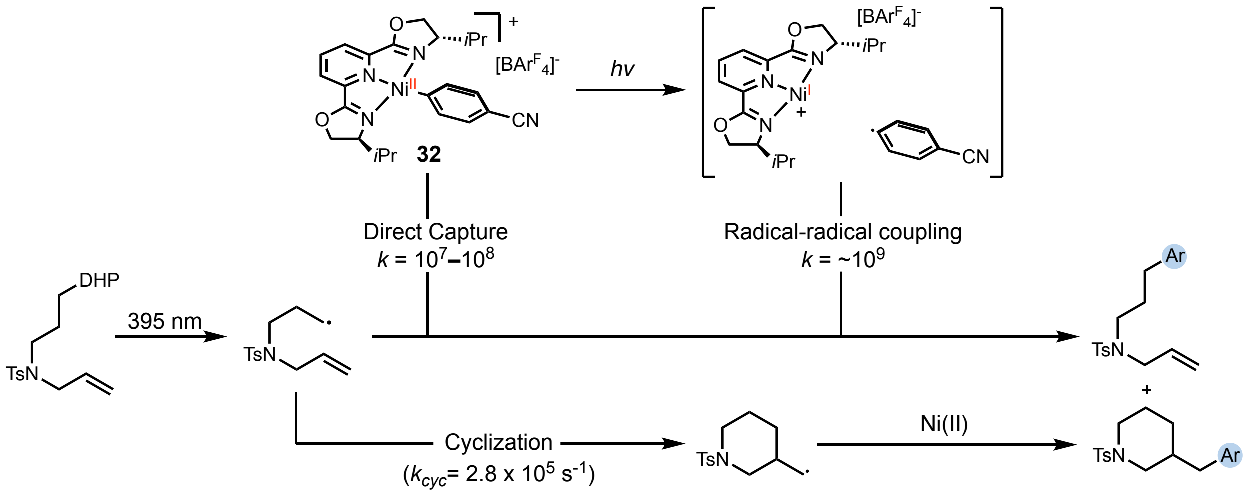

A photostability test was carried out with complexes **32** and **6** to validate the hypothesis. In a nitrogen-filled glove box, a 4 mL vial was charged with nickel complex **32** or **6** (10 µmol) and THF (1.0 mL). The vial was capped and sealed with electrical tape and removed from the glove box. The reaction mixture was irradiated for 12 h at 30 °C with 395 nm light using a Penn PhD photoreactor M2. After irradiation the samples were combined with trimethoxybenzene (1.7 mg, 8.7 µmol) internal standard, diluted in ethyl acetate and quenched with a small amount of de-ionized water. The organic layer was extracted and filtered through a pad of Na_2SO_4 and celite and analyzed by GCMS, with yields obtained by ^{1}H-NMR.

Scheme S5. Photostability test with complex **32** and **6**

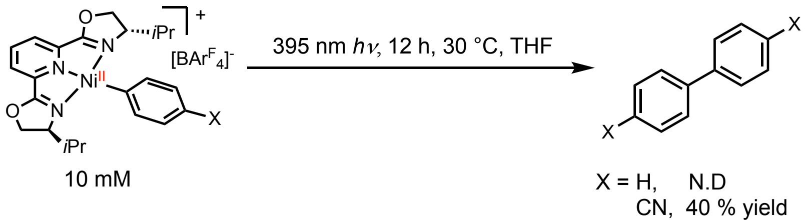

6. *In-situ* Photo EPR Experiments

The EPR instrument was connected to a 390 nm LED lamp via a fiber optic. The specifications of the light source are detailed in a previous study.[1] The EPR experiment was carried out at 5 K using the

following spectrometer settings: microwave frequency = 9.352964 GHz, center field = 3200 G, sweep width = 1600 G, sweep time = 70.0 s, power = 0.4743 mW, modulation frequency = 100.00 kHz, modulation amplitude = 10.00 G. Simulation of the data was carried out using Xepr software.

Scheme S6. Photo EPR radical capture experiment with [(4-Pyrrolidinyl-^{iPr}pybox)Ni(*p*-MeO-C_6H_4)]BAr^F_4 **33**.

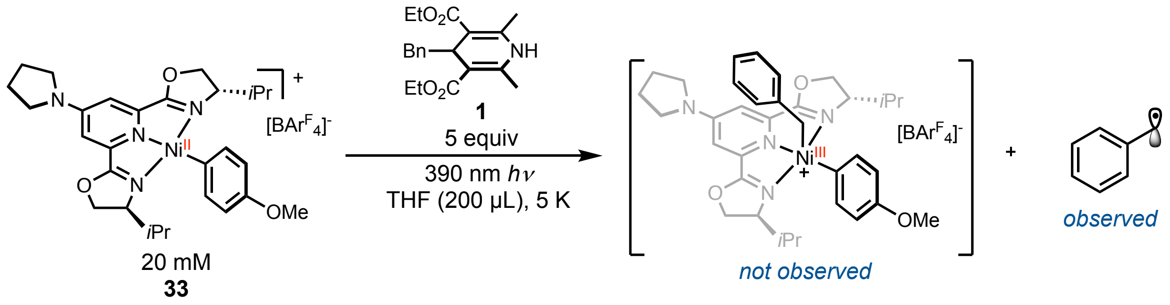

7. Synthesis and Characterization Data

Synthesis of Ligands and Substrates

(S,S)-2,6-Bis(4-isopropyl-2-oxazolin-2-yl)pyridine (^{iPr}pybox **L1**) was purchased commercially. (4S,4'S)-2,2'-(4-chloropyridine-2,6-diyl)bis(4-isopropyl-4,5-dihydrooxazole) (4-Cl-^{iPr}pybox **L2**), [7] (4S,4'S)-2,2'-(4-dimethylamino-2,6-diyl)bis(4-isopropyl-4,5-dihydrooxazole) (4-NMe_2-^{iPr}pybox **L4**),[7] (4S,4'S)-2,2'-(4-methoxy-2,6-diyl)bis(4-isopropyl-4,5-dihydrooxazole) (4-OMe-^{iPr}pybox **L5**),[7] and (4S,4'S)-2,2'-(4-trifluoromethyl-2,6-diyl)bis(4-isopropyl-4,5-dihydrooxazole) (4-CF_3-^{iPr}pybox **L7**)[8], (S,S)-2,6-Bis(4-methyl-2-oxazolin-2-yl)pyridine (^{Me}pybox **L8**)[8] and 2,6-Bis-((S)-4-isopropyl-4,5-dihydro-oxazol-2-yl)-isonicotinic acid methyl ester (4-CO_2Me-^{iPr}pybox **L9**)[9] were synthesized according to reported procedures, and characterization matched literature reports.

Diethyl 4-benzyl-2,6-dimethyl-1,4-dihydropyridine-3,5-dicarboxylate **1**, [10] Diethyl 4-(3-((N-allyl-4-methylphenyl)sulfonamido)propyl)-2,6-dimethyl-1,4-dihydropyridine-3,5-dicarboxylate **11**,[1] Diethyl 4-(3-((*N*-allyl-4-methylphenyl)sulfonamido)butan-2-yl)-2,6-dimethyl-1,4-dihydropyridine-3,5-dicarboxylate **20**[1] were synthesized following literature reported procedures.

Scheme S7. (4S,4'S)-2,2'-(4-(pyrrolidin-1-yl)pyridine-2,6-diyl)bis(4-isopropyl-4,5-dihydrooxazole) (4-pyrrolidinyl-^{iPr}pybox **L3**).

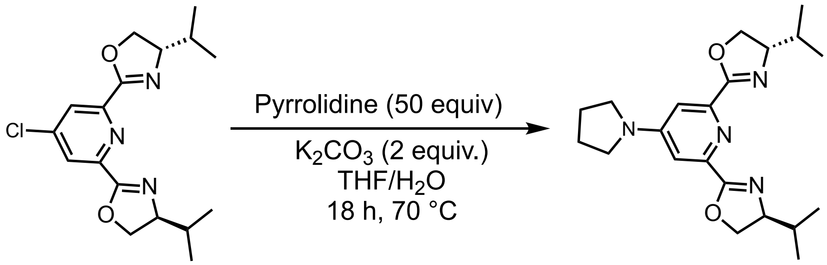

L2 **L3** 60%

(4S,4'S)-2,2'-(4-(pyrrolidin-1-yl)pyridine-2,6-diyl)bis(4-isopropyl-4,5-dihydrooxazole) (4-pyrrolidinyl-^{iPr}pybox **L3**). A flame-dried 50 mL flask was charged with a magnetic stir bar, **L2** (672 mg, 2.0 mmol), pyrrolidine (3.3 mL, 40.0 mmol), K_2CO_3 (553 mg, 4.0 mmol), THF (10 mL) and water (2 mL). The reaction mixture was then refluxed for 18 hours. The reaction mixture was cooled to room temperature and filtered through celite, washed with water (3 x 20 mL), and concentrated *in vacuo*. The residue was purified by flash column chromatography (95:5 ethyl acetate/triethylamine) to afford **L3** as a light-yellow solid (448 mg, 60%).

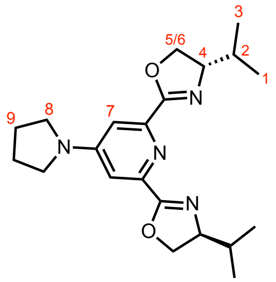

^{1}H NMR (400 MHz, chloroform-*d*) δ 7.26 (s, 2H, H_7), 4.48 (dd, J = 9.8, 8.3 Hz, 2H, $H_{5/6}$), 4.18 (t, J = 8.4 Hz, 2H, $H_{5/6}$), 4.10 (ddd, J = 9.8, 8.4, 6.5 Hz, 2H, H_4), 3.41 (m, 4H, H_8), 2.04 (m, 4H, H_9), 1.86 (m, 2H, H_2), 1.04 (d, J = 6.8 Hz, 6H, H_1/H_3), 0.92 (d, J = 6.8 Hz, 6H, H_1/H_3).

^{13}C NMR (101 MHz, chloroform-*d*) δ 163.41, 152.58, 146.88, 108.52, 72.86, 70.78, 47.60, 32.99, 25.47, 19.33, 18.38.

HRMS (ESI-TOF) m/z: $[M + H]^+$ calcd for $C_{21}H_{31}N_4O_2$ 371.2447, found 371.2417.

Scheme S8. (4S,4'S)-2,2'-(4-methyl-2,6-diyl)bis(4-isopropyl-4,5-dihydrooxazole) (4-Me-^{iPr}pybox **L6**)

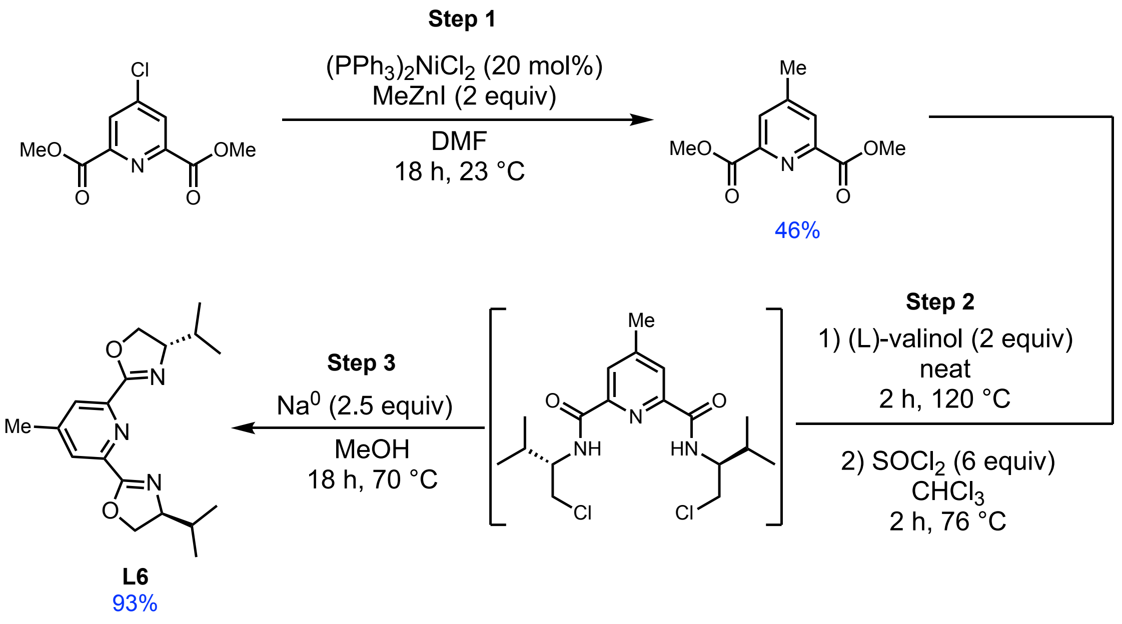

(4S,4'S)-2,2'-(4-methyl-2,6-diyl)bis(4-isopropyl-4,5-dihydrooxazole) (4-Me-^{iPr}pybox **L6**)**.**

Step 1: MeZnI was first prepared by charging a flame-dried two-necked 50 mL flask with a magnetic stir bar, zinc dust (981 mg, 15.0 mmol), fitted with a reflux condenser, and evacuated with nitrogen. Under positive nitrogen flow, a pebble of iodine and anhydrous DMF (10 mL) was added. After 15 minutes, MeI (0.65 mL, 10.0 mmol) was injected, and the reaction mixture was heated to 80 °C for 4 hours. The solution of MeZnI was cooled to room temperature and titrated with iodine prior to use. A flame-dried Schlenk flask was charged with a magnetic stir bar, dimethyl 4-chloropyridine-2,6-dicarboxylate (918 mg, 4.0 mmol) and $(PPh_3)_2NiCl_2$ (523 mg, 20 mol%), evacuated with nitrogen, and placed in an ice bath. The freshly prepared solution of MeZnI was injected and stirred overnight at room temperature. The reaction mixture was quenched with a saturated solution of NH_4Cl and filtered through a pad of celite. The mixture was extracted with DCM (3 x 20 mL), the combined organic layers were washed with water (5 x 10 mL) and concentrated *in vacuo*. The residue was purified by flash column chromatography (5-10% methanol/DCM) to afford dimethyl 4-methylpyridine-2,6-dicarboxylate as a white solid (388 mg, 46%).

Step 2: A flame-dried 50 mL flask was charged with a magnetic stir bar, dimethyl 4-methylpyridine-2,6-dicarboxylate (388 mg, 1.9 mmol), (L)-valinol (392 mg, 3.8 mmol), and fitted with a reflux condenser. The reaction mixture was heated to 120 °C for 2 hours. After cooling to room temperature, chloroform (30 mL) and SOCl2 (0.9 mL, 12.0 mmol) was added, and the reaction mixture was heated to reflux for 2 hours. The reaction mixture was cooled to room temperature and concentrated, producing a brown residue which was used immediately without further purification.

Step 3: A flame-dried 50 mL flask was charged with a magnetic stir bar, MeOH (20 mL), and sodium metal (109 mg, 4.8 mmol). The crude product of step 2 was dissolved in MeOH (10 mL) and added. The reaction flask was fitted with a reflux condenser and heated to reflux, overnight. The reaction mixture was cooled to room temperature and concentrated *in vacuo*. The residue was dissolved in DCM (50 mL), washed with water (3 x 20 mL), and concentrated *in vacuo* to afford **L6** as a tan powder (442 mg, 93% over steps 2 and 3).

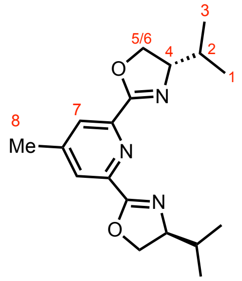

^{1}H NMR (400 MHz, chloroform-*d*) δ 8.06 (s, 2H, H_7), 4.52 (dd, J = 9.7, 8.3 Hz, 2H, $H_{5/6}$), 4.22 (t, J = 8.4 Hz, 2H, $H_{5/6}$), 4.14 (ddd, J = 9.8, 8.4, 6.4 Hz, 2H, H_4), 2.45 (s, 3H, H_8), 1.88 (qqd, J = 6.7, 6.7, 6.7 Hz, 2H, H_2), 1.05 (d, J = 6.7 Hz, 6H, H_1/H_3), 0.94 (d, J = 6.7 Hz, 6H, H_1/H_3).

^{13}C NMR (101 MHz, chloroform-*d*) δ 162.61, 149.02, 146.80, 126.68, 72.97, 71.08, 32.98, 21.04, 19.21, 18.44.

HRMS (ESI-TOF) m/z: $[M + H]^+$ calcd for $C_{18}H_{26}N_3O_2$ 316.2025, found 316.2053.

Synthesis of Diarylzinc Reagents

Diphenyl zinc (Ph_2Zn) was obtained commercially. 1-bromo-4-methylbenzene and 1-iodo-4-

methoxybenzene were dried under vacuum prior to use. *p*-bromotrifluoromethylbenzene was dried over CaH and subject to three cycles of freeze-pump-thaw prior to use. Diarylzinc reagents were synthesized according to modified literature procedures and used without further purification.

Scheme S9. Di-(4-methoxyphenyl)zinc.

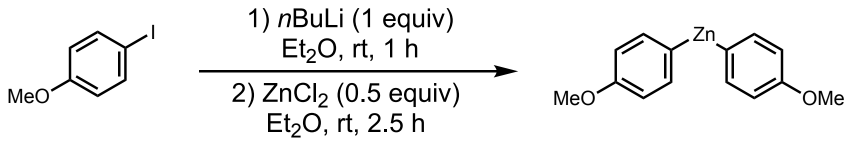

Di-(4-methoxyphenyl)zinc was synthesized based on a modified reported procedure.[11] In a nitrogen-filled glovebox, a 20 mL scintillation vial was charged with 1-iodo-4-methoxybenzene (50 mg, 1.0 equiv, 0.21 mmol), Et_2O (1 mL), and a magnetic stir bar. A solution of n-Butyllithium in hexane (0.17 mL, 1.28 molar, 1.0 equiv) was then added dropwise at room temperature and allowed to stir for 1 hour. A solution of zinc(II) chloride (15 mg, 0.5 equiv, 0.11 mmol) in Et_2O (1 mL) was then added dropwise at room temperature to the reaction mixture, after which a white precipitate formed. The reaction mixture was then stirred for 2.5 hours, after which it was filtered through celite and concentrated *in vacuo*. The solid was then washed (2 x 1 mL) and triturated with pentane resulting in a white solid. The resulting solid was used without further purification.

Scheme S10. Di-*p*-tolylzinc**.**

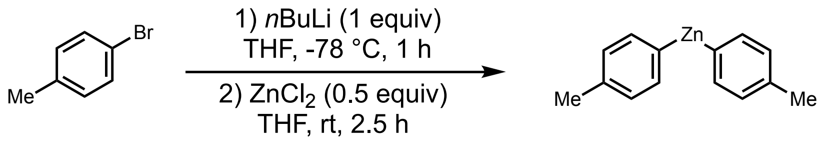

Di-*p*-tolylzinc was synthesized based on a modified reported procedure.[12] In a nitrogen-filled glovebox, a 20 mL scintillation vial was charged with 1-bromo-4-methylbenzene (0.21 g, 0.15 mL, 1 equiv, 1.2 mmol), THF (5 mL), and a magnetic stir bar. A solution of n-Butyllithium in hexanes (78 mg, 0.49 mL, 2.5 molar, 1.0 equiv, 1.2 mmol) was then added dropwise at -78 °C and allowed to sit with intermittent shaking for 1 hour. A solution of zinc(II) chloride (83 mg, 0.5 equiv, 0.61 mmol) in THF (2 mL) was then added in one portion to the reaction mixture, after which it was warmed to room temperature and stirred for 1 hour. The

reaction mixture was then concentrated *in vacuo*, washed with Et_2O, and the washings filtered through celite and concentrated *in vacuo*. The resulting residue was washed with pentane (3 x 1 mL) and triturated to yield a clear residue. The compound was used without further purification. ^1H NMR of this material showed a 1:1 adduct of (*p*-tol)$_2$Zn with Et_2O.

Scheme S11. Di-(4-trifluoromethylphenyl)zinc

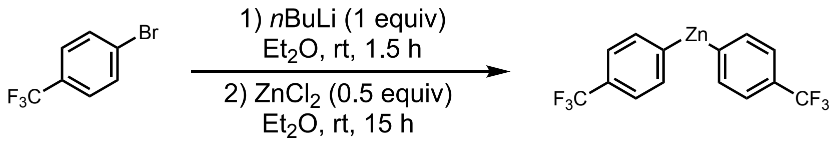

Di-(4-trifluoromethylphenyl)zinc was synthesized based on a modified reported procedure.[13] In a nitrogen-filled glovebox, a 20 mL scintillation vial was charged with *p*-bromotrifluoromethylbenzene (1.0 mL, 1 equiv, 7.2 mmol) and Et_2O (10 mL) and a magnetic sstir bar. A solution of n-Butyllithium in hexanes (5.6 mL, 1.28 molar, 1 equiv) was then added dropwise at -78 °C and allowed to sit with intermittent shaking for 1.5 hours. A solution of zinc(II) chloride (0.49 g, 0.5 equiv, 3.6) in Et_2O (5 mL) was then added in one portion to the reaction mixture, after which it was warmed to room temperature and stirred for 15 hours, resulting in a precipitate forming. The reaction mixture was then filtered through celite and concentrated *in vacuo*. The resulting residue was washed with pentane (3 x 2 mL) and triturated to yield a light orange residue. The compound was used without further purification.

Synthesis of Metal Complexes

4-bromobenzonitrile and 4-bromoacetophenone were dried under vacuum (10 mTorr) prior to use. 1-bromo-2-methyl-4-(trifluoromethyl)benzene was subject to three cycles of freeze-pump-thaw and stored over activated 4 Å molecular sieves prior to use. (^{tBu}bpy)Ni(2-methyl-4-(trifluoromethyl)phenyl)Br **S1**[14] was synthesized following literature reports. 4-substituted (^{iPr}pybox)$NiBr_2$ complexes were synthesized following a modified literature procedure.[15]

Scheme S12. (^{iPr}pybox)$NiBr_2$ **S2**

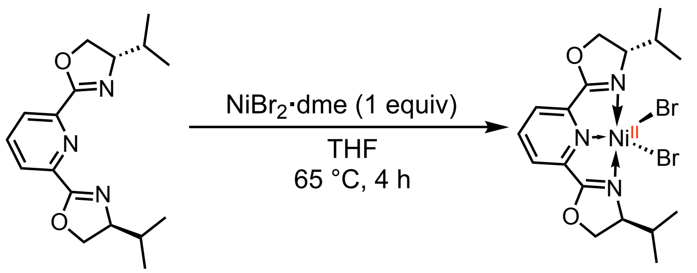

S2
Yellow
87%

(^{iPr}pybox)NiBr$_2$ **S2**. In a nitrogen-filled glovebox, ^{iPr}pybox (120 mg, 1 equiv, 398 µmol), NiBr$_2$(DME) (123 mg, 1 equiv), THF (12 mL), and a magnetic stir bar were added to a 25 mL Schlenk flask. The flask was sealed and heated to 65 °C for 4 hours. After cooling to room temperature, the flask was brought back into a nitrogen-filled glovebox and the solution was then filtered through a pad of celite and concentrated *in vacuo*. The resulting yellow solid was washed with pentane (3 x 5 mL) to yield the target product.

^{1}H NMR (400 MHz, chloroform-*d*) δ 68.21 (s, 2H), 40.55 (br, 2H), 18.26 (s, 2H), 17.43 (s, 1H), 13.82 (s, 2H), 10.33 (br, 2H), 5.29 (br, 6H), 4.84 (br, 6H). *This compound is paramagnetic and shows no ^{13}C NMR spectrum.*

Scheme S13. (4-Pyrrolidinyl-^{iPr}pybox)NiBr$_2$ **S3**

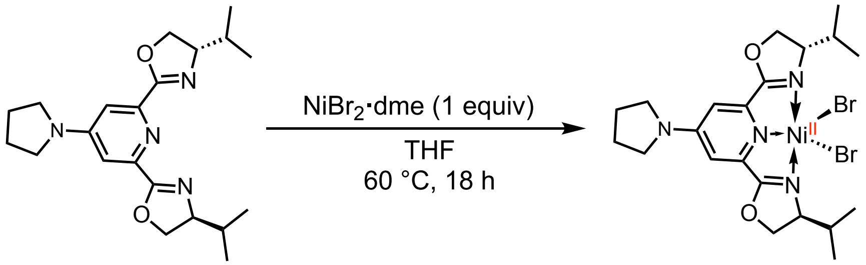

S3
Dark Red
83%

(4-pyrrolidinyl-^{iPr}pybox)NiBr$_2$ **S3**. In a nitrogen-filled glovebox, 4-pyrrolidinyl-^{iPr}pybox (86 mg, 1 equiv, 233 µmol), NiBr$_2$(DME) (72 mg, 1 equiv), THF (3 mL), and a magnetic stir bar were added to a Schlenk flask. The flask was sealed and heated to 60 °C for 18 hours. After cooling to room temperature, the flask was brought back into a nitrogen-filled glovebox and the solution was then filtered through a pad of celite

and concentrated *in vacuo*. The resulting material was washed with excess pentane to yield the target product as a red powder (137 mg, 83 % yield).

^{1}H NMR (400 MHz, benzene-*d*) δ 64.55 (s, 2H), 42.64 (s, 1H), 18.59 (s, 2H), 13.10 (s, 2H), 11.96 (s, 1 H), 6.02 (s, 6H), 5.32 (s, 6H), 0.53 (s, 4H), -6.22 (s, 2H), -6.48 (s, 2H). *This compound is paramagnetic.*

Scheme S14. (4-NMe_2-^{iPr}pybox)$NiBr_2$ **S4**

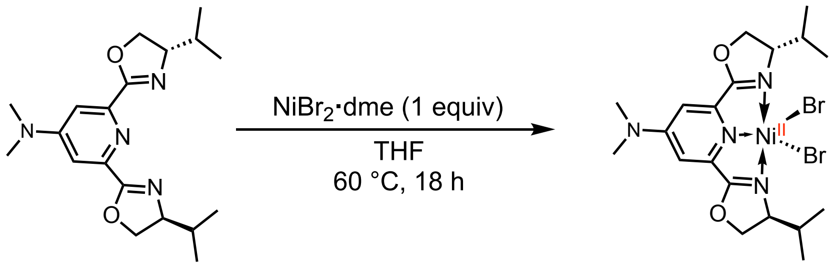

S4
Dark Yellow
88%

(4-NMe_2-^{iPr}pybox)$NiBr_2$ **S4**. In a nitrogen-filled glovebox, 4-NMe_2-^{iPr}pybox (103 mg, 1 equiv, 300 µmol), $NiBr_2$(DME) (93 mg, 1 equiv), THF (4 mL), and a magnetic stir bar were added to a Schlenk flask. The flask was sealed and heated to 60 °C for 18 hours. After cooling to room temperature, the flask was brought back into a nitrogen-filled glovebox and the solution was then filtered through a pad of celite and concentrated *in vacuo*. The resulting powder was washed with excess pentane to yield the target product as a yellow powder (149 mg, 88 % yield).

^{1}H NMR (400 MHz, benzene-*d*) δ 63.14 (s, 2H), 42.11 (s, 1H), 18.27 (s, 2H), 12.92 (s, 2H), 11.61 (s, 1H), 6.04 (s, 6H), 5.22 (s, 6H), -4.08 (s, 6H). *This compound is paramagnetic.*

Scheme S15. (4-OMe-^{iPr}pybox)$NiBr_2$ **S5**

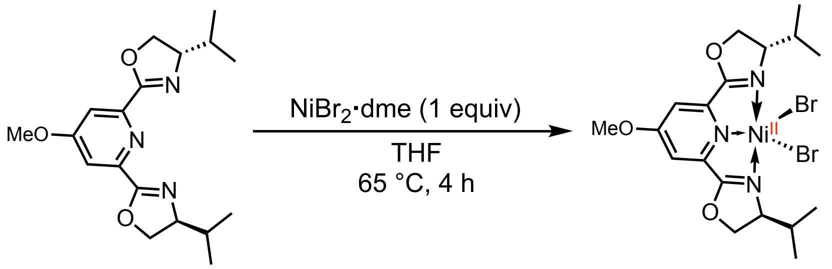

S5
Tan
Quantitative

(4-OMe-^{iPr}pybox)$NiBr_2$ **S5.** In a nitrogen-filled glovebox, 4-OMe-^{iPr}pybox (120 mg, 1 equiv, 398 µmol), $NiBr_2$(DME) (123 mg, 1 equiv), THF (12 mL), and a magnetic stir bar were added to a 25 mL Schlenk flask. The flask was sealed and heated to 65 °C for 4 hours. After cooling to room temperature, the flask was brought back into a nitrogen-filled glovebox and the solution was then filtered through a pad of celite and concentrated *in vacuo*. The resulting tan solid was washed with excess pentane to yield the target product (51 mg, quantitative).

^{1}H NMR (400 MHz, chloroform-*d*) δ 65.07 (s, 2H), 42.40 (br, 2H), 19.34 (s, 2H), 14.56 (s, 2H), 11.74 (br, 2H), 5.74 (s, 6H), 5.41 (s, 6H), 2.41 (s, 3H). *This compound is paramagnetic and shows no ^{13}C NMR spectrum.*

^{1}H NMR (400 MHz, benzene-*d*) δ 61.98 (s, 2H), 40.13 (s, 2H), 16.98 (s, 2H), 12.20 (s, 2H), 10.16 (s, 2H), 5.44 (s, 6H), 4.63 (s, 6H), 0.77 (s, 3H).

Scheme S16. (4-Me-^{iPr}pybox)$NiBr_2$ **S6**

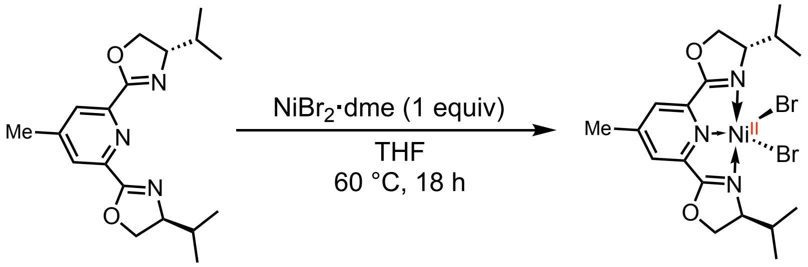

S6
Off White
96%

(4-Me-^{iPr}pybox)$NiBr_2$ **S6.** In a nitrogen-filled glovebox, 4-Me-^{iPr}pybox (63 mg, 200 µmol), $NiBr_2$(DME)

(62 mg, 1 equiv), THF (3 mL), and a magnetic stir bar were added to an oven-dried Schlenk flask. The flask was sealed and heated to 60 °C for 18 hours. The reaction mixture was then returned to a nitrogen-filled glovebox, filtered through a pad of celite and concentrated *in vacuo*. The resulting solid was washed and triturated with excess pentane to yield the target product as an off-white solid (102 mg, 96 % yield).

^{1}H NMR (400 MHz, benzene-*d*) δ 63.58 (s, 2H), 38.97 (s, 2H), 16.50 (s, 2H), 11.69 (s, 2H), 9.33 (s, 2H), 5.13 (s, 6H), 4.29 (s, 6H), -15.59 (s, 3H). *This compound is paramagnetic.*

Scheme S17. (4-CF_3-$^{i\text{Pr}}$pybox)$NiBr_2$ **S7**

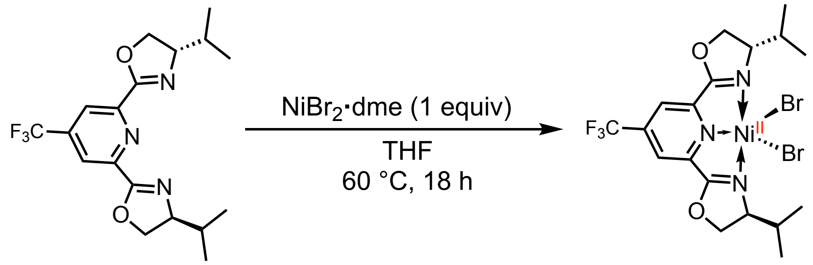

S7
Off White
95%

(4-CF_3-$^{i\text{Pr}}$pybox)$NiBr_2$ **S7**. In a nitrogen-filled glovebox, 4-CF_3-$^{i\text{Pr}}$pybox (200 mg, 1 equiv, 541 μmol), $NiBr_2$(DME) (167 mg, 1 equiv), THF (10 mL), and a magnetic stir bar were added to a Schlenk flask. The flask was sealed and heated to 60 °C for 18 hours. After cooling to room temperature, the flask was brought back into a nitrogen-filled glovebox and the solution was then filtered through a pad of celite and concentrated *in vacuo*. The resulting powder was washed and triturated with excess pentane to yield the target product as an off-white powder (304 mg, 95 % yield).

^{1}H NMR (400 MHz, chloroform-*d*) δ 63.56 (s, 2H), 38.57 (s, 2H), 17.01 (s, 2H), 12.61 (s, 2H), 8.59 (s, 2H), 4.74 (s, 6H), 4.14 (s, 6H). *This compound is paramagnetic and shows no ^{13}C NMR spectrum.*

^{1}H NMR (400 MHz, benzene-*d*) δ 60.50 (s, 2H), 38.03 (s, 2H), 15.37 (s, 2H), 11.23 (s, 2H), 8.32 (s, 2H), 4.83 (s, 6H), 3.82 (s, 6H).

^{19}F NMR (471 MHz, chloroform-*d*) δ -77.55.

Scheme S18. (^{Me}pybox)$NiBr_2$ **S8**

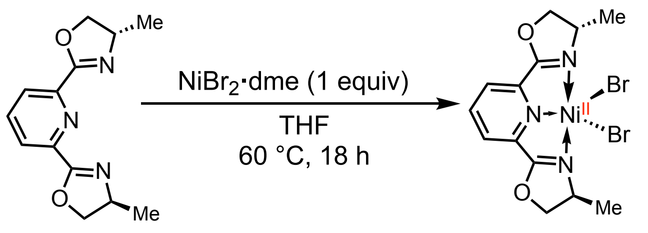

S8
Tan
92%

(^{Me}pybox)$NiBr_2$ **S8**. In a nitrogen-filled glovebox, ^{Me}pybox (250 mg, 1 equiv, 1 mmol), $NiBr_2$(DME) (315 mg, 1 equiv), THF (10 mL), and a magnetic stir bar were added to a Schlenk flask. The flask was sealed and heated to 60 °C for 18 hours. After cooling to room temperature, the flask was brought back into a nitrogen-filled glovebox and the solution was then filtered through a pad of celite and concentrated *in vacuo*. The resulting powder was washed and triturated with excess pentane to yield the target product as a tan powder (433 mg, 92 % yield).

^{1}H NMR (400 MHz, benzene-*d*) δ 65.68 (s, 2H), 40.26 (s, 2H), 15.28 (s, 2H), 14.56 (s, 1H), 14.12 (s, 2H), 6.34 (s, 6H). *This compound is paramagnetic.*

Scheme S19. [(^{iPr}pybox)Ni(Ph)]BAr^F_4 **6**

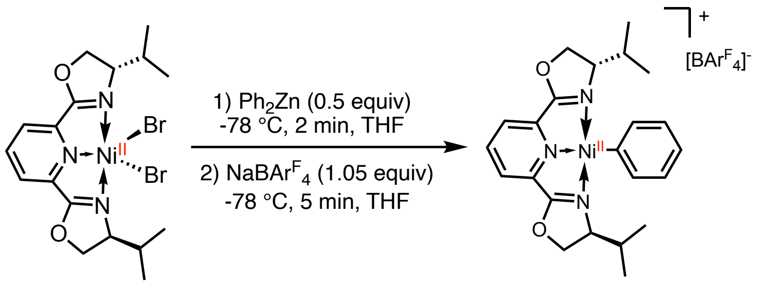

6
Red
48%

[(^{iPr}pybox)Ni(Ph)]BAr^F_4 **6** was synthesized following a modified literature procedure.[15] In a nitrogen filled glovebox, (^{iPr}pybox)$NiBr_2$ (27 mg, 52 μmol, 1.0 equiv) was dissolved in THF (2 mL) and cooled to -78 °C

in a cold well. Separately, $NaBAr^F_4$ (48 mg, 55 µmol, 1.05 equiv) and Ph_2Zn (5.7 mg, 26 µmol, 0.5 equiv) were each diluted in THF (1 ml) and cooled to -78 °C. The solution of Ph_2Zn in THF was added dropwise to the solution of (^{iPr}pybox)$NiBr_2$ while agitating the solution, upon which a rapid color change to dark red was observed. The solution was then left to sit at -78 °C for 2 minutes with intermittent shaking, then $NaBAr^F_4$ was added in one portion. The solution was then left to sit for 5 minutes, after which the reaction mixture was passed through a cold, THF-rinsed plug of alumina and concentrated *in vacuo* to yield a dark residue. The residue was then washed with excess pentane and dried *in vacuo*. The residue was extracted with toluene and filtered through a pad of alumina, then concentrated *in vacuo*. The resulting solid was triturated with pentane to yield the target complex as a red powder (32 mg, 48% yield).

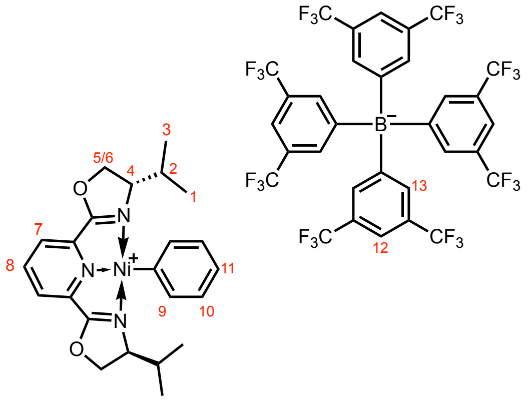

^{1}H NMR (400 MHz, benzene-*d*) δ 8.35 (s, 8H, H_{13}), 7.60 (s, 4H, H_{12}), 7.01 (t, J = 7.3 Hz, 2H, H_{10}), 6.67 (t, J = 8.0 Hz, 1H, H_8), 6.30 (d, J = 8.0 Hz, 2H, H_7), 3.76 (dd, J = 9.6, 6.5 Hz, 2H, $H_{5/6}$), 3.60 (t, J = 9.9 Hz, 2H, $H_{5/6}$), 3.07 (ddd, J = 10.1, 6.6, 3.4 Hz, 2H, H_4), 0.82 – 0.68 (m, 2H, H_2), 0.32 (d, J = 6.8 Hz, 6H, H_1/H_3), 0.01 (d, J = 7.0 Hz, 6H, H_1/H_3). *Peaks for H_9 and H_{11} overlap with the residual solvent signal for benzene-d.*

Scheme S20. [(4-Pyrrolidinyl-^{iPr}pybox)Ni(Ph)]BAr^F_4 **21**

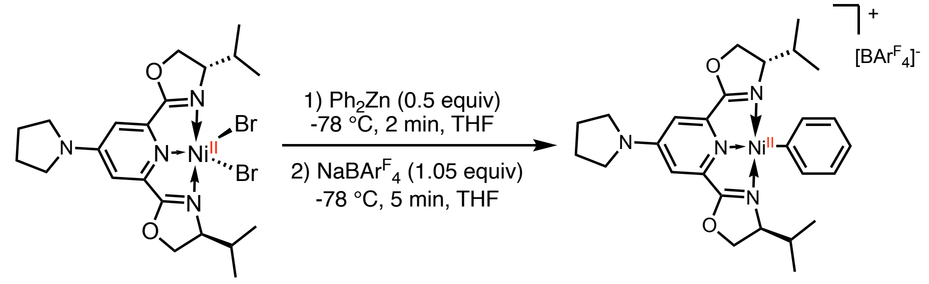

21
Yellow
79%

[(4-Pyrrolidinyl-^{iPr}pybox)Ni(Ph)]BAr$^F{}_4$ **21.** In a nitrogen filled glovebox, (4-Pyrrolidinyl-^{iPr}pybox)$NiBr_2$ **S3** (29.5 mg, 50 µmol, 1 equiv) was dissolved in THF (4 mL) and cooled to -78 °C in a cold well. Separately, NaBAr$^F{}_4$ (46.5 mg, 53 µmol, 1.05 equiv) and Ph_2Zn (5.5 mg, 25 µmol, 0.5 equiv) were diluted in THF (1 ml and 2 mL, respectively) and cooled to -78 °C. The solution of Ph_2Zn in THF was added dropwise to the solution of (4-Pyrrolidinyl-^{iPr}pybox)$NiBr_2$ **S3** while agitating the solution. The solution was then left to sit at -78 °C for 2 minutes with intermittent shaking, then NaBAr$^F{}_4$ was added in one portion. The solution was then left to sit for 5 minutes, after which the reaction mixture was passed through a cold, THF-rinsed plug of celite and concentrated *in vacuo*. The residue was then extracted with toluene and filtered through a pad of celite, then concentrated *in vacuo*. The resulting solid was triturated and washed with excess pentane to yield the target complex as a bright yellow powder (54 mg, 79% yield).

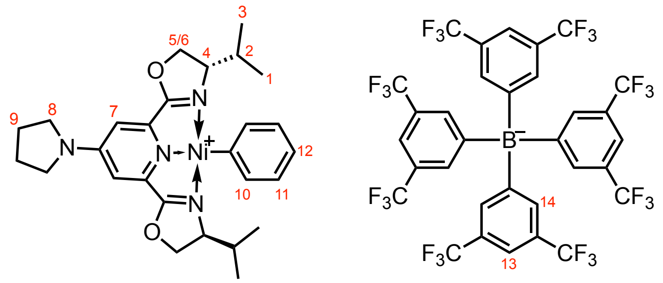

^{1}H NMR (500 MHz, CD_2Cl_2) δ 7.72 (br, 8H, H_{14}), 7.56 (br, 4H, H_{13}), 7.38 (d, J = 6.5 Hz, 2H, H_{10}), 6.99 (t, J = 7.4 Hz, 2H, H_{11}), 6.91 (t, J = 7.3 Hz, 1H, H_{12}), 6.75 (s, 2H, H_7), 4.75 (t, J = 9.8 Hz, 2H, $H_{5/6}$), 4.68 (dd, J = 9.4, 6.4 Hz, 2H, $H_{5/6}$), 3.81 (ddd, J = 9.9, 6.3, 3.5 Hz, 2H, H_4), 3.40 (m, 4H, H_8), 2.10 (m, 4H, H_9), 1.04 (m, 2H, H_2), 0.72 (d, J = 6.9 Hz, 6H, H_1/H_3), 0.49 (d, J = 7.1 Hz, 6H, H_1/H_3).

^{13}C NMR (126 MHz, CD_2Cl_2) δ 169.21, 162.15 (q, *J* = 50.1 Hz), 155.18, 141.58, 141.26, 136.77, 135.19,

129.27 (q, J = 32.0 Hz), 126.90, 124.99 (q, J = 272.3 Hz), 124.61, 117.87, 106.26, 74.48, 65.77, 49.39, 28.68, 25.51, 18.37, 13.77.

^{19}F NMR (471 MHz, CD_2Cl_2) δ -62.83.

UV-Vis (λ(ε), THF, 23 °C): 208 nm (95529 $M^{-1}cm^{-1}$), 329 nm (10524 $M^{-1}cm^{-1}$).

HRMS (ESI-TOF) m/z: $[M–BAr^F{}_4]^+$ calcd for $C_{27}H_{35}N_4NiO_2$ 505.2113, found 505.2184.

Scheme S21. [(4-NMe$_2$-^{iPr}pybox)Ni(Ph)]BAr$^F{}_4$ **22**

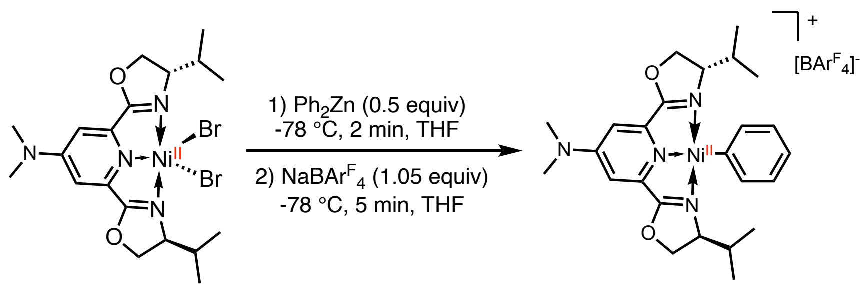

22
Yellow
63%

[(4-NMe$_2$-^{iPr}pybox)Ni(Ph)]BAr$^F{}_4$ **22.** In a nitrogen filled glovebox, (4-NMe$_2$-^{iPr}pybox)NiBr$_2$ **S4** (47.0 mg, 84 µmol, 1 equiv) was dissolved in THF (6 mL) and cooled to -78 °C in a cold well. Separately, NaBAr$^F{}_4$ (77.7 mg, 88 µmol, 1.05 equiv) and Ph_2Zn (9.2 mg, 42 µmol, 0.5 equiv) were diluted in THF (2 ml and 3 mL, respectively) and cooled to -78 °C. The solution of Ph_2Zn in THF was added dropwise to the solution of (4-NMe$_2$-^{iPr}pybox)NiBr$_2$ **S4** while agitating the solution. The solution was then left to sit at -78 °C for 2 minutes with intermittent shaking, then NaBAr$^F{}_4$ was added in one portion. The solution was then left to sit for 5 minutes, after which the reaction mixture was passed through a cold, THF-rinsed plug of celite and concentrated *in vacuo*. The residue was then extracted with toluene and filtered through a pad of celite, then concentrated *in vacuo*. The resulting solid was triturated and washed with excess pentane to yield the target complex as a bright yellow solid (70.1 mg, 63% yield).

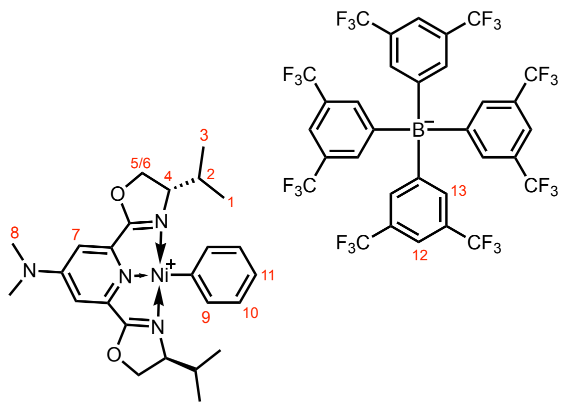

^{1}H NMR (500 MHz, CD_2Cl_2) δ 7.72 (br, H_{13}), 7.56 (br, H_{12}), 7.37 (d, J = 7.3 Hz, 2H, H_9), 6.99 (t, J = 7.3 Hz, 2H, H_{10}), 6.91 (t, J = 7.2 Hz, 1H, H_{11}), 6.86 (s, 2H, H_7), 4.75 (t, J = 9.8 Hz, 2H, $H_{5/6}$), 4.69 (dd, J = 9.5, 6.4 Hz, 2H, $H_{5/6}$), 3.81 (ddd, J = 10.0, 6.4, 3.5 Hz, 2H, H_4), 3.13 (s, 6H, H_8), 1.04 (m, 2H, H_2), 0.72 (d, J = 6.9 Hz, 6H, H_1/H_3), 0.50 (d, J = 7.2 Hz, 6H, H_1/H_3).

^{13}C NMR (126 MHz, CD_2Cl_2) δ 169.16, 162.15 (q, J = 49.6 Hz), 157.94, 141.79, 141.06, 136.73, 135.20, 129.27 (d, J = 32.8 Hz), 126.93, 124.99 (q, J = 272.7 Hz), 124.65, 117.88, 105.68, 74.54, 65.79, 40.95, 28.69, 18.35, 13.76.

^{19}F NMR (471 MHz, CD_2Cl_2) δ -62.77.

UV-Vis (λ(ε), THF, 23 °C): 210 nm (94488 $M^{-1}cm^{-1}$), 329 nm (10810 $M^{-1}cm^{-1}$).

HRMS (ESI-TOF) m/z: $[M–BAr^F{}_4]^+$ calcd for $C_{25}H_{33}N_4NiO_2$ 479.1957, found 479.1945.

Scheme S22. [(4-OMe-^{iPr}pybox)Ni(Ph)]$BAr^F{}_4$ **23**

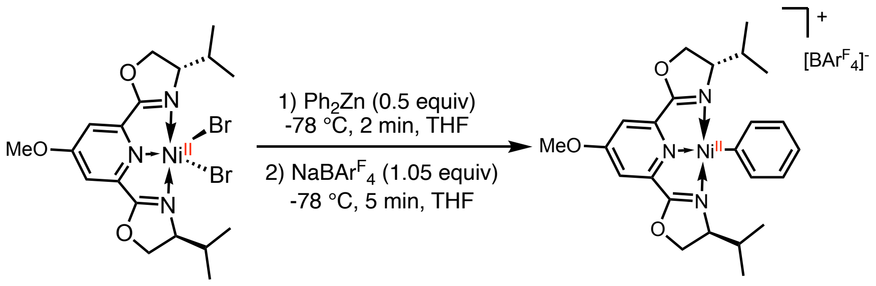

23
Dark Red
78%

[(4-OMe-^{iPr}pybox)Ni(Ph)]$BAr^F{}_4$ **23**. In a nitrogen filled glovebox, (4-OMe-^{iPr}pybox)$NiBr_2$ **S5** (27.5 mg, 50

μmol, 1 equiv) was dissolved in THF (4 mL) and cooled to -78 °C in a cold well. Separately, $NaBAr^F_4$ (46.5 mg, 53 μmol, 1.05 equiv) and Ph_2Zn (5.5 mg, 25 μmol, 0.5 equiv) were diluted in THF (1 ml and 2 mL, respectively) and cooled to -78 °C. The solution of Ph_2Zn in THF was added dropwise to the solution of (4-OMe-$^{i\text{Pr}}$pybox)$NiBr_2$ **S5** while agitating the solution. The solution was then left to sit at -78 °C for 2 minutes with intermittent shaking, then $NaBAr^F_4$ was added in one portion. The solution was then left to sit for 5 minutes, after which the reaction mixture was passed through a cold, THF-rinsed plug of celite and concentrated *in vacuo*. The residue was then extracted with toluene and filtered through a pad of celite, then concentrated *in vacuo*. The resulting solid was triturated and washed with excess pentane to yield the target complex as a dark red solid (51.9 mg, 78% yield).

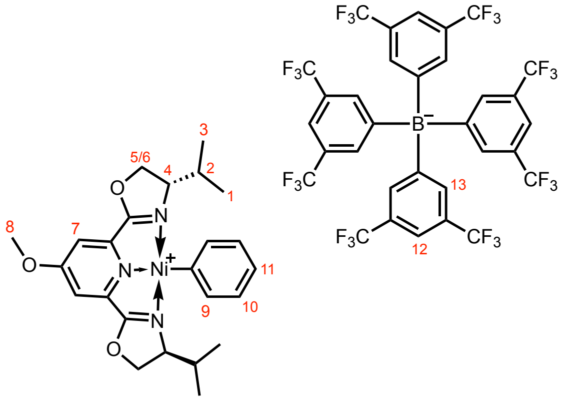

^{1}H NMR (500 MHz, CD_2Cl_2) δ 7.72 (br, H_{13}), 7.55 (br, H_{12}), 7.34 (d, 2H, H_9), 7.31 (s, 2H, H_7), 7.02 (t, J = 7.4 Hz, 2H, H_{10}), 6.94 (t, J = 7.2 Hz, 1H, H_{11}), 4.82 (t, J = 9.9 Hz, 2H, $H_{5/6}$), 4.75 (dd, J = 9.5, 6.5 Hz, 2H, $H_{5/6}$), 4.01 (s, 3H, H_8), 3.87 (ddd, J = 10.1, 6.5, 3.4 Hz, 2H, H_4), 1.05 (qqd, J = 7.0, 7.0, 3.5 Hz, 2H, H_2), 0.72 (d, J = 6.8 Hz, 6H, H_1/H_3), 0.51 (d, J = 7.1 Hz, 6H, H_1/H_3).

^{1}H NMR (400 MHz, benzene-*d*) δ 8.36 (s, 8H, H_{13}), 7.60 (s, 4H, H_{12}), 7.22 (d, J = 6.9 Hz, 2H, H_9), 7.03 (t, J = 7.2 Hz, 2H, H_{10}), 6.04 (s, 2H, H_7), 3.77 (dd, J = 9.6, 6.5 Hz, 2H, $H_{5/6}$), 3.59 (t, J = 9.9 Hz, 2H, $H_{5/6}$), 3.10 (ddd, J = 10.1, 6.4, 3.5 Hz, 2H, H_4), 2.75 (s, 3H, H_8), 0.80 (m, 2H, H_2), 0.37 (d, J = 6.9 Hz, 6H, H_1/H_3), 0.03 (d, J = 7.1 Hz, 6H, H_1/H_3).

^{13}C NMR (126 MHz, CD_2Cl_2) δ 171.56, 168.39, 162.15 (q, J = 49.9 Hz), 143.74, 136.28, 135.21, 129.27 (q, J = 30.8 Hz), 128.70, 127.29, 125.13, 124.99 (q, J = 271.7 Hz), 117.90, 111.13, 75.01, 66.04, 58.24,

28.73, 18.36, 13.79.

^{19}F NMR (471 MHz, CD_2Cl_2) δ -62.77.

UV-Vis (λ(ε), THF, 23 °C): 218 nm (82417 $M^{-1}cm^{-1}$), 403 nm (1003 $M^{-1}cm^{-1}$).

HRMS (ESI-TOF) m/z: $[M–BAr^F{}_4]^+$ calcd for $C_{24}H_{30}N_3NiO_3$ 466.1641, found 466.1477.

Scheme S23. [(4-Me-^{iPr}pybox)Ni(Ph)]$BAr^F{}_4$ **24**

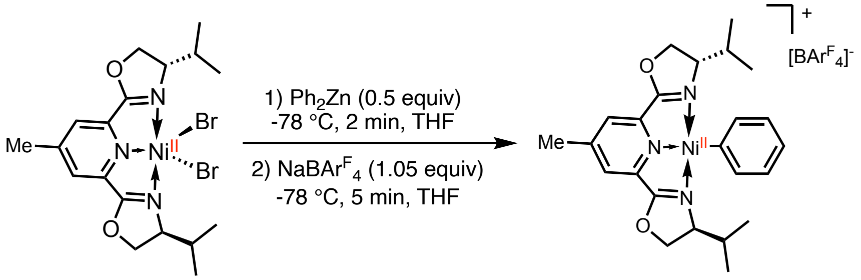

24
Bright Red
69%

[(4-Me-^{iPr}pybox)Ni(Ph)]$BAr^F{}_4$ **24**. In a nitrogen filled glovebox, (4-Me-^{iPr}pybox)$NiBr_2$ **S6** (26.7 mg, 50 µmol, 1 equiv) was dissolved in THF (4 mL) and cooled to -78 °C in a cold well. Separately, $NaBAr^F{}_4$ (46.5 mg, 53 µmol, 1.05 equiv) and Ph_2Zn (5.5 mg, 25 µmol, 0.5 equiv) were diluted in THF (1 ml and 2 mL, respectively) and cooled to -78 °C. The solution of Ph_2Zn in THF was added dropwise to the solution of (4-Me-^{iPr}pybox)$NiBr_2$ **S6** while agitating the solution. The solution was then left to sit at -78 °C for 2 minutes with intermittent shaking, then $NaBAr^F{}_4$ was added in one portion. The solution was then left to sit for 5 minutes, after which the reaction mixture was passed through a cold, THF-rinsed plug of celite and concentrated *in vacuo*. The residue was then extracted with toluene and filtered through a pad of celite, then concentrated *in vacuo*. The resulting solid was triturated and washed with excess pentane to yield the target complex as a bright red powder (45.2 mg, 69% yield).

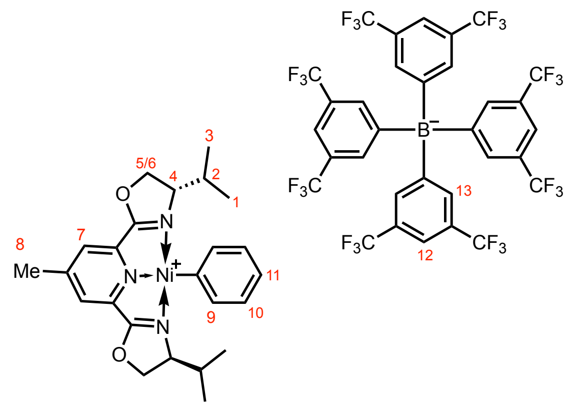

^{1}H NMR (500 MHz, CD_2Cl_2) δ 7.72 (br, H_{13}), 7.68 (s, 2H, H_7), 7.56 (br, H_{12}), 7.33 (d, J = 7.4 Hz, 2H, H_9), 7.03 (t, J = 7.4 Hz, 2H, H_{10}), 6.95 (t, J = 7.3 Hz, 1H, H_{11}), 4.84 (t, J = 9.9 Hz, 2H, $H_{5/6}$), 4.77 (dd, J = 9.5, 6.5 Hz, 2H, $H_{5/6}$), 3.88 (ddd, J = 10.2, 6.5, 3.5 Hz, 2H, H_4), 2.57 (s, 3H, H_8), 1.07 (m, 2H, H_2), 0.72 (d, J = 6.9 Hz, 6H, H_1/H_3), 0.52 (d, J = 7.1 Hz, 6H, H_1/H_3).

^{13}C NMR (126 MHz, CD_2Cl_2) δ 168.55, 162.15 (q, J = 49.8 Hz), 157.20, 142.26, 139.70, 136.13, 135.21, 129.27 (q, J = 31.7 Hz), 127.37, 125.66, 125.25, 124.99 (q, J = 272.3 Hz), 117.90, 75.06, 66.00, 28.75, 22.85, 18.38, 13.80.

^{19}F NMR (471 MHz, CD_2Cl_2) δ -62.76.

UV-Vis (λ(ε), THF, 23 °C): 209 nm (66428 $M^{-1}cm^{-1}$), 409 nm (2689 $M^{-1}cm^{-1}$).

HRMS (ESI-TOF) m/z: $[M–BAr^F{}_4]^+$ calcd for $C_{24}H_{30}N_3NiO_2$ 450.1692, found 450.1217.

Scheme S24. [(4-CF$_3$-^{iPr}pybox)Ni(Ph)]BAr$^F{}_4$ **25**

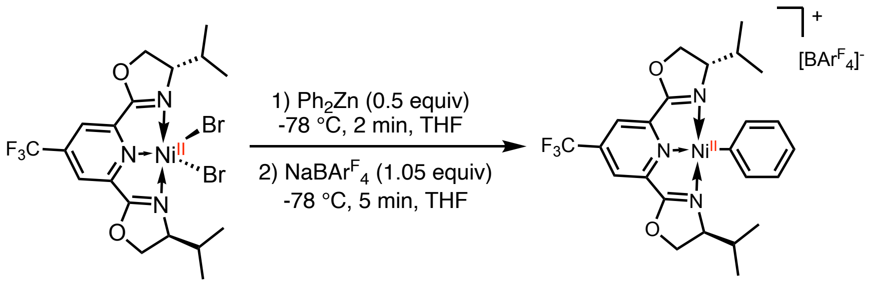

25
Yellow
41%

[(4-CF$_3$-^{iPr}pybox)Ni(Ph)]BAr$^F{}_4$ **25.** In a nitrogen filled glovebox, (4-CF$_3$-^{iPr}pybox)NiBr$_2$ **S7** (29.4 mg, 50

μmol, 1 equiv) was dissolved in THF (4 mL) and cooled to -78 °C in a cold well. Separately, $NaBAr^F_4$ (46.5 mg, 53 μmol, 1.05 equiv) and Ph_2Zn (5.5 mg, 25 μmol, 0.5 equiv) were diluted in THF (1 ml and 2 mL, respectively) and cooled to -78 °C. The solution of Ph_2Zn in THF was added dropwise to the solution of (4-CF_3-^{iPr}pybox)$NiBr_2$ **S7** while agitating the solution. The solution was then left to sit at -78 °C for 2 minutes with intermittent shaking, then $NaBAr^F_4$ was added in one portion. The solution was then left to sit for 5 minutes, after which the reaction mixture was passed through a cold, THF-rinsed plug of celite and concentrated *in vacuo*. The residue was then extracted with toluene and filtered through a pad of celite, then concentrated *in vacuo*. The resulting solid was triturated and washed with excess pentane to yield the target complex as a yellow-brown powder (28.0 mg, 41% yield).

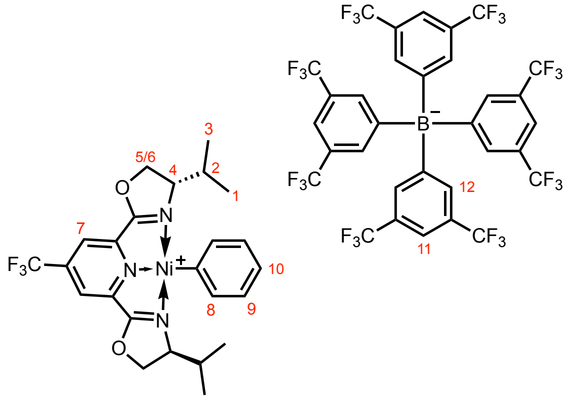

^{1}H NMR (500 MHz, CD_2Cl_2) δ 8.13 (s, 2H, H_7), 7.71 (br, H_{12}), 7.55 (br, H_{11}), 7.27 (d, J = 7.4 Hz, 2H, H_8), 7.07 (t, J = 7.3 Hz, 2H, H_9), 7.00 (t, J = 7.1 Hz, 1H, H_{10}), 4.93 (t, J = 9.9 Hz, 2H, $H_{5/6}$), 4.86 (dd, J = 9.6, 6.6 Hz, 2H, $H_{5/6}$), 3.99 (ddd, J = 10.2, 6.6, 3.5 Hz, 2H, H_4), 1.09 (m, 2H, H_2), 0.73 (d, J = 6.9 Hz, 6H, H_1/H_3), 0.54 (d, J = 7.1 Hz, 6H, H_1/H_3).

^{13}C NMR (126 MHz, THF-*d*) δ 169.45, 162.95 (q, 49.9 Hz), 144.88, 144.43 (m), 141.29, 136.69, 135.74, 130.16 (qq, J = 31.5, 2.8 Hz), 127.92, 125.96, 125.65 (q, J = 272.3 Hz), 122.92 (m), 122.63 (q, J = 3.6 Hz), 118.32 (m), 76.15, 66.73, 29.66, 18.37, 13.94. *Slight decomposition was observed due to prolonged scans.*

^{19}F NMR (471 MHz, CD_2Cl_2) δ -62.75, -64.91.

UV-Vis (λ(ε), THF, 23 °C): 209 nm (81917 $M^{-1}cm^{-1}$), 423 nm (4433 $M^{-1}cm^{-1}$).

HRMS (ESI-TOF) m/z: $[M–BAr^F_4]^+$ calcd for $C_{24}H_{27}F_3N_3NiO_2$ 504.1409, found 504.1663.

Scheme S25. [(4- CO_2Me-^{iPr}pybox)Ni(Ph)]BAr$^F{}_4$ **26**.

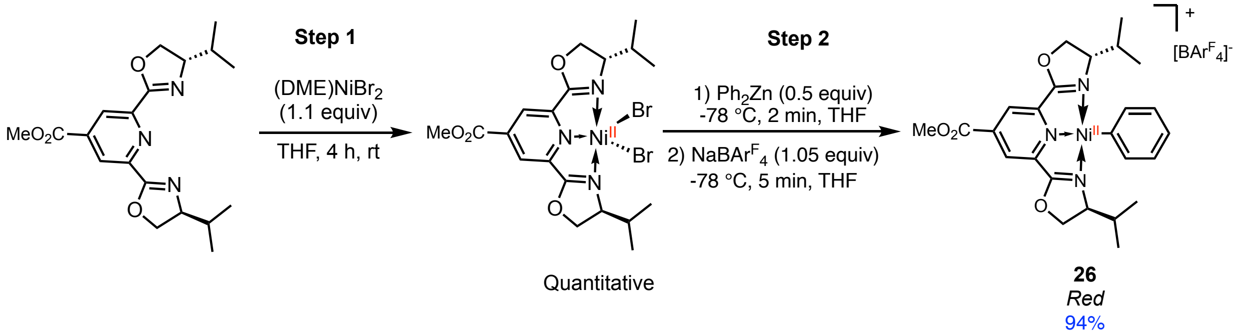

[(4- CO_2Me-^{iPr}pybox)Ni(Ph)]BAr$^F{}_4$ **26**.

Step 1: In a nitrogen filled glovebox, 4-CO_2Me-^{iPr}pybox (17.9 mg, 49.8 µmol, 1 equiv), $NiBr_2$(DME) (16.9 mg, 1.1 equiv), THF (5 mL), and a magnetic stir bar were added to a 20 mL scintillation vial and stirred at room temperature for 4 hours. The solution was then filtered through a pad of celite and concentrated *in vacuo*. The resulting green powder was washed with excess pentane and used without further purification (28.8 mg, quantitative). This is tentatively assigned as (4-CO_2Me-^{iPr}pybox)$NiBr_2$.

Step 2: In a nitrogen filled glovebox, (4-CO_2Me-^{iPr}pybox)$NiBr_2$ (28.8 mg, 49.8 µmol, 1 equiv) was dissolved in THF (5 mL) and cooled to -78 °C in a cold well. Separately, NaBAr$^F{}_4$ (46.4 mg, 52.3 µmol, 1.05 equiv) and Ph_2Zn (5.5 mg, 24.9 µmol, 0.5 equiv) were each diluted in THF (1 mL) and cooled to -78 °C. The solution of Ph_2Zn in THF was added dropwise to the solution of (4-CO_2Me-^{iPr}pybox)$NiBr_2$ while agitating the solution. The solution was then left to sit at -78 °C for 2 minutes, then NaBAr$^F{}_4$ was added in one portion. The solution was then left to sit for 5 minutes with intermittent shaking, after which the reaction mixture was warmed to room temperature concentrated *in vacuo* to approximately 0.5 mL volume and diluted with pentane (10 mL). The supernatant was decanted, and the resulting precipitate was triturated with pentane then extracted with 1:1 toluene:Et_2O and filtered through celite. The resulting solution was concentrated *in vacuo* to approximately 0.5 mL volume and diluted in pentane (10 mL), precipitating out a red residue. The residue was washed and triturated with pentane (3 x 1 mL) to yield the target complex as a red powder (63.3 mg, 94% yield). *The product is unstable in solution over days. Attempts at re-dissolving*

in Et_2O for recrystallization resulted in partial decomposition.

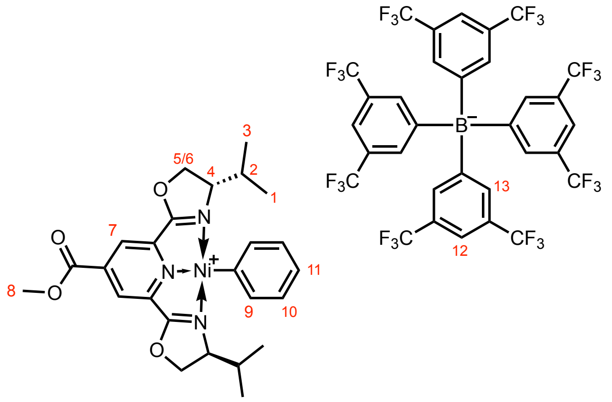

^{1}H NMR (400 MHz, THF-*d*) δ 8.53 (s, 2H, H_7), 7.78 (br, H_{13}), 7.57 (br, H_{12}), 7.38 (d, J = 7.2 Hz, 2H, H_9), 7.03 (t, J = 7.2 Hz, 2H, H_{10}), 6.94 (d, J = 7.2 Hz, 1H, H_{11}), 4.99 (m, 4H, $H_{5/6}$), 4.03 (m, 5H, H_4 and H_8), 0.95 (m, 2H, H_2), 0.74 (d, J = 6.9 Hz, 6H, H_1/ H_3), 0.54 (d, J = 7.0 Hz, 6H, H_1/H_3). *Compound decomposes over long scan times, complicating collection of ^{13}C NMR spectrum.*

^{19}F NMR (377 MHz, THF-*d*) δ -63.41.

HRMS (ESI-TOF) m/z: $[M–BAr^F{}_4]^+$ calcd for $C_{25}H_{30}N_3NiO_4$ 494.1590, found 494.1572

Scheme S26. [(^{iPr}pybox)Ni(*p*-MeO-C_6H_4)]$BAr^F{}_4$ **27**

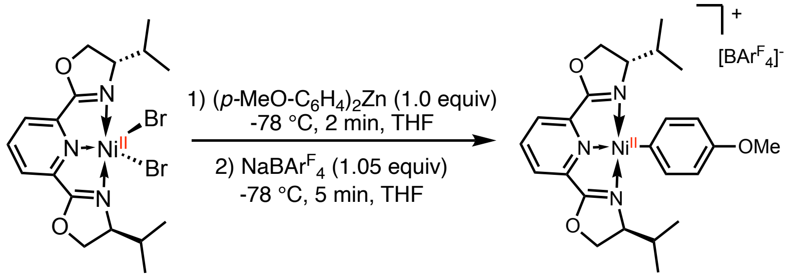

27
Red
56%

[(^{iPr}pybox)Ni(*p*-MeO-C_6H_4)]$BAr^F{}_4$ **27**. In a nitrogen filled glovebox, (^{iPr}pybox)$NiBr_2$ **S2** (45 mg, 87 μmol, 1 equiv) was dissolved in THF (4 mL) and cooled to -78 °C in a cold well. Separately, $NaBAr^F{}_4$ (81 mg, 91 μmol, 1.05 equiv) and (*p*-OMe-Ph)$_2$Zn (24 mg, 87 μmol, 1.0 equiv) were each diluted in THF (1 mL) and cooled to -78 °C. The solution of (*p*-OMe-Ph)$_2$Zn in THF was added dropwise to the solution of

(^{iPr}pybox)$NiBr_2$ **S2** while agitating the solution, upon which a rapid color change from orange to red-brown was observed. The solution was then left to sit at -78 °C for 2 minutes, then $NaBAr^F_4$ was added in one portion. The solution was then left to sit for 5 minutes, after which the reaction mixture was concentrated *in vacuo* to yield a dark residue, which was triturated with pentane. The residue was extracted with toluene and filtered through a pad of celite, then concentrated *in vacuo*. The resulting solid was washed (3 x 3 mL) and triturated with pentane to yield the target complex as a dark red powder (65 mg, 56% yield).

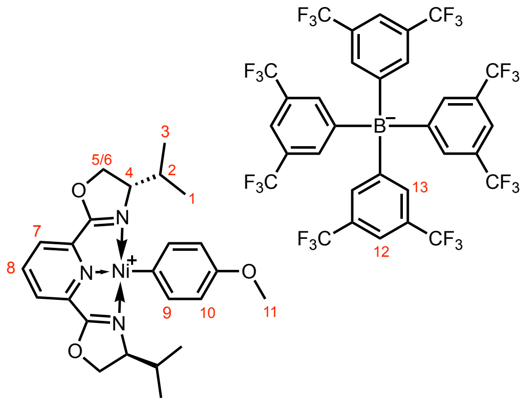

^{1}H NMR (400 MHz, benzene-*d*) δ 8.36 (s, 8H, H_{13}), 7.61 (s, 4H, H_{12}), 7.00 (d, J = 8.4 Hz, 2H, H_9), 6.84 (d, J = 8.3 Hz, 2H, H_{10}), 6.68 (t, J = 7.9 Hz, 2H, H_8), 6.32 (d, J = 7.8 Hz, 2H, H_7), 3.78 (dd, J = 9.6, 6.5 Hz, 2H, $H_{5/6}$), 3.62 (t, J = 9.9 Hz, 2H, $H_{5/6}$), 3.39 (s, 3H, H_{11}), 3.11 (ddd, J = 10.1, 6.4, 3.5 Hz, 2H, H_4), 0.84 (m, 2H, H_2), 0.35 (d, J = 6.9 Hz, 6H, H_1/H_3), 0.04 (d, J = 7.1 Hz, 6H, H_1/H_3).

^{13}C NMR (126 MHz, benzene-*d*) δ 167.56, 162.87 (m), 141.75, 141.39, 135.72, 135.43, 130.05 (q, J = 33.5 Hz), 125.18 (q, J = 272.1 Hz), 124.75, 123.78, 122.84, 118.18, 113.97, 74.15, 65.47, 54.73, 28.33, 17.94, 13.19.

^{19}F NMR (471 MHz, benzene-*d*) δ -62.09

UV-Vis (λ(ε), THF, 23 °C): 213 nm (61612 $M^{-1}cm^{-1}$), 408 nm (3842 $M^{-1}cm^{-1}$).

HRMS (ESI-TOF) m/z: $[M–BAr^F_4]^+$ calcd for $C_{24}H_{30}N_3NiO_3$ 466.1641, found 466.1649.

Scheme S 27. [(^{iPr}pybox)Ni(*p*-tol)]BAr^F_4 **28**

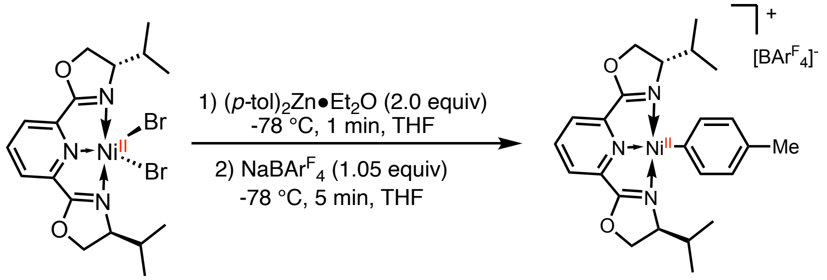

28
Red
58%

[($^{i\text{Pr}}$pybox)Ni(p-tol)]BAr^F_4 **28**. In a nitrogen filled glovebox, ($^{i\text{Pr}}$pybox)$NiBr_2$ **S2** (19 mg, 36 µmol, 1 equiv) was dissolved in THF (3 mL) and cooled to -78 °C in a cold well. Separately, $NaBAr^F_4$ (34 mg, 38 µmol, 1.05 equiv) and (p-tol)$_2$Zn•Et_2O (23 mg, 73 µmol, 2.0 equiv) were each diluted in THF (1 mL) and cooled to -78 °C. The solution of (p-tol)$_2$Zn•Et_2O in THF was added dropwise to the solution of ($^{i\text{Pr}}$pybox)$NiBr_2$ **S2** over the course of 1 minute while agitating the solution, upon which a rapid color change to dark red was observed. $NaBAr^F_4$ was added in one portion as a solution in THF and the solution was then left to sit for 5 minutes, after which the reaction mixture was filtered through a pad of celite and concentrated *in vacuo*. The resulting residue was triturated with pentane, extracted with toluene, and filtered through a pad of celite. The filtrate was concentrated *in vacuo* to yield a dark residue, which was washed (3 x 5 mL) and triturated with pentane to yield the target complex as a red powder (28 mg, 58% yield).

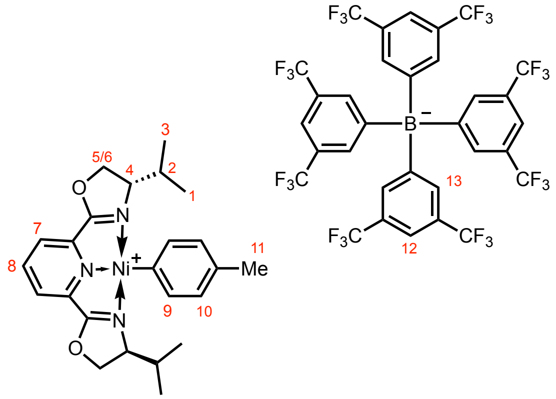

^{1}H NMR (400 MHz, benzene-*d*) δ 8.46 – 8.03 (br, 8H, H_{13}), 7.63 (br, 4H, H_{12}), 7.05 (d, J = 7.9 Hz, 2H, H_9), 6.92 (d, J = 7.5 Hz, 2H, H_{10}), 6.68 (t, J = 7.9 Hz, 1H, H_8), 6.40 (d, J = 7.9 Hz, 2H, H_7), 3.82 (dd, J = 9.6, 6.5 Hz, 2H, $H_{5/6}$), 3.65 (t, J = 9.9 Hz, 2H, $H_{5/6}$), 3.15 (m, H_4), 2.20 (s, 3H, H_{11}), 1.00 – 0.81 (m, 2H,

H_2), 0.36 (d, J = 6.9 Hz, 6H, H_1/H_3), 0.04 (d, J = 7.1 Hz, 6H, H_1/H_3).

^{13}C NMR (126 MHz, benzene-*d*) δ 167.56, 162.76 (q, J = 49.6 Hz), 141.50, 141.37, 135.47, 135.37, 134.96, 134.01, 129.98 (q, J = 33.4 Hz), 129.34, 124.11 (q, J = 272.3 Hz), 123.54, 118.10, 73.88, 65.36, 28.26, 20.73, 17.79, 13.05.

^{19}F NMR (471 MHz, benzene-*d*) δ -62.09

UV-Vis (λ(ε), THF, 23 °C): 216 nm (84602 $M^{-1}cm^{-1}$), 403 nm (4479 $M^{-1}cm^{-1}$).

HRMS (ESI-TOF) m/z: $[M–BAr^F_4]^+$ calcd for $C_{24}H_{30}N_3NiO_2$ 450.1692, found 450.1754.

Scheme S28. [(^{iPr}pybox)Ni(p-CHO-C_6H_4)]BAr^F_4 **29**

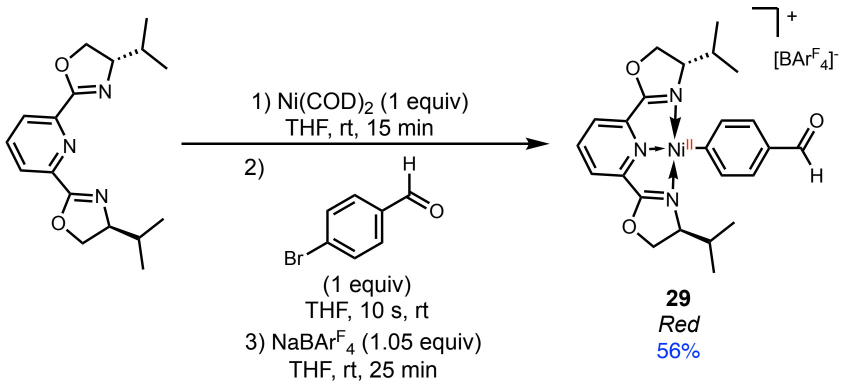

[(^{iPr}pybox)Ni(p-CHO-C_6H_4)]BAr^F_4 **29**. In a nitrogen filled glovebox, a 20 mL scintillation vial was charged with ^{iPr}pybox (15 mg, 50 µmol, 1.0 equiv), Ni(COD)$_2$ (14 mg, 50 µmol, 1.0 equiv), a magnetic stir bar, and THF (5 mL). The mixture was stirred at room temperature for 15 minutes, upon which a color change from light yellow to dark blue/purple was observed. A solution of 4-bromobenzaldehyde (9.2 mg, 50 µmol, 1.0 equiv) in THF (1 mL) was then added in one portion with stirring, upon which an immediate color change to red was observed. The mixture was then stirred for 10 seconds prior to adding a solution of $NaBAr^F_4$ (46 mg, 52 µmol, 1.05 equiv) in THF (1 mL) in one portion. The mixture was stirred for 25 minutes at room temperature after which it was concentrated *in vacuo* and triturated with pentane. The residue was then extracted with Et_2O, filtered through a plug of celite, and concentrated *in vacuo*. The solid was then washed and triturated with excess pentane. Recrystallization from Et_2O layered in pentane yielded the target

complex as a red powder (37 mg, 56% yield).

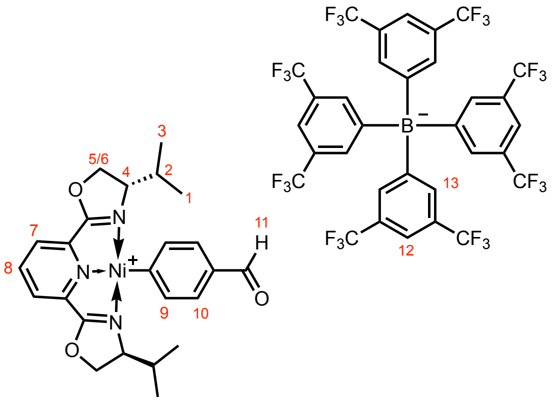

^{1}H NMR (400 MHz, acetone-*d*) δ 9.92 (s, 1H, H_{11}), 8.62 (t, J = 8.0 Hz, 1H, H_8), 8.25 (d, J = 7.9 Hz, 2H, H_7), 7.89 (br, 2H, H_{10}), 7.79 (br, 8H, H_{13}), 7.67 (br, 4H, H_{12}), 7.54 (d, J = 7.6 Hz, 2H, H_9), 5.12 (t, J = 9.8 Hz, 2H, $H_{5/6}$), 5.06 (dd, J = 9.6, 6.3 Hz, 2H, $H_{5/6}$), 4.10 (ddd, J = 9.8, 6.2, 3.4 Hz, 2H, H_4), 1.01 (m, 2H, H_2), 0.79 (d, J = 6.8 Hz, 6H, H_1/H_3), 0.53 (d, J = 7.1 Hz, 6H, H_1/H_3). *Decomposition of the compound was observed in acetone-d, resulting in formation of small amounts of biaryl.*

^{1}H NMR (500 MHz, CD_2Cl_2) δ 8.26 (t, *J* = 8.0 Hz, 1H, H_8), 7.88 (m, 4H, H_{10} and H_7), 7.78 (br, H_{13}), 7.55 (br, H_{12}), 7.48 (br, 2H, H_9), 4.84 (t, *J* = 9.9 Hz, 2H, $H_{5/6}$), 4.77 (dd, *J* = 9.6, 6.5 Hz, 2H, $H_{5/6}$), 3.80 (ddd, *J* = 10.2, 6.6, 3.2 Hz, 2H, H_4), 0.91 (m, 2H, H_2), 0.69 (d, *J* = 6.8 Hz, 6H, H_1/H_3), 0.45 (d, *J* = 7.0 Hz, 6H, H_1/H_3). *Due to broadening of the aryl peaks in CD_2Cl_2, the resonance for H_{11} was not observed.*

^{13}C NMR (126 MHz, CD_2Cl_2) δ 168.99, 162.16 (q, *J* = 49.7 Hz), 143.79, 142.67, 135.21, 129.28 (q, *J* = 32.7 Hz), 125.53, 124.99 (q, *J* = 272.5 Hz), 117.90, 75.24, 66.02, 28.87, 18.19, 13.71. *Due to peak broadening in CD_2Cl_2, resonances were not observed for the aryl portion of the molecule.*

^{19}F NMR (471 MHz, CD_2Cl_2) δ -62.78

UV-Vis (λ(ε), THF, 23 °C): 214 nm (75682 $M^{-1}cm^{-1}$), 270 nm (27754 $M^{-1}cm^{-1}$), 396 nm (4205 $M^{-1}cm^{-1}$).

HRMS (ESI-TOF) m/z: $[M+2NH_4]^{2+}$ calcd for $[C_{56}H_{48}BF_{24}N_5NiO_3]^{2+}$ 681.6410, found 681.6367.

Scheme S29. $[(^{iPr}pybox)Ni(p\text{-}COMe\text{-}C_6H_4)]BAr^F{}_4$ **30**

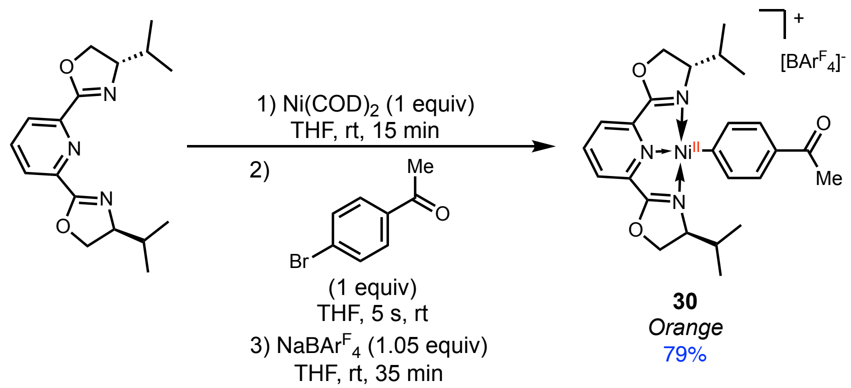

[(^{iPr}pybox)Ni(p-COMe-C_6H_4)]$BAr^F{}_4$ **30.** In a nitrogen filled glovebox, a 20 mL scintillation vial was charged with ^{iPr}pybox (20 mg, 66 µmol, 1.0 equiv), $Ni(COD)_2$ (18 mg, 66 µmol, 1.0 equiv), a magnetic stir bar, and THF (5 mL). The mixture was stirred at room temperature for 15 minutes, upon which a color change from light yellow to dark blue/purple was observed. A solution of 4-bromoacetophenone (13 mg, 66 µmol, 1.0 equiv) in THF (1 mL) was then added in one portion with stirring, upon which an immediate color change to dark brown/red was observed. The mixture was then stirred for 5 seconds prior to adding a solution of $NaBAr^F{}_4$ (62 mg, 70 µmol, 1.05 equiv) in THF (1 mL) in one portion. The mixture was stirred for 35 minutes at room temperature after which it was concentrated *in vacuo* and triturated with pentane. The resulting solid was then extracted with a 1:1 mixture of toluene and Et_2O, filtered through a plug of celite, and concentrated *in vacuo*. The solid was then washed and triturated with excess pentane. Recrystallization from Et_2O layered in pentane yielded the target complex as an orange powder (70 mg, 79% yield).

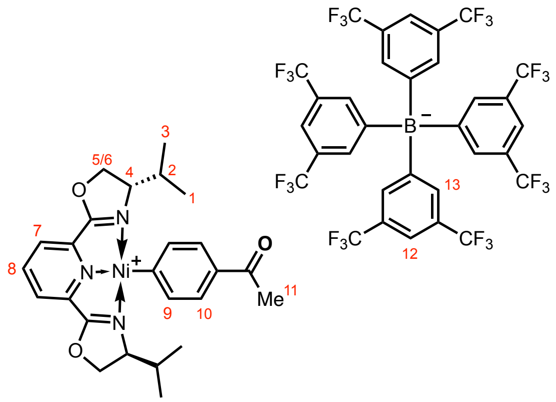

^{1}H NMR (500 MHz, acetone-*d*) δ 8.61 (t, J = 8.0 Hz, 1H, H_8), 8.24 (d, J = 8.0 Hz, 2H, H_7), 7.80 – 7.77

(br, H_{13}), 7.74 (d, J = 8.2 Hz, 2H, H_{10}), 7.67 (br, H_{12}), 7.63 (d, J = 8.2 Hz, 2H, H_9), 5.11 (t, J = 9.8 Hz, 2H, $H_{5/6}$), 5.06 (dd, J = 9.6, 6.2 Hz, 2H, $H_{5/6}$), 4.09 (ddd, J = 9.9, 6.2, 3.4 Hz, 2H, H_4), 2.50 (s, 3H, H_{11}), 1.04 (m, 2H, H_2), 0.78 (d, J = 6.9 Hz, 6H, H_1/H_3), 0.53 (d, J = 7.1 Hz, 6H, H_1/H_3).

^{1}H NMR (500 MHz, THF-*d*) δ 8.45 (t, J = 8.0 Hz, 1H, H_8), 8.11 (d, J = 7.9 Hz, 2H, H_7), 7.78 (br, H_{13}), 7.63 (s, 4H, H_9 and H_{10}), 7.57 (br, H_{12}), 5.00 – 4.88 (m, 4H, $H_{5/6}$), 3.96 (ddd, J = 9.0, 7.2, 3.5 Hz, 2H, H_4), 2.47 (s, 3H, H_{11}), 1.03 (m, 2H, H_2), 0.74 (d, J = 6.9 Hz, 6H, H_1/H_3), 0.52 (d, J = 7.1 Hz, 6H, H_1/H_3).

^{13}C NMR (126 MHz, THF-*d*) δ 197.07, 170.26, 162.99 (q, J = 49.9 Hz), 153.71, 144.54, 143.72, 137.12, 135.84, 135.77, 130.20 (q, J = 31.1 Hz), 126.33, 125.90, 125.69 (q, J = 272.3 Hz), 118.36, 75.88, 66.48, 29.72, 26.55, 18.32, 13.95.

^{19}F NMR (471 MHz, THF-*d*) δ -63.41

UV-Vis (λ(ε), THF, 23 °C): 216 nm (94260 $M^{-1}cm^{-1}$), 269 nm (34171 $M^{-1}cm^{-1}$), 403 nm (4991 $M^{-1}cm^{-1}$).

HRMS (ESI-TOF) m/z: $[M+2H]^{2+}$ calcd for $[C_{57}H_{44}BF_{24}N_3NiO_3]^{2+}$ 671.6222, found 671.6163.

Scheme S30. [(^{iPr}pybox)Ni(p-CF_3-C_6H_4)]BAr^F_4 **31**

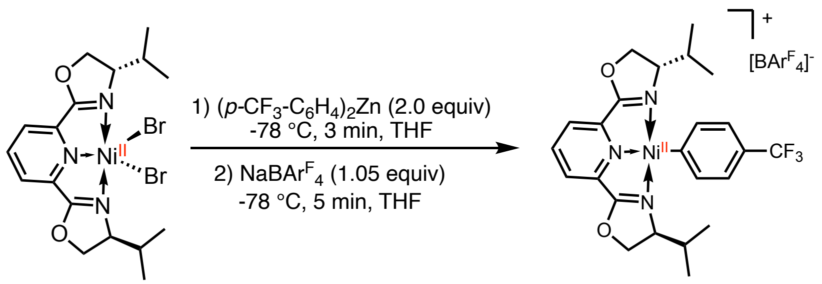

31
Orange
61%

[(^{iPr}pybox)Ni(p-CF_3-C_6H_4)]BAr^F_4 **31.** In a nitrogen filled glovebox, (^{iPr}pybox)$NiBr_2$ **S2** (40 mg, 77 µmol, 1.0 equiv) was dissolved in THF (5 mL) and cooled to -78 °C in a cold well. Separately, $NaBAr^F_4$ (72 mg, 81 µmol, 1.05 equiv) and (p-CF_3-C_6H_4)$_2$Zn (55 mg, 150 µmol, 2.0 equiv) were each diluted in THF (2 mL) and cooled to -78 °C. The solution of (p-CF_3-C_6H_4)$_2$Zn in THF was added dropwise to the solution of (^{iPr}pybox)$NiBr_2$ **S2** over the course of 1 minute while agitating the solution, upon which a rapid color change

to brown/orange was observed. After letting the mixture sit for 3 minutes, NaBAr$^F{}_4$ was added in one portion as a solution in THF and the reaction mixture was then left to sit for 5 minutes, after which the reaction mixture was filtered through a pad of celite and concentrated *in vacuo*. The resulting residue was washed and triturated with excess pentane, extracted with toluene, and filtered through a pad of celite. The filtrate was concentrated *in vacuo* and triturated with pentane to yield the target complex as a bright orange solid (64 mg, 61% yield).

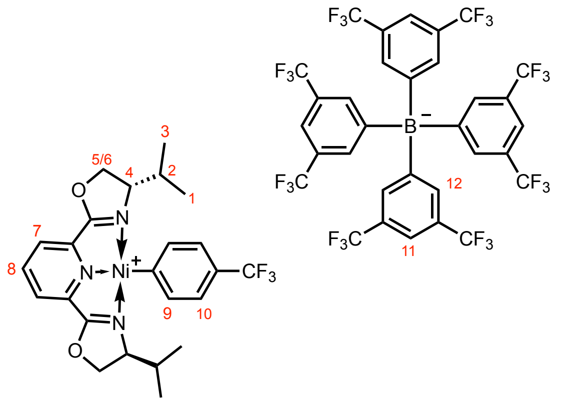

^{1}H NMR (500 MHz, THF-*d*) δ 8.46 (t, J = 8.0 Hz, 1H, H_8), 8.12 (d, J = 8.0 Hz, 2H, H_7), 7.86 – 7.73 (br, H_{12}), 7.70 (d, J = 7.8 Hz, 2H, H_{10}), 7.57 (br, H_{11}), 7.32 (d, J = 7.8 Hz, 2H, H_9), 4.98 – 4.89 (m, 4H, $H_{5/6}$), 3.94 (ddd, J = 10.0, 7.0, 3.4 Hz, 2H, H_4), 0.99 (dqq, J = 7.4, 3.7 Hz, 2H, H_2), 0.74 (d, J = 6.8 Hz, 6H, H_1/H_3), 0.53 (d, J = 7.1 Hz, 6H, H_1/H_3).

^{13}C NMR (126 MHz, THF-*d*) δ 170.32, 162.96 (d, J = 49.9 Hz), 150.77, 144.62, 143.71, 137.53, 135.74, 130.15 (q, J = 31.7 Hz), 128.17 (q, J = 31.7 Hz), 125.96, 125.96 (q, J = 271.1 Hz), 125.66 (q, J = 272.2 Hz), 123.14 (q, J = 3.8 Hz), 118.83 – 117.56 (m), 75.88, 66.42, 29.74, 18.22, 13.90.

^{19}F NMR (471 MHz, THF-*d*) δ -63.01, -63.41.

UV-Vis (λ(ε), THF, 23 °C): 216 nm (62746 $M^{-1}cm^{-1}$), 263 nm (24054 $M^{-1}cm^{-1}$), 377 nm (2957 $M^{-1}cm^{-1}$).

HRMS (ESI-TOF) m/z: [M–BAr$^F{}_4$]$^+$ calcd for $C_{24}H_{27}F_3N_3NiO_2$ 504.1409, found 504.1359.

Scheme S31. [($^{i\text{Pr}}$pybox)Ni(*p*-CN-C_6H_4)]BAr$^F{}_4$ **32**

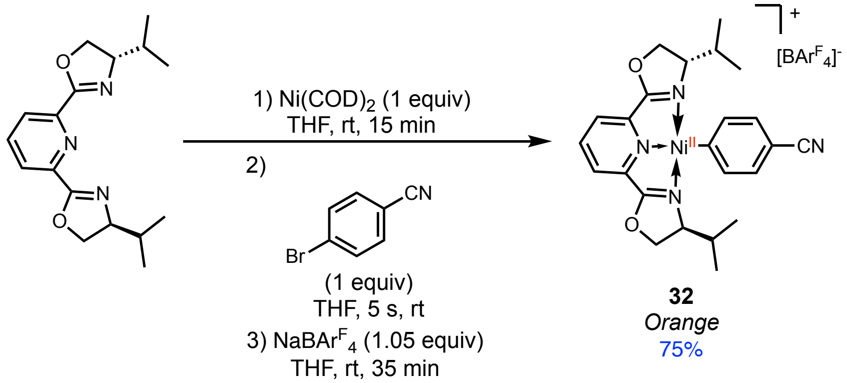

[(^{iPr}pybox)Ni(p-CN-C_6H_4)]BAr^F_4 **32.** In a nitrogen filled glovebox, a 20 mL scintillation vial was charged with ^{iPr}pybox (20 mg, 66 µmol, 1.0 eqiuv), $Ni(COD)_2$ (18 mg, 66 µmol, 1.0 equiv), a magnetic stir bar, and THF (5 mL). The mixture was stirred at room temperature for 15 minutes, upon which a color change from light yellow to dark blue/purple was observed. A solution of 4-bromobenzonitrile (12 mg, 66 µmol, 1.0 equiv) in THF (1 mL) was then added in one portion with stirring, upon which an immediate color change to dark brown/red was observed. The mixture was then stirred for 5 seconds prior to adding a solution of $NaBAr^F_4$ (62 mg, 70 µmol, 1.05 equiv) in THF (1 mL) in one portion. The mixture was stirred for 35 minutes at room temperature after which it was concentrated *in vacuo* and triturated with pentane. The resulting solid was then extracted with Et_2O, filtered through a plug of celite, and concentrated *in vacuo*. The solid was then washed and triturated with pentane. Recrystallization from Et_2O layered in pentane yielded the target complex as a light orange powder (66 mg, 76% yield).

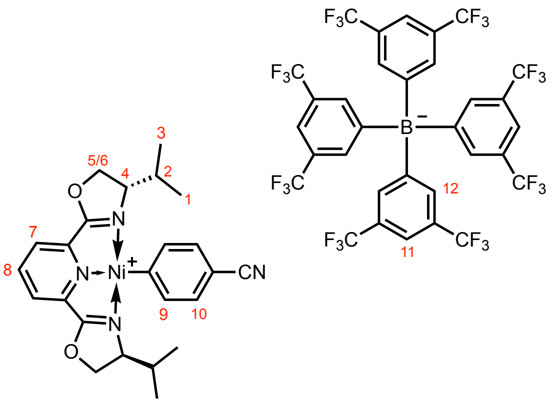

^{1}H NMR (500 MHz, THF-*d*) δ 8.46 (t, J = 8.0 Hz, 1H, H_8), 8.12 (d, J = 7.9 Hz, 2H, H_7), 7.79 (br, H_{12}), 7.70 (d, J = 7.9 Hz, 2H, H_{10}), 7.57 (br, H_{11}), 7.33 (d, J = 7.9 Hz, 2H, H_9), 5.00 – 4.92 (m, 4H, $H_{5/6}$), 3.93

(ddd, J = 10.0, 6.6, 3.5 Hz, 2H, H_4), 0.99 (qqd, J = 7.0, 7.0, 3.4 Hz, 2H, H_2), 0.75 (d, J = 6.9 Hz, 6H, H_1/H_3), 0.55 (d, J = 7.1 Hz, 6H, H_1/H_3). *Decomposition of the compound was observed, resulting in formation of small amounts of biaryl.*

^{1}H NMR (500 MHz, CD_2Cl_2) δ 8.27 (t, J = 7.9 Hz, 1H, H_8), 7.88 (d, J = 7.9 Hz, 2H, H_7), 7.73 (br, H_{12}), 7.62 (br, 2H, H_{10}), 7.56 (br, H_{11}), 7.33 (br, 2H, H_9), 4.83 (t, J = 9.8 Hz, 2H, $H_{5/6}$), 4.77 (dd, J = 9.6, 6.6 Hz, 2H, $H_{5/6}$), 3.76 (br, 2H, H_4), 0.70 (d, J = 6.6 Hz, 6H, H_1/H_3), 0.48 (d, J = 6.9 Hz, 6H, H_1/H_3). *Due to peak broadening and solvent overlap in CD_2Cl_2, a resonance was not observed for H_2.*

^{13}C NMR (126 MHz, CD_2Cl_2) δ 169.13, 162.19 (q, J = 49.6 Hz), 144.00, 142.68, 135.24, 129.30 (q, J = 30.4 Hz), 125.67, 125.02 (q, J = 272.6 Hz), 117.93, 75.27, 66.00, 28.95, 18.15, 13.69. *Due to peak broadening in CD_2Cl_2, resonances were not observed for the aryl portion of the molecule.*

^{13}C NMR (126 MHz, THF-*d*) δ 170.45, 162.98 (q, J = 49.9 Hz), 144.75, 143.72, 138.04, 135.76, 133.81, 130.84 – 129.65 (m), 129.52, 129.00, 126.04, 125.68 (q, J = 272.3 Hz), 118.37, 110.01, 75.92, 66.39, 29.82, 18.28, 13.92.

^{19}F NMR (471 MHz, CD_2Cl_2) δ -62.77

UV-Vis (λ(ε), THF, 23 °C): 216 nm (82273 $M^{-1}cm^{-1}$), 378 nm (4166 $M^{-1}cm^{-1}$).

FT-IR: (neat, ATR, cm^{-1}) 2967.60, 2239.30, 1353.12, 1272.51, 1112.90, 681.49

HRMS (ESI-TOF) m/z: $[M–BAr^F_4]^+$ calcd for $C_{24}H_{27}N_4NiO_2$ 461.1487, found 461.1532.

Scheme S32. [(4-Pyrrolidinyl-^{iPr}pybox)Ni(*p*-MeO-C_6H_4)]BAr^F_4 **33**

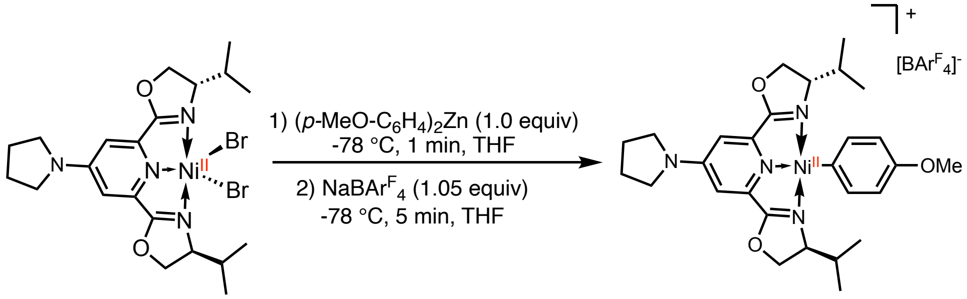

33
Yellow
64%

[(4-Pyrrolidinyl-^{iPr}pybox)Ni(p-MeO-C_6H_4)]BAr^F_4 **33**. In a nitrogen filled glovebox, (4-Pyrrolidinyl-^{iPr}pybox)$NiBr_2$ **S3** (25 mg, 42 µmol, 1 equiv) was dissolved in THF (5 mL) and cooled to -78 °C in a cold well. Separately, $NaBAr^F_4$ (39 mg, 45 µmol, 1.05 equiv) and (p-MeO-C_6H_4)$_2$Zn (12 mg, 42 µmol, 1.0 equiv) were each diluted in THF (1 mL) and cooled to -78 °C. The solution of (p-MeO-C_6H_4)$_2$Zn in THF was added dropwise to the solution of (4-Pyrrolidinyl-^{iPr}pybox)$NiBr_2$ **S3** over the course of 1 minute while agitating the solution, upon which a rapid color change from bright orange to dark yellow was observed. $NaBAr^F_4$ was added in one portion as a solution in THF and the solution was then left to sit for 5 minutes, after which the reaction mixture was filtered through a pad of celite and concentrated *in vacuo*. The resulting residue was triturated with pentane (2 x 1 mL), extracted with toluene, and filtered through a pad of celite. The filtrate was concentrated *in vacuo* to yield a yellow residue, which was washed (3 x 5 mL) and triturated with pentane to yield the target complex as an orange/yellow powder (38 mg, 64% yield).

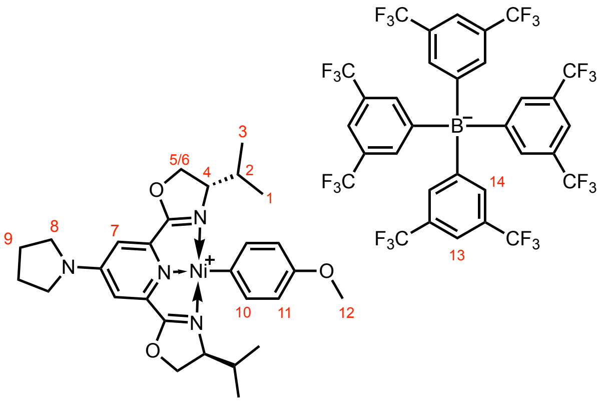

^{1}H NMR (500 MHz, CD_2Cl_2) 7.72 (br, 8H, H_{14}), 7.56 (br, 4H, H_{13}), 7.21 (d, J = 8.4 Hz, 2H, H_{10}), 6.75 (s, 2H, H_7), 6.70 (d, J = 8.5 Hz, 2H, H_{11}), 4.75 (t, J = 9.8 Hz, 2H, $H_{5/6}$), 4.69 (dd, J = 9.4, 6.3 Hz, 2H, $H_{5/6}$), 3.83 – 3.76 (m, 2H, H_4), 3.72 (s, 3H, H_{12}), 3.45 – 3.38 (m, 4H, H_8), 2.16 – 1.97 (m, 4H, H_9), 1.08 (m, 2H, H_2), 0.72 (d, J = 6.9 Hz, 6H, H_1/H_3), 0.52 (d, J = 7.1 Hz, 6H, H_1/H_3).

^{13}C NMR (126 MHz, CD_2Cl_2) δ 169.10, 162.15 (q, J = 49.9 Hz), 158.49, 155.14, 141.57, 136.57, 135.19, 129.26 (q, J = 32.4 Hz), 124.98 (q, J = 272.4 Hz), 117.87, 114.51, 113.43, 106.26, 74.48, 65.77, 55.35, 49.43, 28.71, 25.55, 18.47, 13.80.

^{19}F NMR (471 MHz, CD_2Cl_2) δ -62.86

UV-Vis (λ(ε), THF, 23 °C): 213 nm (81864 $M^{-1}cm^{-1}$), 340 nm (10533 $M^{-1}cm^{-1}$).

HRMS (ESI-TOF) m/z: $[M–BAr^F_4]^+$ calcd for $C_{28}H_{37}N_4NiO_3$ 535.2219, found 535.2152.

Scheme S33. [(4-CF_3-^{iPr}pybox)Ni(*p*-tol)]BAr^F_4 **34**.

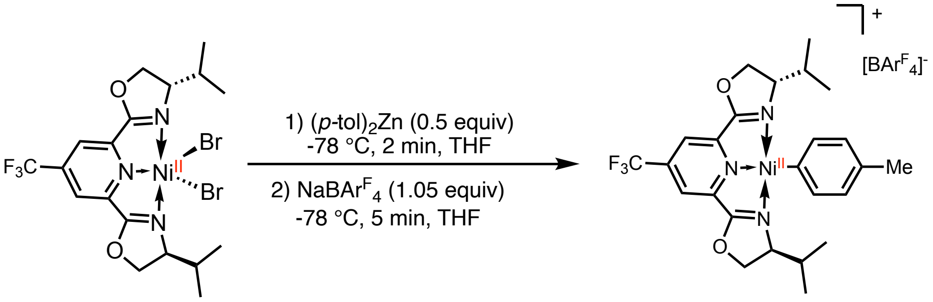

34
Red
62%

[(4-CF_3-^{iPr}pybox)Ni(*p*-tol)]BAr^F_4 **34**. In a nitrogen filled glovebox, (4-CF_3-^{iPr}pybox)$NiBr_2$ (22.5 mg, 38.3 μmol, 1.0 equiv) was dissolved in THF (5 mL) and cooled to -78 °C in a cold well. Separately, $NaBAr^F_4$ (35.6 mg, 40.2 μmol, 1.05 equiv) and $(p\text{-tol})_2Zn$ (4.74 mg, 19.1 μmol, 0.5 equiv) were each diluted in THF (1 mL) and cooled to -78 °C. The solution of $(p\text{-tol})_2Zn$ in THF was added dropwise to the solution of (4-CF_3-^{iPr}pybox)$NiBr_2$ over the course of 2 minutes while agitating the solution. $NaBAr^F_4$ was added in one portion as a solution in THF and the reaction mixture was then left to sit for 5 minutes with intermittent shaking, after which the reaction mixture was warmed to room temperature and concentrated *in vacuo*. The resulting residue was triturated with pentane. The residue was extracted with toluene and filtered through a pad of celite, then concentrated *in vacuo* to a volume of approximately 0.5 mL and diluted with pentane (10 mL). The supernatant was decanted and the resulting residue washed and triturated with excess pentane to yield a dark red powder (32.9 mg, 62% yield).

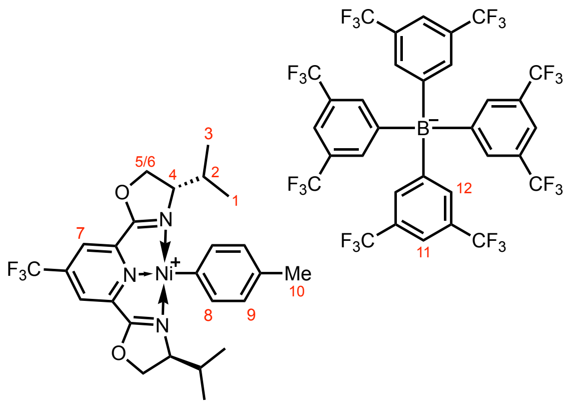

^{1}H NMR (400 MHz, THF-*d*) δ 8.59 (s, 2H, H_7), 7.78 (br, H_{12}), 7.57 (br, H_{11}), 7.22 (d, J = 7.9 Hz, 2H, H_8), 6.91 (d, J = 7.8 Hz, 2H, H_9), 5.01 (m, 4H, H_5/H_6), 4.05 (ddd, J = 10.0, 6.6, 3.5 Hz, 2H, H_4), 2.27 (s, 3H, H_{10}), 1.13 (m, 2H, H_2), 0.74 (d, J = 6.8 Hz, 6H, H_1/H_3), 0.55 (d, J = 7.0 Hz, 6H, H_1/H_3).

^{13}C NMR (126 MHz, THF-*d*) δ 169.36, 162.95 (q, 49.9 Hz), 144.84, 144.32 (m), 136.34, 135.74, 135.36, 130.22 (q, J = 31.3 Hz), 129.82, 128.87, 125.66 (q, J = 272.3 Hz), 122.58 (m), 118.33 (m), 76.09, 66.70, 29.67, 20.88, 18.36, 13.93. *Due to overlap with other signals, a resonance for the ^{iPr}pybox CF_3 could not be identified.*

^{19}F NMR (377 MHz, THF-*d*) δ -63.41, -65.18.

UV-Vis (λ(ε), THF, 23 °C): 214 nm (64615 $M^{-1}cm^{-1}$), 426 nm (1766 $M^{-1}cm^{-1}$).

HRMS (ESI-TOF) m/z: $[M–BAr^F{}_4]^+$ calcd for $C_{25}H_{29}F_3N_3NiO_2$ 518.1565, found 518.1415

Scheme S34. [(4-OMe-^{iPr}pybox)Ni(p-CF_3-C_6H_4)]$BAr^F{}_4$ **35**.

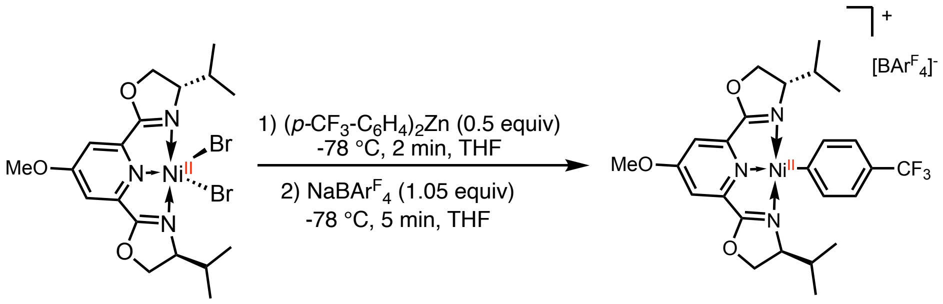

35
Yellow
54%

[(4-OMe-^{iPr}pybox)Ni(p-CF_3-C_6H_4)]BAr$^F_4$ **35**. In a nitrogen filled glovebox, (4-OMe-^{iPr}pybox)$NiBr_2$ (40.0 mg, 72.7 µmol, 1.0 equiv) was dissolved in THF (8 mL) and cooled to -78 °C in a cold well. Separately, NaBAr$^F_4$ (68.0 mg, 76.4 µmol, 1.05 equiv) and (p-CF_3-C_6H_4)$_2$Zn (12.9 mg, 36.4 µmol, 0.5 equiv) were each diluted in THF (1 mL) and cooled to -78 °C. The solution of (p-CF_3-C_6H_4)$_2$Zn in THF was added dropwise to the solution of (4-OMe-^{iPr}pybox)$NiBr_2$ over the course of 2 minutes while agitating the solution. NaBAr$^F_4$ was added in one portion as a solution in THF and the reaction mixture was then left to sit for 5 minutes with intermittent shaking, after which the reaction mixture was warmed to room temperature and concentrated *in vacuo* to a volume of approximately 0.5 mL. Pentane (10 mL) was then added and the supernatant removed, the resulting residue was then triturated with pentane (2 x 1 mL). The resulting residue was then extracted with 1:1 Et_2O:Toluene and filtered through a pad of celite. The filtrate was concentrated *in vacuo* to approximately 0.5 mL volume and diluted with pentane (10 mL), after which the supernatant was decanted, and the remaining orange residue was dried *in vacuo*. The residue was triturated with excess pentane to yield the product as a yellow powder (55.2 mg, 54% yield).

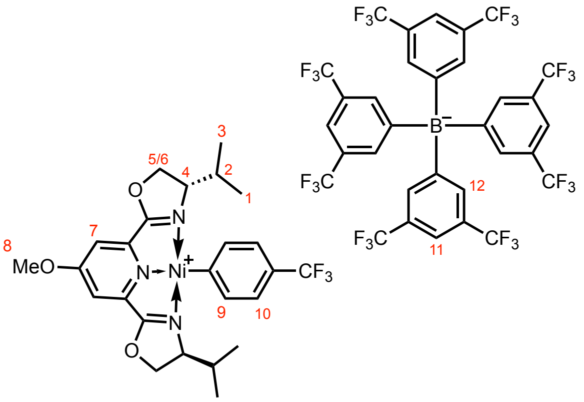

^{1}H NMR (400 MHz, THF-*d*) δ 7.78 (br, H_{12}), 7.69 (m, 4H, H_{10} and H_7), 7.57 (br, H_{11}), 7.30 (d, J = 7.8 Hz, 2H, H_9), 4.93 (m, 4H, $H_{5/6}$), 4.10 (s, 3H, H_8), 3.90 (ddd, J = 9.9, 6.7, 3.4 Hz, 2H, H_4), 0.94 (m, 2H, H_2), 0.74 (d, J = 6.8 Hz, 6H, H_1/H_3), 0.52 (d, J = 7.1 Hz, 6H, H_1/H_3).

^{13}C NMR (101 MHz, THF-*d*) δ 172.98, 170.24, 162.98 (q, J = 49.8 Hz), 144.71, 137.74, 135.76, 130.24 (q, J = 32.0), 129.07, 128.01 (m), 126.02 (q, J = 271.0 Hz), 125.68 (q, J = 272.2 Hz), 123.03 (q, J = 4.0 Hz), 118.35 (m), 112.12, 75.79, 66.49, 58.75, 29.78, 18.19, 13.91.

^{19}F NMR (377 MHz, THF-*d*) δ -62.98, -63.43.

UV-Vis (λ(ε), THF, 23 °C): 215 nm (75473 $M^{-1}cm^{-1}$), 379 nm (1545 $M^{-1}cm^{-1}$).

HRMS (ESI-TOF) m/z: $[M–BAr^F{}_4]^+$ calcd for $C_{25}H_{29}F_3N_3NiO_3$ 534.1514, found 534.1523

Scheme S35. $[(4\text{-}CF_3\text{-}^{iPr}pybox)Ni(p\text{-}CF_3\text{-}C_6H_4)]BAr^F{}_4$ **36**

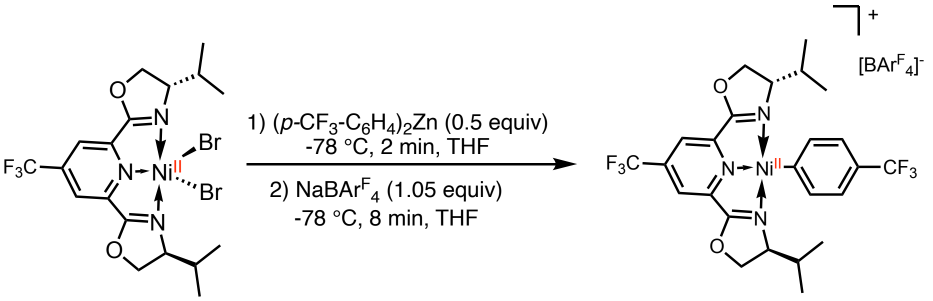

36
Orange
75%

$[(4\text{- }CF_3\text{-}^{iPr}pybox)Ni(p\text{-}CF_3\text{-}C_6H_4)]BAr^F{}_4$ **36**. In a nitrogen filled glovebox, $(4\text{-}CF_3\text{-}^{iPr}pybox)NiBr_2$ **S7** (40 mg, 68 μmol, 1.0 equiv) was dissolved in THF (5 mL) and cooled to -78 °C in a cold well. Separately, $NaBAr^F{}_4$ (63.3 mg, 71 μmol, 1.05 equiv) and $(p\text{-}CF_3\text{-}C_6H_4)_2Zn$ (12 mg, 34 μmol, 0.5 equiv) were each diluted in THF (1 mL) and cooled to -78 °C. The solution of $(p\text{-}CF_3\text{-}C_6H_4)_2Zn$ in THF was added dropwise to the solution of $(4\text{-}CF_3\text{-}^{iPr}pybox)NiBr_2$ **S7** over the course of 2 minutes while agitating the solution. $NaBAr^F{}_4$ was added in one portion as a solution in THF and the solution was then left to sit for 8 minutes, after which the reaction mixture was warmed to room temperature and concentrated *in vacuo*. The resulting residue was triturated with pentane and washed with toluene (2 x 1 mL). The resulting orange residue was then extracted with 1:1 Et_2O:Toluene and filtered through a pad of celite. The filtrate was concentrated *in vacuo* to approximately 3 mL volume and diluted with pentane (10 mL), after which the supernatant was decanted and the remaining orange residue was dried *in vacuo*. The residue was triturated with excess pentane to yield a dark orange powder (73 mg, 75% yield).

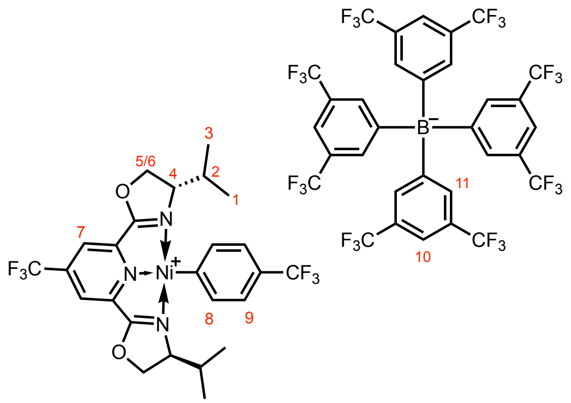

^{1}H NMR (400 MHz, THF-*d*) 8.64 (s, 2H, H_7), 7.78 (br, H_{11}), 7.70 (d, J = 7.8 Hz, 2H, H_9), 7.57 (br, H_{10}), 7.34 (d, J = 7.8 Hz, 2H, H_8), 5.04 (br, 4H, $H_{5/6}$), 4.01 (br, 2H, H_4), 1.01 (m, 2H, H_2), 0.75 (d, J = 7.0 Hz, 6H, H_1/H_3), 0.55 (d, J = 7.0 Hz, 6H, H_1/H_3). *Compound decomposes over long scan times, complicating collection of ^{13}C NMR spectrum.*

^{19}F NMR (377 MHz, benzene-*d*) δ -62.14, -62.15, -64.95.

UV-Vis (λ(ε), THF, 23 °C): 212 nm (77890 $M^{-1}cm^{-1}$), 412 nm (5095 $M^{-1}cm^{-1}$).

HRMS (ESI-TOF) m/z: $[M–BAr^{F}_{4}]^{+}$ calcd for $C_{25}H_{26}F_6N_3NiO_2$ 572.1283, found 572.1285.

Scheme S36. $[(^{iPr}pybox)Ni(Mes)]BAr^{F}_{4}$ **16**

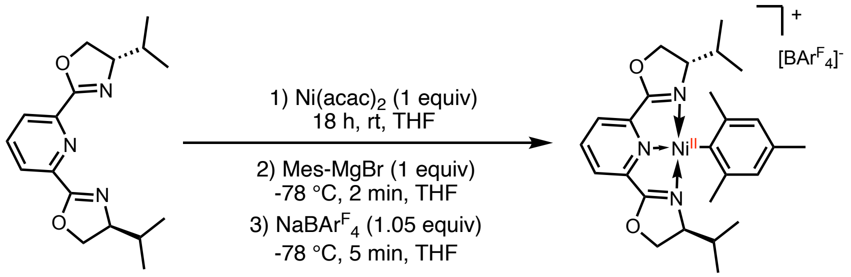

16
Red
89%

$[(^{iPr}pybox)Ni(Mes)]BAr^{F}_{4}$ **16**. In a nitrogen filled glovebox, $Ni(acac)_2$ (10 mg, 39 µmol, 1.0 equiv) and ^{iPr}pybox (12 mg, 39 µmol, 1.0 equiv) were stirring overnight in THF (5 mL) at room temperature to yield a cloudy teal solution. The solution was then cooled to -78 °C and a pre-cooled solution of MesMgBr (39 µL, 1.0 M, 39 µmol, 1.0 eqiuv) in THF (1 mL) was added dropwise, upon which rapid color change to

orange/red was observed. After letting sit for 2 minutes, a pre-cooled solution of $NaBAr^F_4$ (36 mg, 41 µmol, 1.05 equiv) in THF (1 mL) was added in one portion, after which the solution was allowed to sit for 5 minutes. The reaction mixture was then warmed to room temperature and filtered through a pad of alumina, after which the resulting solution was concentrated to yield a red residue. The residue was washed (3 x 2 mL) and triturated with pentane to yield the target product as a dark red crystalline solid (46.5 mg, 89 %).

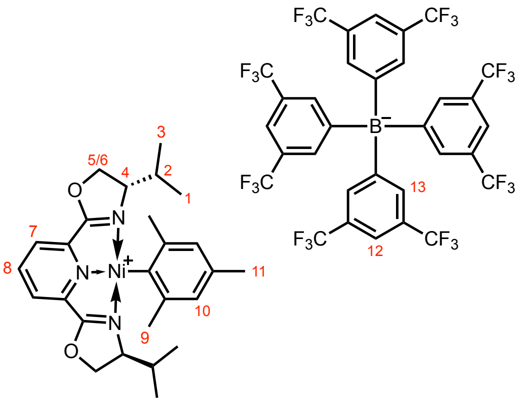

^{1}H NMR (500 MHz, benzene-*d*) δ 8.37 (br, 8H, H_{13}), 7.61 (br, 4H, H_{12}), 6.64 (t, J = 7.9 Hz, 1H, H_8), 6.58 (s, 2H, H_{10}), 6.30 (d, J = 7.9 Hz, 2H, H_7), 3.74 (dd, J = 9.7, 6.6 Hz, 2H, $H_{5/6}$), 3.54 (t, J = 9.9 Hz, 2H, $H_{5/6}$), 2.99 (ddd, J = 10.2, 6.6, 3.4 Hz, 2H, H_4), 2.82 (s, 6H, H_9), 2.18 (s, 3H, H_{11}), 1.20 (m, 2H, H_2), 0.38 (d, J = 6.9 Hz, 6H, H_1/H_3), -0.02 (d, J = 7.1 Hz, 6H, H_1/H_3).

^{13}C NMR (126 MHz, benzene-*d*) δ 168.09, 162.76 (q, J = 49.5 Hz), 142.68, 141.77, 141.22, 135.48, 135.39, 132.62, 130.01 (q, J = 30.6 Hz), 127.03, 125.18 (q, J = 272.8 Hz), 123.89, 118.15, 73.94, 66.61, 28.55, 23.97, 20.49, 17.99, 13.26.

^{19}F NMR (471 MHz, benzene-*d*) δ -61.15

UV-Vis (λ(ε), THF, 23 °C): 215 nm (91303 $M^{-1}cm^{-1}$), 282 nm (36885 $M^{-1}cm^{-1}$), 415 nm (4258 $M^{-1}cm^{-1}$).

HRMS (ESI-TOF) m/z: $[M–BAr^F_4]^+$ calcd for $C_{26}H_{34}N_3NiO_2$ 478.2005, found 478.2031.

Scheme S37. $[(^{Me}pybox)Ni(Ph)]BAr^F_4$ **19**

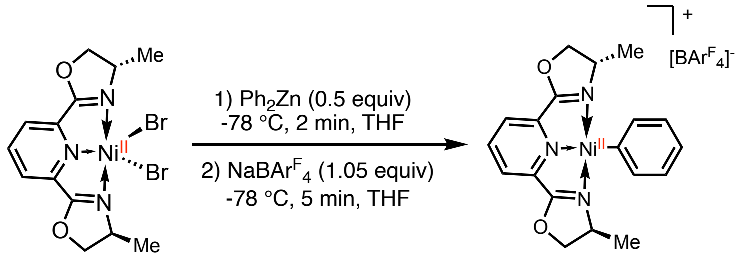

19
Red
28%

[(^{Me}pybox)Ni(Ph)]$BAr^F{}_4$ **19**. In a nitrogen filled glovebox, (^{Me}pybox)$NiBr_2$ **S8** (23.2 mg, 50 µmol, 1.0 equiv) was dissolved in THF (4 mL) and cooled to -78 °C in a cold well. Separately, $NaBAr^F{}_4$ (46.5 mg, 53 µmol, 1.05 equiv) and Ph_2Zn (5.5 mg, 25 µmol, 0.5 equiv) were diluted in THF (1 mL and 2 mL, respectively) and cooled to -78 °C. The solution of Ph_2Zn in THF was added dropwise to the solution of (^{Me}pybox)$NiBr_2$ **S8** while agitating the solution. The solution was then left to sit at -78 °C for 2 minutes with intermittent shaking, then $NaBAr^F{}_4$ was added in one portion as a solution in THF. The reaction mixture was then left to sit for 5 minutes, after which the reaction mixture was passed through a cold, THF-rinsed plug of celite and concentrated *in vacuo*. The residue was then extracted with toluene and filtered through a pad of celite, then concentrated *in vacuo*. The resulting solid was triturated and washed with excess pentane to yield the target complex as a red powder (17.2 mg, 28% yield).

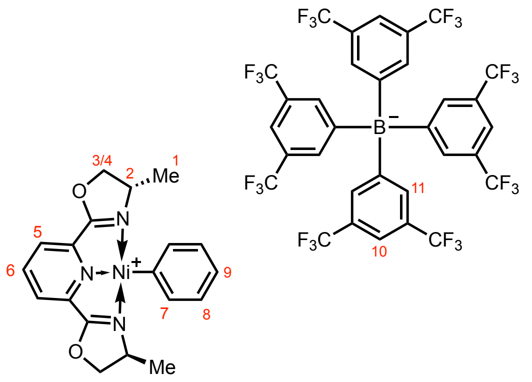

^{1}H NMR (500 MHz, CD_2Cl_2) δ 8.23 (t, J = 7.9 Hz, 1H, H_6), 7.85 (d, J = 8.0 Hz, 2H, H_5), 7.72 (br, H_{11}), 7.56 (br, H_{10}), 7.35 (d, J = 7.2 Hz, 2H, H_7), 7.06 (t, J = 7.3 Hz, 2H, H_8), 6.98 (t, J = 7.3 Hz, 1H, H_9), 5.03 (t, J = 9.4 Hz, 2H, $H_{3/4}$), 4.55 (dd, J = 9.1, 6.7 Hz, 2H, $H_{3/4}$), 3.99 (ddq, J = 9.6, 6.7, 6.7 Hz, 2H, H_2), 0.74 (d,

J = 6.6 Hz, 6H, H_1).

^{13}C NMR (126 MHz, CD_2Cl_2) δ 168.53, 162.15 (q, *J* = 50.0 Hz), 143.06, 142.98, 139.71, 136.30, 135.20, 129.27 (q, *J* = 30.3 Hz), 127.59, 125.27, 124.99 (q, *J* = 272.4 Hz), 124.95, 117.90, 81.25, 57.85, 20.50.

^{19}F NMR (471 MHz, CD_2Cl_2) δ -62.82

UV-Vis (λ(ε), THF, 23 °C): 208 nm (119096 $M^{-1}cm^{-1}$), 408 nm (3912 $M^{-1}cm^{-1}$).

HRMS (ESI-TOF) m/z: $[M–BAr^F{}_4]^+$ calcd for $C_{19}H_{20}N_3NiO_2$ 380.0909, found 380.1204.

Scheme S38. (dppe)Ni(*p*-CF_3-*o*-tol)Br **7**

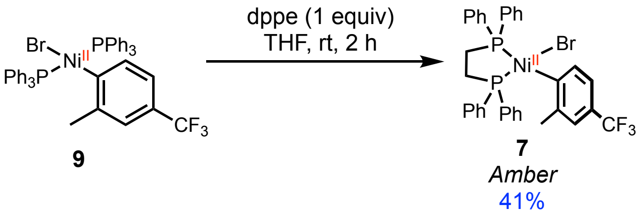

(dppe)Ni(*p*-CF_3-*o*-tol)Br **7**. In a nitrogen filled glovebox, $(PPh_3)_2$Ni(*p*-CF_3-*o*-tol)Br **9** (91.1 mg, 163 µmol, 1.0 equiv) and 1,2-Bis(diphenylphosphino)ethane (dppe) (64.8 mg, 163 µmol, 1.0 equiv) were combined in a 20 mL scintillation vial along with a magnetic stir bar and THF (10 mL). The resulting solution was stirred at room temperature for 2 hours, then concentrated to approximately 0.5 mL and diluted with pentane (10 mL). The supernatant was removed and the resulting orange precipitate dried *in vacuo* and triturated with pentane. The material was then extracted with 1:1 Et_2O:THF and filtered through a pad of celite and concentrated to dryness. The solid was then washed with Et_2O (2 x 2 mL) and pentane (2 x 2 mL), concentrated *in vacuo* and triturated with pentane. The resulting solid was recrystallized from THF layered in pentane to yield the target product as an orange powder (46.4 mg, 41% yield).

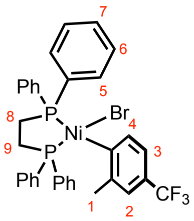

^{1}H NMR (500 MHz, benzene-*d*) δ 8.22 (m, 2H, $H_{5/6}$), 7.91 (dd, *J* = 11.1, 7.8 Hz, 2H, $H_{5/6}$), 7.78 (m, 2H,

$H_{5/6}$), 7.66 (t, 7.0 Hz, 1H, H_7), 7.12 (m, 5H, $H_{5/6}$, H_3 and H_7, *overlapping*), 7.02 (d, J = 7.5 Hz, 1H, H_4), 6.95 (m, 3H, $H_{5/6}$ and H_7), 6.87 (td, J = 7.6, 1H, H_7), 6.82 (s, 1H, H_2), 6.71 (td, J = 7.8, 2.5 Hz, 2H, $H_{5/6}$), 6.44 (m, 2H, $H_{5/6}$), 2.49 (s, 3H, H_1), 1.94 – 1.75 (m, 2H, $H_{8/9}$), 1.67 – 1.47 (m, 1H, $H_{8/9}$), 1.23 – 1.13 (m, 1H, $H_{8/9}$).

^{13}C NMR (126 MHz, benzene-*d*) δ 167.78 – 166.04 (m), 143.94 (t, J = 2.3 Hz), 136.61 (m), 134.96 (d, J = 11.2 Hz), 134.63 (d, J = 11.3 Hz), 133.25 (d, J = 9.6 Hz), 132.31 (d, J = 1.9 Hz), 132.01 (d, J = 1.8 Hz), 131.75 (d, J = 8.3 Hz), 131.43, 131.19 (d, J = 2.2 Hz), 131.06, 130.80, 130.53 (d, J = 2.3 Hz), 130.43, 130.34 (d, J = 2.6 Hz), 129.63 (d, J = 5.0 Hz), 129.16 (t, J = 10.2 Hz), 128.72 (d, J = 9.5 Hz), 126.40 (q, J = 270.9 Hz), 125.32 (q, J = 31.4 Hz), 124.82 – 124.49 (m), 119.85, 28.34 (dd, J = 27.1, 21.0 Hz), 26.48, 22.33 (dd, J = 25.2, 12.1 Hz).

^{19}F NMR (471 MHz, benzene-*d*) δ -60.92.

^{31}P NMR (202 MHz, benzene-*d*) δ 56.51 (d, J = 21.4 Hz), 35.89 (d, J = 21.5 Hz).

UV-Vis (λ(ε), THF, 23 °C): 409 nm (1707 $M^{-1}cm^{-1}$), 268 nm (20106 $M^{-1}cm^{-1}$), 209 nm (51444 $M^{-1}cm^{-1}$)

HRMS (ESI-TOF) m/z: $[M-Br]^+$ calcd for $C_{34}H_{30}F_3NiP_2$ 615.1128, found 615.1088.

Scheme S39. $(PPh_3)_2Ni(p\text{-}CF_3\text{-}C_6H_4)Br$ **8**.

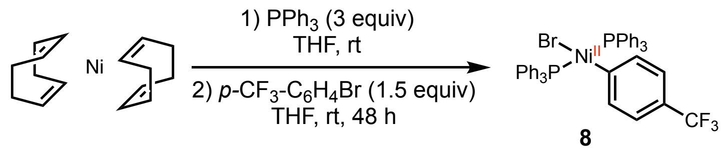

8
Yellow
75%

$(PPh_3)_2Ni(p\text{-}CF_3\text{-}C_6H_4)Br$ **8**. Complex **8** was synthesized following a modified literature procedure.[16] In a nitrogen filled glovebox, $Ni(COD)_2$ (50.0 mg, 182 μmol, 1.0 equiv), PPh_3 (143.0 mg, 545 μmol, 3.0 equiv), and a magnetic stir bar were combined in a 20 mL scintillation vial and combined with THF (8 mL). 1-bromo-2-methyl-4-(trifluoromethyl)benzene (38.2 μL, 273 μmol, 1.5 equiv) was added to the resulting dark red solution. The reaction was then stirred at room temperature for 2 days, resulting in a gradual color change to murky yellow. The resulting solution was then concentrated *in vacuo* and triturated with pentane.

The residue was then extracted with Et_2O and filtered through celite, then concentrated to dryness and washed with excess pentane. Recrystallization from THF layered in pentane at -35 °C resulted in precipitated of the product as a yellow solid (110 mg, 75% yield).

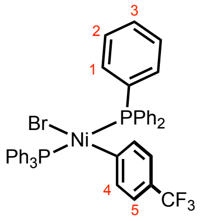

^{1}H NMR (500 MHz, benzene-*d*) δ 7.78 – 7.70 (m, 12H, H_2), 7.05 – 6.92 (m, 20H, H_5 and $H_{1/3}$), 6.37 (d, J = 7.9 Hz, 2H, H_4).

Scheme S40. $(PPh_3)_2$Ni(*p*-CF_3-*o*-tol)Br **9**

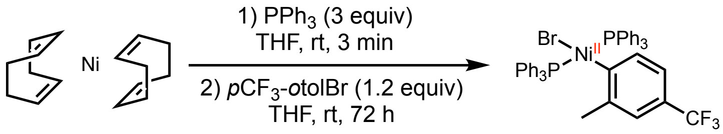

9
Yellow
67%

$(PPh_3)_2$Ni(*p*-CF_3-*o*-tol)Br **9**. In a nitrogen filled glovebox, $Ni(COD)_2$ (40.0 mg, 145 µmol, 1.0 equiv), PPh_3 (114.0 mg, 436 µmol, 3.0 equiv), and a magnetic stir bar were added to a 20 mL scintillation vial and combined with THF (8 mL). The resulting dark red solution was stirred at room temperature for 3 minutes, followed by addition of 1-bromo-2-methyl-4-(trifluoromethyl)benzene (27.1 µL, 175 µmol, 1.2 equiv). The solution was then allowed to stir for 3 days after which the resulting yellow solution was concentrated to dryness and triturated with pentane. The resulting residue was extracted with Et_2O/THF (4:1) and filtered through a pad of celite, then concentrated *in vacuo*. The resulting solid was washed (3 x 4 mL) and triturated with pentane to yield the target complex as a yellow powder (79.8 mg, 67% yield).

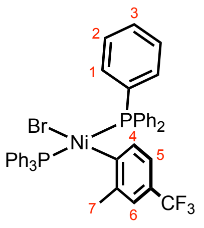

^{1}H NMR (500 MHz, benzene-*d*) δ 7.79 – 7.68 (m, 12H, H_2), 7.25 (d, J = 7.8 Hz, 1H, H_4), 7.03 – 6.95 (m, 18H, H_1/H_3), 6.34 (d, J = 7.8 Hz, 1H, H_5), 6.32 (s, 1H, H_6), 2.45 (s, 3H, H_7).

^{13}C NMR (126 MHz, benzene-*d*) δ 164.88 (t, J = 32.5 Hz), 143.72 (t, J = 3.3 Hz), 136.45 (t, J = 4.3 Hz), 135.13 (t, J = 5.4 Hz), 132.22 (t, J = 21.8 Hz), 129.97, 127.99 (*overlapping with solvent signal)*, 126.07 (m), 125.26 (m), 124.50 (q, J = 3.9 Hz), 119.37 (m), 26.35.

^{19}F NMR (471 MHz, benzene-*d*) δ -61.24.

^{31}P NMR (202 MHz, benzene-*d*) δ 22.71.

UV-Vis (λ(ε), THF, 23 °C): 270 nm (14124 $M^{-1}cm^{-1}$), 210 nm (48700 $M^{-1}cm^{-1}$)

HRMS (ESI-TOF) m/z: $[M-Br]^+$ calcd for $C_{44}H_{36}F_3NiP_2$ 741.1598, found 741.1572.

Scheme S41. $(PPh_3)_2$Ni(*p*-CF_3-*o*-tol)Br **10**

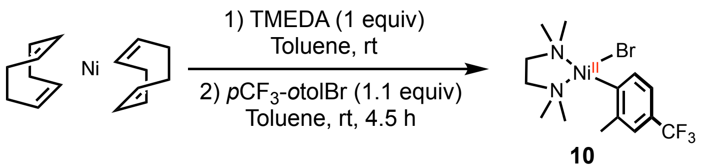

(TMEDA)Ni(*p*-CF_3-*o*-tol)Br **10**. In a nitrogen filled glovebox, $Ni(COD)_2$ (80.0 mg, 291 µmol, 1.0 equiv), tetramethylethylenediamine (TMEDA) (43.8 µL, 291 µmol, 1.0 equiv), and a magnetic stir bar were added to a 20 mL scintillation vial and combined with toluene (6 mL), followed by addition of 1-bromo-2-methyl-4-(trifluoromethyl)benzene (49.7 µL, 320 µmol, 1.1 equiv). The solution was then allowed to stir for 4.5 hours after which the resulting bright orange solution was concentrated to approximately 0.5 mL and diluted with pentane (10 mL). The resulting suspension was stirred for 2 minutes, after which the supernatant was removed and the precipitate concentrated *in vacuo*. The resulting orange solid was further triturated with

excess pentane to yield the target product (106 mg, 88% yield).

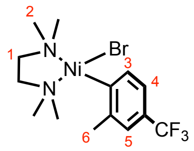

^{1}H NMR (500 MHz, benzene-*d*) δ 7.71 (d, *J* = 7.8 Hz, 2H, H_3), 7.17 (m, H_4, *overlapping with solvent signal*), 7.15 (s, 1H, H_5), 3.49 (s, 3H, H_6), 2.21 (br, 3H, H_2), 2.09 (br, 3H, H_2), 1.71 (br, 3H, H_2), 1.44 (br, 1H, H_1), 1.33 – 1.19 (br, 1H, H_1), 1.04 (br, 4H, H_1/H_2), 0.96 (br, 1H, H_1).

^{13}C NMR (126 MHz, benzene-*d*) δ 155.97, 144.34, 136.61, 126.52 (q, *J* = 271.0 Hz), 124.74 (q, *J* = 31.1 Hz), 121.92 (q, *J* = 3.3 Hz), 118.26 (q, *J* = 3.6 Hz), 60.31, 56.37, 49.21, 48.77, 46.79, 46.65, 27.24.

^{19}F NMR (471 MHz, benzene-*d*) δ -60.82.

UV-Vis (λ(ε), THF, 23 °C): 318 nm (2734 $M^{-1}cm^{-1}$), 256 nm (9901 $M^{-1}cm^{-1}$), 210 nm (21058 $M^{-1}cm^{-1}$)

HRMS (ESI-TOF) m/z: $[M-Br]^+$ calcd for $C_{14}H_{22}F_3NiN_2$ 333.1089, found 333.1050

8. Electrochemical Characterization

All electrochemical measurements were carried out using a three-electrode configuration with a 3 mm (0.071 cm^2) glassy carbon working electrode (MF-2012, BASi), a platinum wire counter electrode (CHI115 from CH Instruments, Inc.), and a 0.5 mm diameter silver wire reference electrode (MF-2017, BASi) in a nitrogen filled glovebox. Ferrocene (Fc/Fc^+) added as an internal reference to calibrate each sample. Tetrabutylammonium hexafluorophosphate was dried under vacuum (10 mTorr) overnight at 90 ºC. Cyclic voltammograms (CVs) are plotted in IUPAC notation with negative currents corresponding to reduction.

For reversible and quasi-reversible redox events, $E_{1/2}$ is used as an approximation of the formal potential (E°) and is taken as the average of the anodic (E_{pa}) and cathodic (E_{pc}) peak potentials. For complexes in which re-oxidation of nickel(I) was obscured by decomposition peaks, $E_{p/2}$ was used instead. For fully irreversible events, E_{pa} was obtained with freshly polished working electrode and at the same scan

rate across different complexes. While E_{pa} does not reflect the absolute value of E°, E_{pa} was used to compare relative ease of oxidation between each complex. THF was used as a solvent unless otherwise specific. In the case of complexes **26** and **36**, oxidation occurred outside the solvent window, so DCM was used instead.

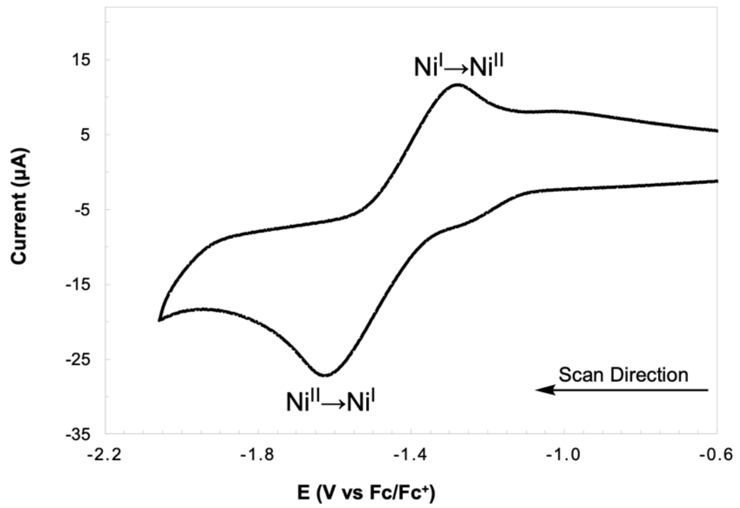

Figure S21. CV of [(^{iPr}pybox)Ni(Ph)]BAr$^F_4$ **6.** $E_{1/2}$ (Ni^{II}/Ni^{I}) = -1.45 V (vs Fc/Fc$^+$). Solvent = THF; temperature = 295 K; scan rate = 1000 mV/s; [Ni] = 1.0 mM; [$TBAPF_6$] = 100 mM.

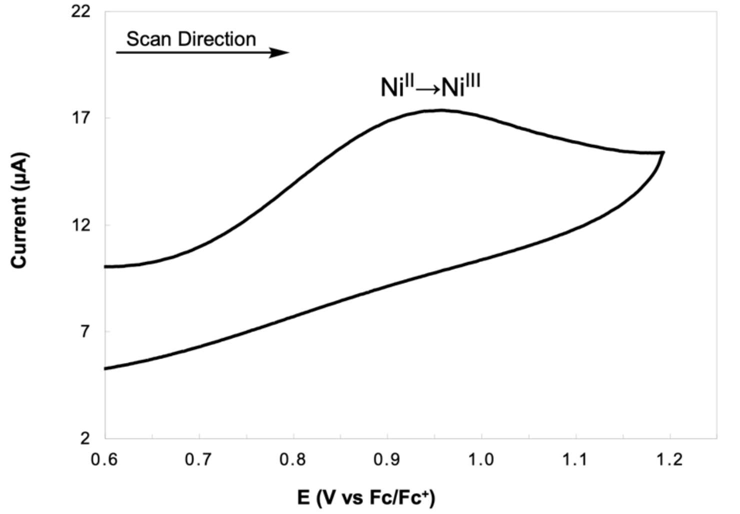

Figure S22. CV of [(^{iPr}pybox)Ni(Ph)]BAr$^F_4$ **6.** E_{pa} (Ni^{III}/Ni^{II}) = 0.91 V (vs Fc/Fc$^+$). Solvent = THF; temperature = 295 K; scan rate = 100 mV/s; [Ni] = 1.0 mM; [$TBAPF_6$] = 100 mM.

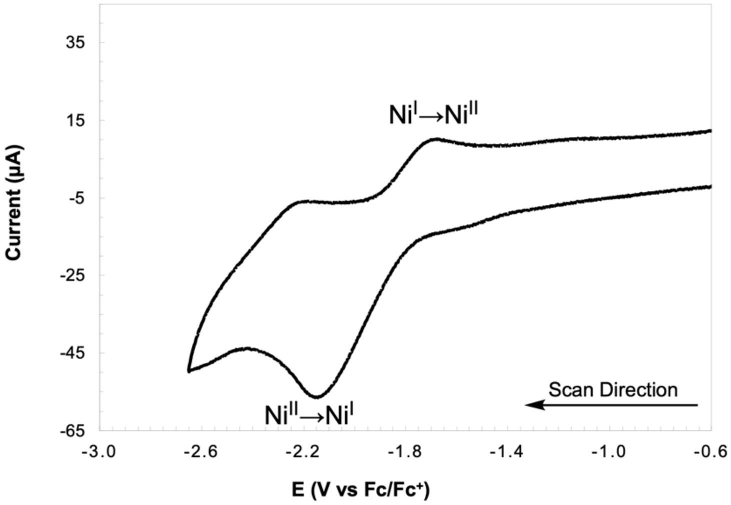

Figure S23. CV of [(4-Pyrrolidinyl-$^{i\mathrm{Pr}}$pybox)Ni(Ph)]BAr$^F_4$ **21.** $E_{1/2}$ (Ni^{II}/Ni^{I}) = -1.91 (vs Fc/Fc$^+$). Solvent = THF; temperature = 295 K; scan rate = 3030 mV/s; [Ni] = 1.0 mM; [TBAPF$_6$] = 100 mM.

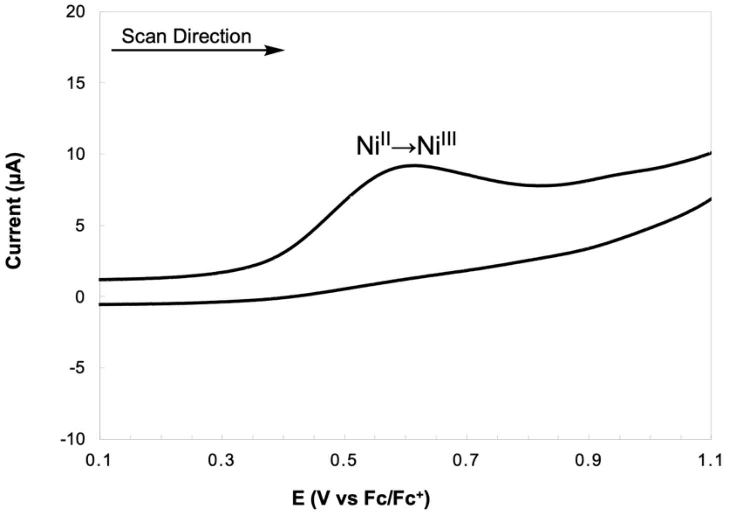

Figure S24. CV of [(4-Pyrrolidinyl-$^{i\mathrm{Pr}}$pybox)Ni(Ph)]BAr$^F_4$ **21.** E_{pa} (Ni^{III}/Ni^{II}) = 0.62 V (vs Fc/Fc$^+$). Solvent = THF; temperature = 295 K; scan rate = 100 mV/s; [Ni] = 1.0 mM; [TBAPF$_6$] = 100 mM.

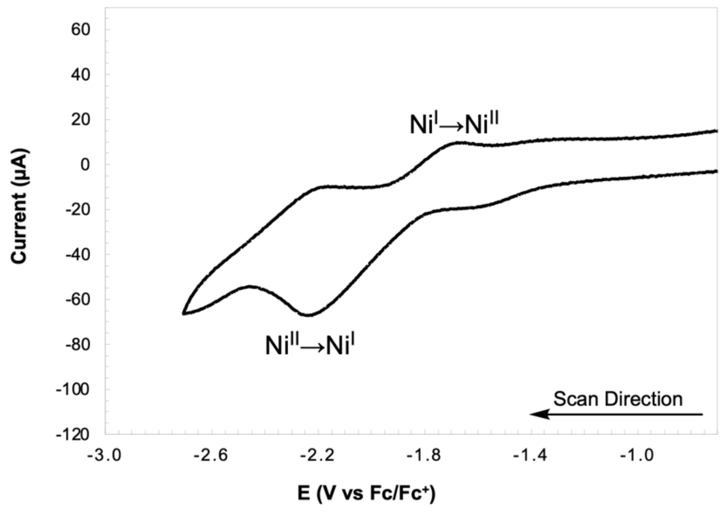

Figure S25. CV of [(4-NMe$_2$-^{iPr}pybox)Ni(Ph)]BAr$^F_4$ **22.** $E_{1/2}$ (Ni^{II}/Ni^{I}) = -1.96 V (vs Fc/Fc$^+$). Solvent = THF; temperature = 295 K; scan rate = 3030 mV/s; [Ni] = 1.0 mM; [TBAPF$_6$] = 100 mM.

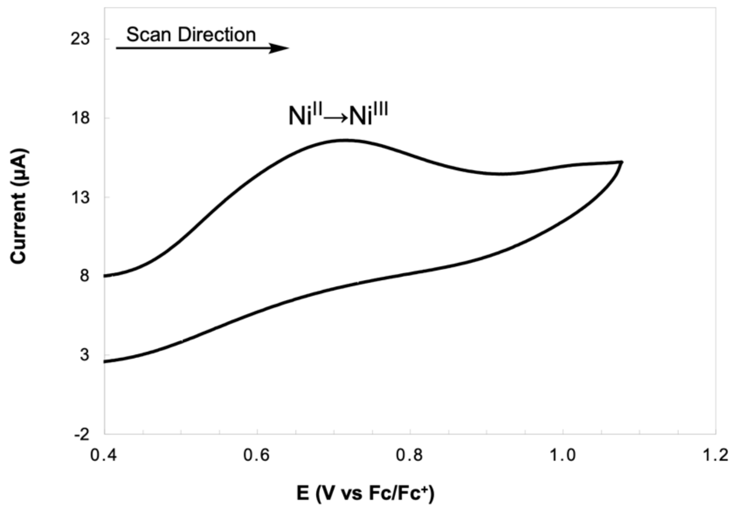

Figure S26. CV of [(4-NMe$_2$-^{iPr}pybox)Ni(Ph)]BAr$^F_4$ **22.** E_{pa} (Ni^{III}/Ni^{II}) = 0.72 V (vs Fc/Fc$^+$). Solvent = THF; temperature = 295 K; scan rate = 100 mV/s; [Ni] = 1.0 mM; [TBAPF$_6$] = 100 mM.

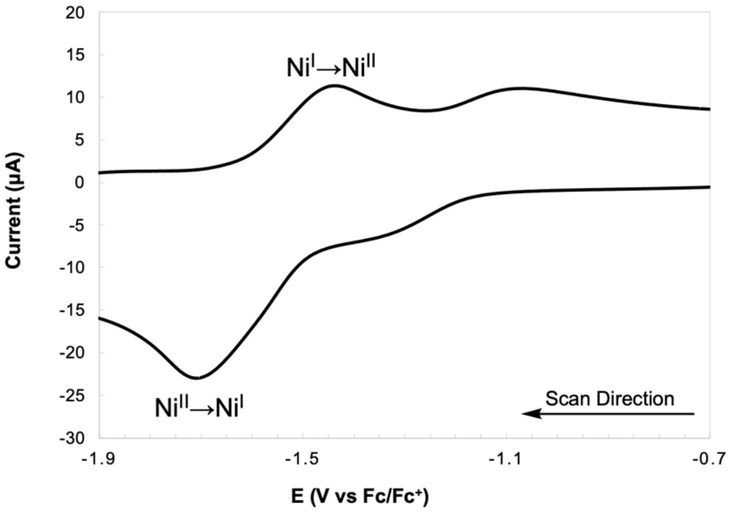

Figure S27. CV of [(4-OMe-$^{i\text{Pr}}$pybox)Ni(Ph)]BAr$^F_4$ **23.** $E_{1/2}$ (Ni^{II}/Ni^{I}) = -1.57 V (vs Fc/Fc^+). Solvent = THF; temperature = 295 K; scan rate = 500 mV/s; [Ni] = 1.0 mM; [$TBAPF_6$] = 100 mM.

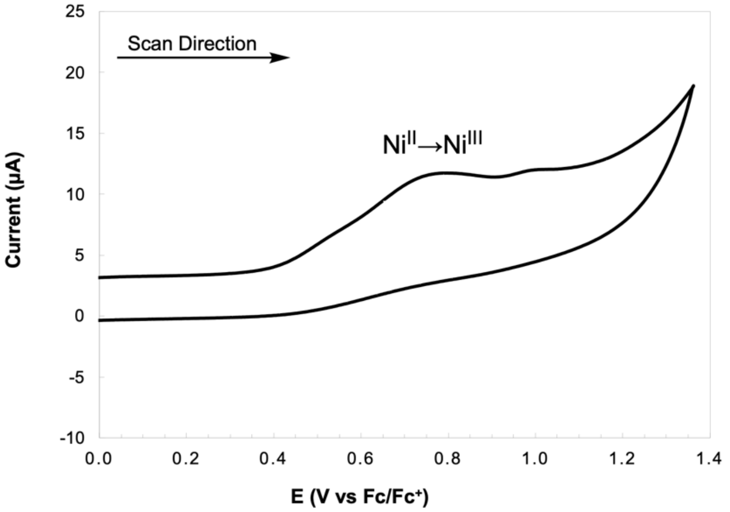

Figure S28. CV of [(4-OMe-$^{i\text{Pr}}$pybox)Ni(Ph)]BAr$^F_4$ **23.** E_{pa} (Ni^{III}/Ni^{II}) = 0.80 V (vs Fc/Fc^+). Solvent = THF; temperature = 295 K; scan rate = 100 mV/s; [Ni] = 1.0 mM; [$TBAPF_6$] = 100 mM.

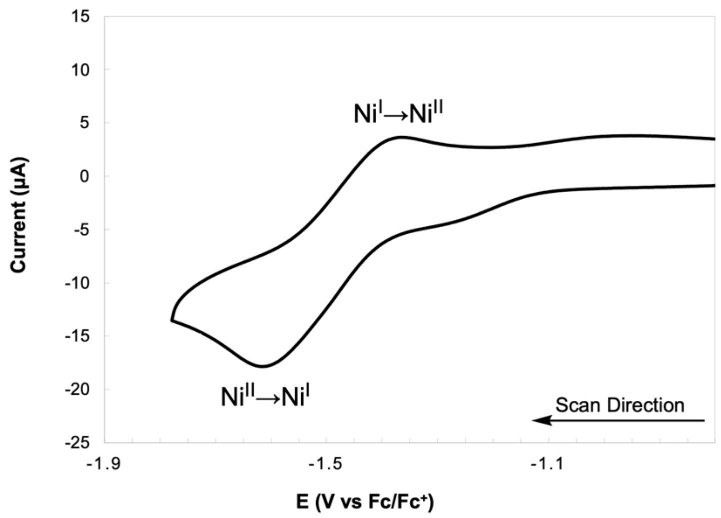

Figure S 29. CV of [(4-Me-^{iPr}pybox)Ni(Ph)]BAr$^F{}_4$ **24.** $E_{1/2}$ (Ni^{II}/Ni^{I}) = -1.49 V (vs Fc/Fc^+). Solvent = THF; temperature = 295 K; scan rate = 500 mV/s; [Ni] = 1.0 mM; [$TBAPF_6$] = 100 mM.

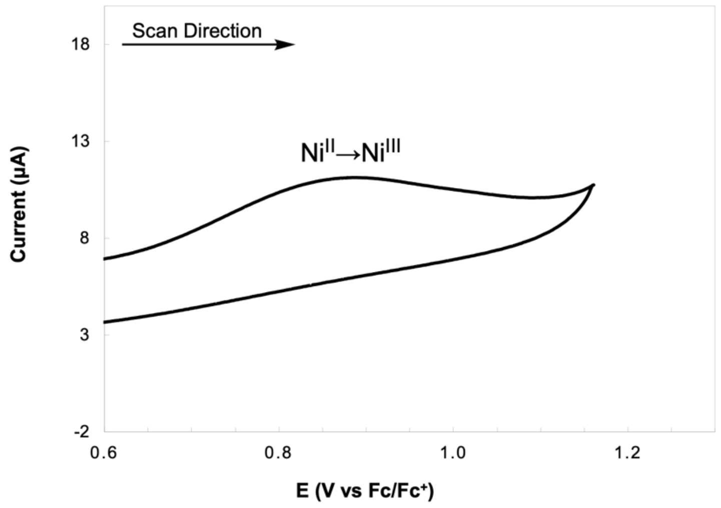

Figure S30. CV of [(4-Me-^{iPr}pybox)Ni(Ph)]BAr$^F{}_4$ **24.** E_{pa} (Ni^{III}/Ni^{II}) = 0.89 V (vs Fc/Fc^+). Solvent = THF; temperature = 295 K; scan rate = 100 mV/s; [Ni] = 1.0 mM; [$TBAPF_6$] = 100 mM.

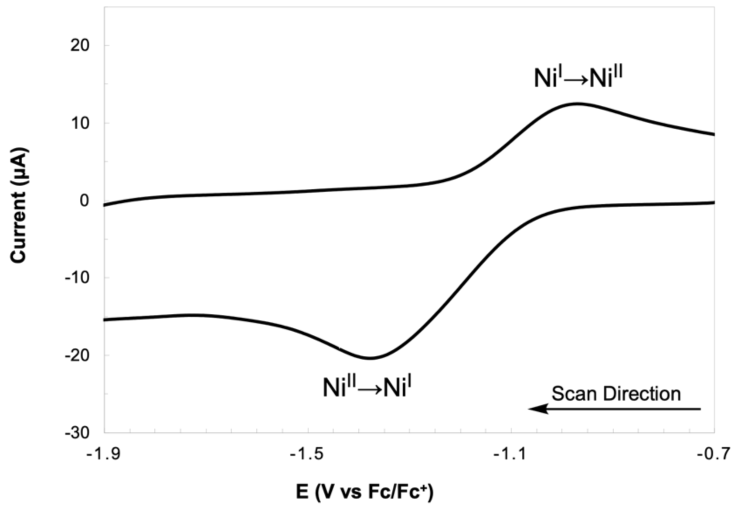

Figure S31. CV of [(4-CF_3-^{iPr}pybox)Ni(Ph)]BAr^F_4 **25.** $E_{1/2}$ (Ni^{II}/Ni^{I}) = -1.17 V (vs Fc/Fc^+). Solvent = THF; temperature = 295 K; scan rate = 500 mV/s; [Ni] = 1.0 mM; [$TBAPF_6$] = 100 mM.

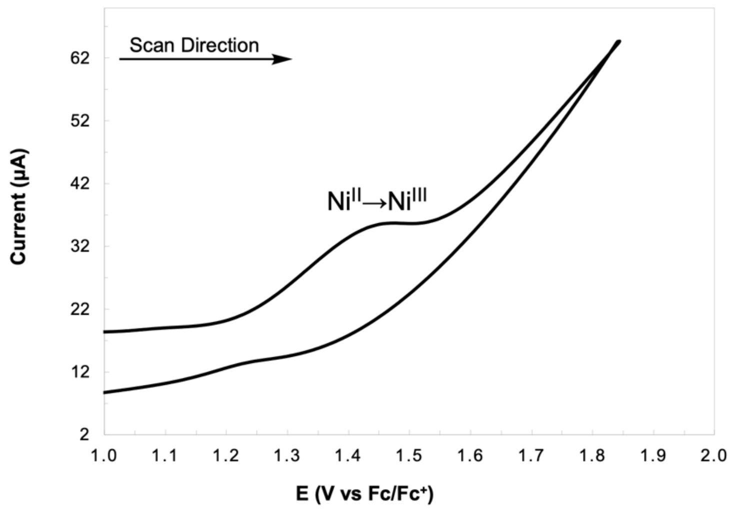

Figure S32. CV of [(4-CF_3-^{iPr}pybox)Ni(Ph)]BAr^F_4 **25.** E_{pa} (Ni^{III}/Ni^{II}) = 1.47 V (vs Fc/Fc^+). Solvent = THF; temperature = 295 K; scan rate = 100 mV/s; [Ni] = 1.0 mM; [$TBAPF_6$] = 100 mM.

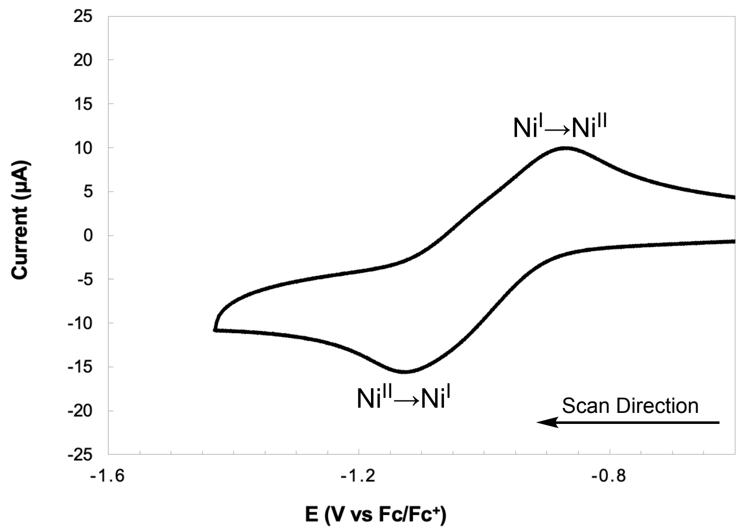

Figure S33. CV of [(4-CO_2Me-^{iPr}pybox)Ni(Ph)]BAr^F_4 **26.** $E_{1/2}$ (Ni^{II}/Ni^{I}) = -1.00 V (vs Fc/Fc^+). Solvent = THF; temperature = 295 K; scan rate = 500 mV/s; [Ni] = 1.0 mM; [$TBAPF_6$] = 100 mM.

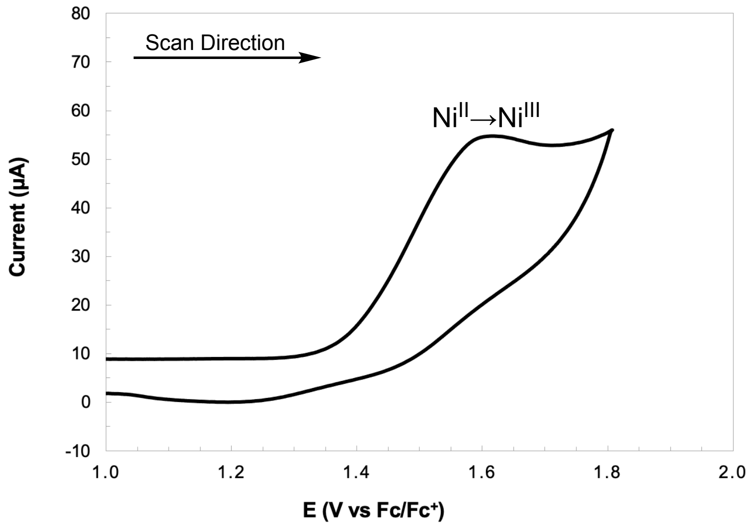

Figure S34. CV of [(4-CO_2Me-^{iPr}pybox)Ni(Ph)]BAr^F_4 **26.** E_{pa} (Ni^{III}/Ni^{II}) = 1.67 V (vs Fc/Fc^+). Solvent = DCM; temperature = 295 K; scan rate = 100 mV/s; [Ni] = 1.0 mM; [$TBAPF_6$] = 100 mM.

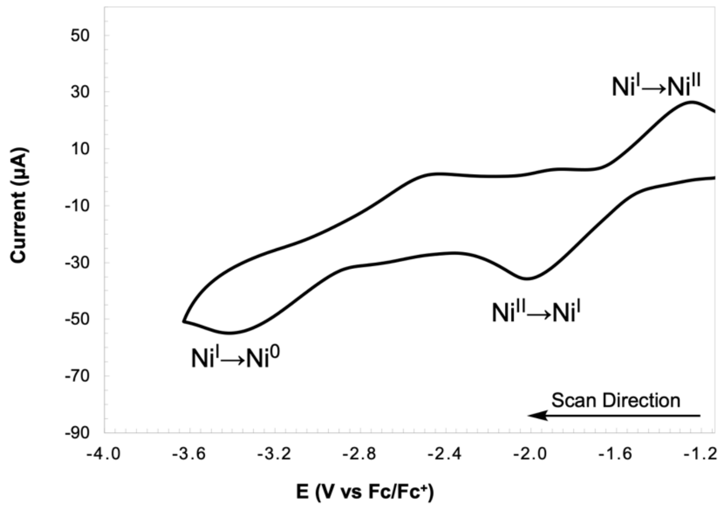

Figure S35. CV of [(^{iPr}pybox)Ni(*p*-MeO-C_6H_4)]BAr^F_4 **27.** $E_{1/2}$ (Ni^{II}/Ni^{I}) = -1.63 V (vs Fc/Fc^+). Solvent = THF; temperature = 295 K; scan rate = 500 mV/s; [Ni] = 1.0 mM; [$TBAPF_6$] = 100 mM.

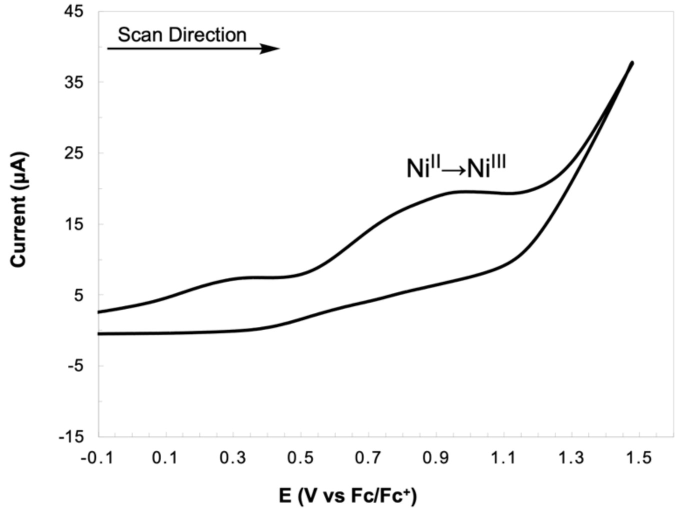

Figure S36. CV of [(^{iPr}pybox)Ni(*p*-MeO-C_6H_4)]BAr^F_4 **27.** E_{pa} (Ni^{III}/Ni^{II}) = 0.99 V (vs Fc/Fc^+). Solvent = THF; temperature = 295 K; scan rate = 100 mV/s; [Ni] = 1.0 mM; [$TBAPF_6$] = 100 mM.

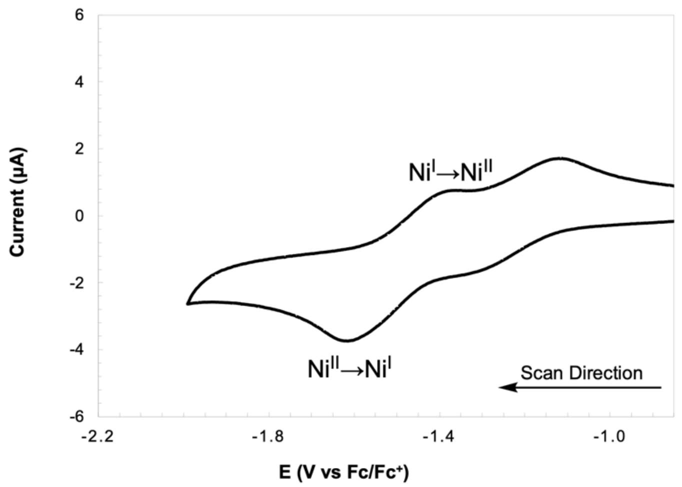

Figure S37. CV of [(^{iPr}pybox)Ni(p-tol)]BAr$^F_4$ **28.** $E_{1/2}$ (Ni^{II}/Ni^{I}) = -1.49 V (vs Fc/Fc$^+$). Solvent = THF; temperature = 295 K; scan rate = 100 mV/s; [Ni] = 1.0 mM; [TBAPF$_6$] = 100 mM.

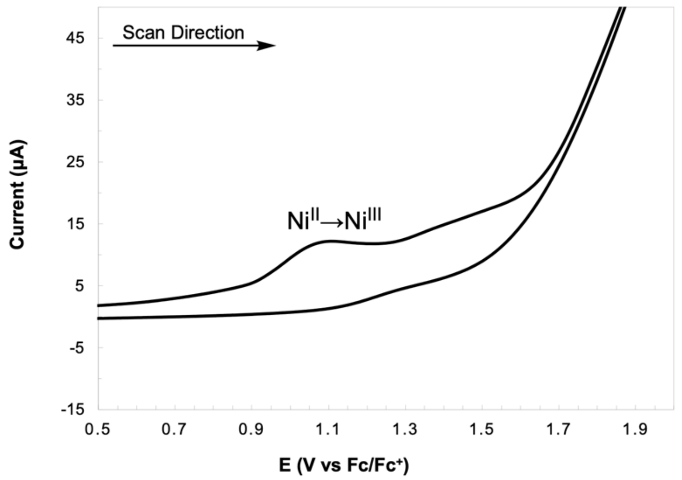

Figure S38. CV of [(^{iPr}pybox)Ni(p-tol)]BAr$^F_4$ **28.** E_{pa} (Ni^{III}/Ni^{II}) = 1.11 V (vs Fc/Fc$^+$). Solvent = THF; temperature = 295 K; scan rate = 100 mV/s; [Ni] = 1.0 mM; [TBAPF$_6$] = 100 mM.

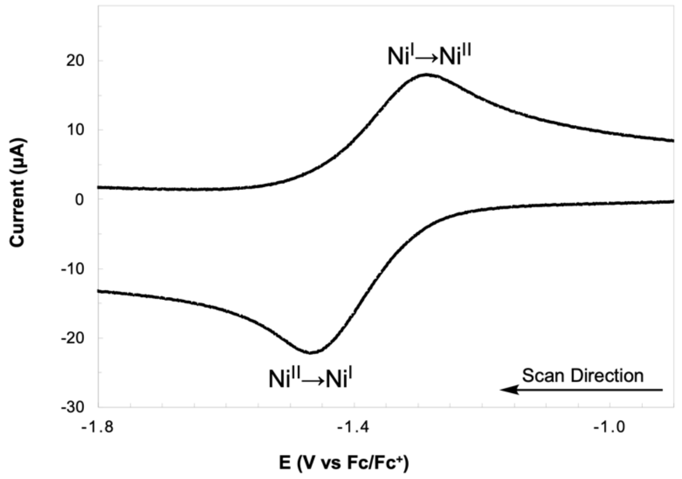

Figure S39. CV of [($^{i\text{Pr}}$pybox)Ni(*p*-CHO-C_6H_4)]$BAr^F{}_4$ **29.** $E_{1/2}$ (Ni^{II}/Ni^{I}) = -1.33 V (vs Fc/Fc^+). Solvent = THF; temperature = 295 K; scan rate = 500 mV/s; [Ni] = 1.0 mM; [$TBAPF_6$] = 100 mM.

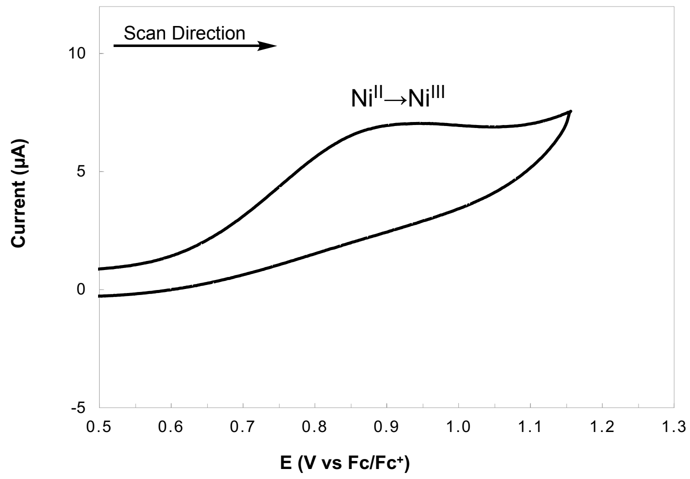

Figure S40. CV of [($^{i\text{Pr}}$pybox)Ni(*p*-CHO-C_6H_4)]$BAr^F{}_4$ **29.** E_{pa} (Ni^{III}/Ni^{II}) = 0.95 V (vs Fc/Fc^+). Solvent = THF; temperature = 295 K; scan rate = 100 mV/s; [Ni] = 1.0 mM; [$TBAPF_6$] = 100 mM.

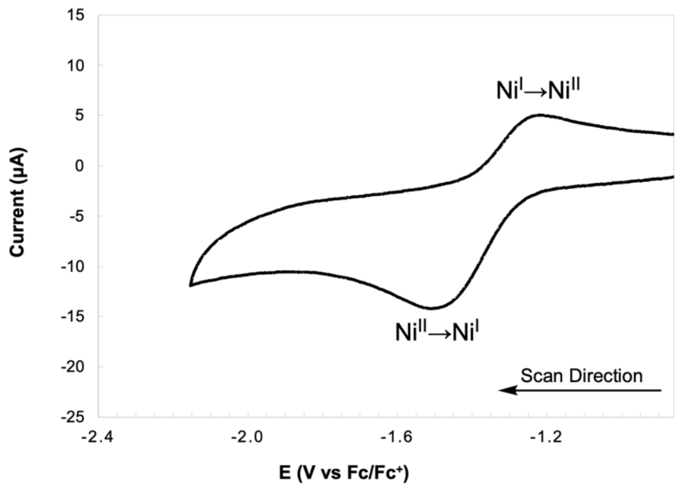

Figure S 41. CV of [(^{iPr}pybox)Ni(*p*-COMe-C_6H_4)]BAr$^F_4$ **30.** $E_{1/2}$ (Ni^{II}/Ni^{I}) = -1.36 V (vs Fc/Fc$^+$). Solvent = THF; temperature = 295 K; scan rate = 1000 mV/s; [Ni] = 1.0 mM; [$TBAPF_6$] = 100 mM.

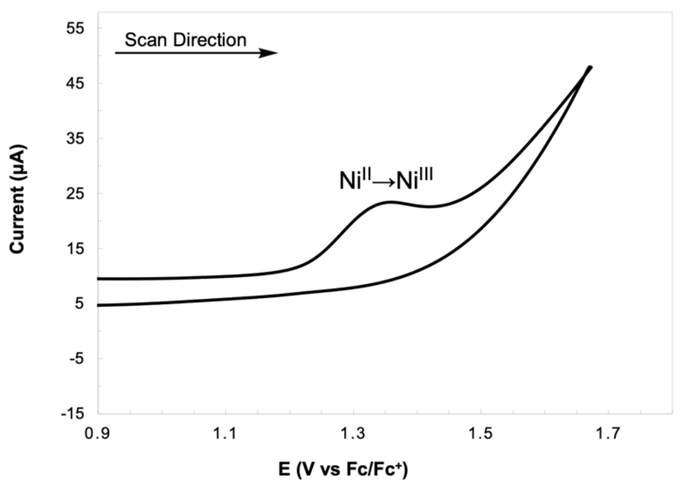

Figure S42. CV of [(^{iPr}pybox)Ni(*p*-COMe-C_6H_4)]BAr$^F_4$ **30.** E_{pa} (Ni^{III}/Ni^{II}) = 1.36 V (vs Fc/Fc$^+$). Solvent = THF; temperature = 295 K; scan rate = 100 mV/s; [Ni] = 1.0 mM; [$TBAPF_6$] = 100 mM.

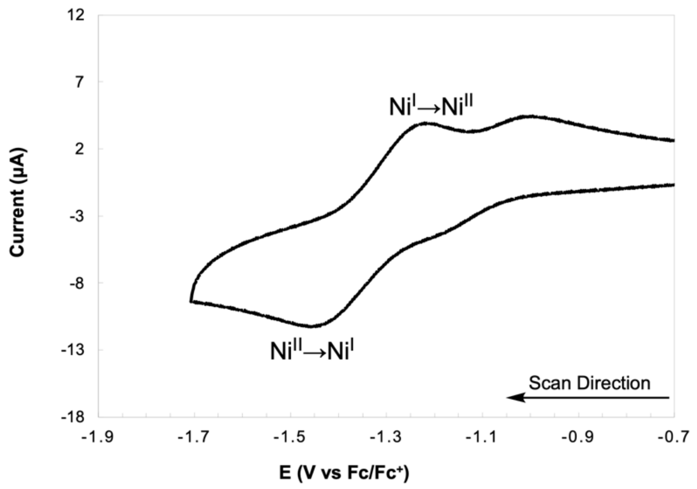

Figure S43. CV of [(^{iPr}pybox)Ni(p-CF$_3$-C$_6$H$_4$)]BAr$^{F}{}_4$ **31.** $E_{1/2}$ (Ni^{II}/Ni^{I}) = -1.35 V (vs Fc/Fc$^+$). Solvent = THF; temperature = 295 K; scan rate = 1000 mV/s; [Ni] = 1.0 mM; [TBAPF$_6$] = 100 mM.

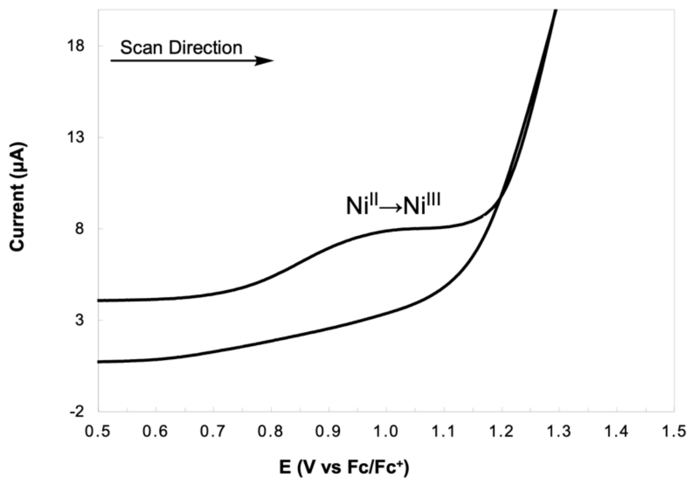

Figure S44. CV of [(^{iPr}pybox)Ni(p-CF$_3$-C$_6$H$_4$)]BAr$^{F}{}_4$ **31.** E_{pa} (Ni^{III}/Ni^{II}) = 1.08 V (vs Fc/Fc$^+$). Solvent = THF; temperature = 295 K; scan rate = 100 mV/s; [Ni] = 1.0 mM; [TBAPF$_6$] = 100 mM.

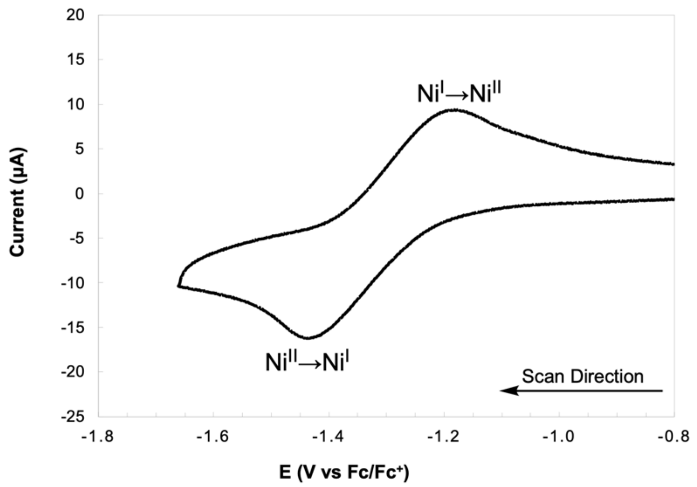

Figure S45. CV of [(^{iPr}pybox)Ni(p-CN-C_6H_4)]BAr^F_4 **32.** $E_{1/2}$ (Ni^{II}/Ni^{I}) = -1.32 V (vs Fc/Fc^+). Solvent = THF; temperature = 295 K; scan rate = 250 mV/s; [Ni] = 1.0 mM; [$TBAPF_6$] = 100 mM.

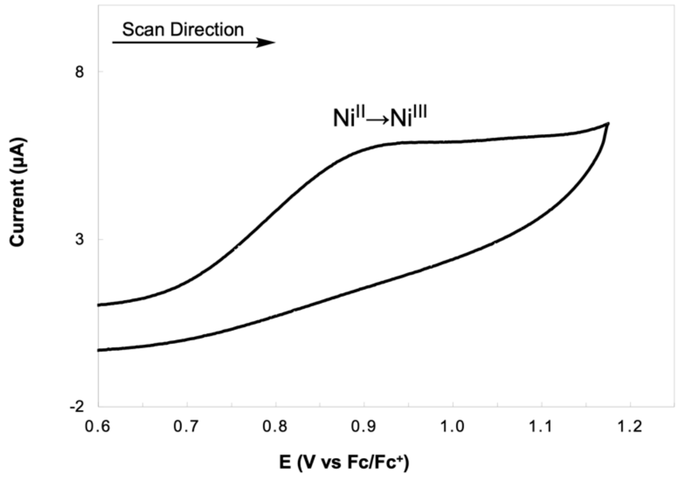

Figure S46. CV of [(^{iPr}pybox)Ni(p-CN-C_6H_4)]BAr^F_4 **32.** E_{pa} (Ni^{III}/Ni^{II}) = 0.87 V (vs Fc/Fc^+). Solvent = THF; temperature = 295 K; scan rate = 100 mV/s; [Ni] = 1.0 mM; [$TBAPF_6$] = 100 mM.

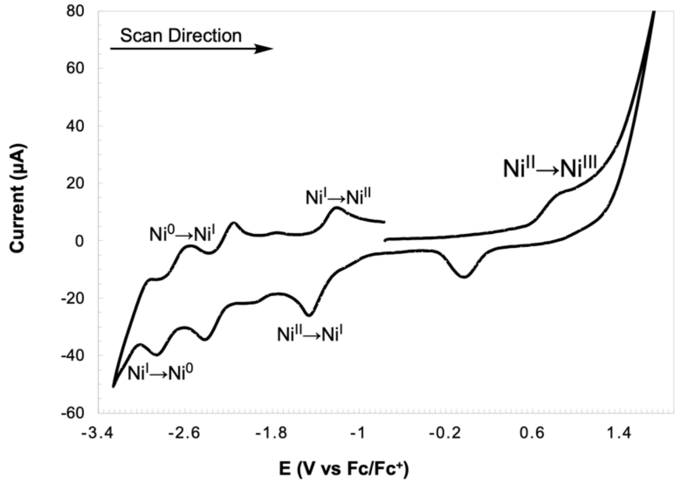

Figure S47. Full solvent window CV of [(^{iPr}pybox)Ni(*p*-CN-C_6H_4)]BAr^F_4 **32** showing additional redox features**.** Solvent = THF; temperature = 295 K; scan rate = 500 mV/s; [Ni] = 1.0 mM; [$TBAPF_6$] = 100 mM.

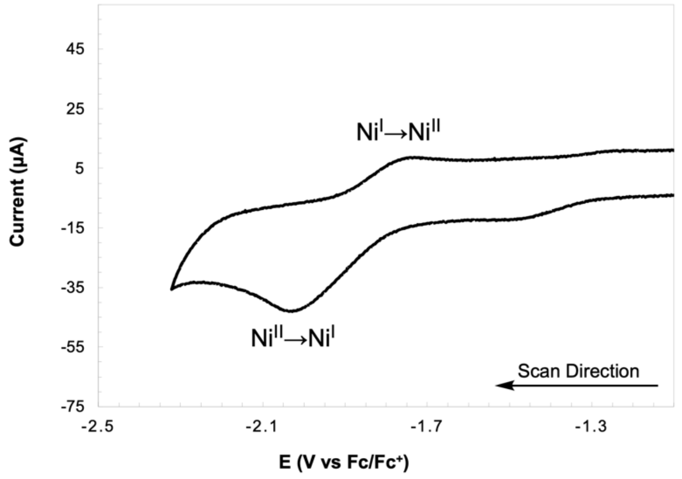

Figure S48. CV of [(4-Pyrrolidinyl-^{iPr}pybox)Ni(*p*-MeO-C_6H_4)]BAr^F_4 **33.** $E_{1/2}$ (Ni^{II}/Ni^{I}) = -1.87 V (vs Fc/Fc^+). Solvent = THF; temperature = 295 K; scan rate =3030 mV/s; [Ni] = 1.0 mM; [$TBAPF_6$] = 100 mM.

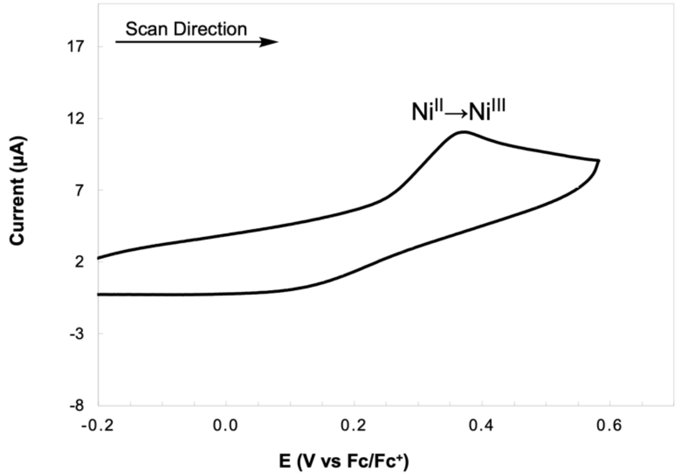

Figure S49. CV of [(4-Pyrrolidinyl-$^{i\text{Pr}}$pybox)Ni(p-MeO-C_6H_4)]BAr^F_4 **33.** E_{pa} (Ni^{III}/Ni^{II}) = 0.37 V (vs Fc/Fc^+). Solvent = THF; temperature = 295 K; scan rate = 100 mV/s; [Ni] = 1.0 mM; [$TBAPF_6$] = 100 mM.

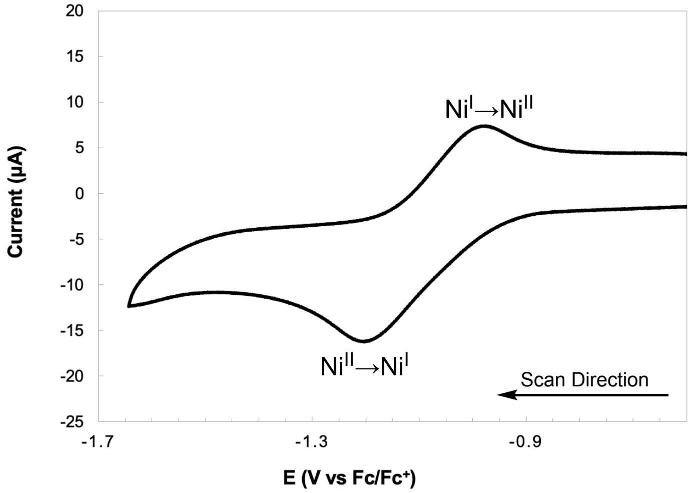

Figure S50. CV of [(4-CF_3-$^{i\text{Pr}}$pybox)Ni(p-tol)]BAr^F_4 **34.** $E_{1/2}$ (Ni^{II}/Ni^{I}) = -1.09 V (vs Fc/Fc^+). Solvent = THF; temperature = 295 K; scan rate =500 mV/s; [Ni] = 1.0 mM; [$TBAPF_6$] = 100 mM.

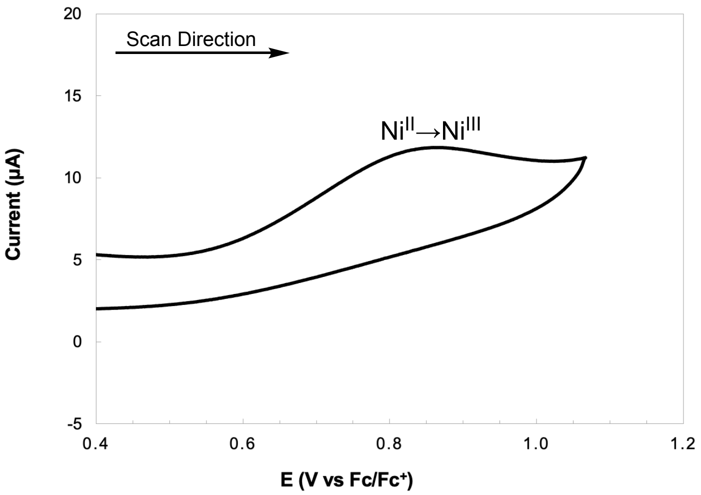

Figure S 51. CV of [(4-CF_3-$^{i\text{Pr}}$pybox)Ni(*p*-tol)]BAr^F_4 **34.** E_{pa} (Ni^{III}/Ni^{II}) = 0.86 V (vs Fc/Fc^+). Solvent = THF; temperature = 295 K; scan rate = 100 mV/s; [Ni] = 1.0 mM; [$TBAPF_6$] = 100 mM.

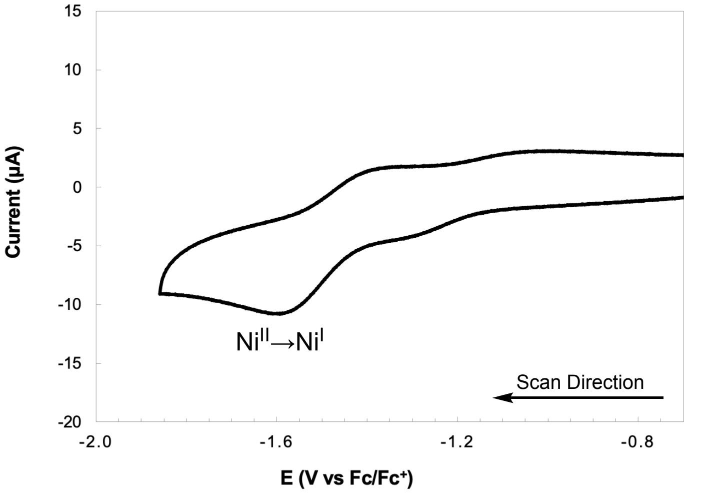

Figure S52. CV of [(4-OMe-$^{i\text{Pr}}$pybox)Ni(*p*-CF_3-C_6H_4)]BAr^F_4 **35.** $E_{p/2}$ (Ni^{II}/Ni^{I}) = -1.42 V (vs Fc/Fc^+). Solvent = THF; temperature = 295 K; scan rate =500 mV/s; [Ni] = 1.0 mM; [$TBAPF_6$] = 100 mM.

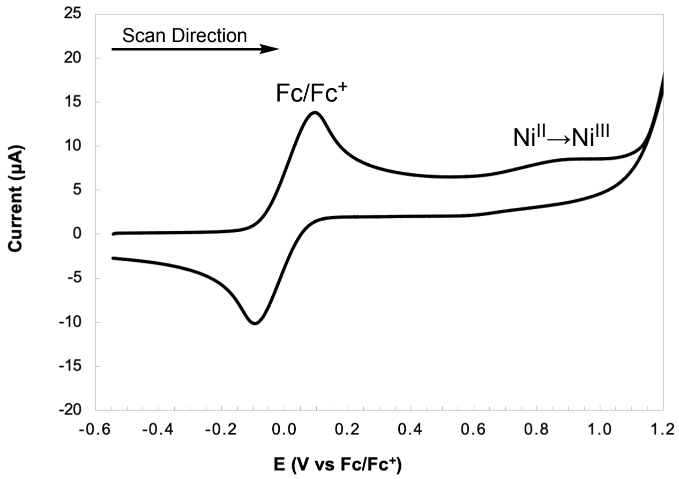

Figure S 53. CV of [(4-OMe-^{iPr}pybox)Ni(p-CF_3-C_6H_4)]BAr^F_4 **35.** E_{pa} (Ni^{III}/Ni^{II}) = 0.95 V (vs Fc/Fc^+). Solvent = THF; temperature = 295 K; scan rate = 100 mV/s; [Ni] = 1.0 mM; [$TBAPF_6$] = 100 mM.

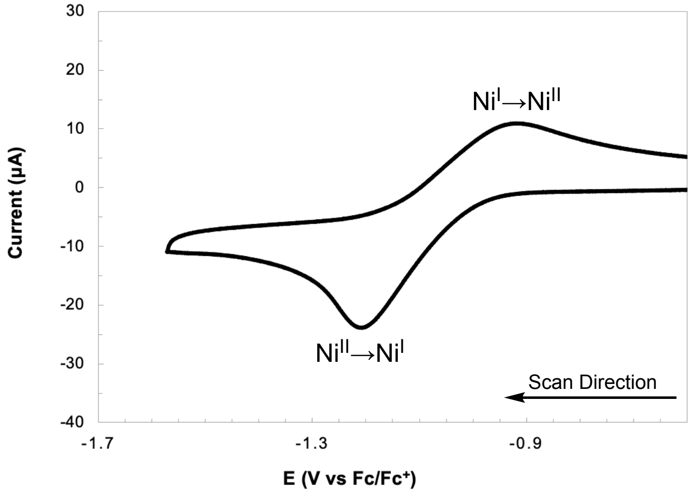

Figure S54. CV of [(4-CF_3-^{iPr}pybox)Ni(p-CF_3-C_6H_4)]BAr^F_4 **36.** $E_{1/2}$ (Ni^{II}/Ni^{I}) = -1.06 V (vs Fc/Fc^+). Solvent = THF; temperature = 295 K; scan rate =500 mV/s; [Ni] = 1.0 mM; [$TBAPF_6$] = 100 mM.

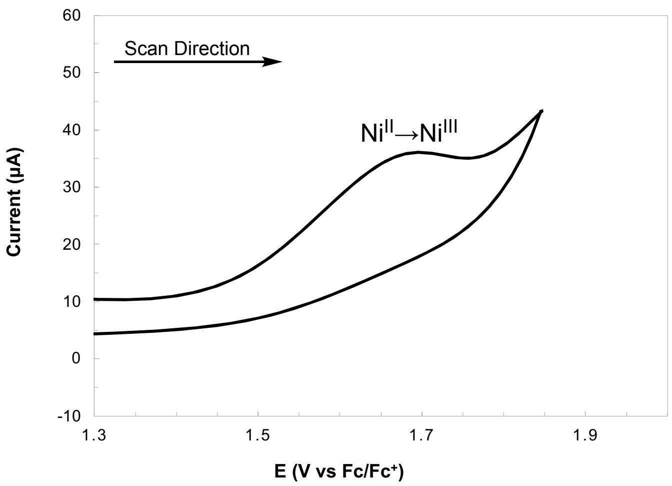

Figure S 55. CV of [(4-CF_3-^{iPr}pybox)Ni(*p*-CF_3-C_6H_4)]BAr^F_4 **36.** E_{pa} (Ni^{III}/Ni^{II}) = 1.70 V (vs Fc/Fc^+). Solvent = DCM; temperature = 295 K; scan rate = 100 mV/s; [Ni] = 1.0 mM; [$TBAPF_6$] = 100 mM.

9. Tabulated Hammett Parameters and Relative Rate Data

Table S2. Tabulated data for Hammett parameters for each actor and supporting ligand substituent

		Actor Ligand				Supporting Ligand			
Complex	$\log(k_X/k_H)$	σ_p	σ_p^+	σ_R	$\sigma^{\bullet}_{JJ}$	σ_p	σ_p^+	σ_R	$\sigma^{\bullet}_{JJ}$
**6**	0.00	0.00	0.00	0.00	0.00	0.00	0.00	0.00	0.00
**21**	-0.20	0.00	0.00	0.00	0.00	-0.72[c]	-2.07[c]	-0.56[d]	1.00[d]
**22**	-0.29	0.00	0.00	0.00	0.00	-0.83	-1.7	-0.56	1.00
**23**	0.05	0.00	0.00	0.00	0.00	-0.27	-0.78	-0.43	0.23
**24**	-0.05	0.00	0.00	0.00	0.00	-0.17	-0.31	-0.13	0.15
**25**	-0.06	0.00	0.00	0.00	0.00	0.54	0.61	0.09	-0.01
**26**	-0.17	0.00	0.00	0.00	0.00	0.45	0.49	0.16[e]	0.33
**27**	-0.53	-0.27	-0.78	-0.43	0.23	0.00	0.00	0.00	0.00
**28**	-0.11	-0.17	-0.31	-0.13	0.15	0.00	0.00	0.00	0.00
**29**	-0.28	0.42	0.73	0.23	0.54[b]	0.00	0.00	0.00	0.00
**30**	-0.53	0.5	0.73[a]	0.16	0.54	0.00	0.00	0.00	0.00
**31**	-0.48	0.54	0.61	0.09	-0.01	0.00	0.00	0.00	0.00
**32**	0.04	0.66	0.66	0.18	0.42	0.00	0.00	0.00	0.00
**33**	-0.02	-0.27	-0.78	-0.43	0.23	-0.72[c]	-2.07[c]	0.56[d]	1.00[d]
**34**	0.28	-0.17	-0.31	-0.13	0.15	0.54	0.61	0.09	-0.01
**35**	-0.46	0.54	0.61	0.09	-0.01	-0.27	-0.78	-0.43	0.23

Hammett parameters including σ[17], σ_R[17], σ_p^+[17], and $\sigma^{\bullet}_{JJ}$[18] were utilized in fitting the data. [a]No value reported, value for *p*-CHO used as a substitute. [b]No value reported, value for *p*-COMe used as a substitute. [c]No value reported, value for *p*-NEt_2 used as a substitute. [d]No value reported, value for *p*-NMe_2 used as a substitute. [e]No value reported, value for CO_2Et used as substitute.

10. Modeling Using Hammett Parameters

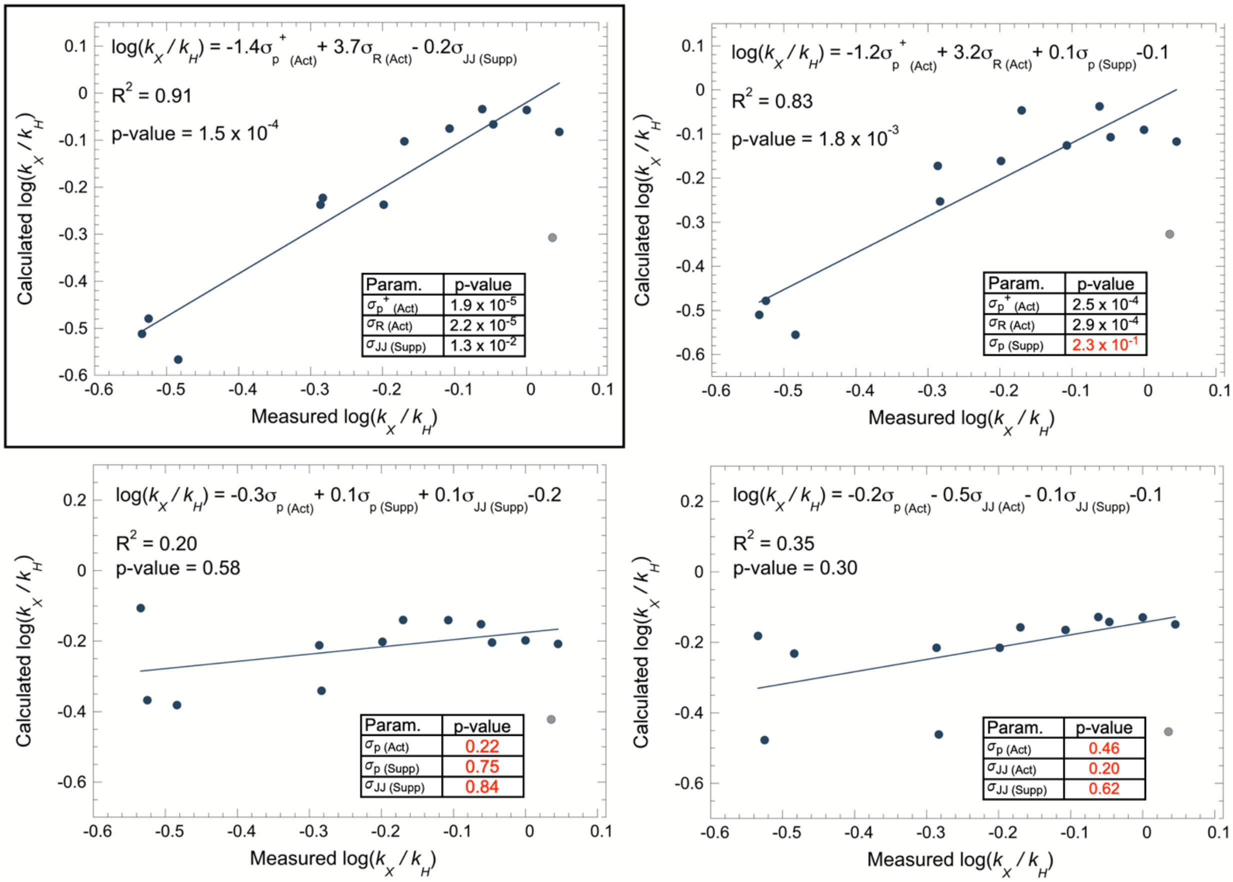

Figure S56. Alternative fits involving multiple Hammett parameters, with complex **32** with a grey marker. The p-value of the overall fit is displayed in the top left corner of each plot, while individual p-values of each parameter is shown in the bottom right table. P-values greater than 0.05 are considered statistically insignificant and are highlighted in red.

11. Computational Benchmarking and Workflow

Chart S1. Workflow for generation and benchmarking of optimized geometries, calculation of computational descriptors, initial regression modeling with computational descriptors, and expanded calculations with experimental and computational regression modeling.

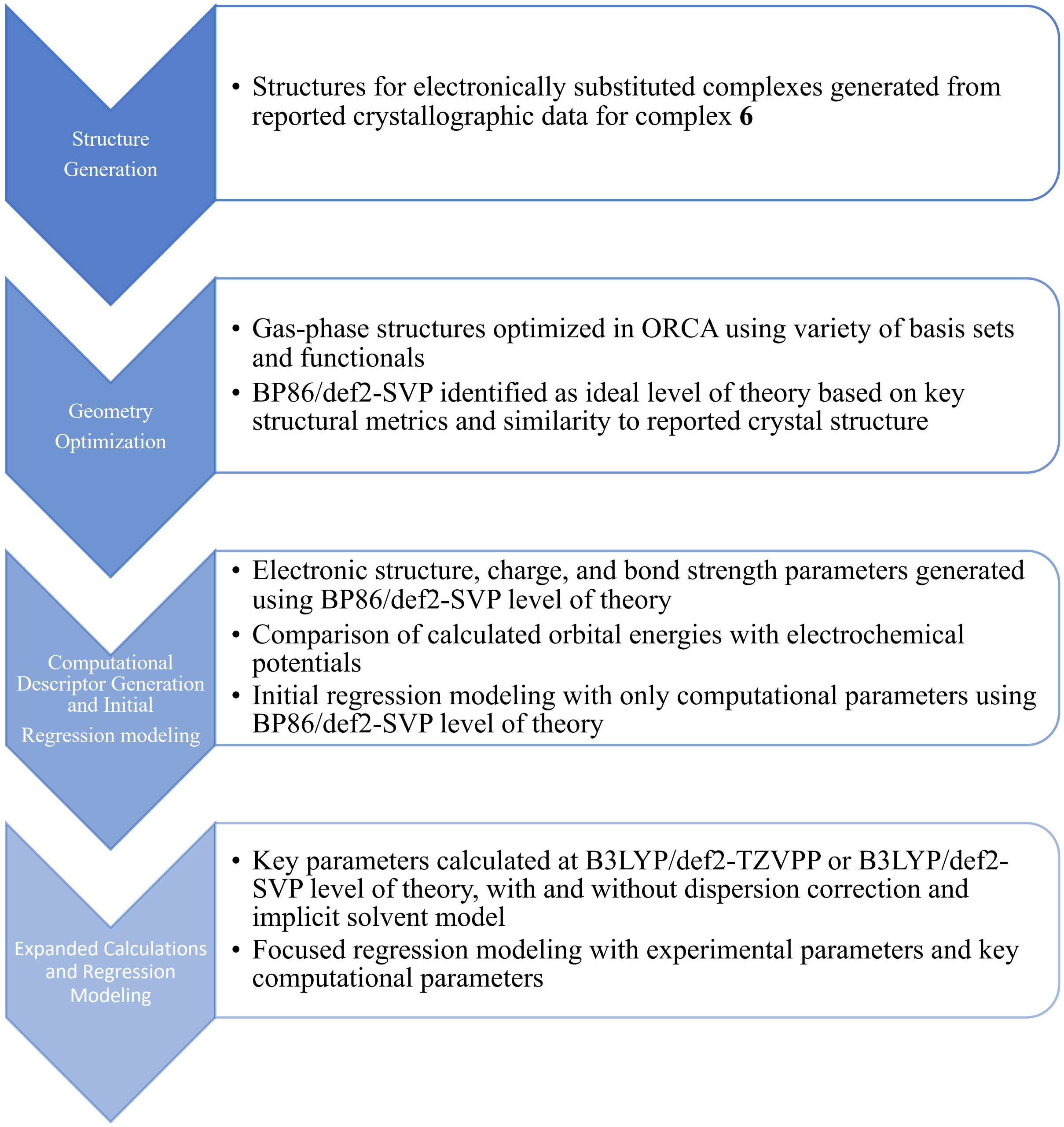

Density Functional Theory (DFT) calculations run using ORCA 4.2.1 [19] on NYU GREENE supercomputers. Calculations were carried out on the electronically varied nickel(II) intermediates synthesized in the study. To simplify calculations, reduce computational cost and obtain properties pertaining solely to the nickel center and directly-bound ligands, each nickel(II) complex was treated as a closed-shell cation, with the BAr^F_4 counter anion excluded from the structure. To determine the ideal level of theory, a variety of basis sets and functionals were used to optimize the gas-phase structure of $[(^{i\text{Pr}}\text{pybox})\text{Ni(Ph)}]^+$, with the reported crystal structure of $[(^{i\text{Pr}}\text{pybox})\text{Ni(Ph)}]BAr^F_4$ **6**[15] as a benchmark for calculations. The following bond and dihedral angles were used alongside bond lengths to evaluate optimized structures:

α = C2—C1—Ni bond angle

β = H—C2—C1—Ni dihedral angle

γ = N2—Ni—C1—C2 dihedral angle

Scheme S42. Select bond and dihedral angles evaluated in computational benchmarking.

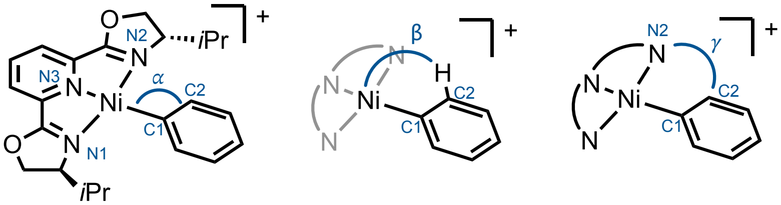

A series of functionals including B3LYP[20,21], ωB97X-D3[22], BP86[23] and B97-D[21], as well as basis sets including m6-31g*[24], def2-SVP[25], def2-TZVPP[25] were screened for the geometry optimization. D3 dispersion correction[26] was also used for select functionals. All structures were generally reasonable, with no major geometric distortions from square planarity. We found BP86/def2-SVP to give the best agreement with reported crystallographic data (Table S2).

Scheme S43. Labeled atoms in computed structure of complex **31**.

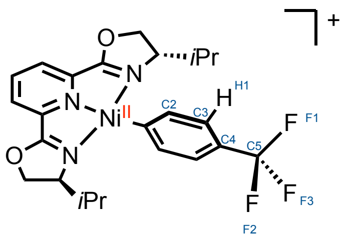

Initial optimization of [(^{iPr}pybox)Ni(p-CF_3-C_6H_4)]$^+$ **31** with BP86/def2-SVP showed distortion of the CF_3 substituent (C5) out of planarity of the aryl ring and an eclipsed conformation of F1 relative to C3. To adjust for the distortion in the initial optimized structure of **31**, the dihedral angle between C5 and H1 was constrained to 0.4° and the dihedral angle between F1 and C3 was constrained to 85.8°. Both values were chosen based on the dihedral angles observed in the BAr^F_4 anion in the crystal structure of **6**. Only a slight increase in the calculated energy of the constrained structure compared to the initial distorted structure was observed (+0.006 kcal/ mol), and so the constrained structure was used for later calculations.

Table S3. Comparison of optimized geometry of [(^{iPr}pybox)Ni(Ph)]$^+$ **6** using various levels of theory and reported crystal structure

Method	Ni—C1 (Å)	Average Ni—N1 and Ni—N2 (Å)	Ni—N3 (Å)	α (°)	β (°)	γ (°)
Crystal Structure[15]	1.881(3)	1.894	1.898	120.6	1.1	69.2
B3LYP/m6-31g*	1.971	2.015	2.019	122	1.2	91.3
B3LYP-D3/def2-TZVPP	1.895	1.989	1.991	118.8	1.8	60.7
ωB97X-D3/m6-31g*	1.959	1.997	2.012	121.7	1.6	90.5
BP86/m6-31g*	1.961	1.975	1.981	122.1	2.1	90.1
B97-D/def2-SVP	1.876	1.910	1.930	120	0.5	58.5
**BP86/def2-SVP**	**1.881**	**1.904**	**1.912**	**120.3**	**0.8**	**65.6**

Table S4. Comparison of optimized geometries of electronically substituted complexes using BP86/def2-SVP and reported crystal structure

Complex	Ni—C1 (Å)	Average Ni—N1 and Ni—N2 (Å)	Ni—N3 (Å)	α (°)	β (°)	γ (°)
Crystal[15]	1.881(3)	1.894	1.898	120.6	1.1	69.2
**6**	1.881	1.904	1.912	120.3	0.8	65.6
**21**	1.880	1.906	1.909	120.9	0.8	66.1
**22**	1.880	1.906	1.909	121.0	0.9	65.9
**23**	1.879	1.905	1.912	120.3	0.4	66.4
**24**	1.880	1.907	1.910	120.5	0.9	66.2
**25**	1.881	1.907	1.910	120.3	0.8	65.3
**26**	1.882	1.907	1.907	120.2	0.1	65.3
**27**	1.880	1.906	1.909	120.5	1.2	61.1
**28**	1.881	1.904	1.912	120.6	0.7	63.4
**29**	1.877	1.905	1.912	120.8	0.4	67.1
**30**	1.878	1.906	1.912	120.4	0.7	66.1
**31**	1.880	1.904	1.911	120.6	0.8	66.6
**32**	1.879	1.906	1.910	120.6	0.6	67.4

12. Description of Computational Parameters

All calculations were based on geometries optimized using BP86/def2-SVP level of theory, which was found to produce the best match with reported crystallographic data. Computational parameters were then calculated using BP86/def2-SVP.

After initial screening of several computational descriptors, select parameters were also calculated using B3LYP-D3/def2-TZVPP level of theory. For select parameters, a conductor-like polarizable continuum model (CPCM) with THF was included. Due to the introduction of artifacts when using the Mulliken charge model with large basis sets, Mulliken charges were calculated using B3LYP-D3/def2-SVP as well. In the context of Minimum Basis Set (MBS) Mulliken charges, using a smaller basis set has been shown to significantly improve the reliability of the Mulliken charge model and show good agreement with experimental Hammett values. [27, 28]

To decouple effects stemming from the supporting ligand and aryl ligand, calculations were carried out on the supporting ligand and the nickel–aryl fragment, as well as the full nickel(II) complex. Geometries

used for calculations on the supporting ligand were taken from the "frozen" optimized geometry of the full complex. Geometries used for calculations on the nickel–aryl fragment were taken from the "frozen" optimized geometry of the full complex, and the nickel–aryl fragment was treated as a closed-shell cation.

LUMO: Energy of the Lowest Unoccupied Molecular Orbital (LUMO) of the full nickel(II) complex in hartrees.

$LUMO_{Pybox}$: Energy of the LUMO calculated on the isolated supporting ligand, using the frozen geometry from optimization of the full complex in hartrees.

HOMO: Energy of the Highest Occupied Molecular Orbital (HOMO) of the full nickel(II) complex in hartrees.

$HOMO_{Ni}$: The highest occupied molecular orbital localized on the nickel center, in the full nickel(II) complex in hartrees.

$Dipole_{Ni\text{–}Ar}$: Dipole moment of the isolated nickel–aryl fragment in Debye, using the frozen geometry from optimization of the full complex.

$HOMO_{Pybox}$: Energy of the HOMO calculated on the isolated supporting ligand, using the frozen geometry from optimization of the full complex in hartrees.

$\%Ni_{HOMO}$: Percentage of nickel character for the HOMO of the full nickel(II) complex.

BO: Mayer bond order of the Ni—C1 bond in the full nickel(II) complex.

$Mull_{Ni}$, $Mull_{C1}$: Mulliken charge on the nickel center or C1 of the aryl group in the full nickel(II) complex.

$Loew_{Ni}$, $Loew_{C1}$: Löwdin charge on the nickel center or C1 of the aryl group in the full nickel(II) complex.

NBO_{Ni}, NBO_{C1}: Charge on the nickel center or C1 of the aryl group in the full nickel(II) complex derived from Natural Bond Orbital (NBO) analysis.[29]

$Hirsh_{Ni}$, $Hirsh_{C1}$: Hirshfeld charge on the nickel center or C1 of the aryl group in the full nickel(II) complex.

Dipole: Dipole moment of the full nickel(II) complex in Debye.

NBO_{dz2}: Energy of the nickel-centered dz^2 orbital determined through NBO analysis.

13. Tabulated Data on Computational Parameters

Table S5. Calculated molecular orbital and bond order descriptors using BP86/def2-SVP level of theory

Complex	*LUMO*	$LUMO_{Pybox}$	*HOMO*	$HOMO_{Ni}$	$HOMO_{Pybox}$	$\%Ni_{HOMO}$	*BO*
**6**	-0.24452	-0.08232	-0.29652	-0.29652	-0.17305	89.4	0.9110
**21**	-0.21397	-0.06732	-0.27498	-0.27498	-0.15695	92.3	0.9141
**22**	-0.21696	-0.06861	-0.27746	-0.27746	-0.15831	92.3	0.9136
**23**	-0.23021	-0.07731	-0.28735	-0.28735	-0.16704	91.4	0.9130
**24**	-0.23772	-0.07924	-0.29166	-0.29166	-0.17019	90.6	0.9111
**25**	-0.25436	-0.09702	-0.30383	-0.30383	-0.18314	89.1	0.910
**27**	-0.24051	-0.08232	-0.27398	-0.29356	-0.17305	10.4	0.9187
**28**	-0.24274	-0.08232	-0.29153	-0.2948	-0.17305	22.5	0.9129
**29**	-0.25088	-0.08232	-0.28461	-0.30412	-0.17305	0.3	0.9152
**30**	-0.24844	-0.08232	-0.27809	-0.30143	-0.17305	0.3	0.9158
**31**	-0.25047	-0.08232	-0.30353	-0.30353	-0.17305	88.6	0.9107
**32**	-0.25368	-0.08232	-0.30754	-0.30754	-0.17305	88.9	0.9139

Table S6. Calculated charge and NBO descriptors using BP86/def2-SVP level of theory

Complex	$Mull_{Ni}$	$Mull_{C1}$	$Loew_{Ni}$	$Loew_{C1}$	NBO_{Ni}	NBO_{C1}	*Dipole*	NBO_{dz2}
**6**	0.365786	-0.098620	0.132196	-0.106265	0.94496	-0.27255	4.25536	-0.32705
**21**	0.356168	-0.108790	0.116603	-0.109851	0.93399	-0.28119	6.82884	-0.30474
**22**	0.357351	-0.106629	0.117993	-0.10953	0.93506	-0.28057	7.60057	-0.30733
**23**	0.362977	-0.108886	0.125074	-0.108064	0.93857	-0.27535	5.25209	-0.31749
**24**	0.364893	-0.106371	0.129442	-0.10749	0.94249	-0.27476	4.86594	-0.32226
**25**	0.373426	-0.106482	0.138896	-0.106261	0.95229	-0.27099	2.42643	-0.33484
**27**	0.367139	-0.090274	0.130627	-0.129161	0.94974	-0.30362	4.60448	-0.32402
**28**	0.366876	-0.096193	0.131786	-0.113209	0.94652	-0.28077	4.36424	-0.32532
**29**	0.369474	-0.092618	0.132917	-0.092882	0.94456	-0.25476	9.47139	-0.33546
**30**	0.369704	-0.095427	0.133231	-0.096673	0.94521	-0.25856	9.23897	-0.33251
**31**	0.369052	-0.086089	0.132588	-0.099811	0.94433	-0.26575	11.49229	-0.3346
**32**	0.370981	-0.095410	0.133466	-0.098489	0.94484	-0.26327	11.09717	-0.33908

Table S7. Calculated Hirshfeld charge descriptors using BP86/def2-SVP level of theory

Complex	$Hirsh_{Ni}$	$Hirsh_{C1}$
**6**	0.049291	-0.072068
**21**	0.037363	-0.075751
**22**	0.040705	-0.075397
**23**	0.043865	-0.073742
**24**	0.047141	-0.072756
**25**	0.054605	-0.070967
**27**	0.048249	-0.085657
**28**	0.049571	-0.076556
**29**	0.05106	-0.063015
**30**	0.050548	-0.065721
**31**	0.050817	-0.067491
**32**	0.051186	-0.065106

Table S8. Additional calculated descriptors using B3LYP-D3/def2-TZVPP level of theory

Complex	*HOMO*	*LUMO*	$Mull_{Ni}$	$Mull_{C1}$	$Loew_{Ni}$	$Loew_{C1}$
**6**	-0.32211	-0.22304	0.225133	-0.043688	-0.186836	-0.090105
**21**	-0.30805	-0.19249	0.21433	-0.068697	-0.196056	-0.093862
**22**	-0.31007	-0.19598	0.216096	-0.060807	-0.195110	-0.093635
**23**	-0.31683	-0.20935	0.222718	-0.069693	-0.190870	-0.092274
**24**	-0.31897	-0.21604	0.224722	-0.062181	-0.187793	-0.091393
**25**	-0.32744	-0.2351	0.232517	-0.062405	-0.182010	-0.089850

**27**	-0.29321	-0.22072	0.219016	-0.106216	-0.188235	-0.105735
**28**	-0.31091	-0.22162	0.22249	-0.078059	-0.187270	-0.095184
**29**	-0.32914	-0.22929	0.228758	-0.054516	-0.183427	-0.076171
**30**	-0.32207	-0.22708	0.227504	-0.066056	-0.183029	-0.080330
**31**	-0.33745	-0.22926	0.224327	-0.045158	-0.183243	-0.081865
**32**	-0.33377	-0.23227	0.23304	-0.104667	-0.180746	-0.037149

Table S9. Additional calculated descriptors using B3LYP-D3/def2-TZVPP/CPCM(THF) level of theory

Complex	$Mull_{C1}$	$Loew_{C1}$	$Hirsh_{Ni}$	NBO_{C1}
**6**	-0.018555	-0.08889	-0.085432	-0.21796
**21**	-0.02154	-0.092808	-0.089006	-0.2265
**22**	-0.021311	-0.092717	-0.088842	-0.22627
**23**	-0.021477	-0.090738	-0.0867	-0.22079
**24**	-0.020981	-0.090031	-0.085969	-0.22016
**25**	-0.021654	-0.088025	-0.08375	-0.21579
**26**	-0.021981	-0.0884	-0.084212	-0.21752
**27**	-0.058446	-0.104344	-0.100208	-0.25463
**28**	-0.042146	-0.094211	-0.090976	-0.2295
**29**	-0.025401	-0.068638	-0.068933	-0.18146
**30**	-0.017177	-0.07375	-0.072656	-0.18895
**31**	-0.032497	-0.077183	-0.076085	-0.20057

Table S10. Dipole moment of the nickel–aryl fragment calculated using B3LYP-D3/def2-TZVPP/CPCM(THF) level of theory

$[Ni–Aryl]^+$	$Dipole_{Ni–Ar}$
Ni(Ph), **S10**	6.27013
Ni(*p*-MeO-C_6H_4), **S11**	9.43975
Ni(*p*-tol), **S12**	7.50387
Ni(*p*-CHO-C_6H_4), **S13**	14.01844
Ni(*p*-COMe-C_6H_4), **S14**	14.55431
Ni(*p*-CF_3-C_6H_4), **S15**	17.48496
Ni(*p*-CN-C_6H_4), **S16**	16.04271

Table S11. Calculated Mulliken charge descriptors using B3LYP-D3/def2-SVP level of theory

Complex	$Mull_{C1}$
**6**	-0.103502
**21**	-0.107255
**22**	-0.107009
**23**	-0.106863
**24**	-0.106354
**25**	-0.105832
**26**	-0.105207
**27**	-0.096296
**28**	-0.099593
**29**	-0.093730

**30**	-0.093598
**31**	-0.090276
**32**	-0.091957
**33**	-0.098792
**34**	-0.101274
**35**	-0.093726

14. Evaluation of Computationally Determined MO Energies

To evaluate the accuracy of computationally derived orbital energies, the HOMO and LUMO energies determined using BP86/def2-SVP and B3LYP-D3/def2-TZVPP were compared to electrochemical potentials for the Ni^{II}/Ni^{I} couple and Ni^{III}/Ni^{II} couple for complexes **6**, **21-25**, and **27-32**. $E_{1/2}$ (Ni^{II}/Ni^{I}) should reflect the LUMO energy of the nickel(II) complex, while E_{pa} (Ni^{III}/Ni^{II}) should reflect the HOMO energy. Regardless of which basis set and functional was used, the LUMO energy had a good correlation with $E_{1/2}$ (Ni^{II}/Ni^{I}). In contrast, the HOMO energy was poorly correlated with E_{pa} (Ni^{III}/Ni^{II}). Comparing E_{pa} (Ni^{III}/Ni^{II}) with $HOMO_{Ni}$, the highest occupied molecular orbital localized on nickel using the BP86/def2-SVP level of theory, gave a better fit, but still insufficient. Due to greater inaccuracy and localization of the HOMO off nickel with B3LYP-D3/def2-TZVPP, molecular orbital plots were generated exclusively with BP86/def2-SVP level of theory.

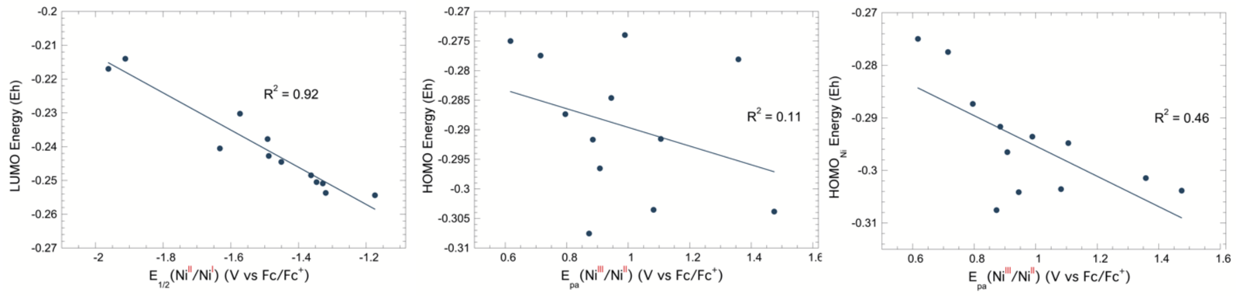

Figure S57. Comparison of electrochemical potentials with computationally derived MO energies, using BP86/def2-SVP level of theory.

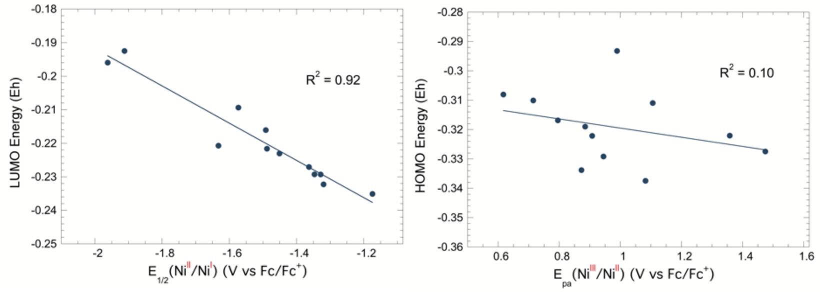

Figure S58. Comparison of electrochemical potentials with computationally derived MO energies, using B3LYP-D3/def2-TZVPP level of theory.

15. Initial Data Modeling with Computational Parameters

Initial screening of purely computational parameters was carried out with BP86/def2-SVP level of theory and included charge, electronic structure, and bond strength parameters. Attempts at fitting the kinetic data for singly-substituted complexes using computational descriptors resulted in modest fits. As $[(^{iPr}\text{pybox})\text{Ni}(p\text{-CN-}C_6H_4)]\text{BAr}^F_4$ **32** was an outlier for the plots involving only Hammett values, it was excluded from attempts at fitting with computational descriptors. Complex **26** and **33-35** were incorporated after initial computational screening and are thus excluded from the following models. Generally, the only parameters that were significant were the HOMO of the full complex and the charge on *ipso* carbon (C1) of the aryl group.

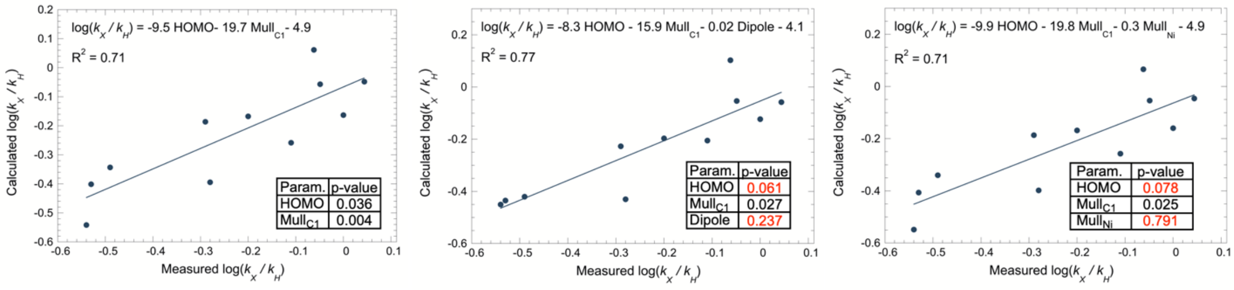

Figure S59. Three best regression fits using only computational parameters with BP86/def2-SVP level of theory. The individual p-value of each parameter is shown in the bottom right table. P-values greater than 0.05 are considered statistically insignificant and are highlighted in red.

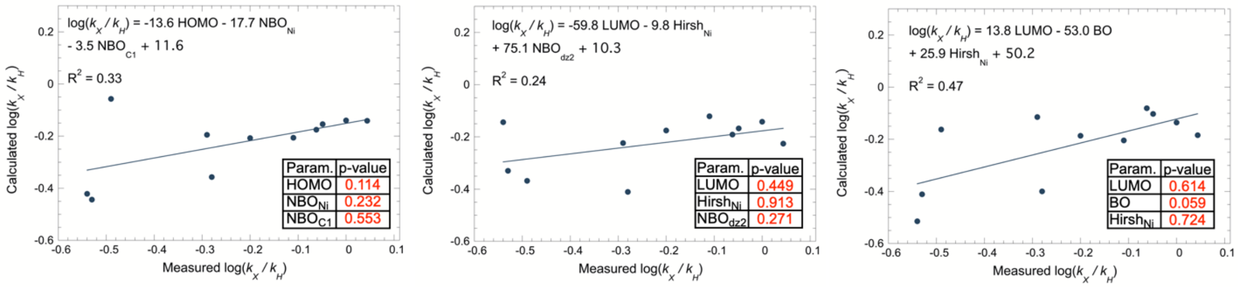

Figure S60. Three alternative regression fits using only computational parameters with BP86/def2-SVP level of theory, for comparison to the better fits. The individual p-value of each parameter is shown in the bottom right table. P-values greater than 0.05 are considered statistically insignificant and are highlighted in red.

16. Linear regression analysis with Mixed Experimental and Computational Descriptors

As computational and experimental values alone provided insufficient fits and MO energies did not accurately reflect experimental values, we sought a model that had a mix of experimental and computational values. Prior screening of computational descriptors alone revealed that the charge on C1 of the nickel complex is an important factor.

Attempts to fit the data using $E_{1/2}$ (Ni^{II}/Ni^{I}), E_{pa} (Ni^{III}/Ni^{II}), and the charge on C1 yielded poor correlations ($R^2 < 0.34$) across multiple charge models, including NBO, Löwdin, Mulliken, and Hirshfeld, at the B3LYP-D3/def2-TZVPP/CPCM(THF) level of theory. However, for the Löwdin, Hirshfeld, and NBO charge models, improved fits ($R^2 > 0.75$) were achieved by treating [(^{iPr}pybox)Ni(*p*-MeO-C_6H_4)]BAr^F_4 **27** as an outlier. Given that the *p*-OMe group is resonance-donating but inductively withdrawing, we suspected that the positive charge stabilization in this case involved subtle inductive and field effects not well captured by these charge models. To better account for inductive contributions, we tested a model using the dipole moment of the nickel–aryl fragment, which provided an excellent fit to the data ($R^2 = 0.86$) and accurately predicted the test complexes **33-35** well ($R^2_{test} = 0.89$). The Mulliken charge model also yielded a good fit ($R^2 = 0.76$) when calculated using the higher level of BP86/def2-SVP, and improved further with B3LYP-D3/def2-SVP level of theory ($R^2 = 0.85$).[27] Unlike models such as Hirshfeld,

which partition electron density in real space, Mulliken analysis partitions based on atomic wavefunctions.[27] We suspect that this key difference enables the Mulliken model to better capture the inductive and field effects on the *ipso*-carbon charge, even for substituents with divergent inductive and resonance characteristics. For the final fit, values of $E_{1/2}$ (Ni^{II}/Ni^{I}), E_{pa} (Ni^{III}/Ni^{II}), $Dipole_{Ni–Ar}$, and $Mull_{C1}$ were normalized to obtain comparable coefficients.

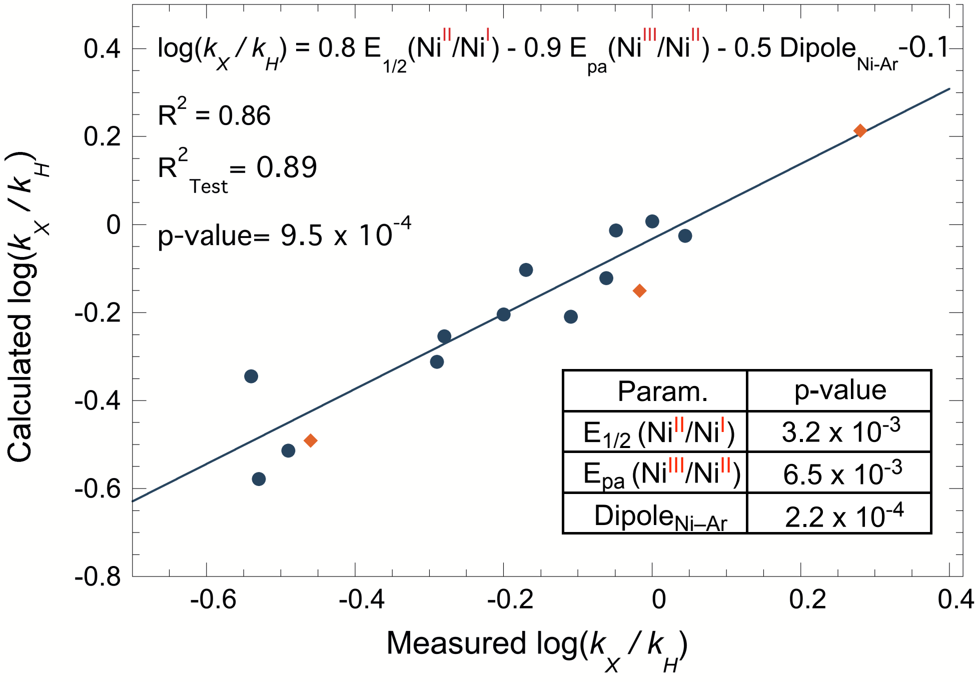

Figure S61. Final fit of kinetic data with $E_{1/2}$ (Ni^{II}/Ni^{I}), E_{pa} (Ni^{III}/Ni^{II}), and the dipole moment of the nickel aryl fragment ($Dipole_{Ni–Ar}$) calculated with B3LYP-D3/def2-TZVPP/CPCM(THF) level of theory. Complexes **33-35** are shown in orange. The p-value of the overall fit is displayed in the top left corner of the plot, while individual p-values of each parameter is shown in the bottom right table.

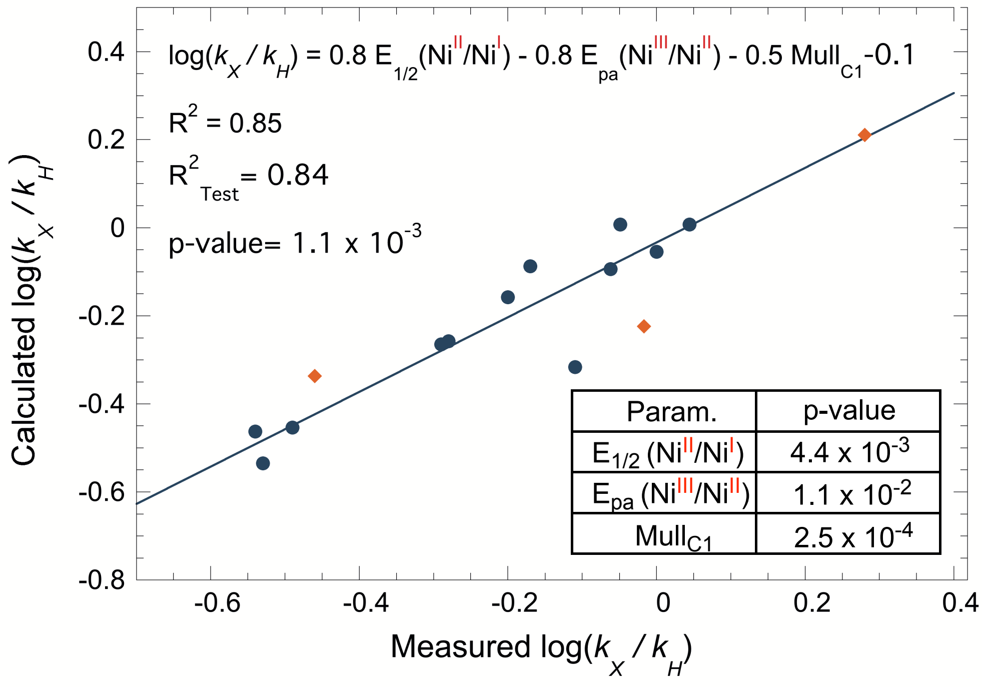

Figure S62. Final fit of kinetic data with $E_{1/2}$ (Ni^{II}/Ni^{I}), E_{pa} (Ni^{III}/Ni^{II}), and the Mulliken charge on the *ipso*-carbon bound to nickel ($Mull_{C1}$) calculated with B3LYP-D3/def2-SVP level of theory. Complexes **33-35** are shown in orange. The p-value of the overall fit is displayed in the top left corner of the plot, while individual p-values of each parameter is shown in the bottom right table.

17. Test for Collinearity in Mixed Computational and Experimental Model

To ensure no collinearity in the model involving the nickel–aryl dipole moment, $E_{1/2}$ (Ni^{II}/Ni^{I}), E_{pa} (Ni^{III}/Ni^{II}), and $Dipole_{Ni–Ar}$ calculated using B3LYP-D3/def2-TZVPP/CPCM(THF) were plotted against each other. To ensure no collinearity in the model involving the Mulliken charge on the *ipso*-carbon (C1), $E_{1/2}$ (Ni^{II}/Ni^{I}), E_{pa} (Ni^{III}/Ni^{II}), and $Mull_{C1}$ calculated using B3LYP-D3/def2-SVP were plotted against each other. Additionally, Pearson's correlation coefficient (r), the coefficient of determination (R^2), and Variable Inflation Factor (VIF) were analyzed. All values obtained support a lack of collinearity among the parameters.

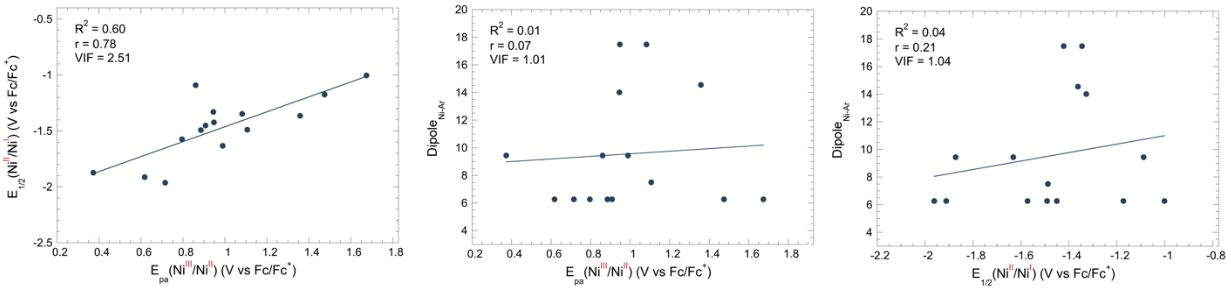

Figure S63. Evaluation of collinearity in independent variables involved in final mixed computational/experimental model with nickel–aryl dipole moment.

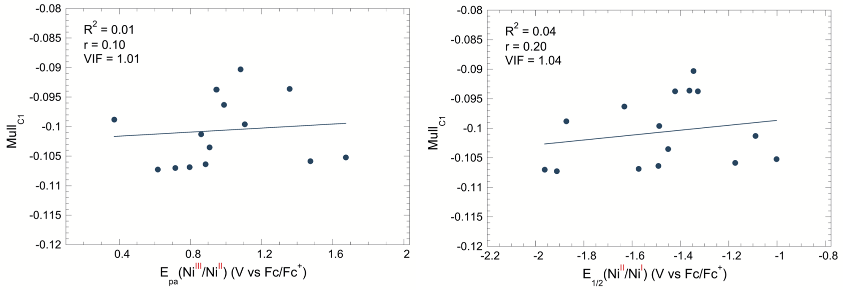

Figure S64. Evaluation of collinearity in independent variables involved in final mixed computational/experimental model with Mulliken charge on *ipso*-carbon.

18. Cross Validation for Mixed Computational and Experimental Models

We carried out cross validation to test for overfitting and to ensure that the regression coefficients

and R^2 values obtained for the final model are reliable. The full dataset for complexes **6** and **21-35**, excluding complex **32**, was randomly split into a train set and a validation set (~70:30, respectively). The data was optimized using the test set, then the validation set was added back without adjusting the regression coefficients. This process was repeated four times with different splits of test and validation set.

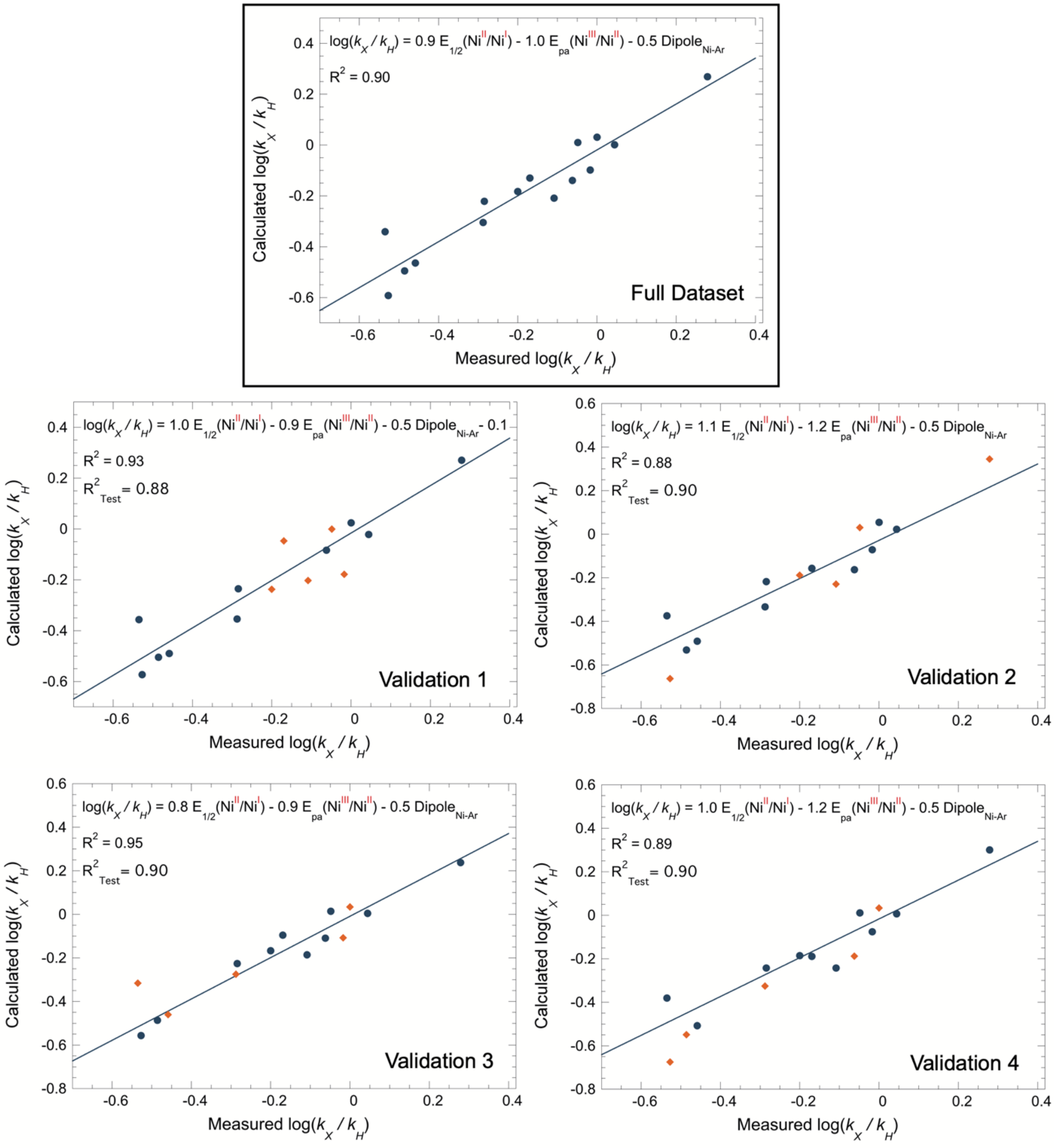

Figure S65. Cross validation plot for model involving nickel–aryl dipole moment. Plot for each iteration

with different split of train and test set, showing regression equation obtained for the train set. The R^2 value for the training set and R^2 value of the combined train and test set is shown.

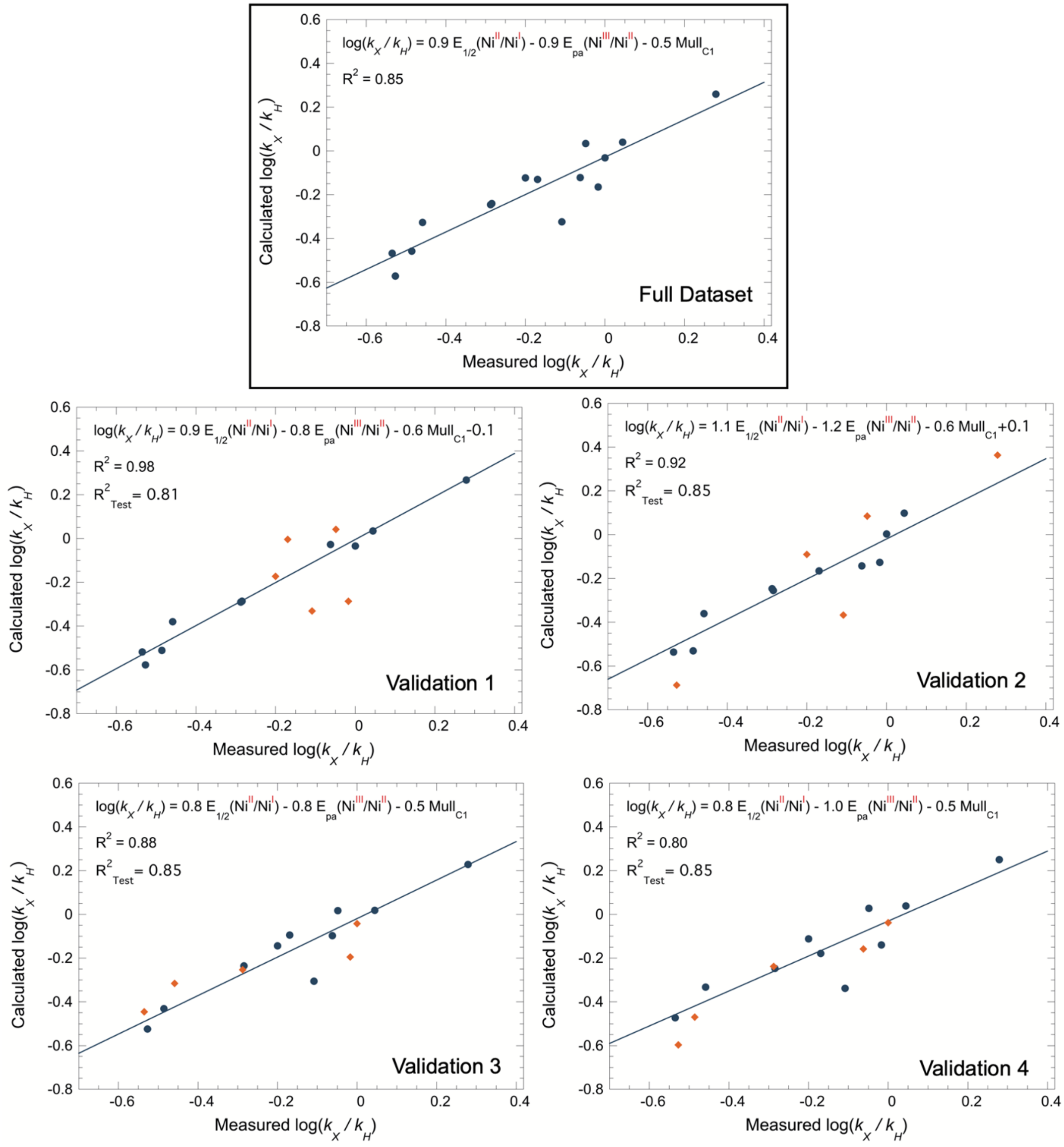

Figure S66. Cross validation plot for model involving *ipso*-carbon Mulliken charge. Plot for each iteration with different split of train and test set, showing regression equation obtained for the train set. The R^2 value for the training set and R^2 value of the combined train and test set is shown.

19. Complex LUMO Calculations for Redox Activity Series

LUMO energies of the fully nickel(II) aryl halide complex were calculated for complexes **3-5, 7-10, S1,** and **S9**. Geometry optimization and frequency calculation was carried out using BP86/def2-SVP level of theory. Frequency calculations using BP86/def2-SVP level of theory were also carried out on the free ligand, using the frozen geometry from optimization of the full nickel(II) phenyl bromide complex.

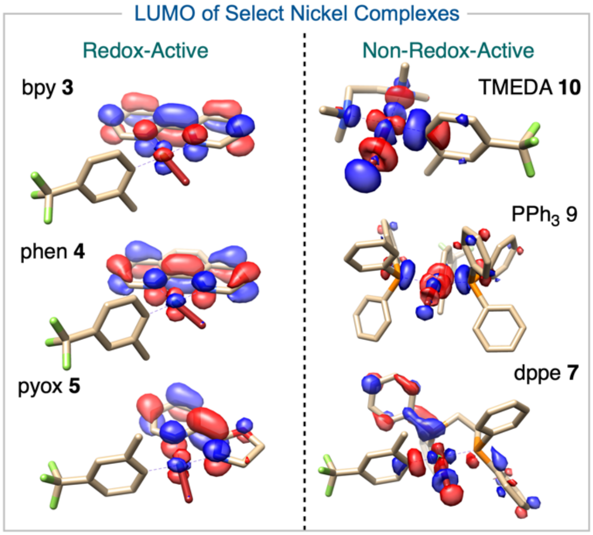

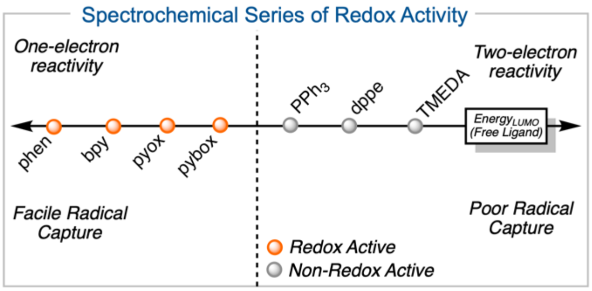

Figure S67. LUMO of select nickel complexes and spectrochemical series of ligand redox activity based on free ligand LUMO energy.

Table S12. Additional calculated complex and ligand LUMO energies using BP86/def2-SVP level of theory and radical capture yields

Complex	LUMO (hartrees)	LUMO (kcal/mol)	Radical Capture Yield (%)	$LUMO_{Free\ ligand}$ (hartrees)
(bpy)Ni(p-CF^3-$o$tol)Br **3**	-0.12837	-80.55	95[1]	-0.09052
(phen)Ni(p-CF^3-$o$tol)Br **4**	-0.12691	-79.64	99[1]	-0.09255
(pyox)Ni(p-CF^3-$o$tol)Br **5**	-0.12788	-80.24	90[1]	-0.08591
(dppe)Ni(p-CF^3-$o$tol)Br **7**	-0.08487	-53.26	N.D.	-0.06127
$(PPh_3)_2$Ni(p-CF^3-$o$tol)Br **9**	-0.08871	-55.67	9	-0.06412
$(PPh_3)_2$Ni(p-CF_3-C_6H_4)Br **8**	-0.08843	-55.49	2	-0.06412
(TMEDA)Ni(p-CF^3-$o$tol)Br **10**	-0.07481	-46.94	18	0.02539
(^{tBu}bpy)Ni(p-CF^3-$o$tol)Br **S1**	-0.11886	-74.58	92[1]	N/A
(^{CF3}bpy)Ni(p-CF^3-$o$tol)Br **S9**	-0.14979	-93.99	81[1]	N/A

20. Cartesian Coordinates of Optimized Structures

Gas-phase optimized geometries of full complexes using BP86/def2-SVP level of theory. Ligand and nickel–aryl geometries were taken from the optimized structure of each complex and used without further optimization.

Full Complexes

(bpy)Ni(p-CF_3-o-tol)Br **3**

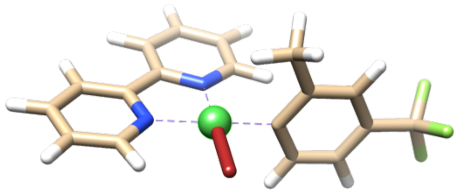

39

C	4.44942	2.98653	5.83916
Ni	4.50565	5.72415	6.88398
N	4.58466	4.31964	5.59993
Br	4.46371	7.43121	8.42504
C	4.54112	2.01774	4.83697
N	4.80241	6.87062	5.28972
C	4.78582	2.41653	3.51114
C	4.92325	3.78413	3.24897
C	4.81758	4.71489	4.29841
C	4.93489	6.16918	4.12397
C	5.15593	6.81962	2.89392
C	5.23948	8.21701	2.86402
C	5.10011	8.92906	4.06801
C	4.88434	8.21871	5.25532
C	4.18030	4.56353	8.33542
C	5.23165	3.95518	9.07141
C	4.90648	2.99656	10.05336
C	3.57064	2.64667	10.32634
C	2.53114	3.27189	9.61552
C	2.84245	4.22753	8.63455
C	6.68520	4.30703	8.82909
H	5.71883	2.51661	10.62358
C	3.28552	1.61702	11.39059
H	2.01651	4.71984	8.09634
H	1.48412	3.01589	9.83347
H	4.77161	8.71188	6.23711
H	5.41261	8.74277	1.91293
H	5.15914	10.02706	4.09489
H	5.26256	6.23713	1.96829
H	4.41885	0.95839	5.10578
H	4.25791	2.70853	6.88454
H	5.11494	4.13684	2.22624
H	4.86665	1.67868	2.69943
H	6.78039	5.09485	8.05609
H	7.27387	3.42205	8.50395
H	7.16162	4.69233	9.75512

F	1.96167	1.34151	11.50711
F	3.71935	2.01797	12.61688
F	3.91708	0.43581	11.13240

(phen)Ni(*p*-CF_3-*o*-tol)Br **4**

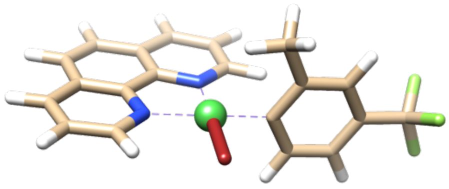

41

C	4.53703	2.94070	5.83883
Ni	4.53486	5.70643	6.89663
N	4.65379	4.27326	5.64162
Br	4.41687	7.44932	8.39025
C	4.65457	2.00083	4.79451
N	4.87281	6.85605	5.30200
C	4.90171	2.42129	3.48701
C	5.03032	3.81327	3.23596
C	4.89799	4.69298	4.34882
C	5.01611	6.10551	4.16661
C	5.26618	6.65844	2.87676
C	5.36714	8.07332	2.79572
C	5.21938	8.83036	3.95812
C	4.97362	8.18925	5.19467
C	4.17844	4.55160	8.34064
C	5.21272	3.97914	9.12741
C	4.87035	3.02038	10.10377
C	3.53362	2.63647	10.32120
C	2.50991	3.22667	9.55941
C	2.83842	4.18069	8.58235
C	6.66424	4.37474	8.95185
H	5.66940	2.56872	10.71441
C	3.23057	1.60312	11.37638
H	2.02433	4.64378	8.00185
H	1.46160	2.94382	9.73357
H	4.85191	8.74393	6.14186
H	5.56105	8.55573	1.82507
H	5.29296	9.92767	3.93168
H	4.54568	0.93322	5.03601
H	4.33936	2.62144	6.87168
H	4.99629	1.69865	2.66223
C	5.39781	5.75286	1.76436
C	5.28418	4.39005	1.93838
H	5.59410	6.17054	0.76474
H	5.38812	3.71179	1.07721
F	1.90494	1.32585	11.46873
F	3.64322	1.99942	12.61148
F	3.86695	0.42264	11.12492
H	6.77682	5.13583	8.15478
H	7.30238	3.50097	8.69839
H	7.07157	4.81419	9.88697

(pyox)Ni(*p*-CF_3-*o*-tol)Br **5**

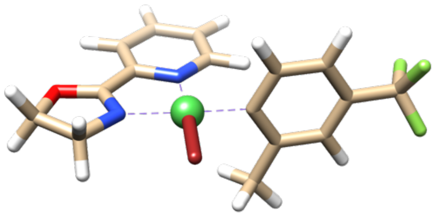

38

H	1.85318	2.41913	15.16558
C	2.72096	3.01378	14.81881
H	3.52975	2.32285	14.50680
H	1.28426	4.12828	13.50237
O	3.21821	3.78206	15.96818
C	2.36550	4.08429	13.74630
C	3.21385	5.06614	15.57155
Br	2.00235	6.97679	11.82989
N	2.77766	5.34209	14.37799
H	4.25378	5.05315	18.15335
C	1.79382	9.94885	14.02026
H	1.10011	12.00207	14.17463
C	4.17660	6.05211	17.70072
C	3.66447	6.17241	16.40170
C	1.93947	11.34525	13.89637
Ni	2.81611	7.25603	13.95641
C	4.57448	7.21268	18.37926
N	3.52832	7.38018	15.74234
C	2.88259	9.10983	13.65820
H	4.98082	7.15739	19.39926
C	3.92055	8.49166	16.41716
C	4.43952	8.44692	17.71741
C	3.12965	11.91819	13.40764
H	3.80790	9.44194	15.87613
H	4.73633	9.38849	18.20152
C	4.05869	9.68975	13.13906
C	3.28011	13.41699	13.35640
C	4.19261	11.08330	13.01824
H	5.11630	11.52049	12.61000
C	0.49290	9.35275	14.50873
H	4.89217	9.04803	12.81093
H	0.04486	8.71118	13.71998
H	-0.24275	10.13382	14.78656
H	0.65455	8.69831	15.39206
F	4.12443	13.81594	12.36998
F	3.78635	13.91699	14.52273
F	2.09359	14.04777	13.15336
H	2.90322	3.95022	12.78645

[(^{iPr}pybox)Ni(Ph)]$^+$ **6**

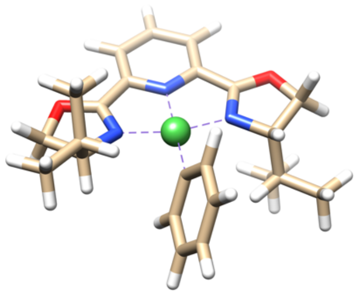

57

O	-1.01394	4.89002	24.55348
O	5.52051	7.45647	25.45150
N	0.76763	5.37875	23.24172
N	2.24643	6.16024	25.09193
N	4.22269	6.80514	23.71243
C	-0.20020	4.76846	22.29713
H	-0.34424	5.46489	21.44633
C	-1.48527	4.68138	23.17119
H	-2.22233	5.48158	22.96040
H	-1.99152	3.69915	23.14019
C	0.22963	5.33357	24.44354
C	1.05249	5.76798	25.58373
C	0.79277	5.81970	26.96455
C	1.82487	6.28580	27.80571
C	3.06919	6.69014	27.27772
C	3.24009	6.61436	25.88435
C	4.37076	6.96784	25.01102
C	6.38712	7.56965	24.26332
H	7.16391	6.78488	24.35823
H	6.86680	8.56477	24.30056
C	5.43026	7.36065	23.05311
H	5.83236	6.59591	22.35694
C	0.31429	3.41685	21.73025
H	1.27981	3.66273	21.23663
C	0.58572	2.36773	22.81921
H	1.31763	2.72658	23.57236
H	1.01045	1.44995	22.36644
H	-0.34001	2.06032	23.35164
C	-0.65111	2.89548	20.65293
H	-0.24658	1.97493	20.18794
H	-0.80820	3.63813	19.84433
H	-1.64504	2.63084	21.07330
C	5.10271	8.63423	22.22622
H	4.33693	8.30396	21.49234
C	6.34232	9.09264	21.44075
H	7.15444	9.45141	22.10842
H	6.08291	9.93465	20.76867
H	6.75375	8.27754	20.81052
C	4.49576	9.76564	23.06832
H	3.57128	9.44464	23.59010
H	4.22067	10.61930	22.41796
H	5.20268	10.15934	23.82901
C	2.82439	5.99566	21.34699
C	2.06584	6.80007	20.46956

H	1.29662	7.49073	20.85707
C	2.29436	6.74823	19.07941
H	1.70357	7.39071	18.40682
C	3.26796	5.88206	18.55406
H	3.44239	5.83928	17.46767
C	4.01878	5.07128	19.42240
H	4.78020	4.38536	19.01707
C	3.80536	5.13104	20.81437
H	4.40713	4.48312	21.47393
Ni	2.53799	6.07612	23.20404
H	-0.17995	5.50419	27.36666
H	3.87912	7.05523	27.92448
H	1.65590	6.33612	28.89174

(dppe)Ni(p-CF_3-o-tol)Br **7**

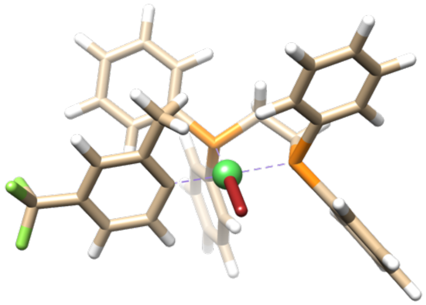

71

C	2.96783	6.59455	19.83471
C	2.39845	7.70208	19.14593
C	1.02178	8.20561	19.50762
C	3.11958	8.31194	18.10141
H	2.68025	9.17359	17.57480
C	4.39082	7.84388	17.71842
C	5.19188	8.59328	16.68766
C	4.93670	6.72053	18.36455
H	5.91732	6.32922	18.05634
C	4.22234	6.10692	19.40776
H	4.66959	5.22797	19.89748
Ni	2.04338	5.75565	21.29406
P	3.12506	7.00164	22.66128
P	0.99822	4.83462	23.05336
C	3.45210	8.78634	22.34239
C	4.78206	6.30482	23.10592
C	0.92285	3.01165	23.30781
C	-0.76205	5.37115	23.24950
C	-0.00023	2.26950	22.53286
C	-1.33854	6.20122	22.26614
C	5.75897	7.06090	23.79021
C	2.73502	9.81304	22.99691
C	-1.52834	4.99096	24.37436
C	1.78807	2.32253	24.18623
C	-0.06306	0.87265	22.65144
H	-0.65933	2.79206	21.82392
C	4.41488	9.13869	21.36756
C	6.97747	6.46749	24.15796
H	5.57059	8.11924	24.02937
C	5.04400	4.95310	22.79371

C	-2.65959	6.65904	22.41348
H	-0.74962	6.46167	21.37331
C	1.71869	0.92335	24.30271
H	2.52620	2.86713	24.79417
C	0.79236	0.19538	23.53803
H	-0.78642	0.30912	22.04177
C	-2.84623	5.45120	24.51822
H	-1.10082	4.31946	25.13687
C	-3.41250	6.28939	23.53958
H	-3.10194	7.30251	21.63704
C	6.26262	4.36154	23.16813
H	4.28259	4.36931	22.24906
C	7.23064	5.11881	23.84887
H	7.73805	7.06422	24.68569
C	4.65327	10.48658	21.05814
H	4.97513	8.35445	20.83694
C	2.98076	11.16187	22.68697
H	1.97523	9.57250	23.75540
C	3.93821	11.50127	21.71691
H	2.41618	11.94996	23.20833
H	2.39442	0.40153	24.99847
H	0.73763	-0.90087	23.63252
H	-4.44724	6.64763	23.65394
H	-3.43818	5.14905	25.39642
H	6.45815	3.30797	22.91622
H	8.18953	4.66000	24.13556
H	5.39255	10.73742	20.28197
H	4.12426	12.55823	21.47047
C	1.81831	5.51864	24.60067
C	2.22459	6.96743	24.31233
H	2.72465	4.90877	24.80013
H	1.15367	5.43816	25.48503
H	2.86240	7.39661	25.11302
H	1.32183	7.60575	24.20892
Br	0.87571	4.40961	19.77081
H	0.76376	9.13989	18.97047
H	0.94113	8.40135	20.59889
H	0.25984	7.43521	19.25981
F	4.40758	9.21942	15.77262
F	6.04541	7.78634	16.00560
F	5.96400	9.56911	17.26155

$(PPh_3)_2Ni(p\text{-}CF_3\text{-}C_6H)Br$ **8**

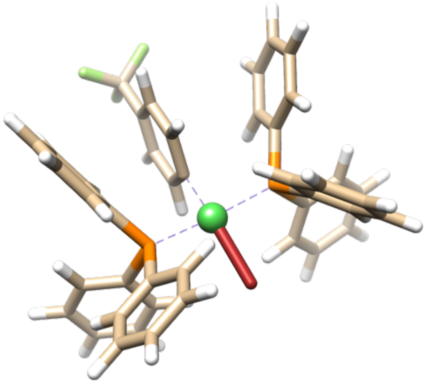

84

Br	8.25629	7.58250	10.93281

Ni	9.14811	7.07373	13.10595
P	11.08325	8.10380	12.65125
P	7.10942	6.43082	13.74748
C	11.79255	4.02453	17.84331
C	7.02843	4.36918	11.79149
H	7.91833	4.86081	11.37197
C	9.93235	6.25551	14.59996
C	10.50111	4.97804	14.36968
H	10.48320	4.53282	13.36030
C	12.14242	8.62063	14.08694
C	12.18989	9.96784	14.50873
H	11.64094	10.74011	13.94990
C	9.99488	6.77404	15.91509
H	9.57185	7.76572	16.13750
C	6.50286	3.21463	11.18638
H	7.00339	2.78936	10.30307
C	5.90631	7.80095	13.42702
C	11.66595	6.22116	10.63880
H	10.56974	6.17582	10.52789
C	6.39864	4.92742	12.92348
C	12.22995	7.08467	11.60474
C	13.61931	8.02519	15.94609
H	14.16132	7.25269	16.51269
C	12.86253	7.65218	14.82463
H	12.82450	6.59315	14.52905
C	13.65952	9.36848	16.35833
H	14.24594	9.65899	17.24396
C	13.63679	7.16526	11.71453
H	14.09759	7.83066	12.45979
C	4.71358	7.62098	12.69915
H	4.46124	6.63163	12.29117
C	10.92249	9.70216	11.72217
C	11.15402	4.78237	16.70928
C	6.80103	6.01070	15.53316
C	6.06224	6.83633	16.40632
H	5.60892	7.76777	16.03823
C	5.34581	2.60821	11.70224
H	4.93738	1.70266	11.22778
C	11.10495	4.24771	15.40835
H	11.54052	3.25810	15.20781
C	12.94124	10.33647	15.63811
H	12.96534	11.39183	15.95225
C	10.59368	6.05220	16.95640
H	10.62757	6.47984	17.97068
C	5.23849	4.30719	13.44382
H	4.74846	4.71889	14.33896
C	12.49414	5.45545	9.80220
H	12.03854	4.79097	9.05111
C	13.89230	5.53509	9.92059
H	14.53951	4.92952	9.26685
C	14.46032	6.39327	10.87717
H	15.55536	6.46591	10.97568

C	6.22670	9.08890	13.91415
H	7.16937	9.24826	14.46261
C	3.84894	8.70686	12.47746
H	2.92285	8.55344	11.90190
C	7.35276	4.80914	16.03574
H	7.93560	4.15308	15.37163
C	4.71299	3.15833	12.83107
H	3.80683	2.68768	13.24233
C	7.17568	4.44781	17.37878
H	7.62631	3.51693	17.75467
C	11.95201	10.17120	10.87968
C	9.77186	10.49717	11.91189
C	4.16296	9.97976	12.98023
H	3.48294	10.82775	12.80404
C	5.88639	6.47110	17.75390
H	5.30492	7.12700	18.42085
C	5.35506	10.16834	13.70161
H	5.61296	11.16421	14.09391
C	6.44307	5.27981	18.24429
H	6.30684	4.99753	19.29970
C	11.83693	11.42123	10.24819
H	12.84850	9.55599	10.71031
C	9.66518	11.75134	11.28948
H	8.94553	10.11915	12.53189
C	10.69686	12.21615	10.45546
H	12.64563	11.77292	9.58845
H	8.76143	12.36088	11.44494
H	10.60927	13.19558	9.96045
F	12.28607	2.82131	17.45529
F	10.90985	3.78593	18.85442
F	12.82673	4.71834	18.40052

$(PPh_3)_2Ni(p\text{-}CF_3\text{-}o\text{-}tol)Br$ **9**

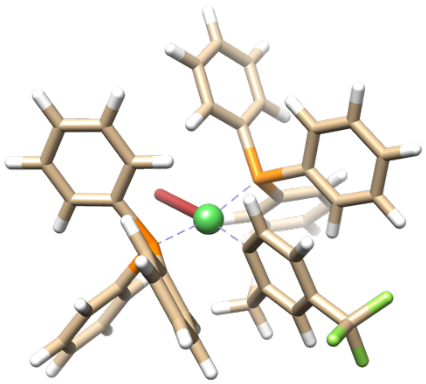

87

Br	8.33695	7.34521	10.85103
Ni	9.21691	6.87871	13.04840
P	11.11281	7.98417	12.57368
P	7.15894	6.30032	13.69321
C	11.68460	4.56335	18.28482
C	6.85991	4.29557	11.68672
H	7.77416	4.72219	11.24905
C	9.95759	6.21365	14.65171
C	10.54233	4.91403	14.63648
C	10.56350	4.08754	13.36894
C	12.18936	8.47471	14.00444

C	12.21576	9.80414	14.47871	C	5.02983	2.68716	11.60586
H	11.64170	10.58582	13.95978	H	4.53240	1.83172	11.12306
C	9.94707	6.93157	15.86916	C	11.10106	4.39266	15.82204
H	9.50722	7.94059	15.90763	H	11.55804	3.39151	15.80901
C	6.22159	3.20417	11.07222	C	12.97659	10.14244	15.61175
H	6.66099	2.75940	10.16639	H	12.98375	11.18435	15.96865
C	6.06182	7.76603	13.41667	C	10.49607	6.40311	17.04500
C	11.77402	6.32709	10.37912	H	10.47397	6.98785	17.97739
H	10.68123	6.23255	10.26507	C	5.11309	4.34948	13.38414
C	6.31034	4.87924	12.84758	H	4.68029	4.78174	14.29881
C	12.29741	7.11873	11.42692	C	12.63727	5.69052	9.47261
C	13.70446	7.83470	15.81683	H	12.21309	5.08143	8.65874
H	14.26877	7.05301	16.34740	C	14.03021	5.82840	9.59932
C	12.94031	7.49275	14.69133	H	14.70414	5.32387	8.88932
H	12.91625	6.44582	14.35488	C	14.55758	6.61557	10.63633
C	13.72281	9.16112	16.28287	H	15.64765	6.73499	10.74261
H	14.31365	9.42655	17.17335	C	6.42784	8.99608	14.01126
C	13.69911	7.25844	11.54495	H	7.33489	9.05763	14.63452
H	14.12837	7.87003	12.35235	C	4.13850	8.86906	12.39616
C	4.91469	7.71500	12.59990	H	3.24695	8.81569	11.75202
H	4.62957	6.77249	12.11082	C	7.37950	4.64262	15.95280
C	10.82612	9.62300	11.74819	H	7.95028	3.98786	15.27744
C	11.08351	5.12400	17.02313	C	4.47512	3.26426	12.76188
C	6.84308	5.85976	15.47241	H	3.54161	2.86513	13.18710
C	6.10622	6.67265	16.35857	C	7.19724	4.25780	17.28833
H	5.66158	7.61451	16.00735	H	7.63264	3.31294	17.64655

C	11.81493	10.21686	10.93599
C	9.62168	10.31777	11.98617
C	4.49682	10.08348	13.00335
H	3.88611	10.98519	12.84092
C	5.92341	6.28337	17.69850
H	5.34518	6.93117	18.37609
C	5.64409	10.14425	13.81374
H	5.93573	11.09281	14.29064
C	6.47077	5.08006	18.16827
H	6.33189	4.78007	19.21830
C	11.60650	11.49114	10.38241
H	12.75338	9.68133	10.72717
C	9.42118	11.59589	11.44077
H	8.82645	9.84326	12.58013
C	10.41206	12.18564	10.63736
H	12.38467	11.94065	9.74579
H	8.47522	12.12602	11.63232
H	10.24999	13.18409	10.20294
H	11.12315	4.59903	12.55768
H	9.53627	3.91631	12.98117
H	11.03334	3.09664	13.52970
F	12.24270	3.33849	18.10074
F	10.76016	4.43156	19.27776
F	12.66234	5.37414	18.78343

(TMEDA)Ni(*p*-CF_3-*o*-tol)Br **10**

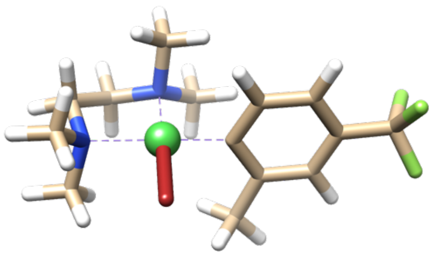

43

C	2.90387	6.66299	19.97274
C	1.97812	7.33566	19.12240
C	0.48743	7.20099	19.33822
C	2.46059	8.08752	18.03329
H	1.74570	8.61341	17.38068
C	3.83662	8.16136	17.74106
C	4.33031	9.02623	16.61141
C	4.74866	7.44854	18.53865
H	5.82174	7.45942	18.29363
C	4.27629	6.70624	19.63416
H	5.00493	6.12026	20.21875
Ni	2.36530	5.77016	21.52541
N	2.72902	7.35853	22.72157
N	1.72138	4.75912	23.27390
C	2.05315	5.66477	24.39784
C	1.92845	7.11206	23.95451
H	3.09269	5.45006	24.71697
H	1.40529	5.47566	25.28651
H	2.24388	7.80380	24.77207
H	0.87294	7.35292	23.71380

Br	1.97180	3.92717	20.17800
C	0.25647	4.59145	23.13210
H	0.06024	3.98788	22.22524
H	-0.23679	5.57396	23.00518
H	-0.17612	4.08600	24.02882
C	2.34887	3.43464	23.47607
H	3.44735	3.55053	23.55664
H	2.13358	2.80311	22.59359
H	1.96183	2.94733	24.40194
C	2.37454	8.69685	22.18840
H	2.93833	8.89097	21.25945
H	2.61091	9.48568	22.93943
H	1.29436	8.73349	21.95455
C	4.18418	7.33996	23.00972
H	4.49576	6.35507	23.40323
H	4.44886	8.12803	23.75488
H	4.73397	7.52771	22.06970
H	-0.09350	7.85784	18.66032
H	0.19304	7.43735	20.38337
H	0.17527	6.14886	19.16577
F	3.39789	9.18592	15.63537
F	5.44662	8.51705	16.02629
F	4.66062	10.28243	17.03616

[(4-Pyrrolidinyl-$^{i\mathrm{Pr}}$pybox)Ni(Ph)]$^{+}$ **21**

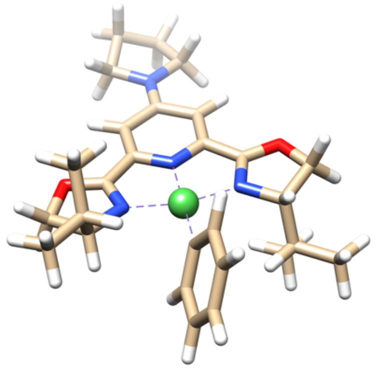

69

O	-0.89316	4.60063	24.70438
O	5.69362	7.07454	25.44333
N	0.79952	5.23185	23.33501
N	2.39339	5.83479	25.14285
N	4.28818	6.59770	23.73007
C	-0.23243	4.72869	22.39638
H	-0.45148	5.52876	21.66043
C	-1.44770	4.49990	23.34389
H	-2.23211	5.27804	23.25720
H	-1.91468	3.50133	23.25220
C	0.34107	5.06671	24.55960
C	1.23440	5.39867	25.68200
C	1.04131	5.32622	27.06040
C	2.12070	5.72579	27.92786
C	3.34571	6.17963	27.31932
C	3.42310	6.21833	25.92828
C	4.50934	6.64100	25.02848
C	6.49471	7.27752	24.22388

H	7.26320	6.47863	24.20670
H	6.99330	8.26032	24.31752
C	5.47143	7.18330	23.05447
H	5.81981	6.46936	22.28018
C	0.24863	3.47868	21.61075
H	1.15348	3.81854	21.06171
C	0.65611	2.31307	22.52332
C	-0.80886	3.06476	20.57398
H	-0.43431	2.22748	19.95176
H	-1.06399	3.89938	19.88901
H	-1.74974	2.71542	21.05116
C	5.13325	8.52375	22.34701
H	4.31718	8.26812	21.63834
C	6.33810	9.01084	21.52612
H	7.20222	9.28265	22.16931
H	6.07067	9.91625	20.94559
H	6.68014	8.24202	20.80330
C	4.60925	9.60022	23.30820
H	3.71692	9.25387	23.86936
H	4.30713	10.50485	22.74378
H	5.37750	9.92128	24.04288
C	2.73983	6.03928	21.37515
C	1.94315	6.94369	20.63757
H	1.21455	7.59603	21.14904
C	2.07762	7.04156	19.23802
H	1.45619	7.75970	18.67880
C	2.99965	6.22806	18.55698
C	3.79056	5.31927	19.28117
H	4.51258	4.67446	18.75447
C	3.66688	5.22909	20.68232
H	4.29913	4.50716	21.22681
Ni	2.57049	5.93070	23.24480
H	3.10270	6.30292	17.46299
N	1.98466	5.68003	29.27626
C	0.78743	5.16186	29.96588
C	3.00480	6.15858	30.22872
C	2.25403	6.20968	31.56849
H	3.39562	7.15001	29.91995
H	3.86519	5.45086	30.26223
C	1.22135	5.07778	31.43787
H	1.74345	7.18896	31.67754
H	2.93239	6.08849	32.43413
H	0.49117	4.17640	29.55059
H	-0.06886	5.86064	29.82528
H	0.36581	5.18077	32.13205
H	1.69552	4.09408	31.63762
H	4.20801	6.48741	27.92261
H	0.08001	4.98097	27.45964
H	1.05408	1.47352	21.91929
H	-0.20104	1.91094	23.10450
H	1.45127	2.60890	23.23794

[(4-NMe$_2$-^{iPr}pybox)Ni(Ph)]$^+$ **22**

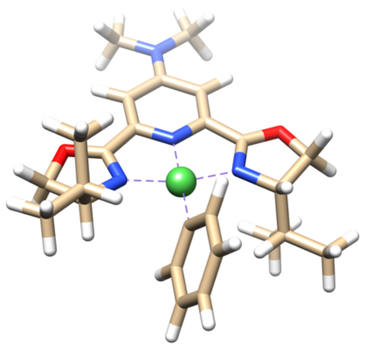

65

O	-0.95434	4.60534	24.57720
O	5.59338	7.12562	25.47538
N	0.77713	5.21874	23.24832
N	2.30917	5.86764	25.09295
N	4.24514	6.61034	23.72744
C	-0.22173	4.69029	22.28787
H	-0.42848	5.47787	21.53489
C	-1.46239	4.45982	23.20234
H	-2.26000	5.21767	23.07144
H	-1.90464	3.44886	23.12151
C	0.28029	5.07726	24.46076
C	1.13484	5.44097	25.60359
C	0.89716	5.39901	26.97683
C	1.94490	5.81990	27.87175
C	3.18771	6.26142	27.29203
C	3.31103	6.26982	25.90319
C	4.42468	6.67936	25.03119
C	6.43319	7.30442	24.27783
H	7.19801	6.50202	24.29883
H	6.93236	8.28696	24.36956
C	5.44599	7.19188	23.07939
H	5.81948	6.47001	22.32441
C	0.29528	3.43551	21.53367
H	1.21005	3.77966	21.00439
C	0.69291	2.28838	22.47382
H	1.47015	2.60254	23.20046
H	1.11041	1.44275	21.89185
H	-0.17327	1.88819	23.04315
C	-0.72834	2.99242	20.47525
H	-0.32443	2.15146	19.87691
H	-0.97604	3.81287	19.77077
H	-1.67674	2.63621	20.93185
C	5.12344	8.52217	22.34607
H	4.33249	8.25436	21.61385
C	6.35183	9.00728	21.55901
H	7.19165	9.29477	22.22705
H	6.09706	9.90230	20.95711
H	6.72401	8.23103	20.85939
C	4.56217	9.60647	23.27682
H	3.65185	9.26246	23.80945
H	4.27613	10.50332	22.69210
H	5.30211	9.93984	24.03479
C	2.77397	6.00632	21.33524

C	1.99291	6.89360	20.56129
H	1.24333	7.54666	21.04071
C	2.16884	6.97246	19.16511
H	1.55881	7.67712	18.57722
C	3.11731	6.15682	18.52429
C	3.89285	5.26499	19.28516
H	4.63539	4.61870	18.78979
C	3.72773	5.19360	20.68318
H	4.34835	4.48394	21.25637
Ni	2.54682	5.92671	23.20016
H	3.25296	6.21674	17.43301
N	1.76521	5.80273	29.22385
C	0.49282	5.36018	29.79287
H	0.27694	4.29981	29.54088
H	-0.35300	5.98615	29.43673
H	0.53847	5.44620	30.89258
C	2.84323	6.22715	30.11577
H	3.13232	7.28446	29.93348
H	3.74594	5.59009	29.99920
H	2.50194	6.14423	31.16222
H	-0.07358	5.04952	27.34543
H	4.03006	6.59515	27.90831

[(4-OMe-^{iPr}pybox)Ni(Ph)]$^+$ **23**

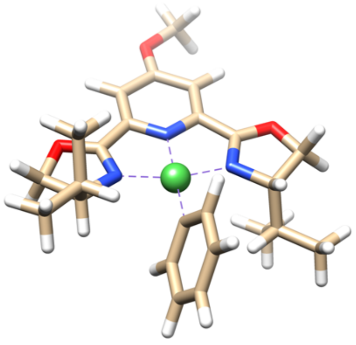

61

O	-0.85450	4.50505	24.60452
O	5.65013	7.17833	25.37711
N	0.83524	5.16359	23.24405
N	2.38842	5.83666	25.06790
N	4.27498	6.64314	23.65674
C	-0.17218	4.61908	22.30109
H	-0.40398	5.40333	21.55220
C	-1.39151	4.36896	23.23860
H	-2.19715	5.12244	23.13365
H	-1.82780	3.35560	23.15753
C	0.36682	5.00240	24.46505
C	1.23682	5.37652	25.59251
C	1.02898	5.31756	26.97845
C	3.30668	6.23798	27.24344
C	3.40469	6.26197	25.85229
C	4.48511	6.70705	24.95613
C	6.45631	7.39494	24.16174
H	7.24805	6.61922	24.16023

H	6.92346	8.39314	24.25191
C	5.44567	7.25954	22.98486
H	5.82111	6.54986	22.21928
C	0.35306	3.37212	21.53884
C	0.77327	2.22764	22.47236
H	1.55105	2.54774	23.19597
H	1.19877	1.38997	21.88485
H	-0.08355	1.81293	23.04529
C	-0.67593	2.91936	20.48952
H	-0.26894	2.08128	19.88915
H	-0.93666	3.73682	19.78650
H	-1.61751	2.55557	20.95424
C	5.07157	8.58326	22.26377
H	4.27517	8.29634	21.54478
C	6.27092	9.10632	21.45749
H	7.11187	9.42125	22.11118
H	5.97829	9.99238	20.86007
H	6.65605	8.34275	20.75105
C	4.49544	9.64663	23.20934
H	3.60205	9.27637	23.75251
H	4.17787	10.53869	22.63408
H	5.23577	9.99639	23.95914
C	2.76945	6.00828	21.29930
C	1.95301	6.88020	20.54612
H	1.20352	7.51918	21.04317
C	2.09730	6.96264	19.14631

H	1.46167	7.65580	18.57270
C	3.04671	6.16550	18.48448
C	3.85721	5.28916	19.22691
H	4.60217	4.65834	18.71478
C	3.72539	5.21427	20.62839
H	4.37328	4.51813	21.18780
Ni	2.58526	5.91444	23.16721
H	3.15708	6.22860	17.39046
O	2.04406	5.76404	29.15495
C	2.09097	5.75806	27.81885
C	0.86090	5.31830	29.83298
H	1.06912	5.43878	30.91057
H	0.65368	4.25012	29.61348
H	-0.01524	5.94110	29.55592
H	1.25834	3.72879	21.00117
H	0.07850	4.94405	27.37847
H	4.12202	6.57742	27.89644

[(4-Me-$^{i\mathrm{Pr}}$pybox)Ni(Ph)]$^{+}$ **24**

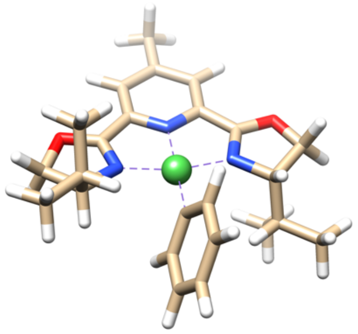

60

O	-0.92540	4.64278	24.59094
O	5.60864	7.22515	25.40916
N	0.79130	5.24859	23.24044
N	2.32975	5.93938	25.07536
N	4.25393	6.67737	23.67682
C	-0.21237	4.70271	22.29373
H	-0.42960	5.48086	21.53392
C	-1.44437	4.47594	23.22147
H	-2.24825	5.22669	23.08920
H	-1.88003	3.46109	23.15839
C	0.30374	5.11874	24.45753
C	1.16201	5.50358	25.59007
C	0.95087	5.47523	26.97668
C	1.99383	5.91032	27.83689
C	3.21275	6.35527	27.26123
C	3.33988	6.35884	25.86371
C	4.44272	6.76564	24.97765
C	6.43842	7.40350	24.20307
H	7.21664	6.61455	24.23004
H	6.92124	8.39519	24.28089
C	5.44502	7.26135	23.01224
H	5.82178	6.53125	22.26665
C	0.30336	3.44131	21.54963
H	1.21042	3.78214	21.00524
C	0.71411	2.30642	22.49882
H	1.49595	2.62984	23.21689
H	1.13124	1.45686	21.92226
H	-0.14610	1.90749	23.07816
C	-0.73061	2.98241	20.50794
H	-0.33085	2.13568	19.91531
H	-0.98887	3.79377	19.79689
H	-1.67250	2.62780	20.97926
C	5.10474	8.57657	22.25917
H	4.31846	8.28746	21.53009
C	6.32766	9.06556	21.46591
H	7.15783	9.38807	22.13000
H	6.05859	9.93956	20.83999
H	6.71929	8.27917	20.78851
C	4.52849	9.66718	23.17280
H	3.62087	9.32130	23.70852
H	4.23369	10.55178	22.57428
H	5.26183	10.02153	23.92754
C	2.77742	6.01262	21.31177
C	1.99202	6.87877	20.52011
H	1.24007	7.54083	20.98331
C	2.16949	6.92408	19.12235
H	1.55805	7.61285	18.51734
C	3.12074	6.09550	18.50258
C	3.89889	5.22413	19.28448
H	4.64284	4.56755	18.80507
C	3.73483	5.18622	20.68403

H	4.35735	4.49264	21.27461
Ni	2.55598	5.96924	23.17866
H	3.25652	6.12934	17.41017
C	1.79834	5.92534	29.32936
H	1.30233	6.87097	29.64073
H	2.76135	5.86778	29.87064
H	1.14943	5.09338	29.66546
H	4.04646	6.69347	27.89342
H	-0.00586	5.11840	27.38392

[(4-CF$_3$-^{iPr}pybox)Ni(Ph)]$^+$ **25**

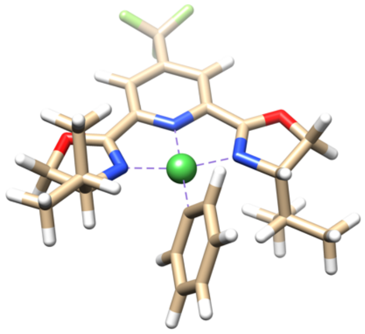

60

O	-0.92802	4.66106	24.58574
O	5.60958	7.23947	25.41214
N	0.79634	5.25176	23.23853
N	2.33366	5.94181	25.07864
N	4.26194	6.67767	23.67862
C	-0.20395	4.69846	22.29141
H	-0.41133	5.46779	21.52010

C	-1.44306	4.49056	23.21385
H	-2.23790	5.24941	23.07405
H	-1.88877	3.47997	23.15602
C	0.30179	5.13128	24.45369
C	1.15821	5.51586	25.58657
C	0.93590	5.50094	26.97281
C	1.99018	5.93471	27.80628
C	3.21464	6.38108	27.26183
C	3.34481	6.36884	25.86392
C	4.44850	6.77386	24.97909
C	6.44363	7.41287	24.20671
H	7.22280	6.62543	24.24152
H	6.92376	8.40599	24.28138
C	5.45303	7.26362	23.01423
H	5.83298	6.53206	22.27181
C	0.31157	3.42360	21.56968
H	1.22787	3.74985	21.03162
C	0.70167	2.29772	22.53848
H	1.47998	2.62163	23.25998
H	1.11562	1.43622	21.97792
H	-0.16712	1.91458	23.11520
C	-0.71417	2.96017	20.52161
H	-0.31366	2.10278	19.94508
H	-0.95693	3.76437	19.79716
H	-1.66449	2.61956	20.98574
C	5.10906	8.57627	22.25788

H	4.31836	8.28479	21.53424
C	6.32782	9.06167	21.45642
H	7.16227	9.38601	22.11417
H	6.05464	9.93400	20.82997
H	6.71505	8.27367	20.77855
C	4.53775	9.67059	23.17048
H	3.63330	9.32820	23.71429
H	4.23856	10.55207	22.56941
H	5.27549	10.02981	23.91861
C	2.78799	6.00009	21.31553
C	1.98946	6.84701	20.51745
H	1.22715	7.50151	20.97361
C	2.16952	6.88214	19.11986
H	1.54872	7.55584	18.50782
C	3.13398	6.06201	18.50970
C	3.92445	5.21006	19.30009
H	4.67881	4.56055	18.82771
C	3.75991	5.18279	20.69979
H	4.39183	4.50459	21.29750
Ni	2.56323	5.96632	23.18297
H	3.27053	6.08716	17.41727
C	1.82258	5.87400	29.32190
F	2.54208	6.84119	29.92633
F	2.25100	4.68017	29.78587
F	0.52855	6.02008	29.67106
H	4.03194	6.73232	27.90644
H	-0.02283	5.16748	27.39243

[(4-CO_2Me-$^{i\text{Pr}}$pybox)Ni(Ph)]$^+$ **26**

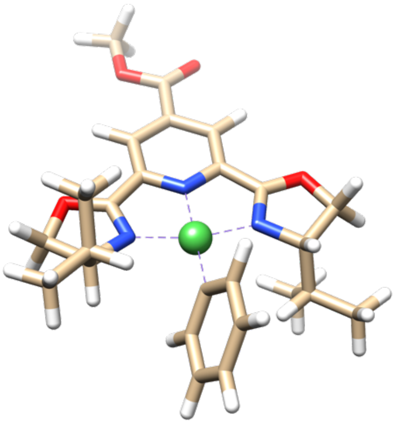

63

O	-0.94109	4.70104	24.60277
O	5.59583	7.28831	25.42252
N	0.78811	5.27678	23.25672
N	2.31616	5.99680	25.09335
N	4.25113	6.71106	23.69279
C	-0.20685	4.71137	22.31163
H	-0.41442	5.47239	21.53211
C	-1.44828	4.50850	23.23206
H	-2.24763	5.25973	23.07797
H	-1.88708	3.49418	23.18566
C	0.28871	5.17185	24.47114
C	1.13812	5.57553	25.60329
C	0.91204	5.57969	26.98668

C	1.95890	6.03019	27.82545
C	3.18963	6.46182	27.27618
C	3.32459	6.43189	25.87895
C	4.43295	6.82166	24.99256
C	6.43320	7.44155	24.21777
H	7.20489	6.64700	24.26176
H	6.92288	8.43076	24.28216
C	5.44432	7.28757	23.02500
H	5.82344	6.54985	22.28829
C	0.31418	3.43163	21.60312
H	1.23152	3.75590	21.06569
C	0.70382	2.31496	22.58262
H	1.47873	2.64699	23.30411
H	1.12138	1.44968	22.03055
H	-0.16606	1.93505	23.15983
C	-0.70585	2.95606	20.55493
H	-0.30167	2.09283	19.98975
H	-0.94533	3.75244	19.82083
H	-1.65838	2.61964	21.01775
C	5.10474	8.59523	22.25855
H	4.31231	8.30074	21.53795
C	6.32409	9.07024	21.45155
H	7.16161	9.39400	22.10578
H	6.05394	9.94031	20.82064
H	6.70575	8.27622	20.77760
C	4.53873	9.69924	23.16279

H	3.63355	9.36470	23.71024
H	4.24265	10.57727	22.55513
H	5.27909	10.06053	23.90736
C	2.78685	6.00750	21.33350
C	1.98883	6.84428	20.52361
H	1.22288	7.50055	20.97118
C	2.17274	6.86730	19.12634
H	1.55157	7.53332	18.50627
C	3.14167	6.04481	18.52647
C	3.93223	5.20287	19.32741
H	4.69009	4.55138	18.86332
C	3.76309	5.18812	20.72668
H	4.39494	4.51695	21.33245
Ni	2.55457	5.99426	23.20099
H	3.28142	6.06018	17.43425
C	1.69324	6.02908	29.31189
O	2.75296	6.48133	30.00558
O	0.63890	5.66055	29.79557
C	2.59611	6.52146	31.44232
H	3.55364	6.89935	31.83914
H	2.38082	5.50804	31.83340
H	1.76249	7.19635	31.71824
H	-0.03719	5.24974	27.43278
H	4.00292	6.81098	27.92587

[($^{i\text{Pr}}$pybox)Ni(p-MeO-C_6H_4)]$^+$ **27**

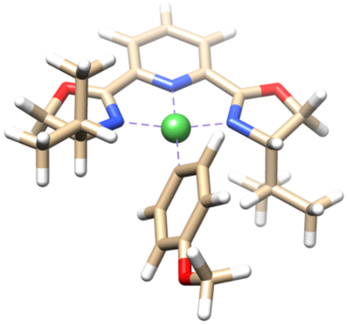

61

O	-0.97223	4.77423	24.70110
O	5.61203	7.27311	25.41845
N	0.74266	5.33413	23.32836
N	2.30981	6.02598	25.14044
N	4.23653	6.69993	23.71061
C	-0.26829	4.76707	22.40205
H	-0.47494	5.51712	21.61196
C	-1.50191	4.59464	23.33767
H	-2.28345	5.36663	23.19276
H	-1.96724	3.59219	23.29552
C	0.26114	5.23480	24.55114
C	1.13341	5.62354	25.66852
C	0.93239	5.62235	27.05956
C	2.00291	6.04564	27.87517
C	3.22881	6.45791	27.31108
C	3.34126	6.43644	25.91032
C	4.43922	6.81170	25.00785
C	6.42958	7.42144	24.19997
H	7.19729	6.62216	24.22991
H	6.92632	8.40766	24.25615
C	5.41992	7.27389	23.02433
H	5.78492	6.53830	22.27878
C	0.23004	3.46941	21.70937
H	1.14646	3.77361	21.15894
C	0.61425	2.36293	22.70245
H	1.40001	2.69652	23.41122
H	1.01629	1.48437	22.16002
H	-0.25486	2.00271	23.29326
C	-0.80546	2.99064	20.67806
H	-0.41736	2.11404	20.12223
H	-1.04164	3.77910	19.93439
H	-1.75805	2.67295	21.15416
C	5.07089	8.58452	22.26670
H	4.26252	8.29332	21.56251
C	6.27609	9.05510	21.43660
H	7.12862	9.37188	22.07461
H	5.99860	9.92893	20.81394
H	6.63911	8.26094	20.75278
C	4.52843	9.68988	23.18359
H	3.63499	9.35777	23.75178
H	4.22139	10.56914	22.58293
H	5.28729	10.04781	23.91109
C	2.73870	5.99785	21.37561
C	1.91164	6.76980	20.53529

H	1.11731	7.40762	20.95911
C	2.08465	6.77379	19.13567
H	1.42707	7.39947	18.51558
C	3.09248	5.97479	18.55040
C	3.92055	5.18714	19.38683
H	4.69624	4.56508	18.91427
C	3.75055	5.20826	20.77738
H	4.41285	4.57773	21.39391
Ni	2.52102	6.01516	23.24282
H	-0.02627	5.29934	27.48868
H	1.87953	6.05464	28.96840
H	4.06922	6.78837	27.93727
O	3.34307	5.89309	17.21893
C	2.56188	6.67104	16.32111
H	2.93904	6.44467	15.30711
H	2.67460	7.76123	16.51585
H	1.48308	6.40247	16.37346

[($^{i\text{Pr}}$pybox)Ni(p-tol)]$^+$ **28**

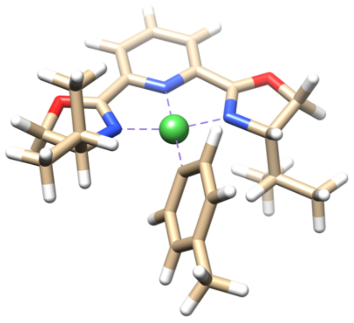

60

O	-0.98424	4.75691	24.58992
O	5.54772	7.33639	25.48321
N	0.77145	5.31361	23.26886
N	2.27108	6.04331	25.12350
N	4.22624	6.73820	23.74228
C	-0.20445	4.72704	22.31721
H	-0.37820	5.45822	21.50168
C	-1.47172	4.57799	23.20958
H	-2.23436	5.36298	23.03504
H	-1.95261	3.58335	23.15678
C	0.25195	5.22163	24.47619
C	1.08628	5.62643	25.61825
C	0.84505	5.62952	27.00312
C	1.88583	6.07345	27.84627
C	3.12122	6.50178	27.31589
C	3.27342	6.47474	25.91848
C	4.39183	6.85987	25.04361
C	6.39842	7.48099	24.28647
H	7.16820	6.68536	24.34355
H	6.88917	8.46979	24.34968
C	5.42211	7.31998	23.08488
H	5.81181	6.58400	22.35206
C	0.32172	3.41239	21.67862
H	1.26791	3.70076	21.17083
C	0.64705	2.32526	22.71426

H	1.40205	2.66684	23.45212
H	1.06577	1.43097	22.21205
H	-0.25257	1.98527	23.27077
C	-0.66167	2.91653	20.60545
H	-0.24999	2.02608	20.08993
H	-0.85524	3.68989	19.83445
H	-1.63869	2.61341	21.03893
C	5.07815	8.62384	22.31390
H	4.28629	8.32253	21.59504
C	6.29497	9.10073	21.50465
H	7.13211	9.42882	22.15724
H	6.02150	9.96819	20.87189
H	6.67812	8.30588	20.83243
C	4.50779	9.72952	23.21392
H	3.60741	9.39216	23.76780
H	4.20228	10.60140	22.60208
H	5.24908	10.10108	23.95289
C	2.80717	5.99521	21.36891
C	2.01434	6.79006	20.51326
H	1.22687	7.44761	20.92053
C	2.23091	6.77826	19.12284
H	1.60889	7.41987	18.47673
C	3.22725	5.96358	18.53983
C	4.00842	5.16753	19.40385
H	4.79219	4.51905	18.97936
C	3.81182	5.18540	20.79898
H	4.45077	4.54446	21.43068
Ni	2.53897	6.02179	23.23070
C	3.43008	5.93684	17.04254
H	3.42221	6.95804	16.60998
H	2.61916	5.36852	16.53768
H	4.38882	5.45613	16.76654
H	3.93801	6.84829	27.96447
H	-0.12064	5.29544	27.40689
H	1.73116	6.08627	28.93571

[($^{i\text{Pr}}$pybox)Ni(p-CHO-C_6H_4)]$^+$ **29**

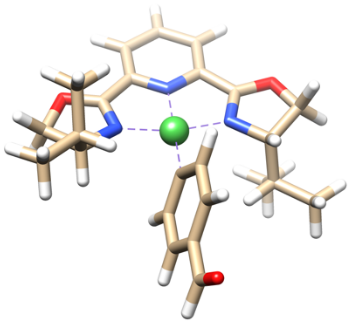

59

O	-0.97872	4.75726	24.50464
O	5.53814	7.36017	25.41266
N	0.79493	5.27063	23.19273
N	2.27399	6.04286	25.04767
N	4.23882	6.72521	23.66905
C	-0.17453	4.66869	22.24303
H	-0.32728	5.37715	21.40384

C	-1.45510	4.56257	23.12129	H	4.33130	8.26582	21.47711
H	-2.19829	5.36044	22.92437	C	6.33366	9.06092	21.42239
H	-1.95516	3.57747	23.08085	H	7.14885	9.41371	22.08933
C	0.26112	5.20777	24.39568	H	6.06567	9.91142	20.76511
C	1.08509	5.63470	25.53831	H	6.74470	8.25794	20.77718
C	0.82864	5.66921	26.92006	C	4.49834	9.70085	23.07575
C	1.85899	6.13527	27.76334	H	3.58302	9.36634	23.60617
C	3.09807	6.55685	27.23626	H	4.20785	10.56119	22.44070
C	3.26524	6.49783	25.84187	H	5.21330	10.08926	23.83178
C	4.39071	6.87106	24.96952	C	2.82980	5.94072	21.30080
C	6.40129	7.49317	24.22328	C	2.06344	6.77918	20.45455
H	7.17994	6.70875	24.30474	H	1.31106	7.46780	20.87612
H	6.87837	8.48903	24.27399	C	2.26092	6.75787	19.06643
C	5.44057	7.29942	23.01346	H	1.68209	7.40805	18.39065
H	5.84498	6.54869	22.30351	C	3.21839	5.88909	18.49729
C	0.34310	3.32735	21.65431	C	3.97814	5.04893	19.33714
H	1.30608	3.58337	21.16071	H	4.72305	4.36620	18.89287
C	0.62452	2.26451	22.72723	C	3.79172	5.07669	20.73112
H	1.36261	2.61408	23.47849	H	4.39694	4.40738	21.36537
H	1.04474	1.35234	22.25948	Ni	2.55920	5.98615	23.15803
H	-0.29539	1.94958	23.26471	C	3.41517	5.86713	17.01970
C	-0.62491	2.81816	20.57324	O	2.79319	6.56364	16.23569
H	-0.21869	1.90531	20.09466	H	4.20375	5.13860	16.66182
H	-0.78656	3.57105	19.77514	H	-0.14046	5.34215	27.32150
H	-1.61658	2.54612	20.99327	H	3.90610	6.92245	27.88512
C	5.10224	8.58542	22.21042	H	1.69253	6.17251	28.85030

[(^{iPr}pybox)Ni(p-COMe-C_6H_4)]$^+$ **30**

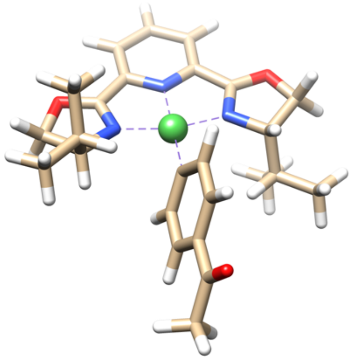

62

O	-0.98144	4.77366	24.75048
O	5.60563	7.30295	25.27783
N	0.71161	5.28098	23.33238
N	2.30612	6.03470	25.09925
N	4.19799	6.68961	23.61196
C	-0.32734	4.71537	22.43531
H	-0.54889	5.46512	21.64851
C	-1.53895	4.55505	23.40264
H	-2.33207	5.31448	23.25717
H	-1.99296	3.54668	23.39665
C	0.25170	5.21731	24.56512
C	1.14377	5.63777	25.65765
C	0.96608	5.67619	27.05161
C	2.04638	6.13425	27.83461
C	3.25799	6.54161	27.23741
C	3.34590	6.47869	25.83597
C	4.42553	6.83458	24.90141
C	6.40185	7.41758	24.04109
H	7.16900	6.61845	24.08015
H	6.89987	8.40427	24.06140
C	5.37078	7.24009	22.88830
H	5.71925	6.48092	22.15841
C	0.14218	3.41158	21.73465
H	1.04622	3.70467	21.15788
C	0.54362	2.30579	22.72160
H	1.34835	2.63555	23.41075
H	0.92552	1.42235	22.17261
H	-0.31302	1.95361	23.33472
C	-0.92425	2.93901	20.73189
H	-0.55804	2.05895	20.16691
H	-1.17425	3.72792	19.99342
H	-1.86597	2.62874	21.23375
C	5.01311	8.53098	22.10138
H	4.19441	8.22652	21.41456
C	6.20784	8.97743	21.24248
H	7.06702	9.31578	21.86044
H	5.92088	9.83091	20.59685
H	6.56455	8.16335	20.57892
C	4.48730	9.66186	22.99677
H	3.60038	9.35002	23.58618
H	4.17658	10.52659	22.37757
H	5.25767	10.03551	23.70369

C	2.66495	5.91314	21.32818
C	1.86736	6.73892	20.50175
H	1.13141	7.43442	20.94074
C	2.01288	6.69814	19.10692
H	1.40891	7.33939	18.44562
C	2.94526	5.82690	18.50053
C	3.73544	4.99889	19.32707
H	4.46752	4.30622	18.88368
C	3.60276	5.04541	20.72779
H	4.23424	4.38242	21.34276
Ni	2.48364	5.97228	23.19674
H	0.01793	5.35844	27.50708
H	1.94165	6.17581	28.92914
H	4.10519	6.89963	27.83876
C	3.03921	5.83538	16.99119
C	4.03293	4.90527	16.31614
H	5.07116	5.11928	16.64716
H	3.96462	5.04250	15.22139
H	3.82452	3.84344	16.56712
O	2.32009	6.58410	16.33832

[(^{iPr}pybox)Ni(p-CF_3-C_6H_4)]$^+$ **31**

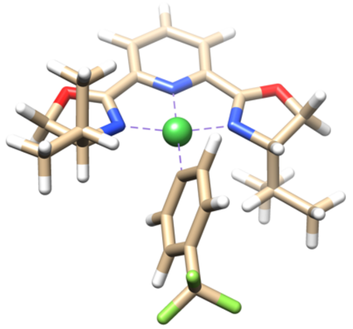

60

O	-1.00512	4.84592	24.48434
O	5.54064	7.31865	25.54121
N	0.80826	5.32509	23.21422
N	2.25401	6.08154	25.10066
N	4.26633	6.71456	23.76820
C	-0.14285	4.72291	22.24647
H	-0.26240	5.41982	21.39226
C	-1.44831	4.64785	23.09056
H	-2.17081	5.45779	22.86712
H	-1.96520	3.67186	23.04454
C	0.24351	5.27843	24.40380
C	1.04670	5.69640	25.56438
C	0.76007	5.73630	26.94004
C	1.78065	6.18120	27.80669
C	3.04022	6.57587	27.30804
C	3.23701	6.51417	25.91753
C	4.39128	6.85926	25.07165
C	6.43400	7.42868	24.37147

H	7.19156	6.62599	24.47194
H	6.93367	8.41305	24.43190
C	5.49732	7.25585	23.13970
H	5.89921	6.49292	22.44142
C	0.37210	3.36542	21.69338
H	1.34951	3.60141	21.21883
C	0.61399	2.31704	22.78984
H	1.33757	2.66997	23.55349
H	1.03509	1.39289	22.34715
H	-0.32288	2.02114	23.30862
C	-0.57519	2.84890	20.59755
H	-0.16518	1.92736	20.13935
H	-0.71444	3.59200	19.78600
H	-1.57755	2.58831	20.99947
C	5.21208	8.54780	22.32634
H	4.45046	8.24624	21.57581
C	6.47451	8.98792	21.56664
H	7.28609	9.31046	22.25321
H	6.24651	9.85025	20.90933
H	6.87361	8.17553	20.92561
C	4.61907	9.68162	23.17524
H	3.68134	9.37593	23.68321
H	4.37060	10.54904	22.53222
H	5.32609	10.05045	23.94845
Ni	2.58419	6.01100	23.21979
H	-0.22399	5.42795	27.31915
H	3.84171	6.92270	27.97513
H	1.59075	6.22163	28.88969
F	3.48268	6.96330	16.50355
C	2.19438	6.77225	20.48436
C	2.44169	6.72521	19.10068
C	3.86044	5.03762	20.84287
C	2.90613	5.93414	21.37042
C	4.09551	4.97750	19.45701
C	3.38672	5.82053	18.58220
C	3.62482	5.74879	17.08744
H	1.88715	7.38833	18.41910
F	2.73975	4.91079	16.48849
H	4.42992	4.36314	21.50342
H	4.83179	4.26553	19.05283
H	1.44555	7.48971	20.86103
F	4.86714	5.29367	16.79990

[(^{iPr}pybox)Ni(p-CN-C_6H_4)]$^+$ **32**

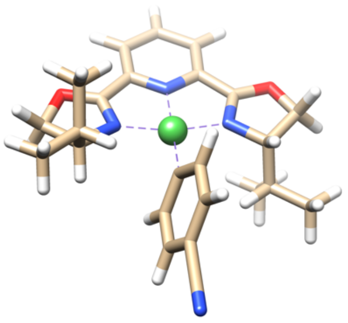

O	-0.94210	4.66778	24.83386
O	5.60280	7.30931	25.30689
N	0.71400	5.24254	23.39851
N	2.32409	5.98863	25.15402
N	4.17855	6.71203	23.64945
C	-0.32666	4.66867	22.50788
H	-0.57686	5.42495	21.73625
C	-1.51914	4.46787	23.49110
H	-2.32717	5.21668	23.37537
H	-1.95617	3.45229	23.47173
C	0.27713	5.14193	24.63725
C	1.17993	5.55644	25.72324
C	1.02611	5.56007	27.12061
C	2.11095	6.02210	27.89515
C	3.30370	6.46653	27.28652
C	3.36800	6.43595	25.88254
C	4.42550	6.83118	24.93818
C	6.37852	7.46721	24.06145
H	7.16469	6.68621	24.07371
H	6.85328	8.46487	24.09592
C	5.33494	7.28991	22.91923
H	5.68636	6.54499	22.17616
C	0.15920	3.38511	21.78096
H	1.04994	3.70436	21.19758
C	0.59443	2.27188	22.74457
H	1.39569	2.60690	23.43541
H	0.99384	1.40860	22.17631
H	-0.24965	1.88786	23.35559
C	-0.91067	2.90722	20.78463
H	-0.53636	2.04089	20.20386
H	-1.18308	3.70078	20.05920
H	-1.84094	2.57505	21.29363
C	4.95354	8.58638	22.15315
H	4.13817	8.27988	21.46332
C	6.13870	9.06487	21.29834
H	6.99259	9.41018	21.91946
H	5.83638	9.92181	20.66432
H	6.50922	8.26657	20.62351
C	4.41078	9.69383	23.06722
H	3.53053	9.35711	23.65288
H	4.08452	10.56317	22.46268
H	5.17603	10.06913	23.77871
C	2.60999	5.96529	21.37598
C	1.79974	6.81596	20.59057
H	1.07749	7.50254	21.06354
C	1.90755	6.82032	19.19044
H	1.28055	7.49318	18.58616
C	2.82575	5.95540	18.54679
C	3.63564	5.09597	19.32889
H	4.34626	4.41909	18.83094
C	3.53055	5.11011	20.72916
H	4.17280	4.42811	21.31063

Ni	2.46837	5.97429	23.24933
C	2.93502	5.94925	17.11442
H	4.15443	6.82718	27.88151
H	2.02510	6.03695	28.99218
H	0.09274	5.21291	27.58501
N	3.02467	5.94325	15.94350

[(4-Pyrrolidinyl-^{iPr}pybox)Ni(p-MeO- C_6H_4)]$^+$ **33**

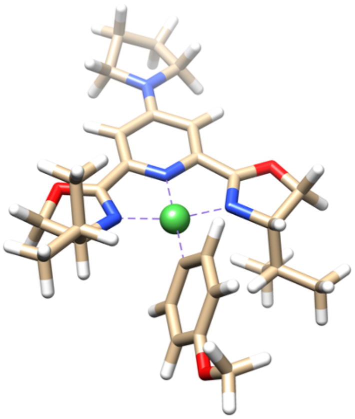

73

O	-0.90050	4.59485	24.70834
O	5.69395	7.05701	25.42737
N	0.78974	5.22492	23.33468
N	2.38818	5.82833	25.13666
N	4.28358	6.57612	23.71866
C	-0.23977	4.71139	22.39966
H	-0.46214	5.50553	21.65832
C	-1.45399	4.48295	23.34874
H	-2.24245	5.25645	23.25649
H	-1.91607	3.48145	23.26303
C	0.33283	5.06413	24.56056
C	1.22776	5.39974	25.67968
C	1.03679	5.33403	27.05894
C	2.11934	5.73156	27.92292
C	3.34608	6.17523	27.31087
C	3.42168	6.20849	25.91936
C	4.50758	6.62368	25.01659
C	6.49109	7.25737	24.20506
H	7.25937	6.45824	24.18669
H	6.99020	8.24026	24.29524
C	5.46436	7.16112	23.03892
H	5.81130	6.44723	22.26402
C	0.24726	3.45885	21.62171
H	1.14896	3.80132	21.06919
C	0.66331	2.30184	22.54114
C	-0.80948	3.03093	20.58993
H	-0.43021	2.19244	19.97219
H	-1.07186	3.85948	19.90036
H	-1.74705	2.67780	21.07106
C	5.12286	8.50037	22.33054
H	4.30554	8.24147	21.62446
C	6.32552	8.98834	21.50689
H	7.18961	9.26540	22.14807
H	6.05510	9.89096	20.92328
H	6.66913	8.21780	20.78676
C	4.59887	9.57698	23.29146

H	3.70605	9.23063	23.85162
H	4.29757	10.48202	22.72722
H	5.36654	9.89751	24.02694
C	2.72974	6.01606	21.36733
C	1.91555	6.88434	20.61219
H	1.16410	7.52207	21.10910
C	2.04212	6.98530	19.21025
H	1.39341	7.68354	18.66203
C	2.99337	6.19133	18.53277
C	3.80976	5.30872	19.27942
H	4.54240	4.69233	18.73619
C	3.68232	5.23088	20.67353
H	4.33646	4.52914	21.21865
Ni	2.56213	5.91664	23.23859
N	1.98462	5.69297	29.27216
C	0.78434	5.18748	29.96544
C	3.00805	6.17127	30.22084
C	2.25821	6.23602	31.56059
H	3.40525	7.15812	29.90543
H	3.86417	5.45845	30.25901
C	1.21842	5.10970	31.43778
H	1.75330	7.21901	31.66284
H	2.93609	6.11678	32.42695
H	0.48052	4.20146	29.55677
H	-0.06741	5.89147	29.82132
H	0.36385	5.22217	32.13179
H	1.68669	4.12431	31.64340
H	1.06476	1.46054	21.94176
H	-0.19038	1.89890	23.12704
H	1.45855	2.60695	23.25187
O	3.19888	6.20036	17.18804
H	2.75628	6.91574	15.34037
C	2.41882	7.07077	16.38184
H	2.57284	8.13880	16.65629
H	1.33257	6.83466	16.44824
H	0.07482	4.99505	27.46119
H	4.21127	6.47988	27.91152

[(4-CF_3-$^{i\text{Pr}}$pybox)Ni(*p*-tol)]$^+$ **34**

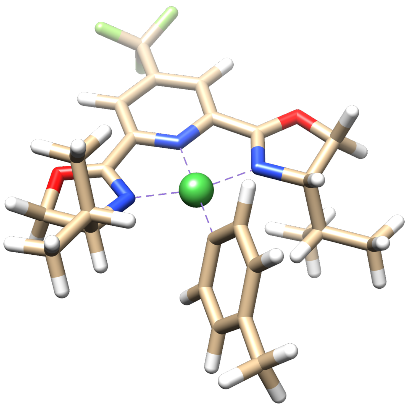

63

O	-0.92087	4.66558	24.57362
O	5.60798	7.23846	25.48531
N	0.81170	5.28172	23.24787

N	2.33451	5.94580	25.10873	H	-0.26212	2.17289	19.90477
N	4.27209	6.70211	23.73458	H	-0.91445	3.83256	19.77458
C	-0.17950	4.73825	22.28585	H	-1.62169	2.66830	20.94429
H	-0.38394	5.51711	21.52348	C	5.12617	8.62379	22.34972
C	-1.42477	4.51311	23.19581	H	4.34247	8.34183	21.61484
H	-2.22129	5.27110	23.06067	C	6.35109	9.12446	21.56704
H	-1.86671	3.50190	23.12015	H	7.18118	9.43287	22.23766
C	0.30864	5.14172	24.45757	H	6.08364	10.01050	20.95753
C	1.15595	5.51041	25.60207	H	6.74217	8.35046	20.87524
C	0.92458	5.47232	26.98633	C	4.54536	9.70194	23.27537
C	1.97308	5.89253	27.83398	H	3.63680	9.34857	23.80501
C	3.20102	6.34822	27.30519	H	4.24937	10.59285	22.68673
C	3.34052	6.35953	25.90828	H	5.27614	10.04948	24.03576
C	4.44954	6.77922	25.03778	C	2.81788	6.05803	21.35126
C	6.44963	7.42963	24.28831	C	2.01122	6.89278	20.55100
H	7.22736	6.64038	24.31558	H	1.23130	7.52977	21.00213
H	6.93086	8.42072	24.38150	C	2.19895	6.94077	19.15549
C	5.46673	7.29968	23.08728	H	1.56249	7.60760	18.55035
H	5.85173	6.58058	22.33546	C	3.17853	6.15052	18.51728
C	0.34645	3.47588	21.54966	C	3.97243	5.30942	19.32933
H	1.26350	3.81574	21.02161	H	4.74049	4.67149	18.86069
C	0.73824	2.33950	22.50518	C	3.80594	5.26727	20.72608
H	1.51293	2.65830	23.23273	H	4.44816	4.59119	21.31538
H	1.15773	1.48699	21.93502	Ni	2.57859	5.99867	23.21581
H	-0.13074	1.94554	23.07431	C	1.79546	5.80704	29.34708
C	-0.67087	3.02051	20.49002	F	2.51004	6.76502	29.97204

F	2.22176	4.60629	29.79534
F	0.49897	5.94646	29.69023
C	3.38811	6.21037	17.02216
H	2.54635	6.71443	16.50841
H	3.50130	5.19811	16.58386
H	4.31309	6.77306	16.77010
H	-0.03644	5.13087	27.39408
H	4.01393	6.68858	27.96106

[(4-OMe-$^{i\text{Pr}}$pybox)Ni(p-CF_3-C_6H_4)]$^+$ **35**

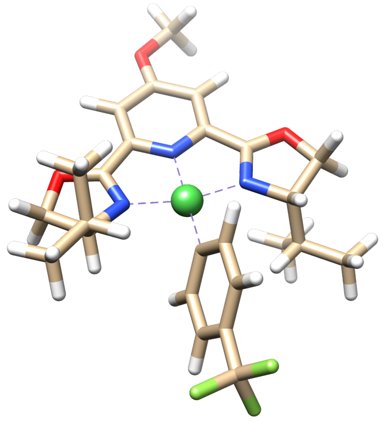

64

O	-0.99550	4.67243	24.55212
O	5.50678	7.26962	25.55396
N	0.75765	5.28363	23.25122
N	2.23932	5.97676	25.12791
N	4.19845	6.72443	23.78618
C	-0.21736	4.73449	22.27627
H	-0.41453	5.51078	21.50910
C	-1.47548	4.50676	23.16767
H	-2.27503	5.25772	23.01316
H	-1.90874	3.49171	23.09137
C	0.23567	5.15205	24.45385
C	1.06198	5.53727	25.61046
C	0.79574	5.50031	26.98670
C	1.82741	5.93991	27.86503
C	3.07287	6.39403	27.33394
C	3.22851	6.39826	25.94803
C	4.35393	6.81030	25.09209
C	6.36911	7.44798	24.37024
H	7.14315	6.65598	24.41655
H	6.85294	8.43779	24.46354
C	5.40621	7.31226	23.15416
H	5.80236	6.58592	22.41505
C	0.32139	3.47170	21.55065
H	1.24464	3.81121	21.03358
C	0.70483	2.33974	22.51497
H	1.47108	2.66353	23.24923
H	1.13095	1.48527	21.95271
H	-0.16985	1.94739	23.07663
C	-0.67939	3.00947	20.47830
H	-0.25889	2.16256	19.90052
H	-0.91699	3.81849	19.75742
H	-1.63464	2.65431	20.92074

C	5.08494	8.63241	22.40185
H	4.31266	8.35065	21.65489
C	6.32497	9.12319	21.63659
H	7.14534	9.42919	22.32013
H	6.07246	10.00803	21.01914
H	6.72309	8.34459	20.95409
C	4.49216	9.71932	23.30982
H	3.57405	9.37249	23.82706
H	4.20967	10.60683	22.70954
H	5.21140	10.07016	24.07983
C	2.79474	6.05094	21.38060
C	2.04128	6.91895	20.56080
H	1.28228	7.59064	20.99685
C	2.24974	6.95545	19.16943
H	1.66095	7.63786	18.53860
C	3.20724	6.11260	18.57834
C	3.95748	5.23548	19.38525
H	4.69791	4.56452	18.92262
C	3.75741	5.21094	20.77649
H	4.35619	4.51094	21.38266
Ni	2.51979	6.01144	23.23831
C	3.47097	6.16512	17.08768
F	2.45933	6.76452	16.41602
F	3.63139	4.92168	16.57053
F	4.60589	6.85836	16.81291
O	1.72492	5.96539	29.19672
C	0.51105	5.53927	29.83246
H	0.67882	5.66722	30.91644
H	0.30104	4.47141	29.61424
H	-0.34631	6.16906	29.51534
H	3.86625	6.72946	28.01531
H	-0.17489	5.14264	27.35109

[(4-CF_3-$^{i\mathrm{Pr}}$pybox)Ni(*p*-CF_3- C_6H_4)]$^+$ **36**

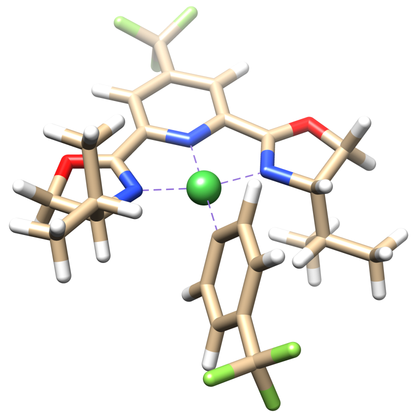

63

O	-0.91628	4.65670	24.58261
O	5.60496	7.24681	25.48418
N	0.81433	5.26924	23.25383
N	2.33610	5.94375	25.11329
N	4.26575	6.71525	23.73544
C	-0.18073	4.73050	22.29220
H	-0.38879	5.51353	21.53496
C	-1.42348	4.50173	23.20541

H	-2.22310	5.25662	23.07257
H	-1.86143	3.48873	23.13199
C	0.31255	5.12993	24.46419
C	1.16118	5.50167	25.60769
C	0.93119	5.46363	26.99217
C	1.97818	5.89053	27.83823
C	3.20249	6.35327	27.30773
C	3.34010	6.36464	25.91056
C	4.44685	6.78955	25.03837
C	6.44488	7.44368	24.28576
H	7.22625	6.65826	24.31167
H	6.92093	8.43702	24.38040
C	5.46052	7.31163	23.08564
H	5.84583	6.59031	22.33594
C	0.33930	3.47059	21.54777
H	1.25343	3.80973	21.01410
C	0.73623	2.33020	22.49646
H	1.51443	2.64568	23.22177
H	1.15240	1.48019	21.92032
H	-0.12992	1.93378	23.06803
C	-0.68519	3.02084	20.49244
H	-0.28050	2.17640	19.89996
H	-0.93368	3.83611	19.78241
H	-1.63289	2.66645	20.95131
C	5.12273	8.63528	22.34624
H	4.33911	8.35580	21.61035
C	6.34904	9.13208	21.56285
H	7.18086	9.43590	22.23325
H	6.08432	10.02003	20.95514
H	6.73621	8.35826	20.86879
C	4.54334	9.71564	23.27037
H	3.63573	9.36429	23.80318
H	4.24688	10.60498	22.67972
H	5.27569	10.06544	24.02813
C	2.81366	6.05854	21.35735
C	2.03726	6.92736	20.56186
H	1.28223	7.59000	21.01747
C	2.22162	6.97584	19.16728
H	1.61694	7.65911	18.55265
C	3.17413	6.14398	18.55330
C	3.94511	5.26563	19.33891
H	4.68124	4.60316	18.85782
C	3.77202	5.22943	20.73362
H	4.38690	4.52987	21.32371
Ni	2.57653	5.99855	23.22137
C	1.80178	5.80526	29.35239
F	2.51914	6.76147	29.97575
F	2.22577	4.60330	29.79791
F	0.50596	5.94764	29.69515
C	3.41078	6.20966	17.05751
F	2.38302	6.80679	16.41018
F	3.57032	4.97123	16.52998

F	4.53566	6.91307	16.77063
H	-0.02778	5.11739	27.40093
H	4.01426	6.69903	27.96230

(dtbbpy)Ni(*p*-CF_3-*o*-tol)Br **S1**

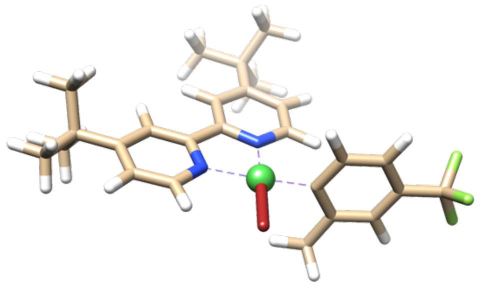

63

C	4.27768	2.84833	5.71325
Ni	4.34023	5.60686	6.70892
N	4.44807	4.17478	5.46151
Br	4.20277	7.36247	8.19385
C	4.38180	1.86465	4.73229
N	4.73686	6.70368	5.10754
C	4.68241	2.20129	3.39044
C	4.85420	3.57290	3.13717
C	4.73167	4.52903	4.16207
C	4.88961	5.97788	3.95896
C	5.16505	6.59563	2.72729
C	5.29914	7.99702	2.63271
C	5.14158	8.71580	3.83767
C	4.86417	8.04350	5.03442
C	4.01016	4.47854	8.18238
C	5.12238	3.93736	8.86829
C	4.96031	3.00014	9.89770
C	3.66202	2.59387	10.26780
C	2.55077	3.14452	9.60947
C	2.70254	4.09301	8.57295
H	6.14225	4.25495	8.59785
H	5.83942	2.58845	10.41701
C	3.49422	1.58254	11.37247
C	1.46896	4.67136	7.91056
H	1.53916	2.83417	9.91377
C	5.59573	8.66789	1.27923
C	5.71277	10.20007	1.40896
H	4.77230	10.65983	1.77505
H	5.93237	10.64407	0.41697
H	6.53315	10.49645	2.09413
C	6.93236	8.11671	0.71801
H	7.77558	8.33536	1.40439
H	7.15796	8.58608	-0.26176
H	6.89670	7.01986	0.56061
C	4.44543	8.34218	0.29122
H	3.47263	8.71497	0.67166
H	4.34401	7.25231	0.11328
H	4.63603	8.82438	-0.68964
C	4.81007	1.11371	2.31092
C	5.10918	1.71036	0.92052
H	4.30570	2.39770	0.58334

H	5.18466	0.89748	0.16996
H	6.07125	2.26269	0.90377
C	5.96641	0.15576	2.69967
H	5.79021	-0.33290	3.67906
H	6.93170	0.69881	2.76227
H	6.07410	-0.64722	1.94068
C	3.48111	0.31824	2.23131
H	2.63541	0.97842	1.94914
H	3.22441	-0.16350	3.19613
H	3.56118	-0.48474	1.46923
H	4.73100	8.57299	5.99443
H	5.23186	9.80992	3.86807
H	5.27576	5.97128	1.82939
H	5.09056	3.92333	2.12489
H	4.04996	2.58707	6.75570
H	4.22412	0.81928	5.03543
F	2.19420	1.25975	11.59532
F	4.00161	2.02900	12.55433
F	4.14993	0.41840	11.09478
H	1.74603	5.41342	7.13668
H	0.82544	5.19068	8.65215
H	0.84806	3.88083	7.43597

(^{CF3}bpy)Ni(*p*-CF_3-*o*-tol)Br **S9**

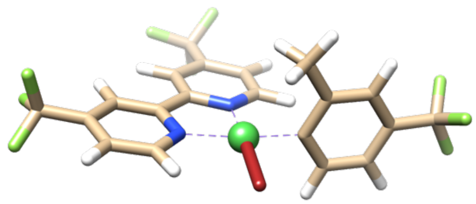

45

C	4.15844	2.83663	5.75649
Ni	4.30089	5.60128	6.71555
N	4.39963	4.15050	5.48910
Br	3.96744	7.37368	8.13465
C	4.19928	1.83957	4.78307
N	4.74214	6.67645	5.10599
C	4.50178	2.18854	3.45129
C	4.75293	3.53372	3.16013
C	4.69659	4.49168	4.18588
C	4.93183	5.92652	3.97977
C	5.31539	6.51033	2.75817
C	5.50621	7.89636	2.69690
C	5.30173	8.66395	3.85988
C	4.92275	8.01385	5.03871
C	4.05359	4.49455	8.22342
C	5.19477	3.98108	8.89790
C	5.00330	3.04195	9.92997
C	3.71300	2.62446	10.31277
C	2.58835	3.16214	9.66595
C	2.76525	4.09958	8.63240
C	6.58783	4.42849	8.51505

H	5.88320	2.63254	10.45158
C	3.57075	1.61610	11.42593
H	1.87436	4.52754	8.14590
H	1.57797	2.85626	9.97319
C	5.97111	8.55643	1.41260
F	5.36449	9.75144	1.22874
F	7.30724	8.77946	1.44068
F	5.70946	7.78394	0.33155
C	4.51754	1.13293	2.36537
F	5.05376	1.60419	1.21458
F	5.23651	0.05105	2.74665
F	3.26249	0.70451	2.08667
H	3.92577	2.59943	6.80379
H	3.99675	0.79791	5.06945
H	4.99469	3.83514	2.13286
H	5.46295	5.89644	1.85993
H	4.74144	8.55304	5.98518
H	5.42958	9.75571	3.85144
F	2.28036	1.25575	11.63838
F	4.05405	2.08988	12.60595
F	4.26631	0.47448	11.15897
H	6.66295	5.53607	8.54111
H	6.84887	4.11015	7.48287
H	7.35473	4.01286	9.19828

$[Ni(Ph)]^+$ **S10**

12

C	2.82439	5.99566	21.34699
C	2.06584	6.80007	20.46956
H	1.29662	7.49073	20.85707
C	2.29436	6.74823	19.07941
H	1.70357	7.39071	18.40682
C	3.26796	5.88206	18.55406
H	3.44239	5.83928	17.46767
C	4.01878	5.07128	19.42240
H	4.78020	4.38536	19.01707
C	3.80536	5.13104	20.81437
H	4.40713	4.48312	21.47393
Ni	2.53799	6.07612	23.20404

$[Ni(p\text{-}MeO\text{-}C_6H_4)]^+$ **S11**

16

C	2.73870	5.99785	21.37561
C	1.91164	6.76980	20.53529
H	1.11731	7.40762	20.95911
C	2.08465	6.77379	19.13567
H	1.42707	7.39947	18.51558
C	3.09248	5.97479	18.55040
C	3.92055	5.18714	19.38683
H	4.69624	4.56508	18.91427
C	3.75055	5.20826	20.77738
H	4.41285	4.57773	21.39391

Ni	2.52102	6.01516	23.24282
O	3.34307	5.89309	17.21893
C	2.56188	6.67104	16.32111
H	2.93904	6.44467	15.30711
H	2.67460	7.76123	16.51585
H	1.48308	6.40247	16.37346

[Ni(*p*-tol)]$^{+}$ **S12**

15

C	2.80717	5.99521	21.36891
C	2.01434	6.79006	20.51326
H	1.22687	7.44761	20.92053
C	2.23091	6.77826	19.12284
H	1.60889	7.41987	18.47673
C	3.22725	5.96358	18.53983
C	4.00842	5.16753	19.40385
H	4.79219	4.51905	18.97936
C	3.81182	5.18540	20.79898
H	4.45077	4.54446	21.43068
Ni	2.53897	6.02179	23.23070
C	3.43008	5.93684	17.04254
H	3.42221	6.95804	16.60998
H	2.61916	5.36852	16.53768
H	4.38882	5.45613	16.76654

[Ni(*p*-CHO-C_6H_4)]$^{+}$ **S13**

14

C	2.82980	5.94072	21.30080
C	2.06344	6.77918	20.45455
H	1.31106	7.46780	20.87612
C	2.26092	6.75787	19.06643
H	1.68209	7.40805	18.39065
C	3.21839	5.88909	18.49729
C	3.97814	5.04893	19.33714
H	4.72305	4.36620	18.89287
C	3.79172	5.07669	20.73112
H	4.39694	4.40738	21.36537
Ni	2.55920	5.98615	23.15803
C	3.41517	5.86713	17.01970
O	2.79319	6.56364	16.23569
H	4.20375	5.13860	16.66182

[Ni(*p*-COMe-C_6H_4)]$^{+}$ **S14**

17

C	2.66495	5.91314	21.32818
C	1.86736	6.73892	20.50175
H	1.13141	7.43442	20.94074
C	2.01288	6.69814	19.10692
H	1.40891	7.33939	18.44562
C	2.94526	5.82690	18.50053
C	3.73544	4.99889	19.32707
H	4.46752	4.30622	18.88368

C	3.60276	5.04541	20.72779
H	4.23424	4.38242	21.34276
Ni	2.48364	5.97228	23.19674
C	3.03921	5.83538	16.99119
C	4.03293	4.90527	16.31614
H	5.07116	5.11928	16.64716
H	3.96462	5.04250	15.22139
H	3.82452	3.84344	16.56712
O	2.32009	6.58410	16.33832

[Ni(*p*-CF_3-C_6H_4)]$^+$ **S15**

15

Ni	2.58419	6.01100	23.21979
F	3.48268	6.96330	16.50355
C	2.19438	6.77225	20.48436
C	2.44169	6.72521	19.10068
C	3.86044	5.03762	20.84287
C	2.90613	5.93414	21.37042
C	4.09551	4.97750	19.45701
C	3.38672	5.82053	18.58220
C	3.62482	5.74879	17.08744
H	1.88715	7.38833	18.41910
F	2.73975	4.91079	16.48849
H	4.42992	4.36314	21.50342
H	4.83179	4.26553	19.05283
H	1.44555	7.48971	20.86103
F	4.86714	5.29367	16.79990

[Ni(*p*-CN-C_6H_4)]$^+$ **S16**

13

C	2.60999	5.96529	21.37598
C	1.79974	6.81596	20.59057
H	1.07749	7.50254	21.06354
C	1.90755	6.82032	19.19044
H	1.28055	7.49318	18.58616
C	2.82575	5.95540	18.54679
C	3.63564	5.09597	19.32889
H	4.34626	4.41909	18.83094
C	3.53055	5.11011	20.72916
H	4.17280	4.42811	21.31063
Ni	2.46837	5.97429	23.24933
C	2.93502	5.94925	17.11442
N	3.02467	5.94325	15.94350

Supporting Ligands

^{iPr}pybox **L1**

45

O	-1.01394	4.89002	24.55348
O	5.52051	7.45647	25.45150
N	0.76763	5.37875	23.24172
N	2.24643	6.16024	25.09193
N	4.22269	6.80514	23.71243

C	-0.20020	4.76846	22.29713
H	-0.34424	5.46489	21.44633
C	-1.48527	4.68138	23.17119
H	-2.22233	5.48158	22.96040
H	-1.99152	3.69915	23.14019
C	0.22963	5.33357	24.44354
C	1.05249	5.76798	25.58373
C	0.79277	5.81970	26.96455
C	1.82487	6.28580	27.80571
C	3.06919	6.69014	27.27772
C	3.24009	6.61436	25.88435
C	4.37076	6.96784	25.01102
C	6.38712	7.56965	24.26332
H	7.16391	6.78488	24.35823
H	6.86680	8.56477	24.30056
C	5.43026	7.36065	23.05311
H	5.83236	6.59591	22.35694
C	0.31429	3.41685	21.73025
H	1.27981	3.66273	21.23663
C	0.58572	2.36773	22.81921
H	1.31763	2.72658	23.57236
H	1.01045	1.44995	22.36644
H	-0.34001	2.06032	23.35164
C	-0.65111	2.89548	20.65293
H	-0.24658	1.97493	20.18794
H	-0.80820	3.63813	19.84433
H	-1.64504	2.63084	21.07330
C	5.10271	8.63423	22.22622
H	4.33693	8.30396	21.49234
C	6.34232	9.09264	21.44075
H	7.15444	9.45141	22.10842
H	6.08291	9.93465	20.76867
H	6.75375	8.27754	20.81052
C	4.49576	9.76564	23.06832
H	3.57128	9.44464	23.59010
H	4.22067	10.61930	22.41796
H	5.20268	10.15934	23.82901
H	-0.17995	5.50419	27.36666
H	3.87912	7.05523	27.92448
H	1.65590	6.33612	28.89174

4-Pyrrolidinyl-$^{i\mathrm{Pr}}$pybox **L3**

57

O	-0.89316	4.60063	24.70438
O	5.69362	7.07454	25.44333
N	0.79952	5.23185	23.33501
N	2.39339	5.83479	25.14285
N	4.28818	6.59770	23.73007
C	-0.23243	4.72869	22.39638
H	-0.45148	5.52876	21.66043
C	-1.44770	4.49990	23.34389
H	-2.23211	5.27804	23.25720

H	-1.91468	3.50133	23.25220
C	0.34107	5.06671	24.55960
C	1.23440	5.39867	25.68200
C	1.04131	5.32622	27.06040
C	2.12070	5.72579	27.92786
C	3.34571	6.17963	27.31932
C	3.42310	6.21833	25.92828
C	4.50934	6.64100	25.02848
C	6.49471	7.27752	24.22388
H	7.26320	6.47863	24.20670
H	6.99330	8.26032	24.31752
C	5.47143	7.18330	23.05447
H	5.81981	6.46936	22.28018
C	0.24863	3.47868	21.61075
H	1.15348	3.81854	21.06171
C	0.65611	2.31307	22.52332
C	-0.80886	3.06476	20.57398
H	-0.43431	2.22748	19.95176
H	-1.06399	3.89938	19.88901
H	-1.74974	2.71542	21.05116
C	5.13325	8.52375	22.34701
H	4.31718	8.26812	21.63834
C	6.33810	9.01084	21.52612
H	7.20222	9.28265	22.16931
H	6.07067	9.91625	20.94559
H	6.68014	8.24202	20.80330
C	4.60925	9.60022	23.30820
H	3.71692	9.25387	23.86936
H	4.30713	10.50485	22.74378
H	5.37750	9.92128	24.04288
N	1.98466	5.68003	29.27626
C	0.78743	5.16186	29.96588
C	3.00480	6.15858	30.22872
C	2.25403	6.20968	31.56849
H	3.39562	7.15001	29.91995
H	3.86519	5.45086	30.26223
C	1.22135	5.07778	31.43787
H	1.74345	7.18896	31.67754
H	2.93239	6.08849	32.43413
H	0.49117	4.17640	29.55059
H	-0.06886	5.86064	29.82528
H	0.36581	5.18077	32.13205
H	1.69552	4.09408	31.63762
H	4.20801	6.48741	27.92261
H	0.08001	4.98097	27.45964
H	1.05408	1.47352	21.91929
H	-0.20104	1.91094	23.10450
H	1.45127	2.60890	23.23794

4-NMe_2-$^{i\text{Pr}}$pybox **L4**

53

O	-0.95434	4.60534	24.57720

O	5.59338	7.12562	25.47538	H	-0.17327	1.88819	23.04315
N	0.77713	5.21874	23.24832	C	-0.72834	2.99242	20.47525
N	2.30917	5.86764	25.09295	H	-0.32443	2.15146	19.87691
N	4.24514	6.61034	23.72744	H	-0.97604	3.81287	19.77077
C	-0.22173	4.69029	22.28787	H	-1.67674	2.63621	20.93185
H	-0.42848	5.47787	21.53489	C	5.12344	8.52217	22.34607
C	-1.46239	4.45982	23.20234	H	4.33249	8.25436	21.61385
H	-2.26000	5.21767	23.07144	C	6.35183	9.00728	21.55901
H	-1.90464	3.44886	23.12151	H	7.19165	9.29477	22.22705
C	0.28029	5.07726	24.46076	H	6.09706	9.90230	20.95711
C	1.13484	5.44097	25.60359	H	6.72401	8.23103	20.85939
C	0.89716	5.39901	26.97683	C	4.56217	9.60647	23.27682
C	1.94490	5.81990	27.87175	H	3.65185	9.26246	23.80945
C	3.18771	6.26142	27.29203	H	4.27613	10.50332	22.69210
C	3.31103	6.26982	25.90319	H	5.30211	9.93984	24.03479
C	4.42468	6.67936	25.03119	N	1.76521	5.80273	29.22385
C	6.43319	7.30442	24.27783	C	0.49282	5.36018	29.79287
H	7.19801	6.50202	24.29883	H	0.27694	4.29981	29.54088
H	6.93236	8.28696	24.36956	H	-0.35300	5.98615	29.43673
C	5.44599	7.19188	23.07939	H	0.53847	5.44620	30.89258
H	5.81948	6.47001	22.32441	C	2.84323	6.22715	30.11577
C	0.29528	3.43551	21.53367	H	3.13232	7.28446	29.93348
H	1.21005	3.77966	21.00439	H	3.74594	5.59009	29.99920
C	0.69291	2.28838	22.47382	H	2.50194	6.14423	31.16222
H	1.47015	2.60254	23.20046	H	-0.07358	5.04952	27.34543
H	1.11041	1.44275	21.89185	H	4.03006	6.59515	27.90831

4-OMe-$^{i\mathrm{Pr}}$pybox **L5**

49

O	-0.85450	4.50505	24.60452
O	5.65013	7.17833	25.37711
N	0.83524	5.16359	23.24405
N	2.38842	5.83666	25.06790
N	4.27498	6.64314	23.65674
C	-0.17218	4.61908	22.30109
H	-0.40398	5.40333	21.55220
C	-1.39151	4.36896	23.23860
H	-2.19715	5.12244	23.13365
H	-1.82780	3.35560	23.15753
C	0.36682	5.00240	24.46505
C	1.23682	5.37652	25.59251
C	1.02898	5.31756	26.97845
C	3.30668	6.23798	27.24344
C	3.40469	6.26197	25.85229
C	4.48511	6.70705	24.95613
C	6.45631	7.39494	24.16174
H	7.24805	6.61922	24.16023
H	6.92346	8.39314	24.25191
C	5.44567	7.25954	22.98486
H	5.82111	6.54986	22.21928
C	0.35306	3.37212	21.53884
C	0.77327	2.22764	22.47236
H	1.55105	2.54774	23.19597
H	1.19877	1.38997	21.88485
H	-0.08355	1.81293	23.04529
C	-0.67593	2.91936	20.48952
H	-0.26894	2.08128	19.88915
H	-0.93666	3.73682	19.78650
H	-1.61751	2.55557	20.95424
C	5.07157	8.58326	22.26377
H	4.27517	8.29634	21.54478
C	6.27092	9.10632	21.45749
H	7.11187	9.42125	22.11118
H	5.97829	9.99238	20.86007
H	6.65605	8.34275	20.75105
C	4.49544	9.64663	23.20934
H	3.60205	9.27637	23.75251
H	4.17787	10.53869	22.63408
H	5.23577	9.99639	23.95914
O	2.04406	5.76404	29.15495
C	2.09097	5.75806	27.81885
C	0.86090	5.31830	29.83298
H	1.06912	5.43878	30.91057
H	0.65368	4.25012	29.61348
H	-0.01524	5.94110	29.55592
H	1.25834	3.72879	21.00117
H	0.07850	4.94405	27.37847
H	4.12202	6.57742	27.89644

4-Me-$^{i\mathrm{Pr}}$pybox **L6**

48

O	-0.92540	4.64278	24.59094
O	5.60864	7.22515	25.40916
N	0.79130	5.24859	23.24044
N	2.32975	5.93938	25.07536
N	4.25393	6.67737	23.67682
C	-0.21237	4.70271	22.29373
H	-0.42960	5.48086	21.53392
C	-1.44437	4.47594	23.22147
H	-2.24825	5.22669	23.08920
H	-1.88003	3.46109	23.15839
C	0.30374	5.11874	24.45753
C	1.16201	5.50358	25.59007
C	0.95087	5.47523	26.97668
C	1.99383	5.91032	27.83689
C	3.21275	6.35527	27.26123
C	3.33988	6.35884	25.86371
C	4.44272	6.76564	24.97765
C	6.43842	7.40350	24.20307
H	7.21664	6.61455	24.23004
H	6.92124	8.39519	24.28089
C	5.44502	7.26135	23.01224
H	5.82178	6.53125	22.26665
C	0.30336	3.44131	21.54963
H	1.21042	3.78214	21.00524
C	0.71411	2.30642	22.49882
H	1.49595	2.62984	23.21689
H	1.13124	1.45686	21.92226
H	-0.14610	1.90749	23.07816
C	-0.73061	2.98241	20.50794
H	-0.33085	2.13568	19.91531
H	-0.98887	3.79377	19.79689
H	-1.67250	2.62780	20.97926
C	5.10474	8.57657	22.25917
H	4.31846	8.28746	21.53009
C	6.32766	9.06556	21.46591
H	7.15783	9.38807	22.13000
H	6.05859	9.93956	20.83999
H	6.71929	8.27917	20.78851
C	4.52849	9.66718	23.17280
H	3.62087	9.32130	23.70852
H	4.23369	10.55178	22.57428
H	5.26183	10.02153	23.92754
C	1.79834	5.92534	29.32936
H	1.30233	6.87097	29.64073
H	2.76135	5.86778	29.87064
H	1.14943	5.09338	29.66546
H	4.04646	6.69347	27.89342
H	-0.00586	5.11840	27.38392

4-CF_3-$^{i\text{Pr}}$pybox **L7**

48

O	-0.92802	4.66106	24.58574
O	5.60958	7.23947	25.41214
N	0.79634	5.25176	23.23853
N	2.33366	5.94181	25.07864
N	4.26194	6.67767	23.67862
C	-0.20395	4.69846	22.29141
H	-0.41133	5.46779	21.52010
C	-1.44306	4.49056	23.21385
H	-2.23790	5.24941	23.07405
H	-1.88877	3.47997	23.15602
C	0.30179	5.13128	24.45369
C	1.15821	5.51586	25.58657
C	0.93590	5.50094	26.97281
C	1.99018	5.93471	27.80628
C	3.21464	6.38108	27.26183
C	3.34481	6.36884	25.86392
C	4.44850	6.77386	24.97909
C	6.44363	7.41287	24.20671
H	7.22280	6.62543	24.24152
H	6.92376	8.40599	24.28138
C	5.45303	7.26362	23.01423
H	5.83298	6.53206	22.27181
C	0.31157	3.42360	21.56968
H	1.22787	3.74985	21.03162
C	0.70167	2.29772	22.53848
H	1.47998	2.62163	23.25998
H	1.11562	1.43622	21.97792
H	-0.16712	1.91458	23.11520
C	-0.71417	2.96017	20.52161
H	-0.31366	2.10278	19.94508
H	-0.95693	3.76437	19.79716
H	-1.66449	2.61956	20.98574
C	5.10906	8.57627	22.25788
H	4.31836	8.28479	21.53424
C	6.32782	9.06167	21.45642
H	7.16227	9.38601	22.11417
H	6.05464	9.93400	20.82997
H	6.71505	8.27367	20.77855
C	4.53775	9.67059	23.17048
H	3.63330	9.32820	23.71429
H	4.23856	10.55207	22.56941
H	5.27549	10.02981	23.91861
C	1.82258	5.87400	29.32190
F	2.54208	6.84119	29.92633
F	2.25100	4.68017	29.78587
F	0.52855	6.02008	29.67106
H	4.03194	6.73232	27.90644
H	-0.02283	5.16748	27.39243

bpy **L9**

20

C	4.41573	2.96710	5.86297
N	4.57144	4.29958	5.63125
C	4.51883	2.00072	4.85973
N	4.81866	6.84786	5.33232
C	4.79951	2.39974	3.54088
C	4.95726	3.76628	3.28612
C	4.83579	4.69562	4.33542
C	4.96869	6.14884	4.16716
C	5.21894	6.80155	2.94350
C	5.31589	8.19802	2.91996
C	5.15944	8.90746	4.12358
C	4.91290	8.19506	5.30374
H	4.78223	8.68596	6.28481
H	5.51181	8.72552	1.97410
H	5.22880	10.00484	4.15556
H	5.33752	6.22054	2.01824
H	4.37806	0.94192	5.12217
H	4.20120	2.68881	6.90379
H	5.17729	4.12008	2.26932
H	4.89271	1.66306	2.72925

phen **L10**

22

C	4.47930	2.92646	5.86145
N	4.61621	4.25866	5.67166
C	4.62541	1.98850	4.81945
N	4.85363	6.84001	5.34271
C	4.92526	2.40866	3.52250
C	5.07332	3.79995	3.27922
C	4.90613	4.67864	4.38840
C	5.03605	6.09081	4.21227
C	5.33486	6.64496	2.93312
C	5.44245	8.05959	2.85786
C	5.25368	8.81547	4.01544
C	4.96119	8.17317	5.24101
H	4.80518	8.72538	6.18484
H	5.67324	8.54243	1.89549
H	5.33085	9.91269	3.99372
H	4.49804	0.92134	5.05415
H	4.24546	2.60903	6.88726
H	5.04531	1.68680	2.70033
C	5.50443	5.74015	1.82513
C	5.37833	4.37756	1.99307
H	5.73956	6.15818	0.83402
H	5.51142	3.70025	1.13505

pyox **L11**

19

N	4.71764	4.26437	5.63121
N	4.88672	6.78446	5.24212
C	4.98556	4.62484	4.39792

C	5.09899	6.04166	4.10804
C	5.38495	6.61590	2.85725
C	5.45439	8.01374	2.77316
C	5.23684	8.77790	3.93476
C	4.95677	8.12542	5.14719
H	4.77549	8.66335	6.09509
H	5.67771	8.50259	1.81277
H	5.28308	9.87670	3.91011
H	5.54684	5.96841	1.98386
O	5.12863	3.65291	3.47844
C	5.00092	2.38848	4.20639
C	4.62204	2.79670	5.65612
H	3.59407	2.49866	5.94673
H	5.30384	2.38452	6.42593
H	5.97572	1.86341	4.13830
H	4.22902	1.78146	3.69404

dppe **L12**

52

P	3.15111	6.99870	22.64292
P	1.02655	4.81209	23.00176
C	3.45623	8.79471	22.36106
C	4.81640	6.32216	23.08865
C	0.93000	2.99036	23.29411
C	-0.72985	5.37048	23.18825
C	0.29467	2.19029	22.31468
C	-1.28070	6.22752	22.21290
C	5.77042	7.07013	23.81291
C	2.69405	9.79428	23.00519
C	-1.51669	4.97980	24.29460
C	1.46892	2.36701	24.44212
C	0.20073	0.80113	22.48884
H	-0.10019	2.66501	21.40242
C	4.45283	9.18500	21.43643
C	6.99369	6.48487	24.17759
H	5.56030	8.11616	24.08537
C	5.10609	4.98683	22.73420
C	-2.59609	6.70313	22.35343
H	-0.67979	6.48631	21.32621
C	1.36947	0.97480	24.61110
H	1.97019	2.95959	25.22134
C	0.73439	0.18854	23.63624
H	-0.29134	0.19198	21.71450
C	-2.82896	5.45821	24.43166
H	-1.11011	4.28440	25.04738
C	-3.36906	6.32477	23.46316
H	-3.01934	7.36658	21.58301
C	6.32914	4.40273	23.10573
H	4.36070	4.41138	22.15937
C	7.27432	5.15202	23.82610
H	7.73642	7.07522	24.73710
C	4.68891	10.54395	21.17946

H	5.03895	8.42103	20.90324
C	2.93094	11.15446	22.74044
H	1.90364	9.52437	23.72140
C	3.93081	11.53250	21.83001
H	2.32815	11.92010	23.25263
H	1.79077	0.50589	25.51423
H	0.65614	-0.90199	23.77037
H	-4.39968	6.69678	23.57107
H	-3.43735	5.14734	25.29555
H	6.54545	3.36145	22.82211
H	8.23651	4.69941	24.11163
H	5.46728	10.83012	20.45519
H	4.11613	12.59816	21.62320
C	1.84572	5.48220	24.55514
C	2.24404	6.93621	24.29000
H	2.75414	4.86943	24.73665
H	1.18577	5.38695	25.44169
H	2.87436	7.35890	25.10010
H	1.33697	7.56900	24.19125

PPh_3 **L13**

34

P	7.14182	6.38205	13.77293
C	6.97729	4.29666	11.84701
H	7.91888	4.70966	11.45812
C	6.39263	3.16916	11.24471
H	6.89714	2.68537	10.39420
C	6.01246	7.81958	13.48504
C	6.34398	4.92809	12.93734
C	4.86417	7.74325	12.67203
H	4.59312	6.79233	12.19126
C	6.81985	5.93509	15.54951
C	6.04575	6.72243	16.42703
H	5.59302	7.66082	16.07634
C	5.17184	2.66429	11.72109
H	4.71608	1.78091	11.24763
C	5.12091	4.40724	13.42111
H	4.62795	4.87344	14.28770
C	6.36294	9.06145	14.06440
H	7.27406	9.14286	14.67959
C	4.06956	8.88315	12.45979
H	3.17718	8.80994	11.81865
C	7.37316	4.72462	16.02764
H	7.98876	4.10155	15.36097
C	4.53552	3.28688	12.80981
H	3.58034	2.89477	13.19126
C	7.15362	4.31247	17.34942
H	7.59375	3.36768	17.70270
C	4.41120	10.10887	13.05386
H	3.78632	10.99959	12.88436
C	5.83268	6.30897	17.75524
H	5.22409	6.93537	18.42648

C	5.56107	10.19547	13.85833
H	5.84132	11.15386	14.32227
C	6.38355	5.10479	18.21972
H	6.21261	4.78198	19.25839

TMEDA **L14**

24

N	2.64729	7.38871	22.70473
N	1.73376	4.74401	23.23140
C	2.08633	5.63473	24.35960
C	1.89349	7.08572	23.95409
H	3.14721	5.44925	24.62512
H	1.48681	5.40423	25.27366
H	2.20425	7.77282	24.77787
H	0.82168	7.28790	23.75062
C	0.27003	4.54831	23.13174
H	0.05952	3.95724	22.21966
H	-0.24600	5.52233	23.03552
H	-0.12445	4.01874	24.03196
C	2.39616	3.42981	23.38206
H	3.49295	3.56995	23.43423
H	2.17072	2.81753	22.48884
H	2.04756	2.90891	24.30618
C	2.17375	8.68891	22.17105
H	2.72586	8.93754	21.24818
H	2.32475	9.49414	22.92737
H	1.09885	8.61837	21.91911
C	4.10417	7.48439	22.96314
H	4.49332	6.53507	23.37600
H	4.32510	8.30877	23.68347
H	4.62201	7.68407	22.00674

21. UV-Vis Spectra

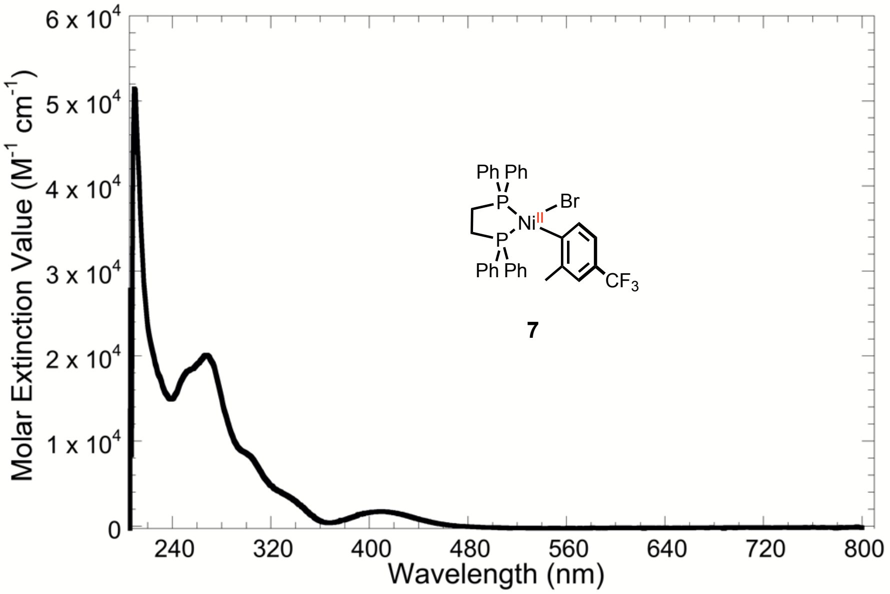

Figure S68. UV-Vis spectrum of (dppe)Ni(*p*-CF_3-*o*-tol)Br **7** in THF at 23 °C.

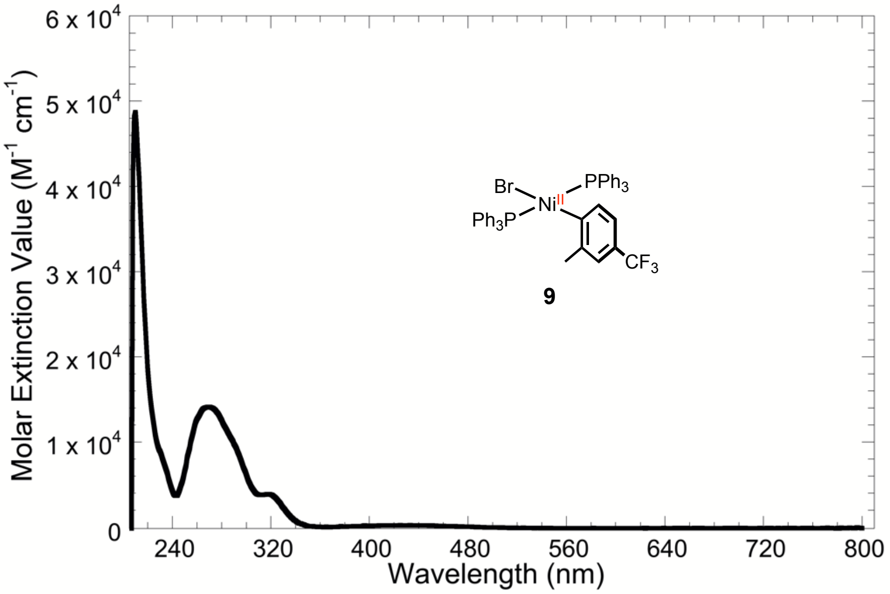

Figure S69 UV-Vis spectrum of $(PPh_3)_2$Ni(*p*-CF_3-*o*-tol)Br **9** in THF at 23 °C.

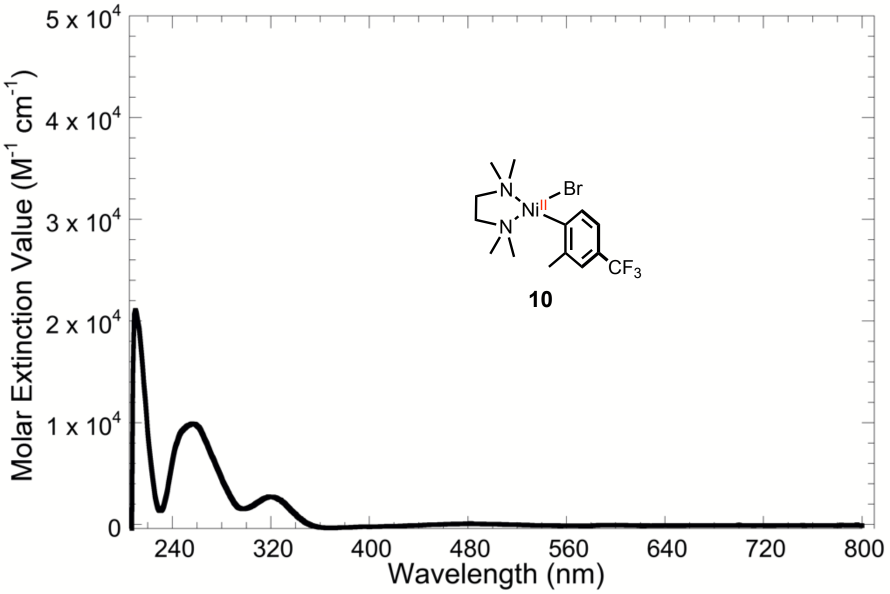

Figure S70. UV-Vis spectrum of (TMEDA)Ni(p-CF_3-o-tol)Br **10** in THF at 23 °C.

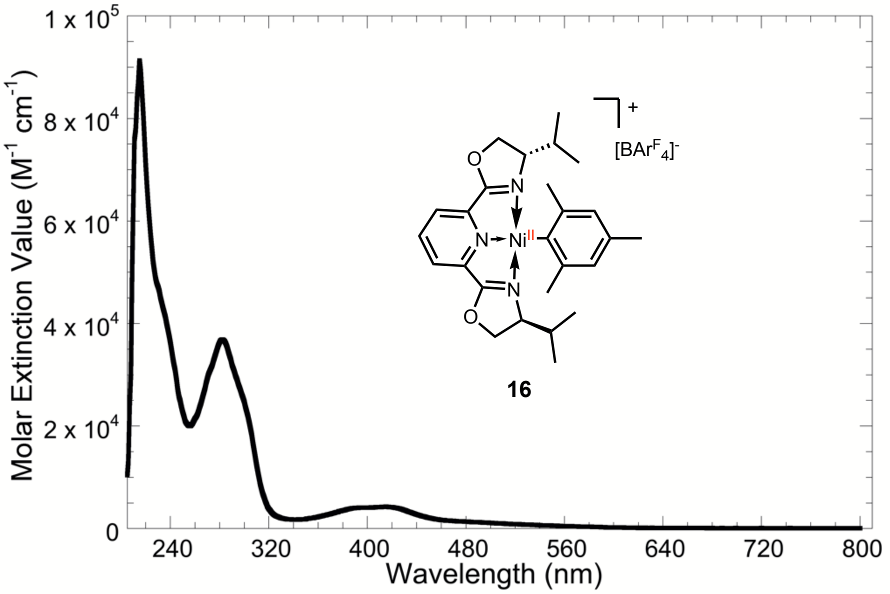

Figure S71. UV-Vis spectrum of [($^{i\text{Pr}}$pybox)Ni(Mes)]$BAr^F{}_4$ **16** in THF at 23 °C.

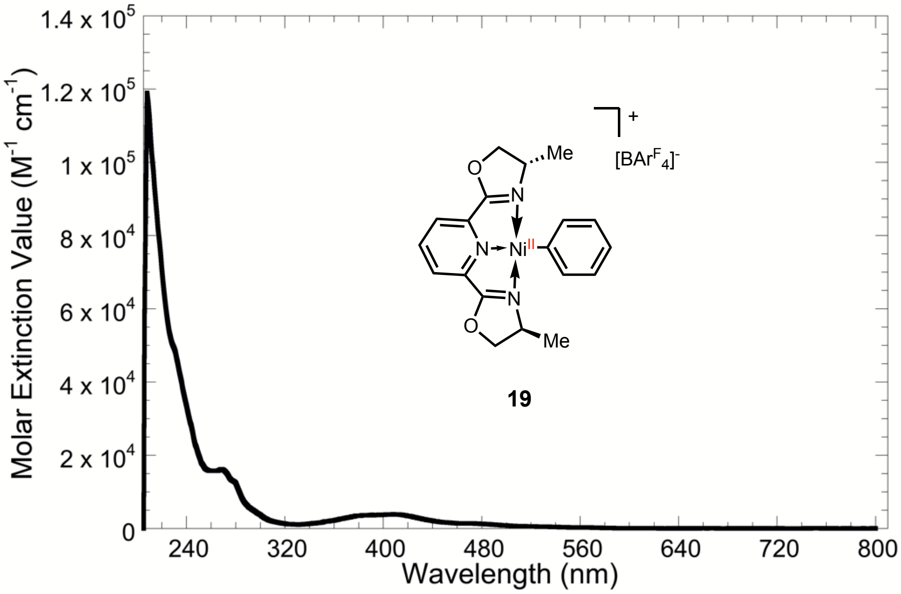

Figure S72. UV-Vis spectrum of [(^{Me}pybox)Ni(Ph)]BAr$^F_4$ **19** in THF at 23 °C.

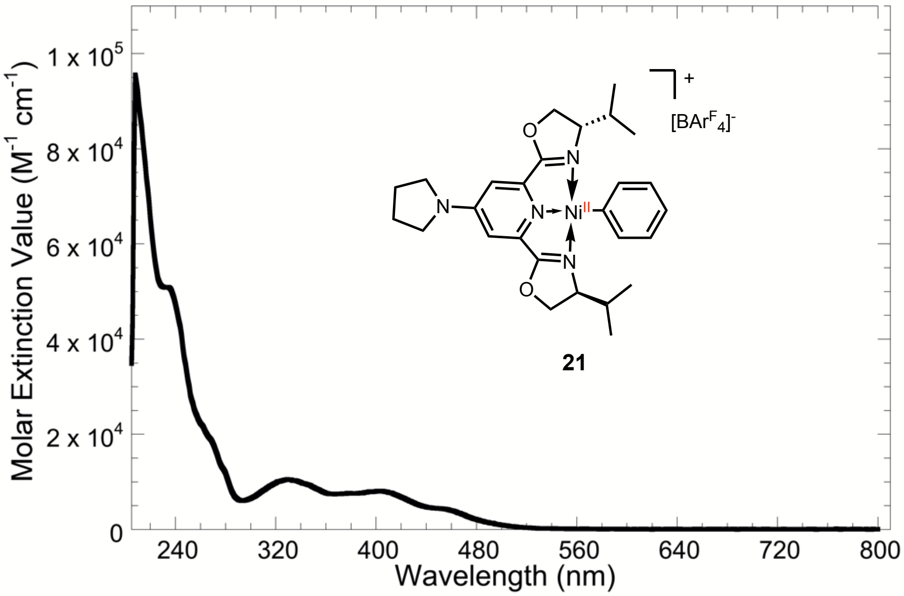

Figure S73. UV-Vis spectrum of [(4-Pyrrolidinyl-$^{i\text{Pr}}$pybox)Ni(Ph)]BAr$^F_4$ **21** in THF at 23 °C.

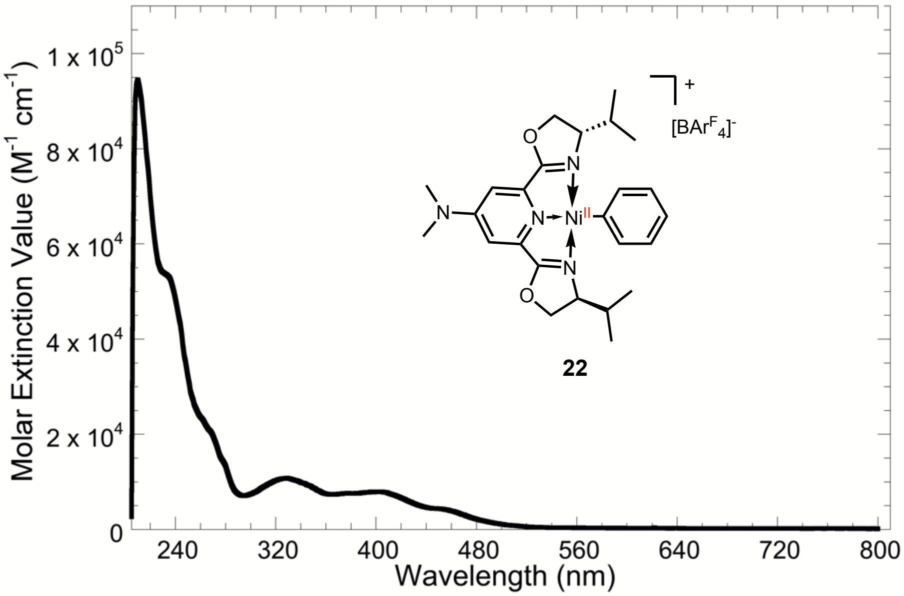

Figure S74. UV-Vis spectrum of [(4-NMe$_2$-^{iPr}pybox)Ni(Ph)]BAr$^F{}_4$ **22** in THF at 23 °C.

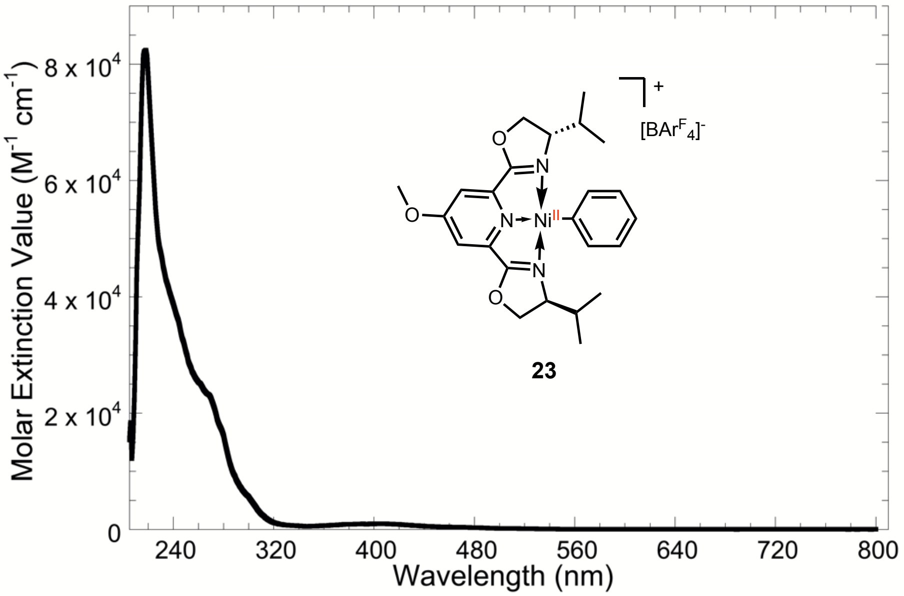

Figure S75. UV-Vis spectrum of [(4-OMe-^{iPr}pybox)Ni(Ph)]BAr$^F{}_4$ **23** in THF at 23 °C.

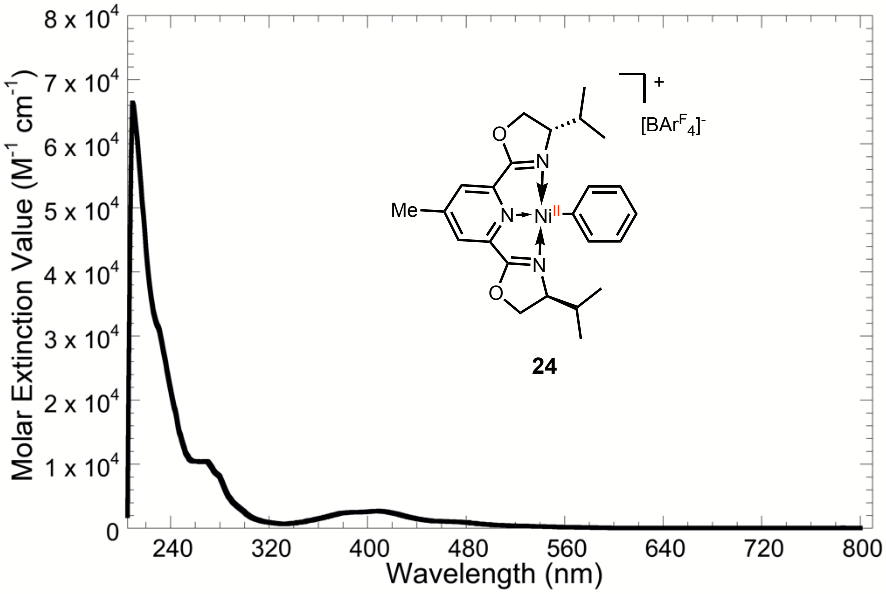

Figure S76. UV-Vis spectrum of [(4-Me-$^{i\text{Pr}}$pybox)Ni(Ph)]BAr^F$_4$ **24** in THF at 23 °C.

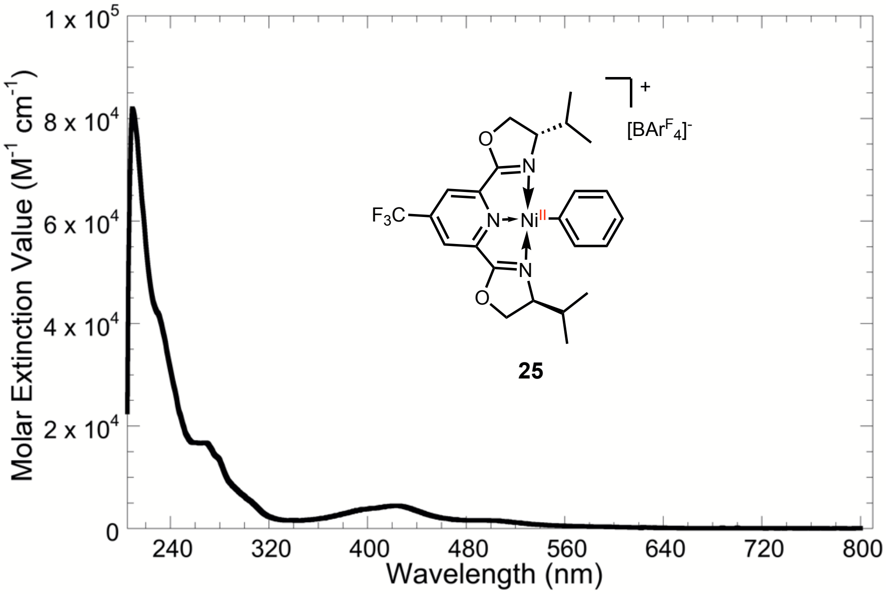

Figure S77. UV-Vis spectrum of [(4-CF$_3$-$^{i\text{Pr}}$pybox)Ni(Ph)]BAr^F$_4$ **25** in THF at 23 °C.

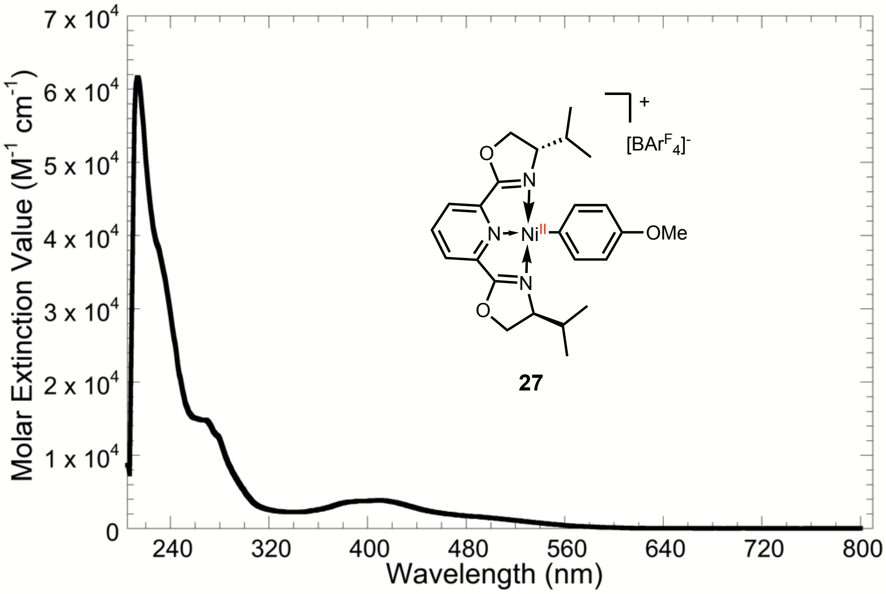

Figure S78. UV-Vis spectrum of [($^{i\mathrm{Pr}}$pybox)Ni(p-MeO-C_6H_4)]BAr$^F_4$ **27** in THF at 23 °C.

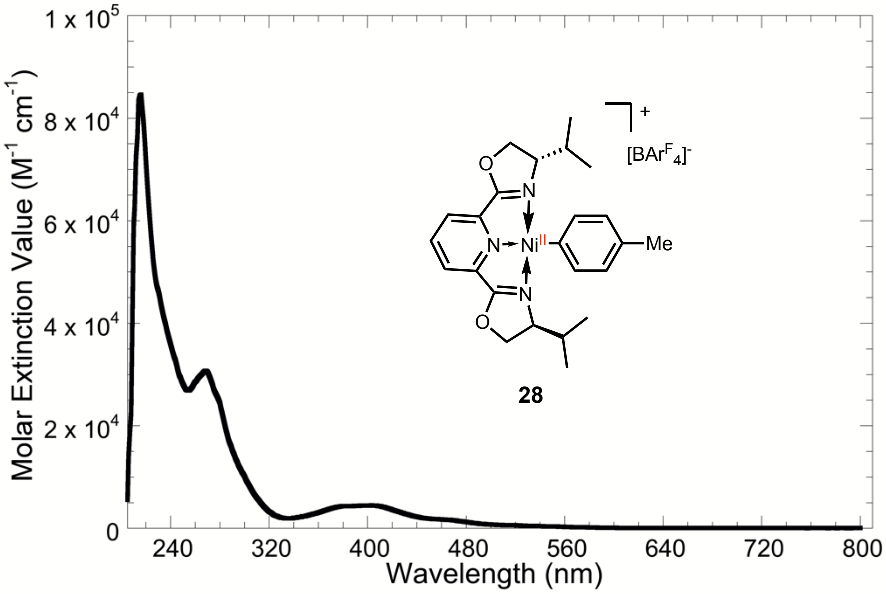

Figure S79. UV-Vis spectrum of [($^{i\mathrm{Pr}}$pybox)Ni(p-tol)]BAr$^F_4$ **28** in THF at 23 °C.

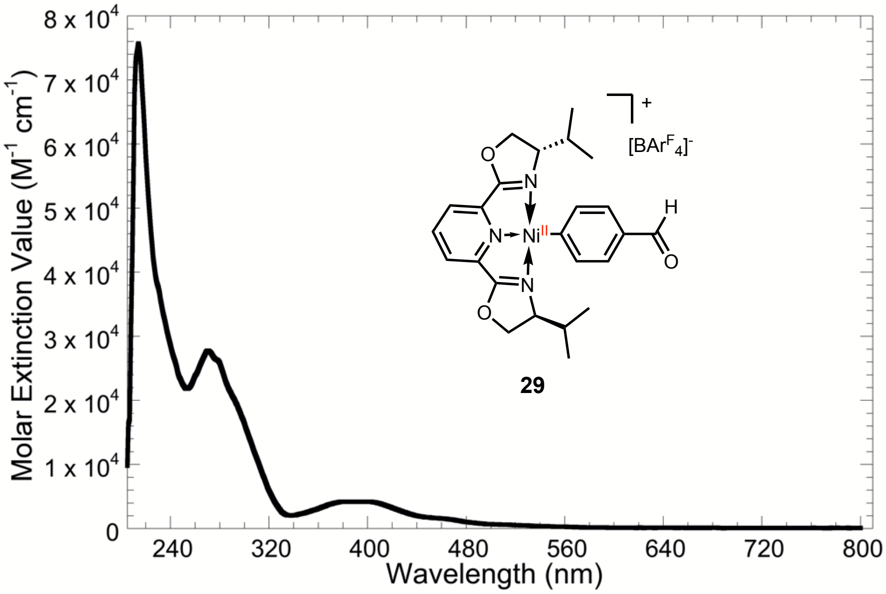

Figure S80. UV-Vis spectrum of [($^{i\text{Pr}}$pybox)Ni(p-CHO-C_6H_4)]BAr$^F_4$ **29** in THF at 23 °C.

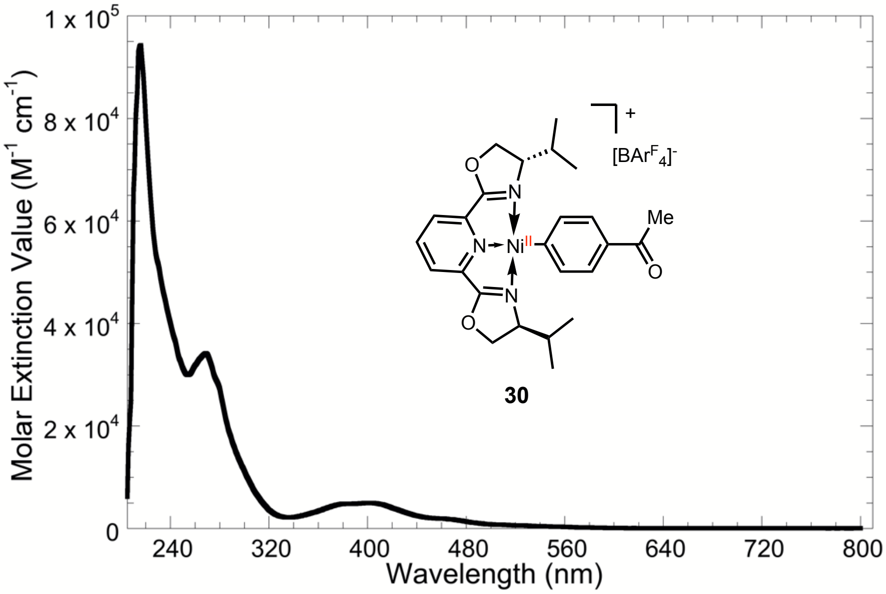

Figure S81. UV-Vis spectrum of [($^{i\text{Pr}}$pybox)Ni(p-COMe-C_6H_4)]BAr$^F_4$ **30** in THF at 23 °C.

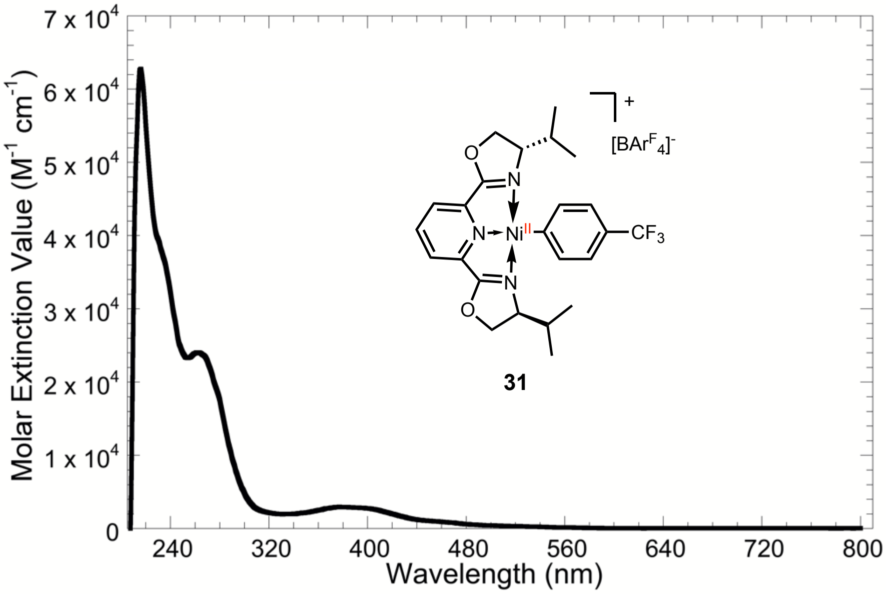

Figure S82. UV-Vis spectrum of [($^{i\text{Pr}}$pybox)Ni(*p*-CF_3-C_6H_4)]$BAr^F{}_4$ **31** in THF at 23 °C.

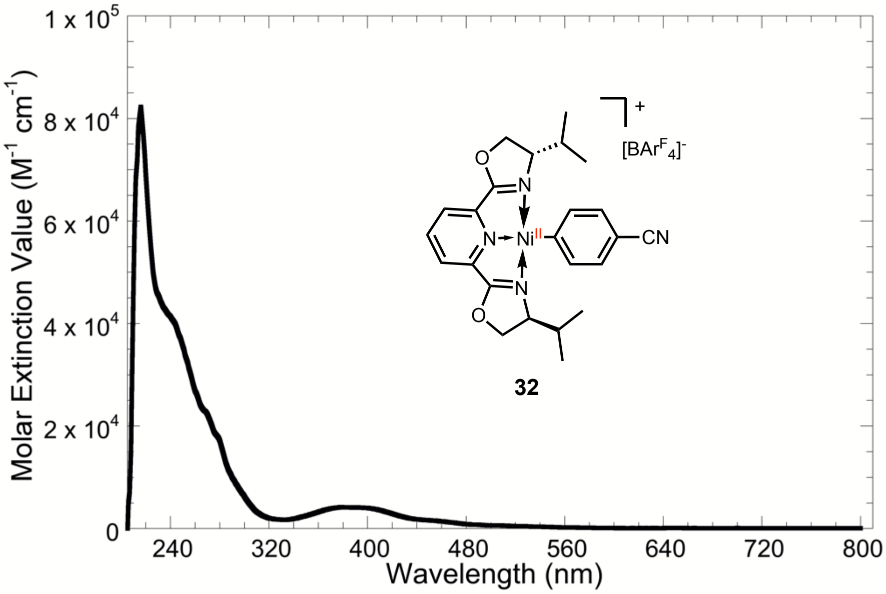

Figure S83. UV-Vis spectrum of [($^{i\text{Pr}}$pybox)Ni(*p*-CN-C_6H_4)]$BAr^F{}_4$ **32** in THF at 23 °C.

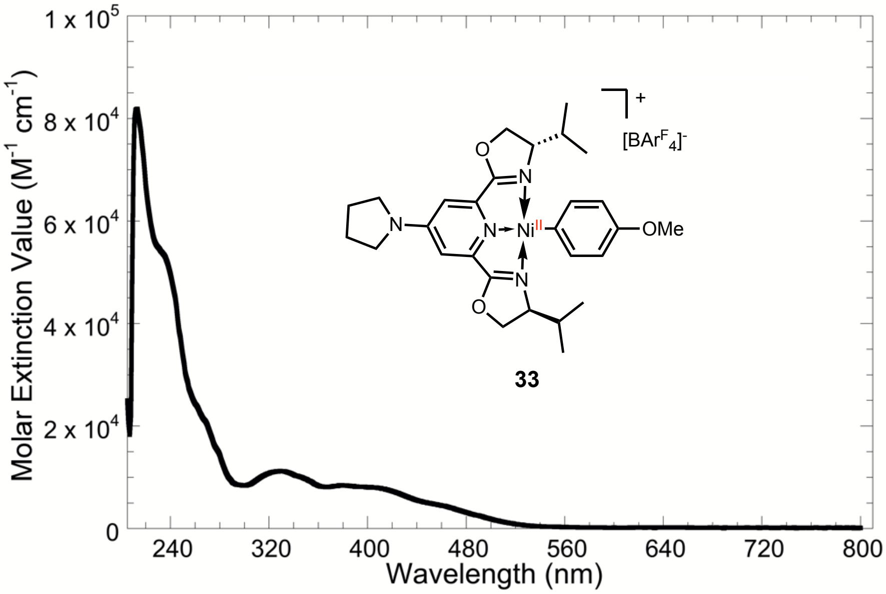

Figure S84. UV-Vis spectrum of [(4-Pyrrolidinyl-$^{i\text{Pr}}$pybox)Ni(p-MeO-C_6H_4)]BAr$^F{}_4$ **33** in THF at 23 °C.

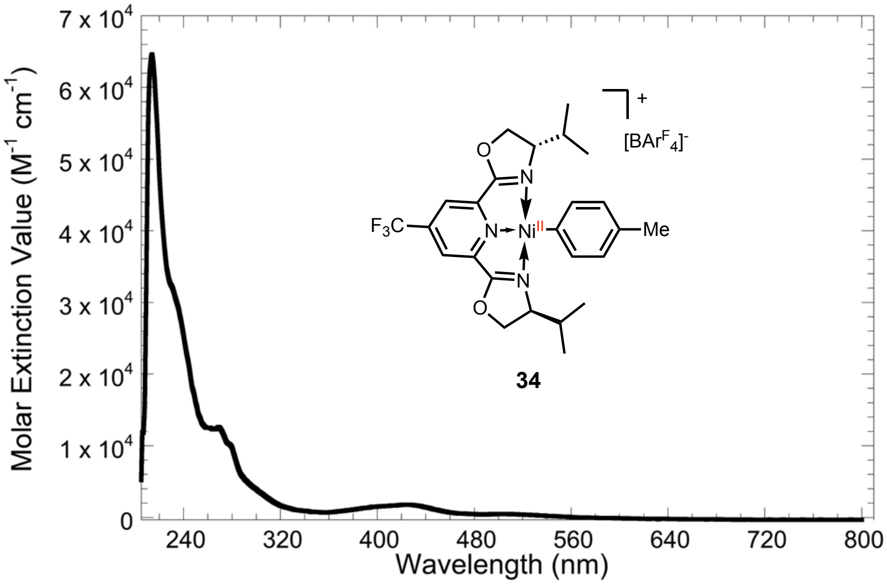

Figure S85. UV-Vis spectrum of [(4-CF_3-$^{i\text{Pr}}$pybox)Ni(p-tol)]BAr$^F{}_4$ **34** in THF at 23 °C.

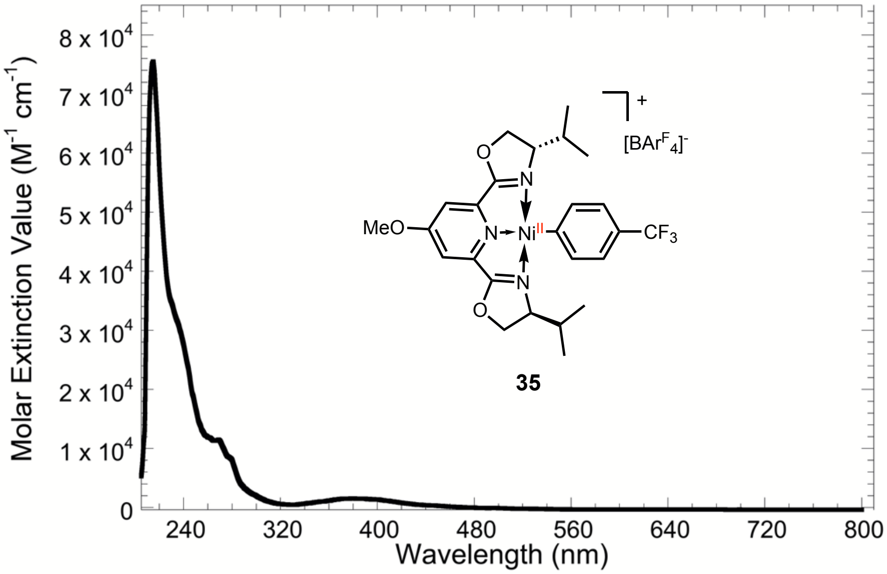

Figure S86. UV-Vis spectrum of [(4-OMe-$^{i\text{Pr}}$pybox)Ni(p-CF_3-C_6H_4)]$BAr^F{}_4$ **35** in THF at 23 °C.

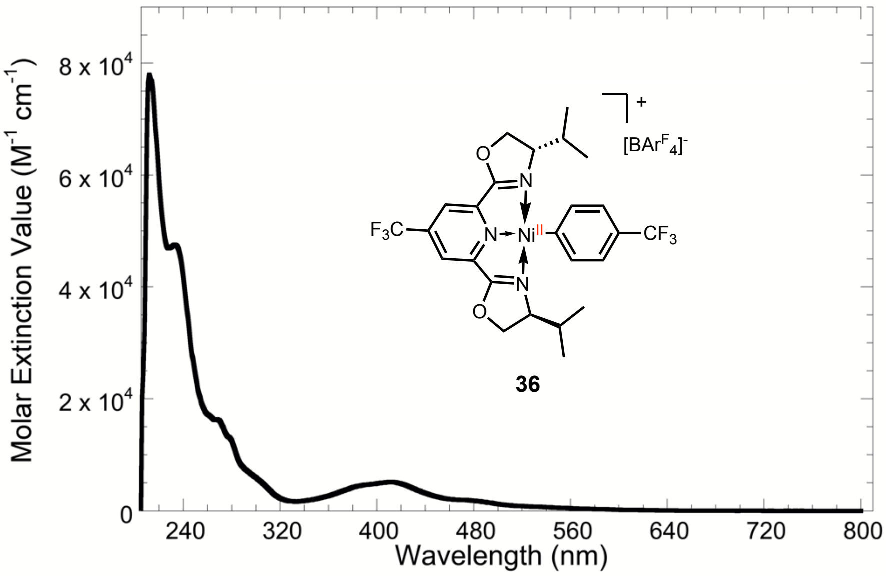

Figure S87. UV-Vis spectrum of [(4-CF_3-$^{i\text{Pr}}$pybox)Ni(p-CF_3-C_6H_4)]$BAr^F{}_4$ **36** in THF at 23 °C.

22. FTIR Spectra

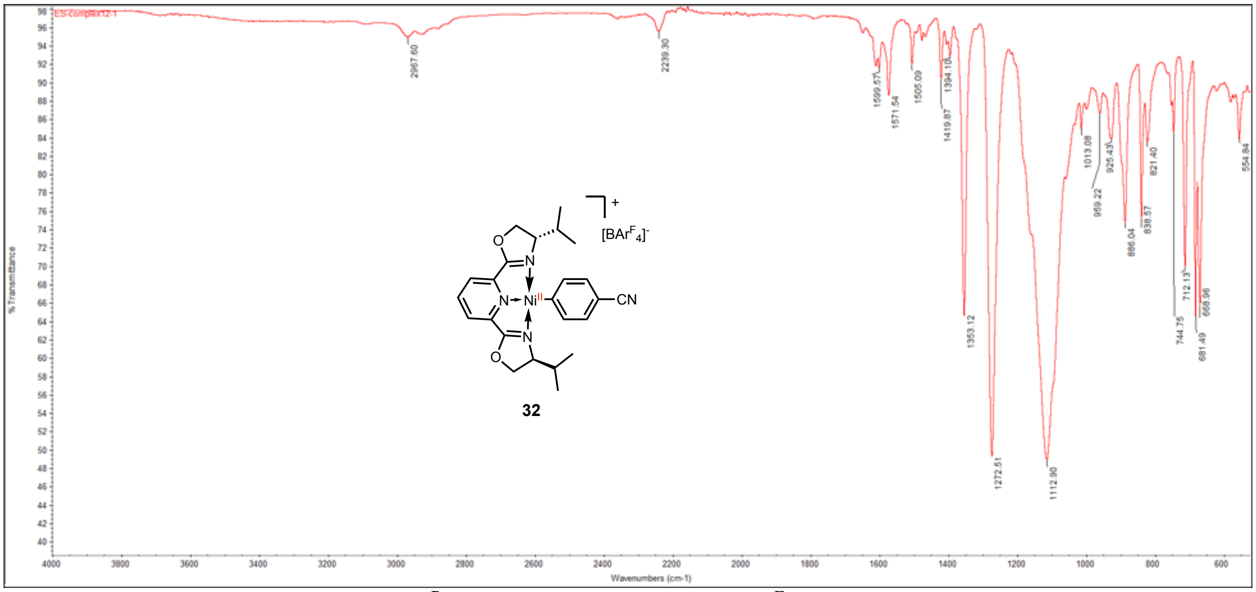

Figure S88. FTIR (neat, ATR) of [($^{i\mathrm{Pr}}$pybox)Ni(p-CN-C_6H_4)]BAr^F_4 **32**.

23. NMR Spectra

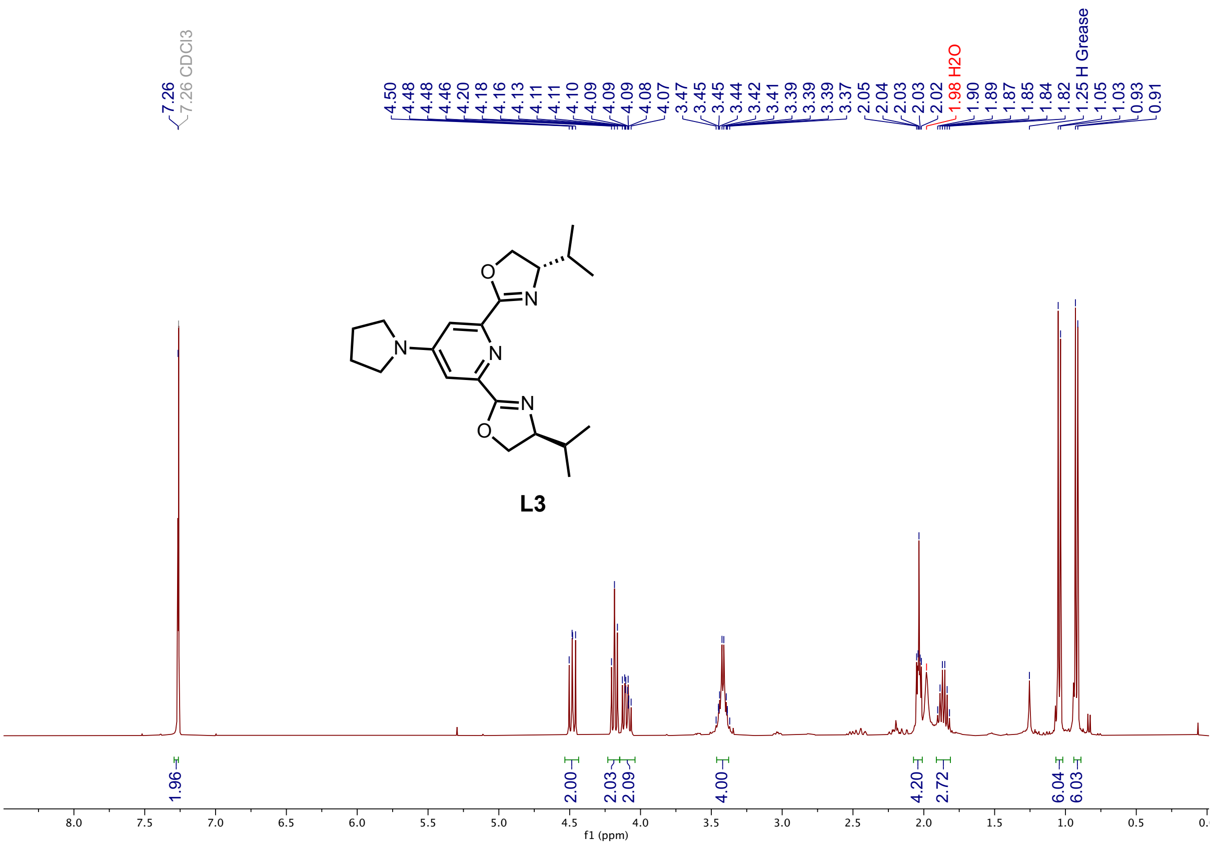

Figure S89. ^{1}H NMR (400 MHz, chloroform-*d*, 25 °C) of 4-Pyrrolidinyl-$^{i\mathrm{Pr}}$pybox **L3.**

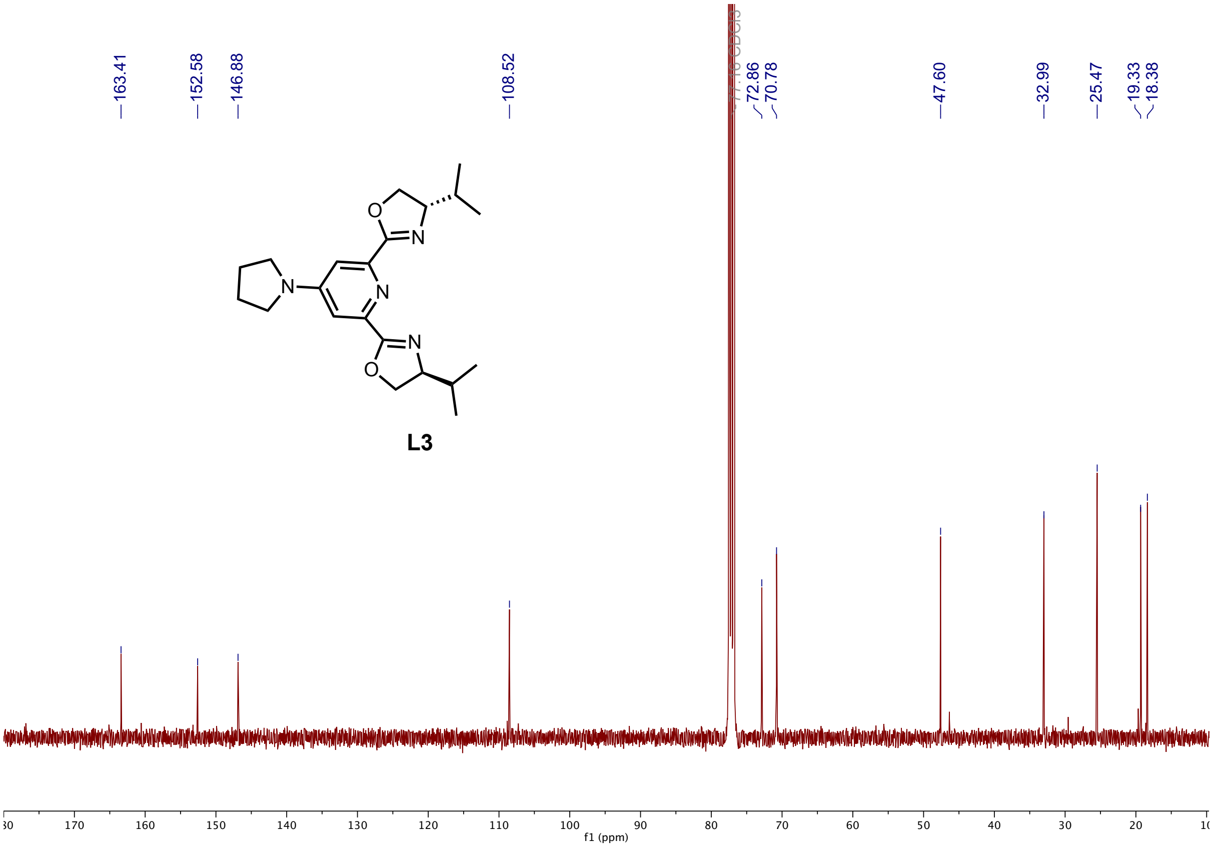

Figure S90. ^{13}C NMR (101 MHz, chloroform-*d*, 25 °C) of 4-Pyrrolidinyl-^{iPr}pybox **L3.**

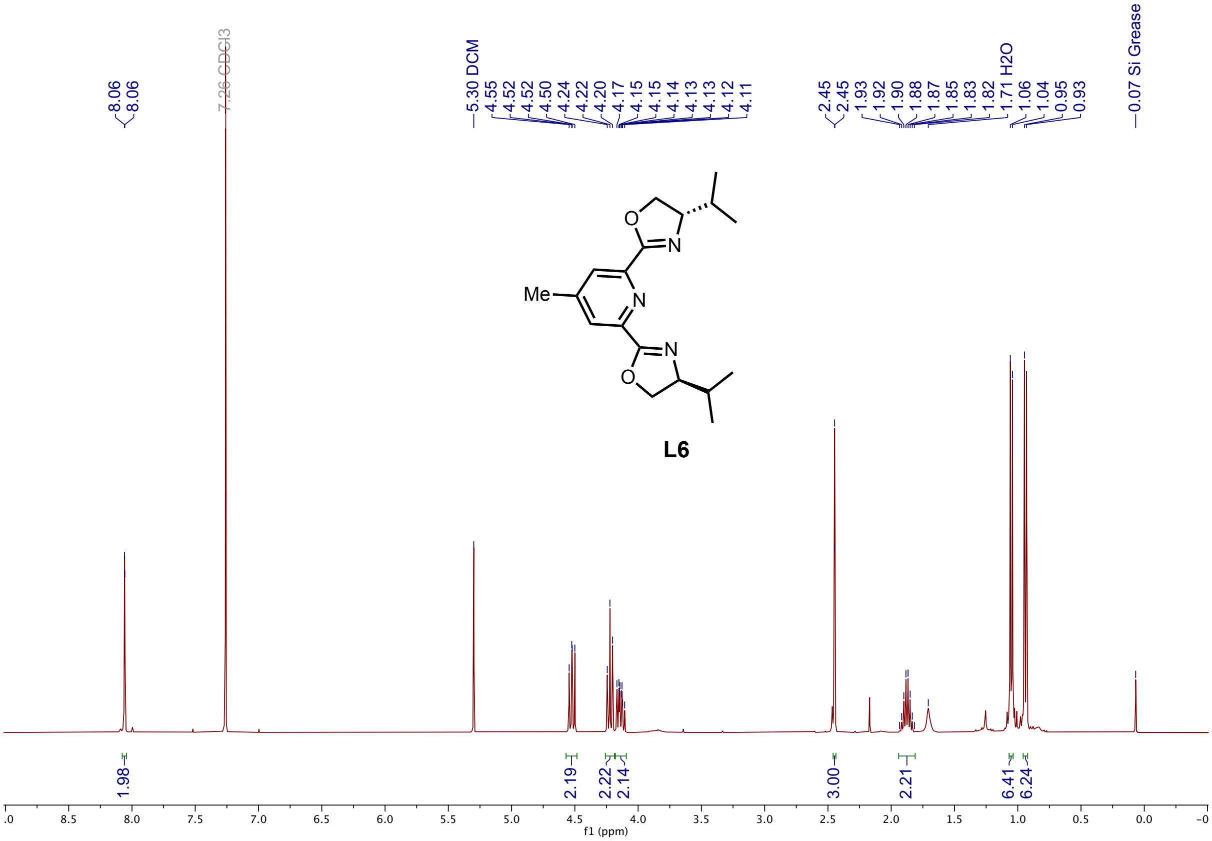

Figure S91. ^{1}H NMR (400 MHz, chloroform-*d*, 25 °C) of 4-Me-$^{i\text{Pr}}$pybox **L6.**

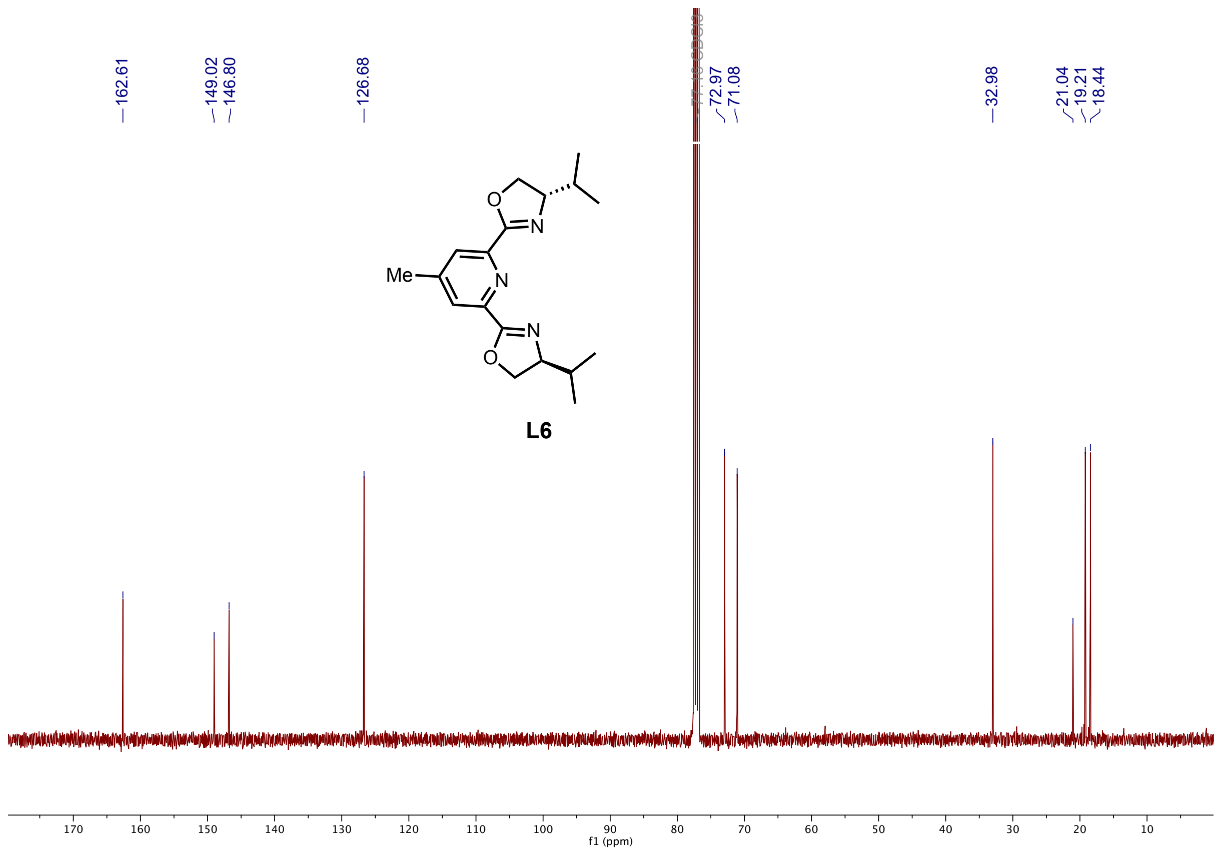

Figure S92. ^{13}C NMR (101 MHz, chloroform-*d*, 25 °C) of 4-Me-^{iPr}pybox **L6.**

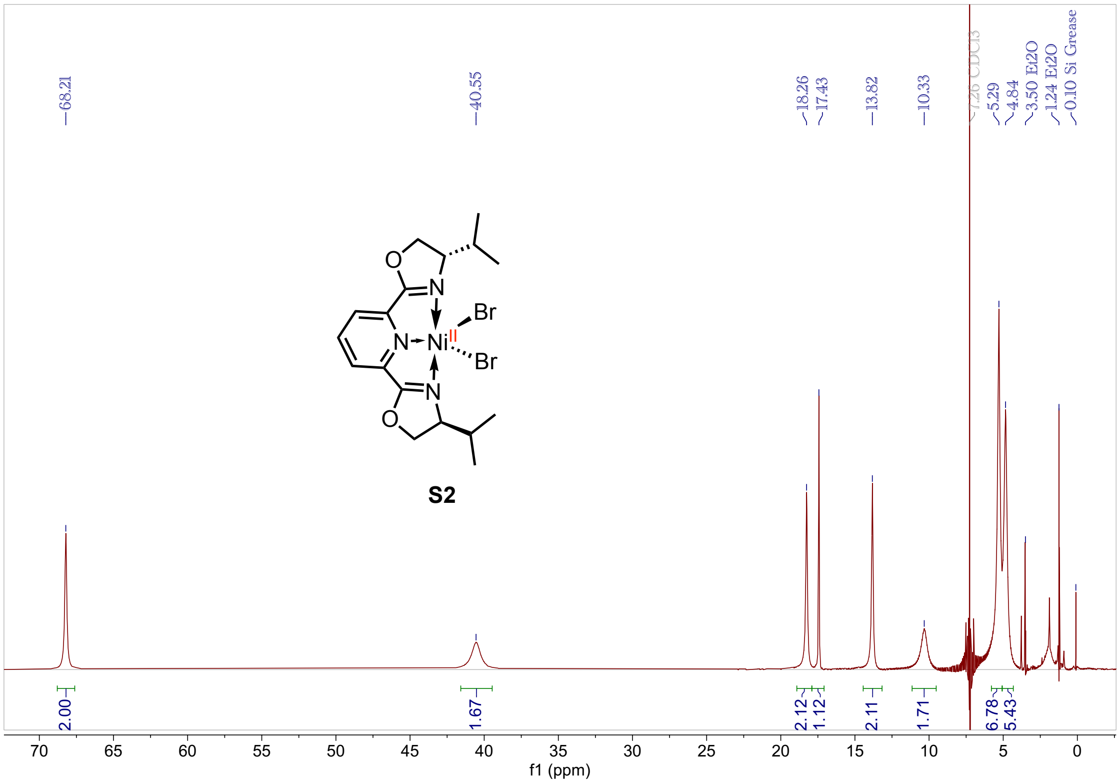

Figure S93. ^{1}H NMR (400 MHz, chloroform-*d*, 25 °C) of ($^{i\text{Pr}}$pybox)$NiBr_2$ **S2.**

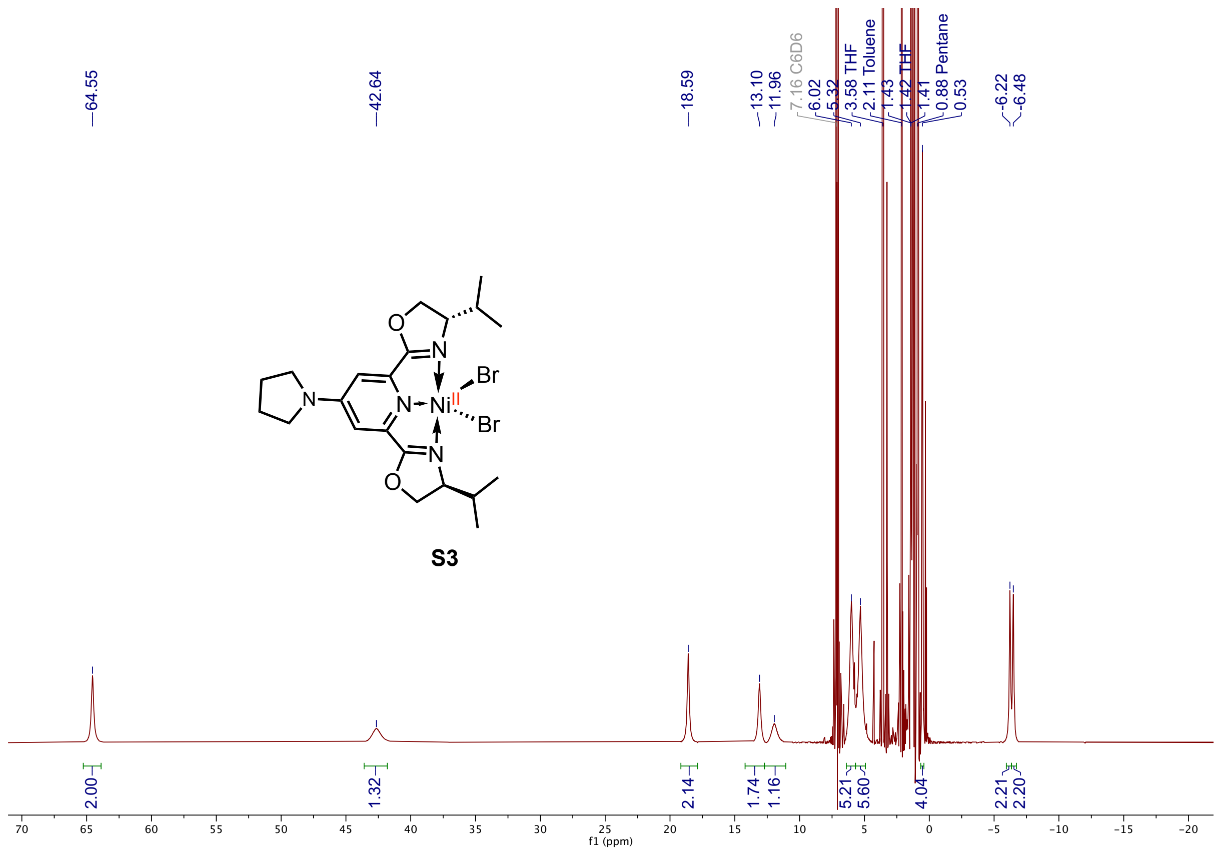

Figure S94. ^{1}H NMR (400 MHz, benzene-*d*, 25 °C) of (4-Pyrrolidinyl-^{iPr}pybox)$NiBr_2$ **S3.**

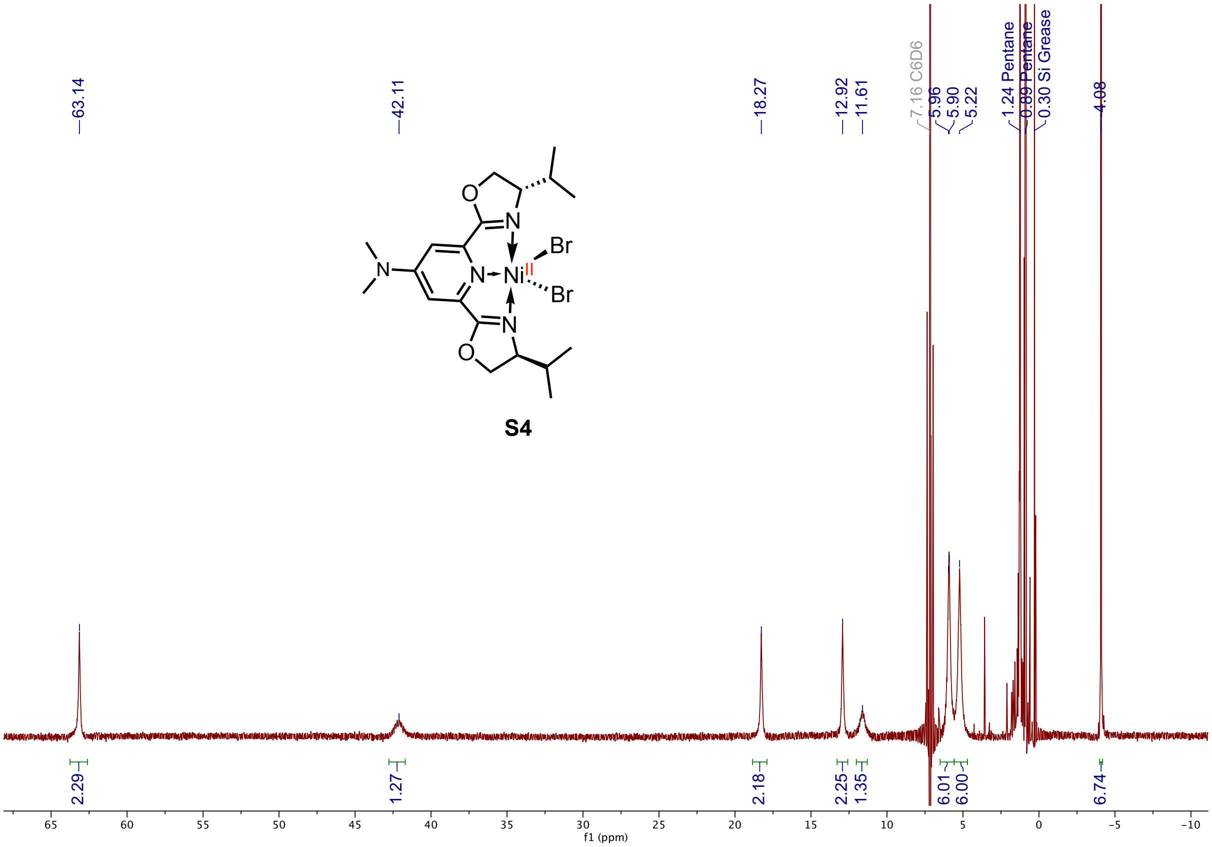

Figure S95. ^{1}H NMR (400 MHz, benzene-*d*, 25 °C) of (4-NMe$_2$-$^{i\text{Pr}}$pybox)NiBr$_2$ **S4.**

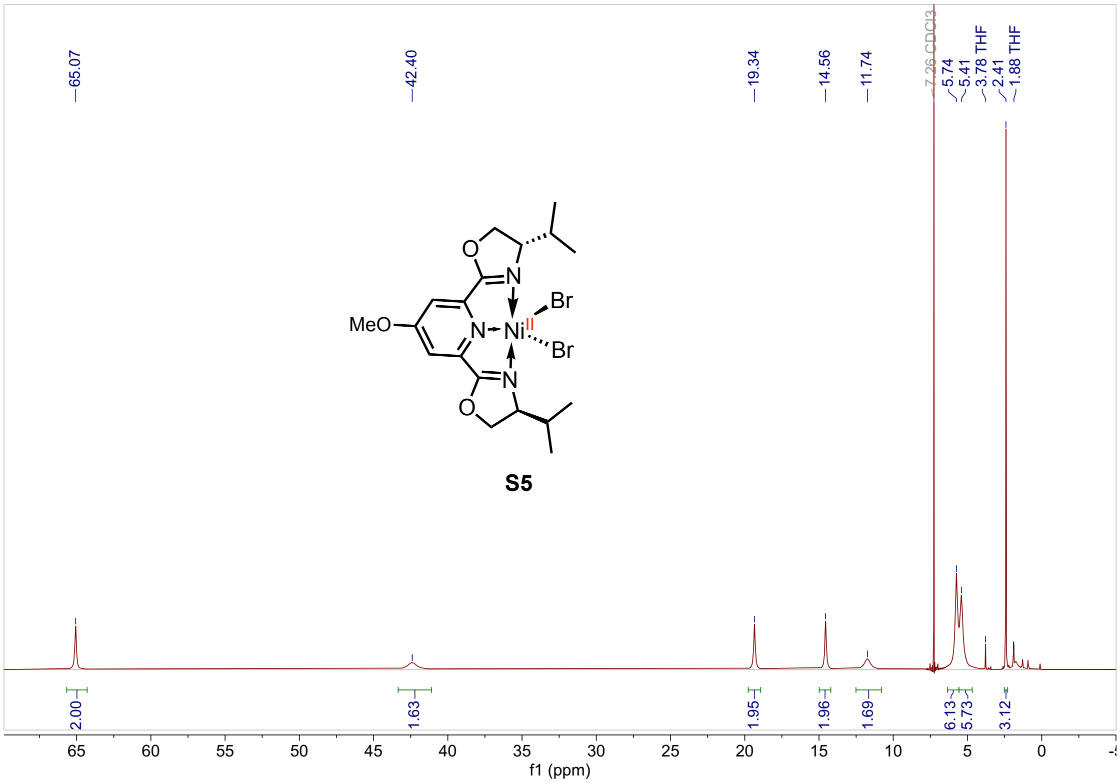

Figure S96. ^{1}H NMR (400 MHz, chloroform-*d*, 25 °C) of (4-OMe-$^{i\text{Pr}}$pybox)$NiBr_2$ **S5.**

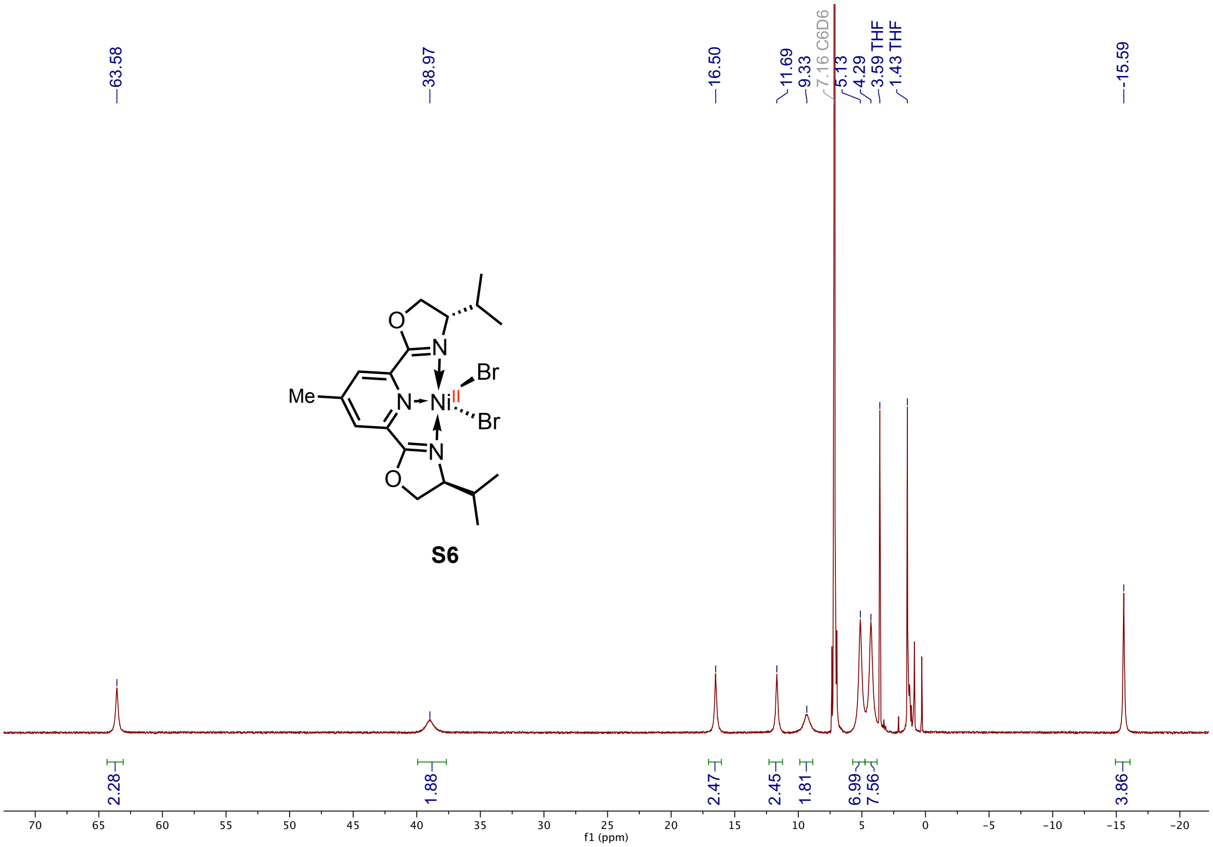

Figure S97. ^{1}H NMR (400 MHz, benzene-*d*, 25 °C) of (4-Me-$^{i\text{Pr}}$pybox)$NiBr_2$ **S6.**

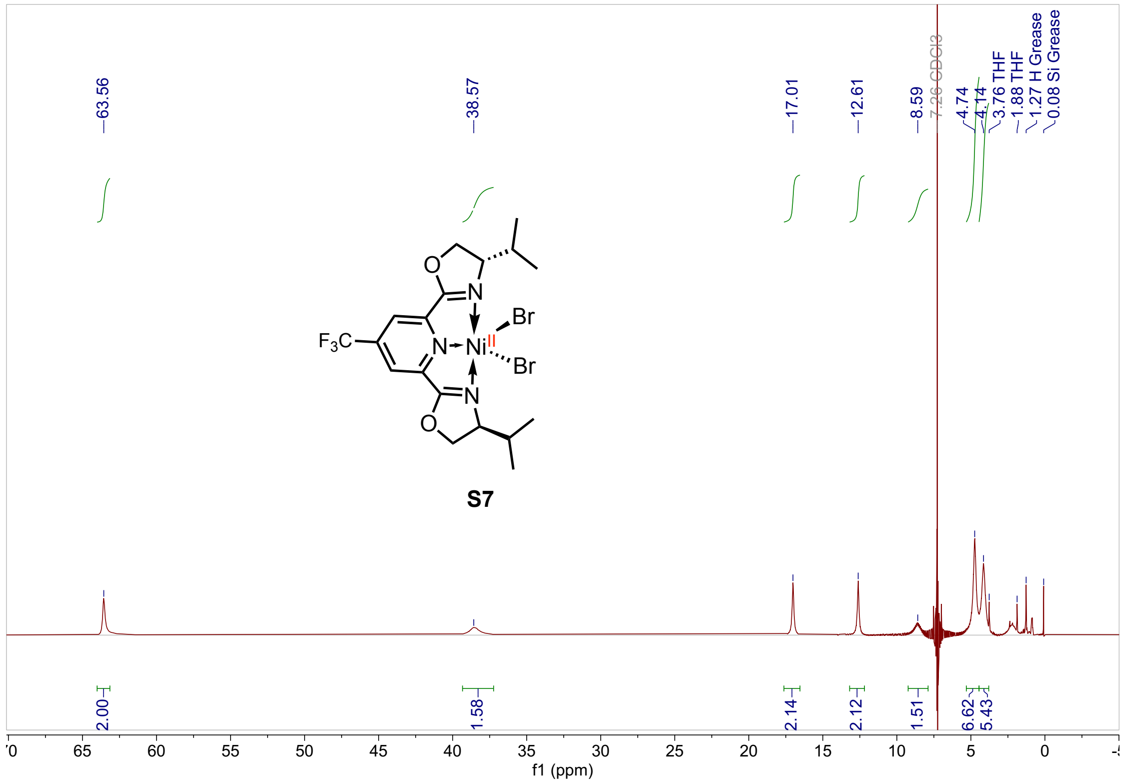

Figure S98. ^{1}H NMR (400 MHz, chloroform-*d*, 25 °C) of (4-CF$_3$-$^{i\mathrm{Pr}}$pybox)NiBr$_2$ **S7.**

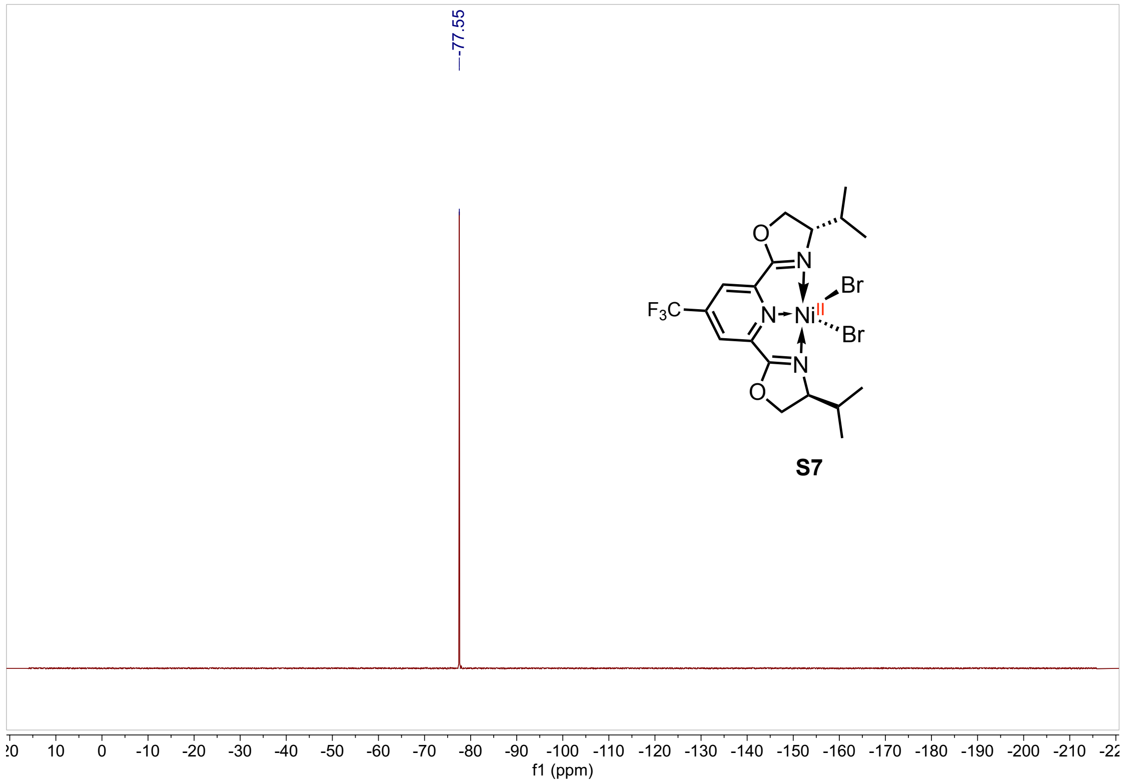

Figure S99. ^{19}F NMR (471 MHz, chloroform-*d*, 25 °C) of (4-CF_3-^{iPr}pybox)$NiBr_2$ **S7.**

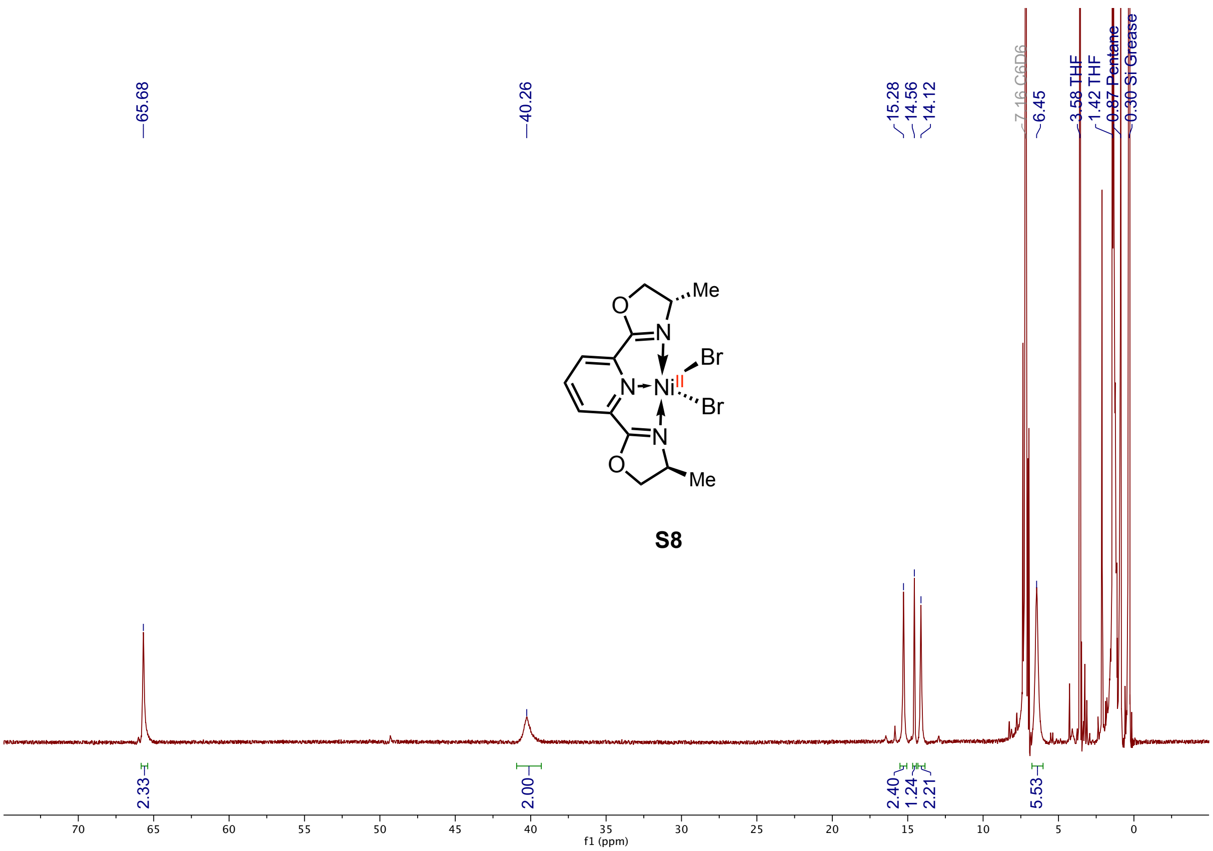

S8

Figure S100. ^{1}H NMR (400 MHz, benzene-*d*, 25 °C) of (^{Me}pybox)$NiBr_2$ **S8.**

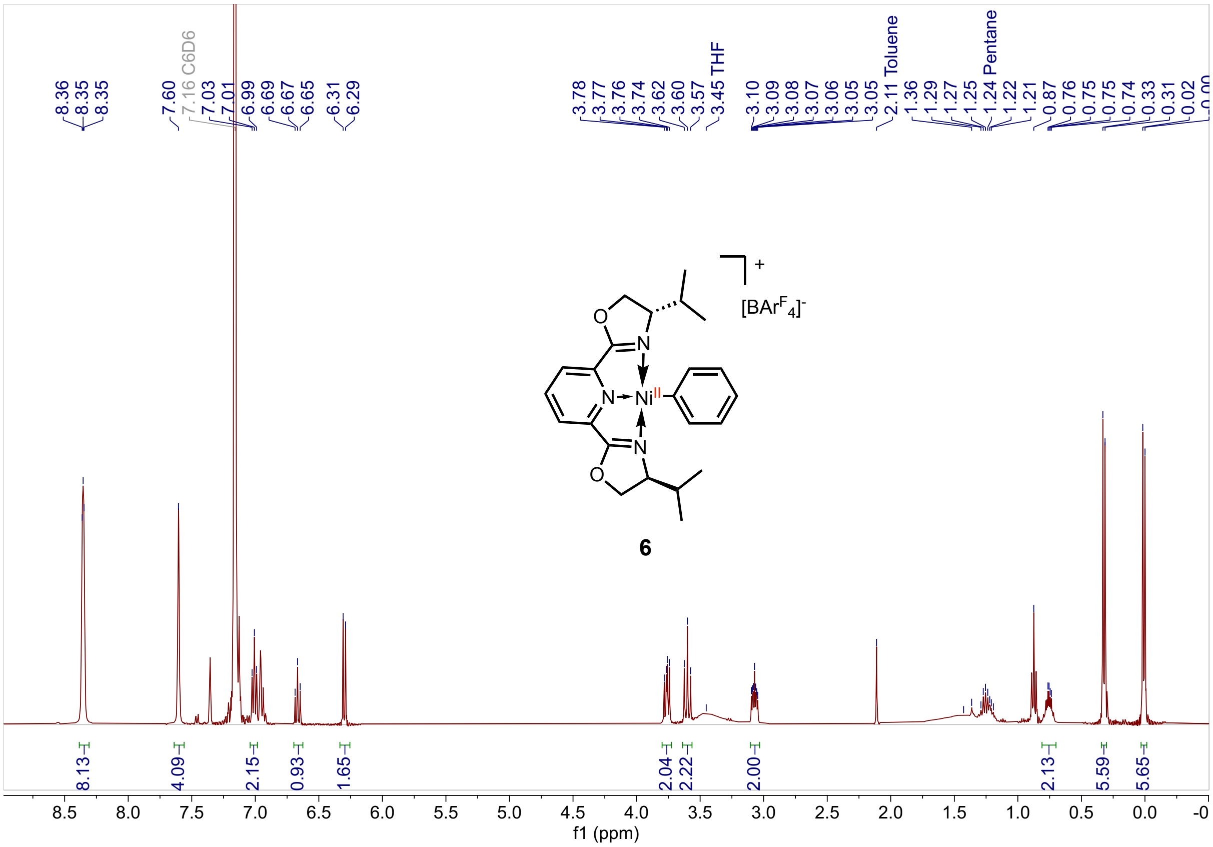

Figure S101. ^{1}H NMR (400 MHz, benzene-*d*, 25 °C) of [(^{iPr}pybox)Ni(Ph)]BAr$^F_4$ **6.**

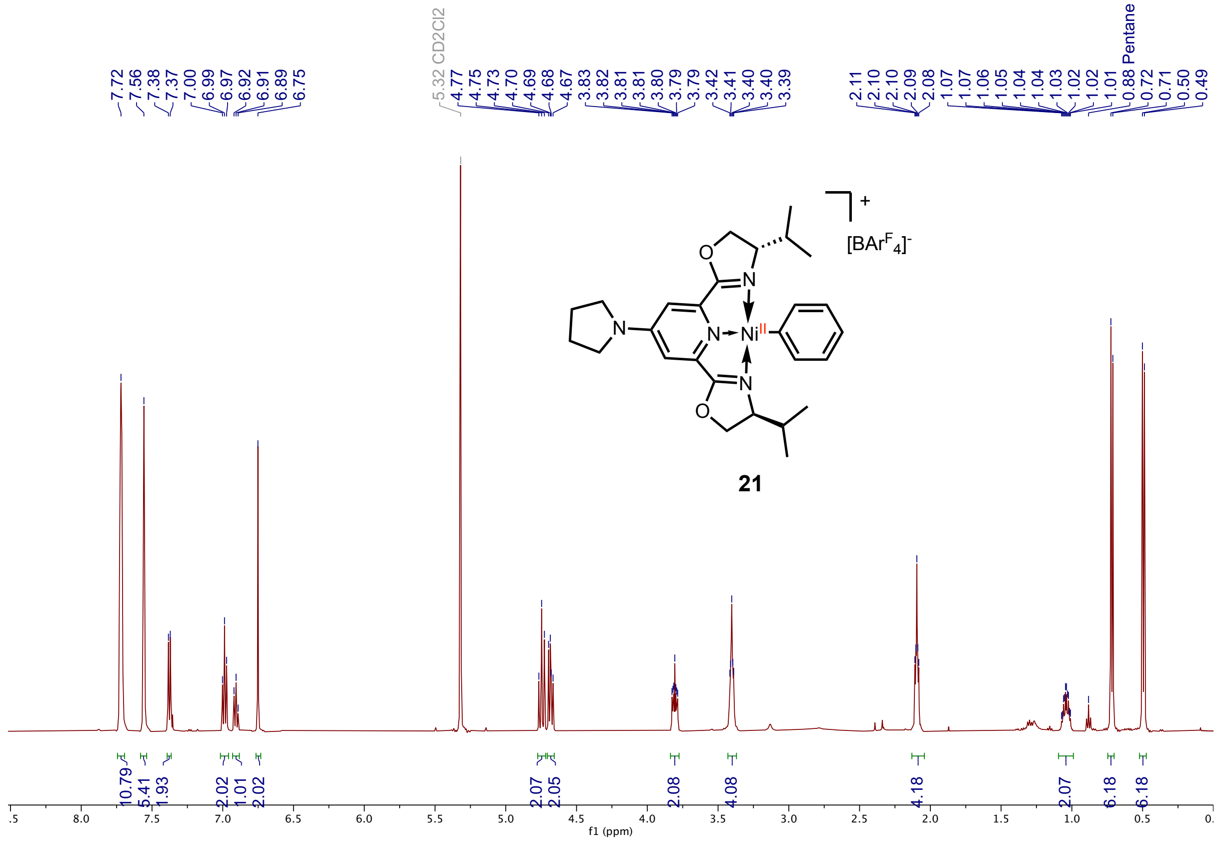

Figure S102. ^{1}H NMR (500 MHz, CD_2Cl_2, 25 °C) of [(4-Pyrrolidinyl-^{iPr}pybox)Ni(Ph)]BAr^F_4 **21.**

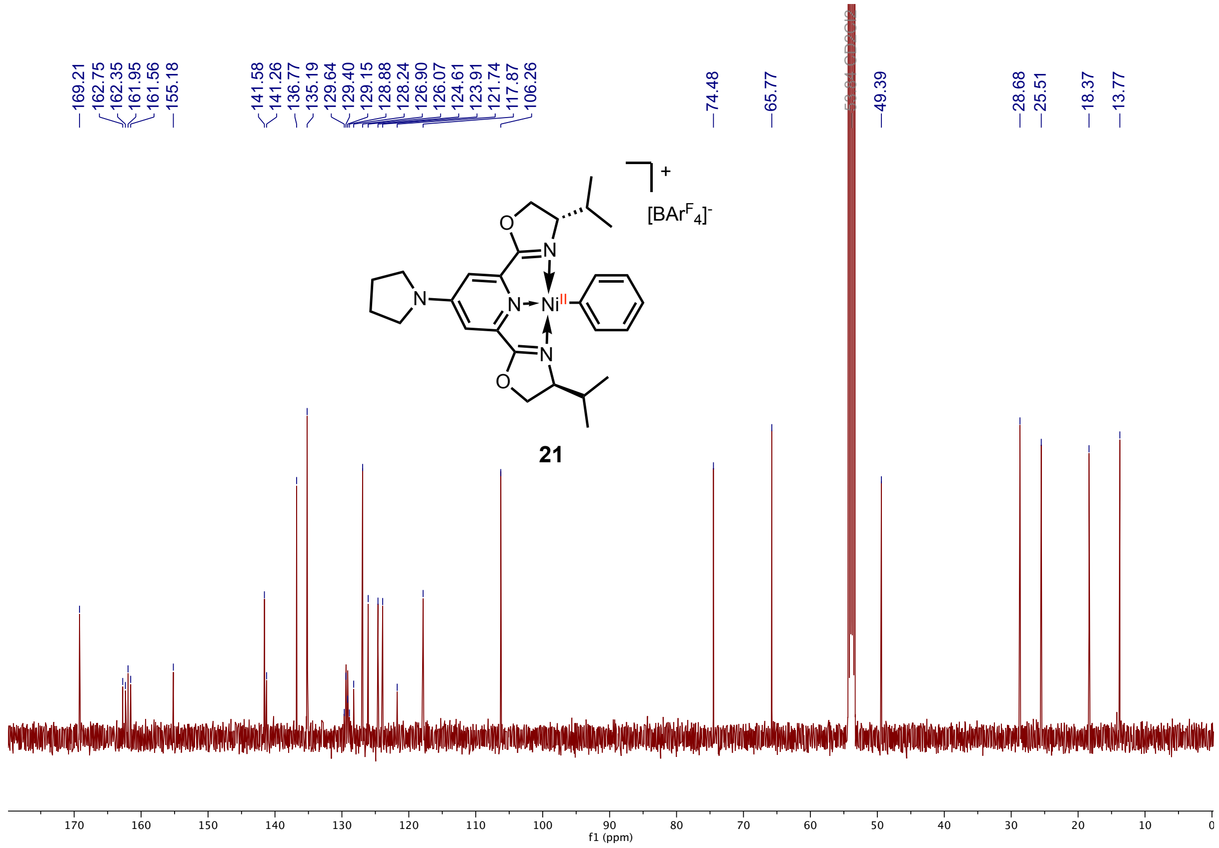

21

Figure S103. ^{13}C NMR (126 MHz, CD_2Cl_2, 25 °C) of [(4-Pyrrolidinyl-^{iPr}pybox)Ni(Ph)]BAr$^{F}_{4}$ **21.**

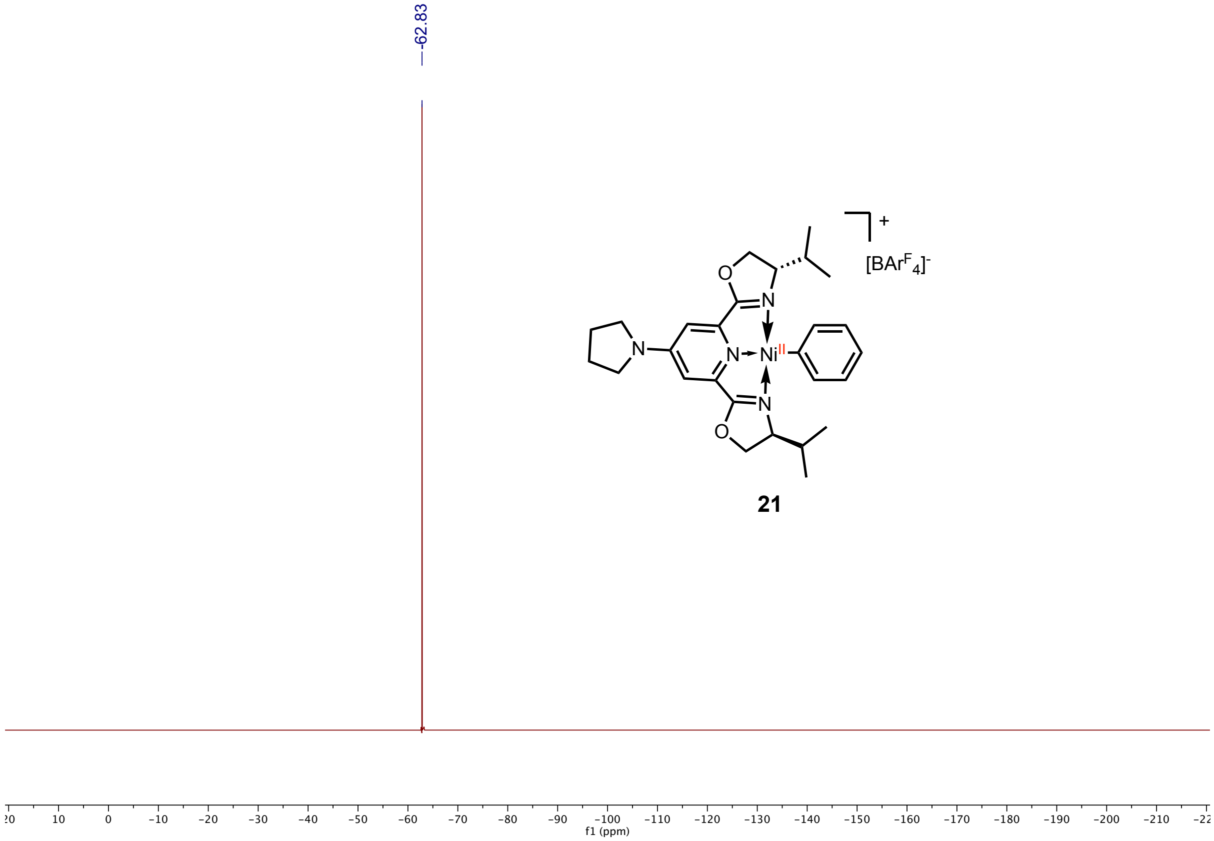

Figure S104. ^{19}F NMR (471 MHz, CD_2Cl_2, 25 °C) of [(4-Pyrrolidinyl-^{iPr}pybox)Ni(Ph)]BAr^F_4 **21.**

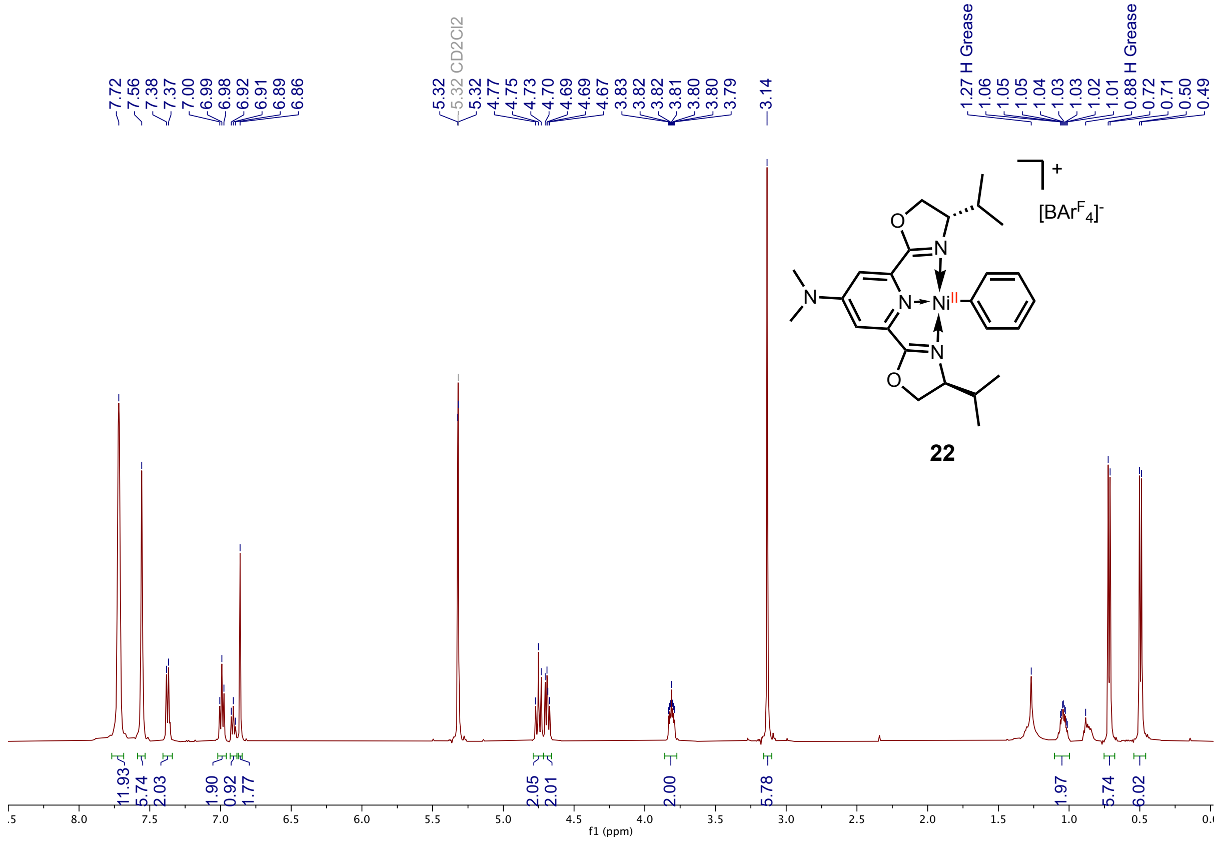

Figure S105. ^{1}H NMR (500 MHz, CD_2Cl_2, 25 °C) of [(4-NMe$_2$-^{iPr}pybox)Ni(Ph)]BAr$^{F}_{4}$ **22.**

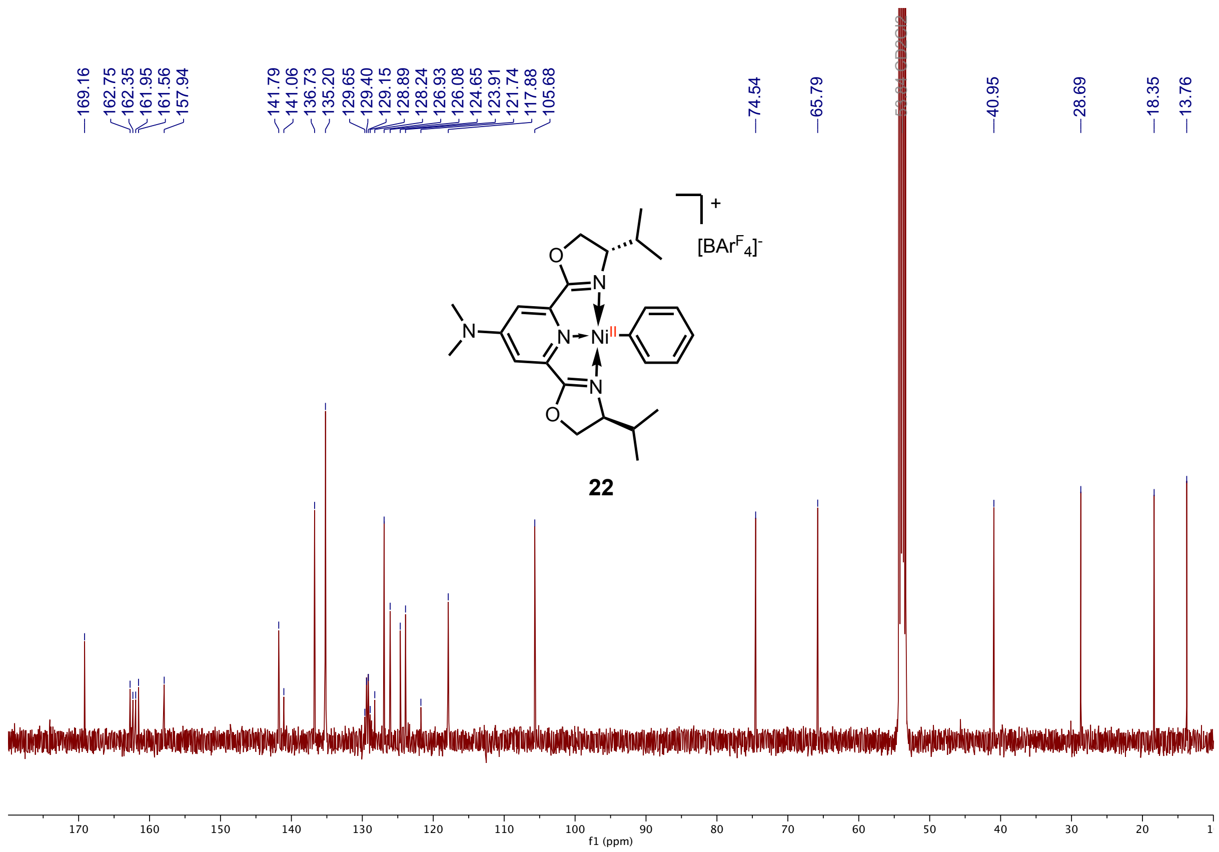

Figure S106. ^{13}C NMR (126 MHz, CD_2Cl_2, 25 °C) of [(4-NMe$_2$-^{iPr}pybox)Ni(Ph)]BAr$^F_4$ **22.**

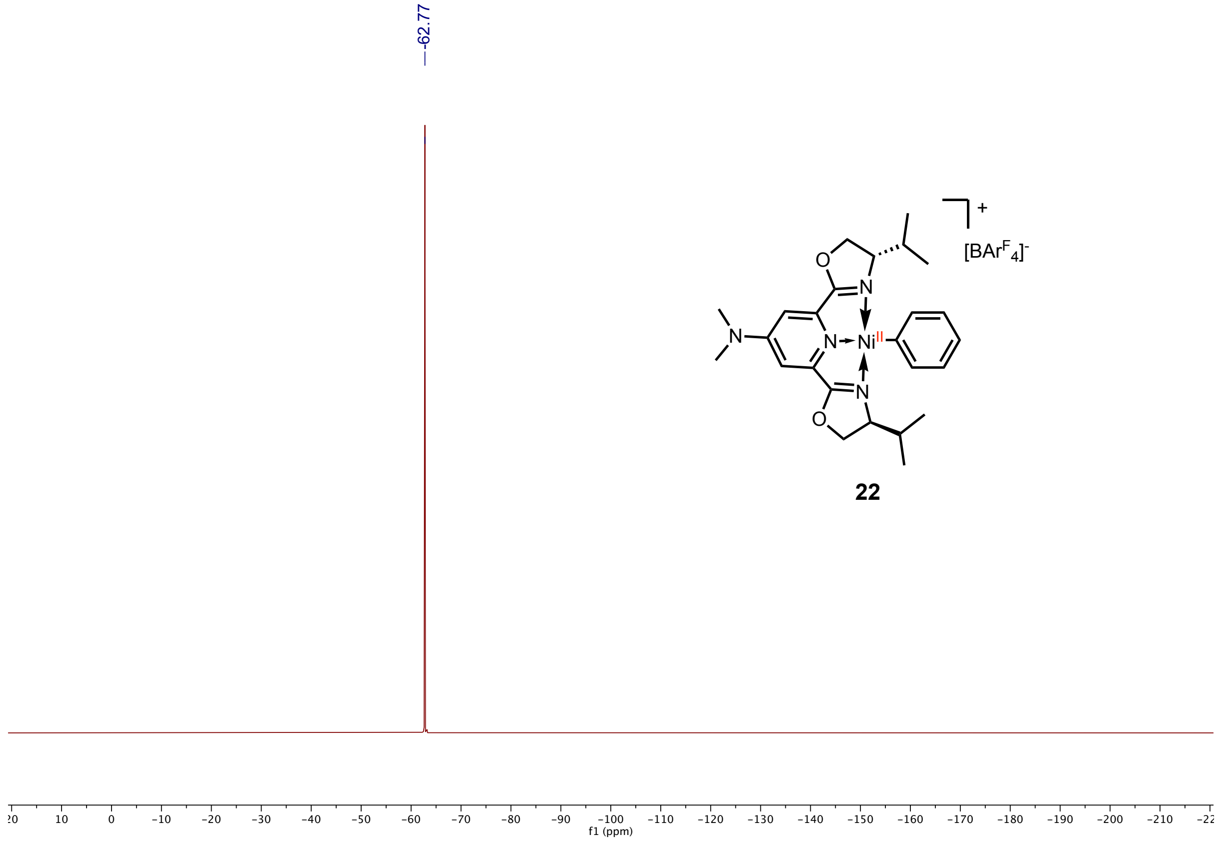

Figure S107. ^{19}F NMR (471 MHz, CD_2Cl_2, 25 °C) of [(4-NMe$_2$-^{iPr}pybox)Ni(Ph)]BAr$^F_4$ **22.**

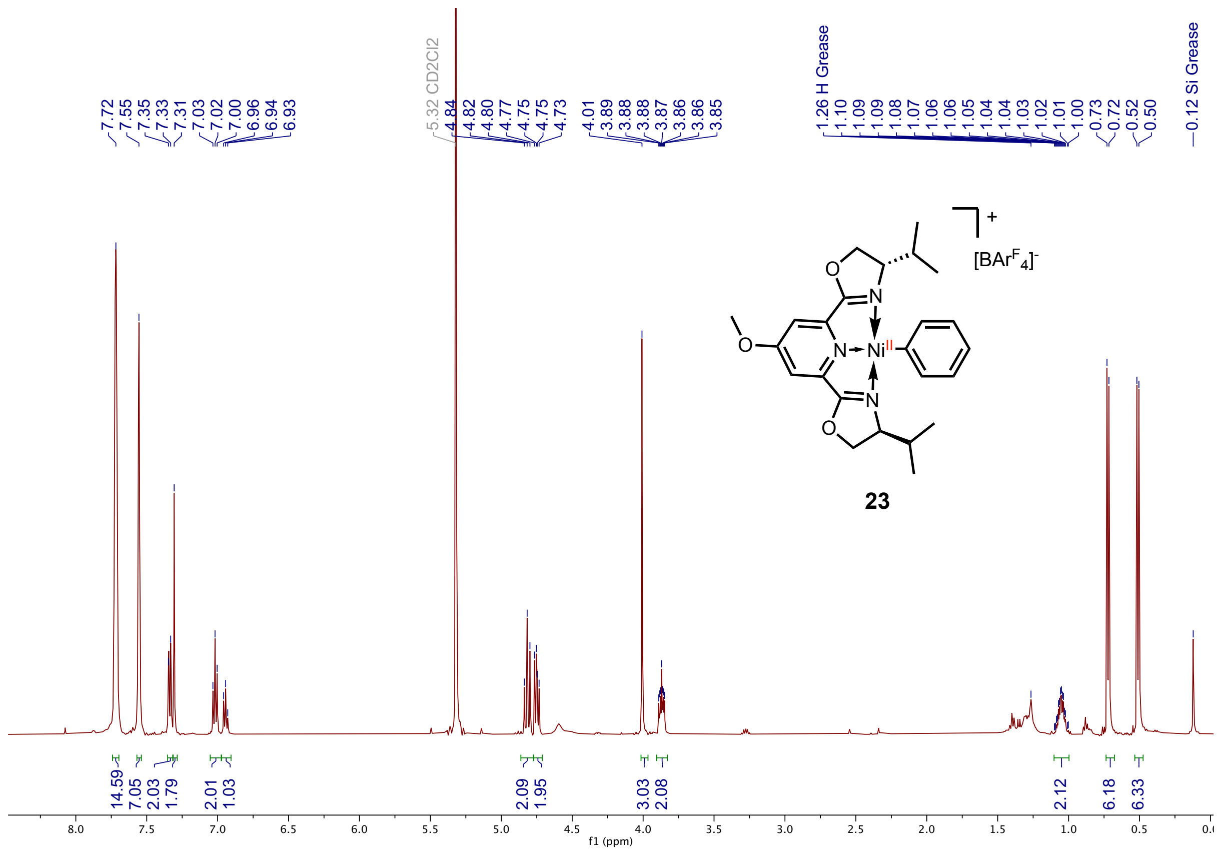

Figure S108. ^{1}H NMR (500 MHz, CD_2Cl_2, 25 °C) of [(4-OMe-^{iPr}pybox)Ni(Ph)]BAr$^F_4$ **23.**

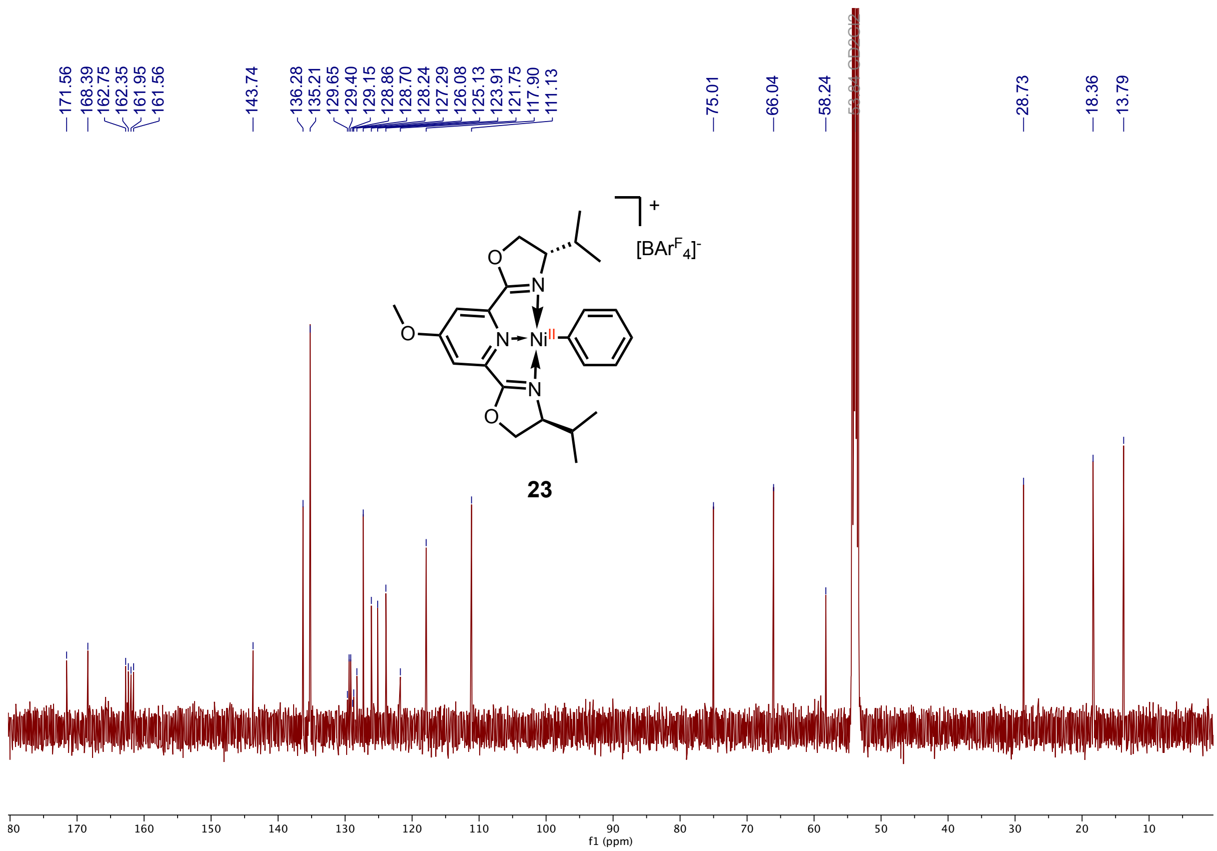

Figure S109. ^{13}C NMR (126 MHz, CD_2Cl_2, 25 °C) of [(4-OMe-^{iPr}pybox)Ni(Ph)]BAr^F_4 **23.**

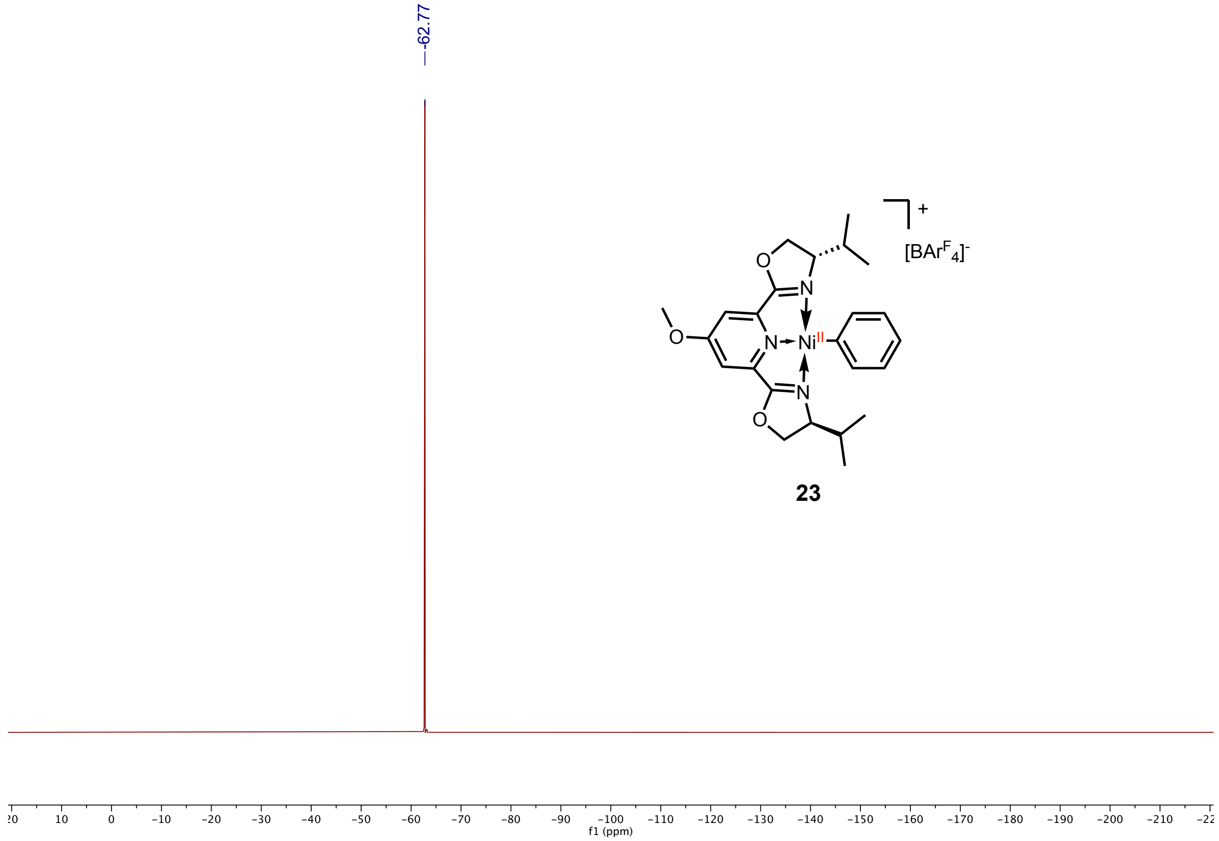

Figure S110. ^{19}F NMR (471 MHz, CD_2Cl_2, 25 °C) of [(4-OMe-^{iPr}pybox)Ni(Ph)]BAr$^F_4$ **23.**

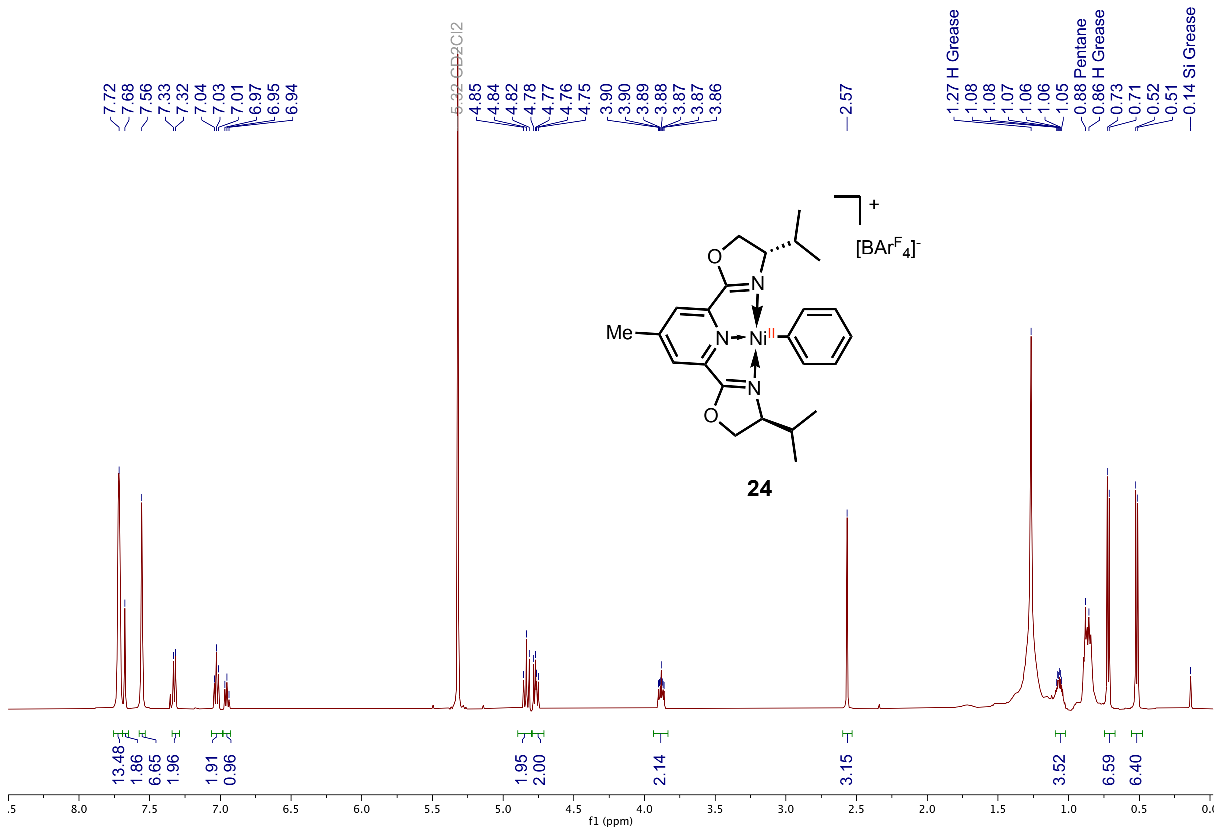

Figure S111. ^{1}H NMR (500 MHz, CD_2Cl_2, 25 °C) of [(4-Me-^{iPr}pybox)Ni(Ph)]BAr^F_4 **24.**

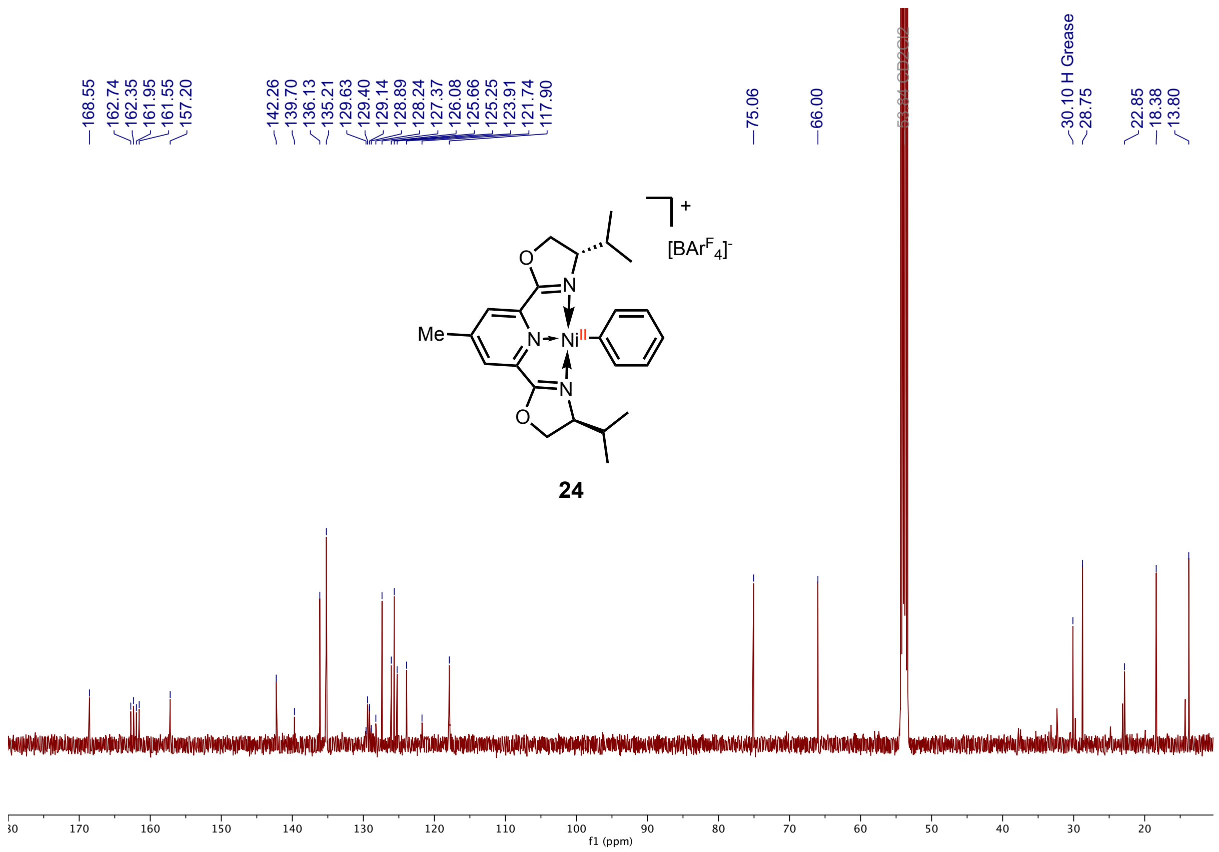

Figure S112. ^{13}C NMR (126 MHz, CD_2Cl_2, 25 °C) of [(4-Me-^{iPr}pybox)Ni(Ph)]BAr^F_4 **24.**

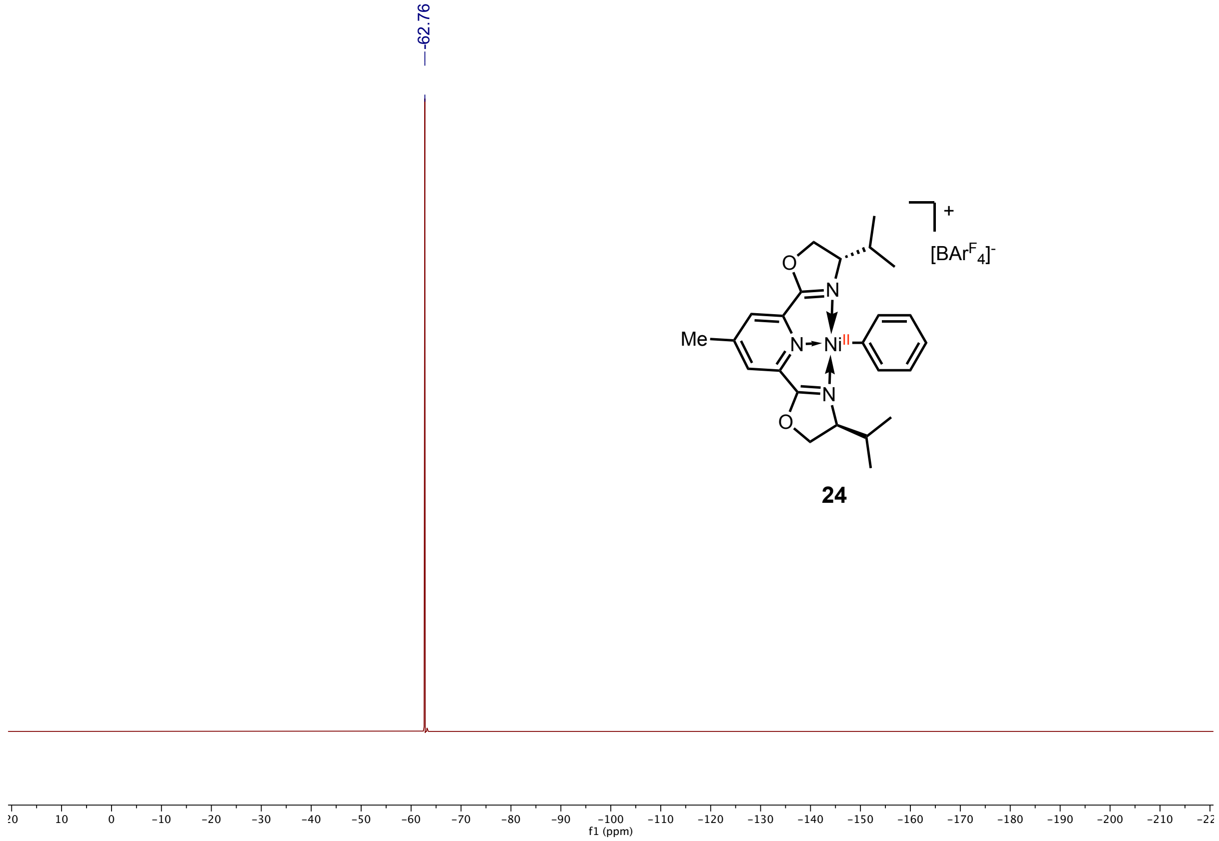

Figure S113. ^{19}F NMR (471 MHz, CD_2Cl_2, 25 °C) of [(4-Me-$^{i\mathrm{Pr}}$pybox)Ni(Ph)]BAr^F_4 **24.**

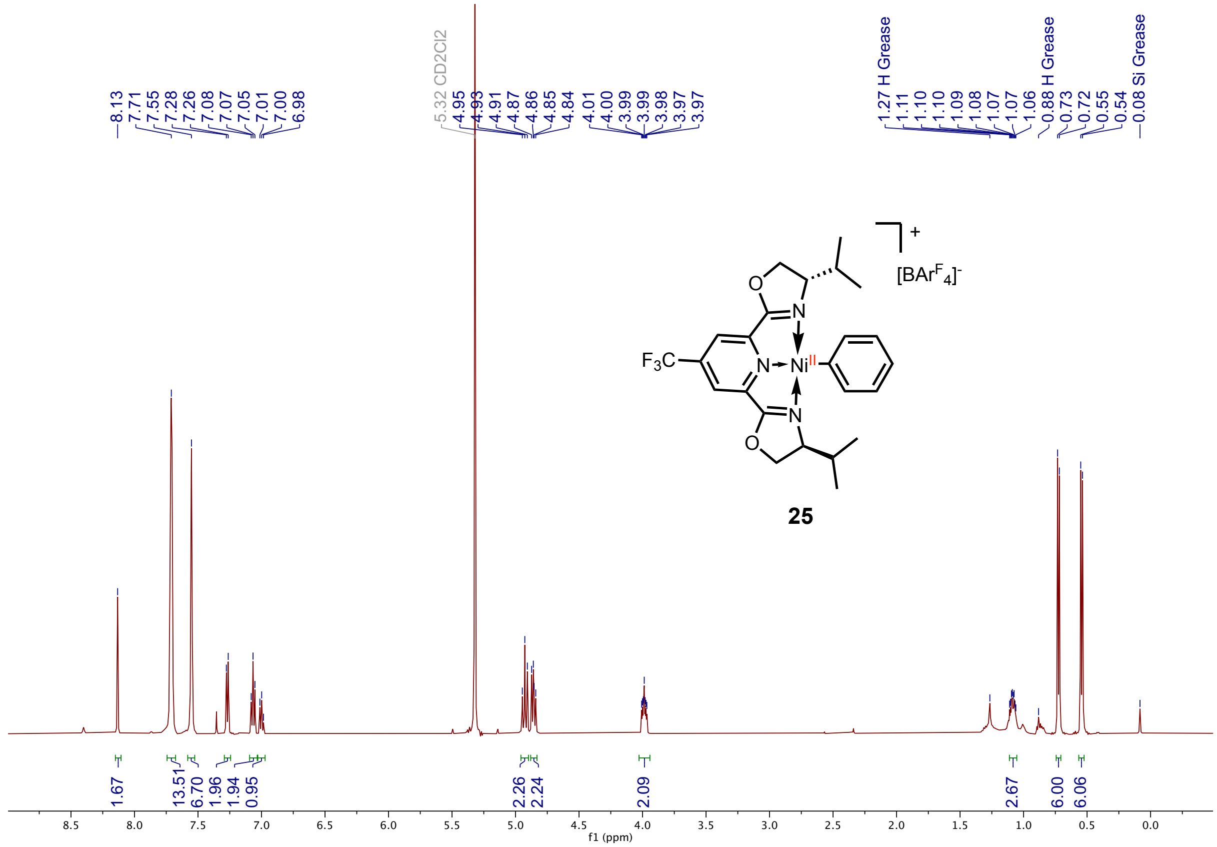

Figure S 114. ^{1}H NMR (500 MHz, CD_2Cl_2, 25 °C) of [(4-CF_3-^{iPr}pybox)Ni(Ph)]BAr^F_4 **25.**

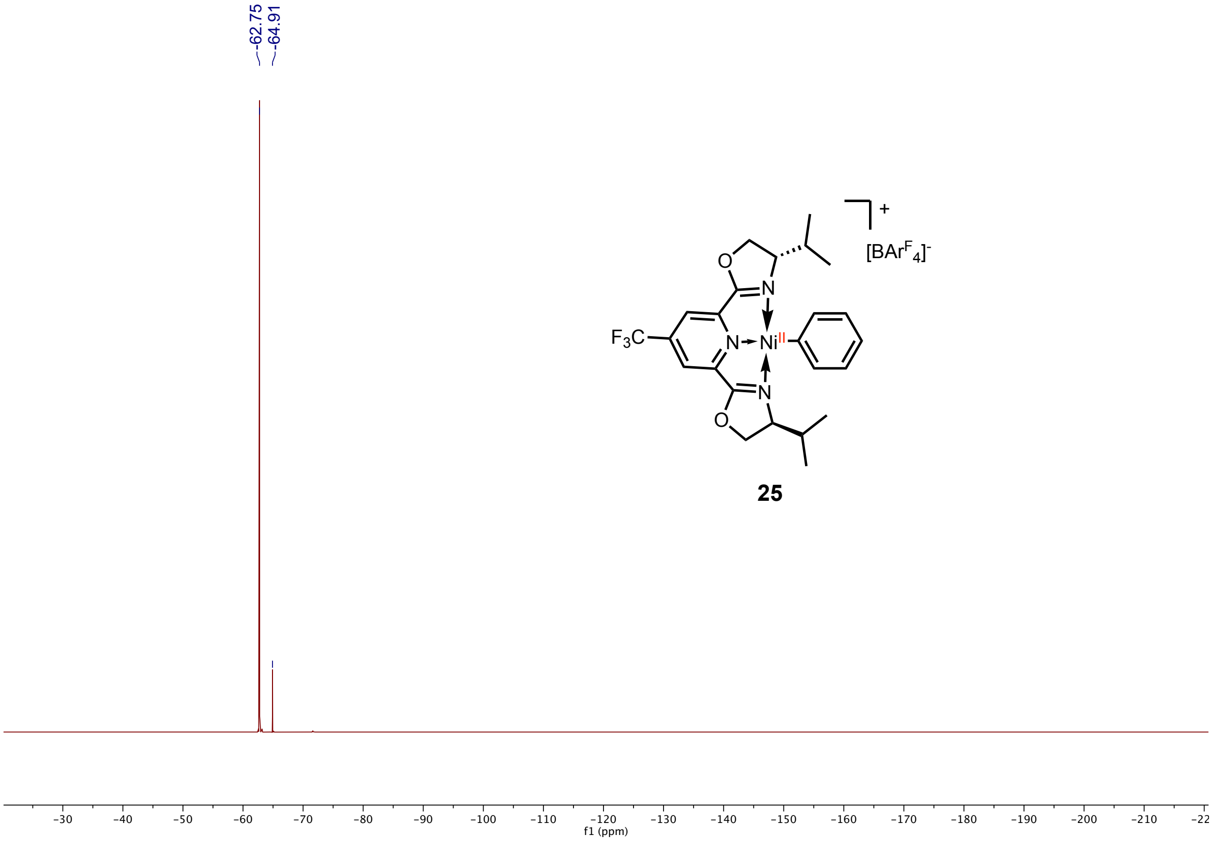

Figure S115. ^{19}F NMR (471 MHz, CD_2Cl_2, 25 °C) of [(4-CF_3-^{iPr}pybox)Ni(Ph)]BAr^F_4 **25.**

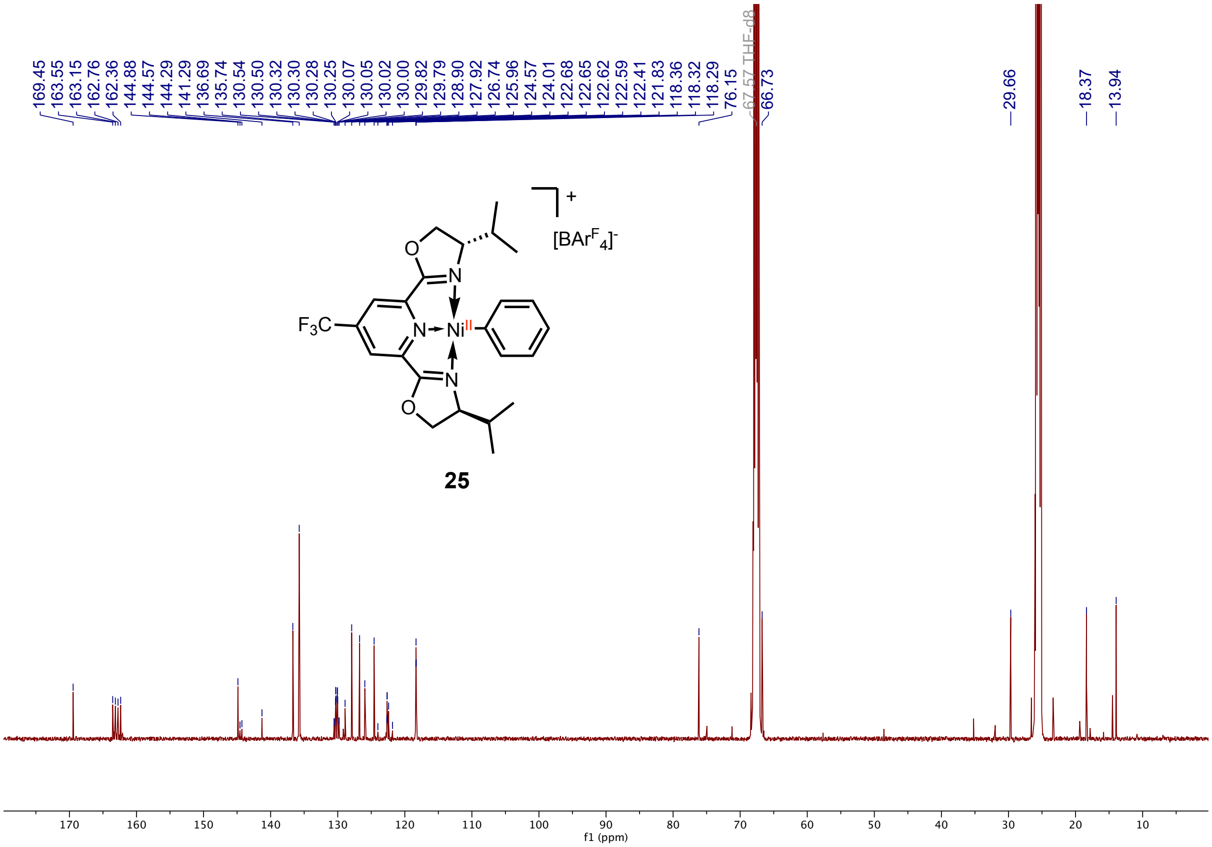

Figure S116. ^{13}C NMR (126 MHz, THF-*d*, 25 °C) of [(4-CF_3-^{iPr}pybox)Ni(Ph)]$BAr^F{}_4$ **25.**

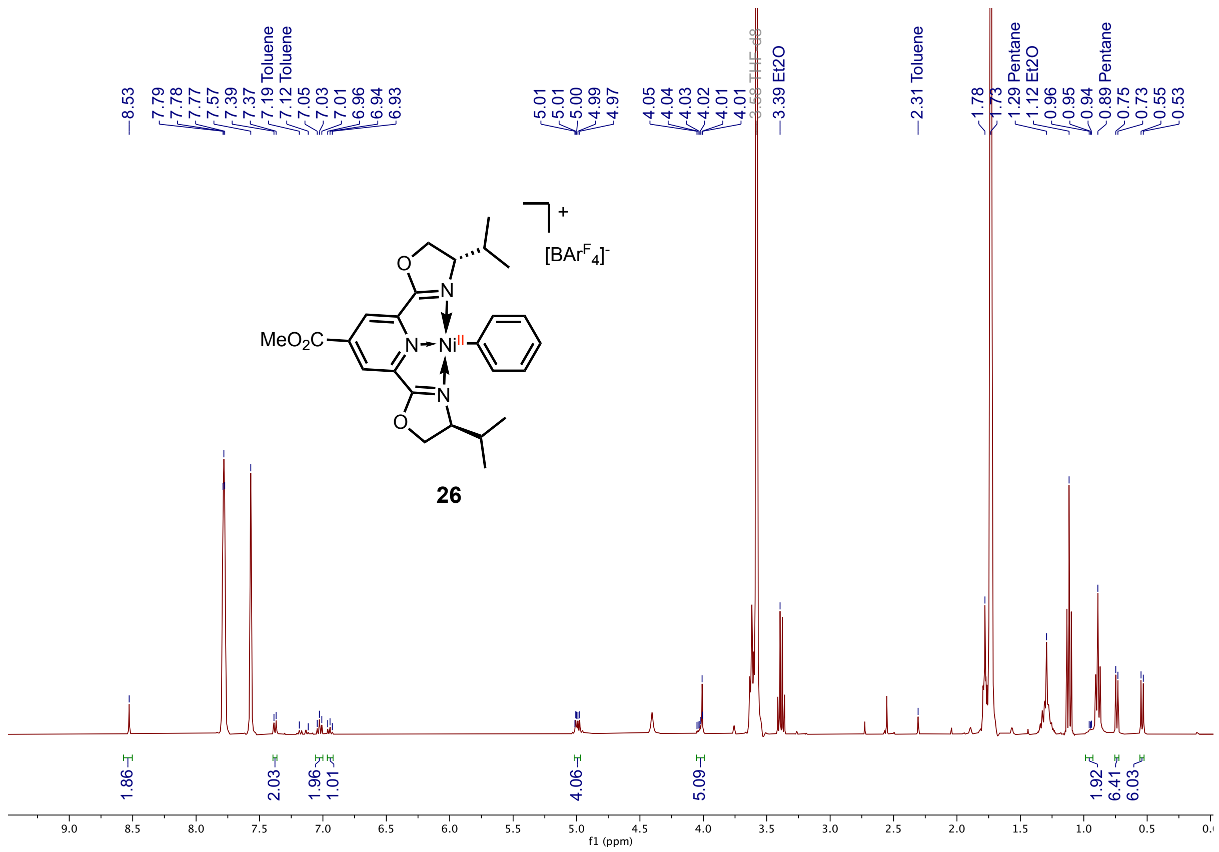

Figure S 117. ^{1}H NMR (400 MHz, THF-*d*, 25 °C) of [(4-CO_2Me-^{iPr}pybox)Ni(Ph)]BAr^F_4 **26.**

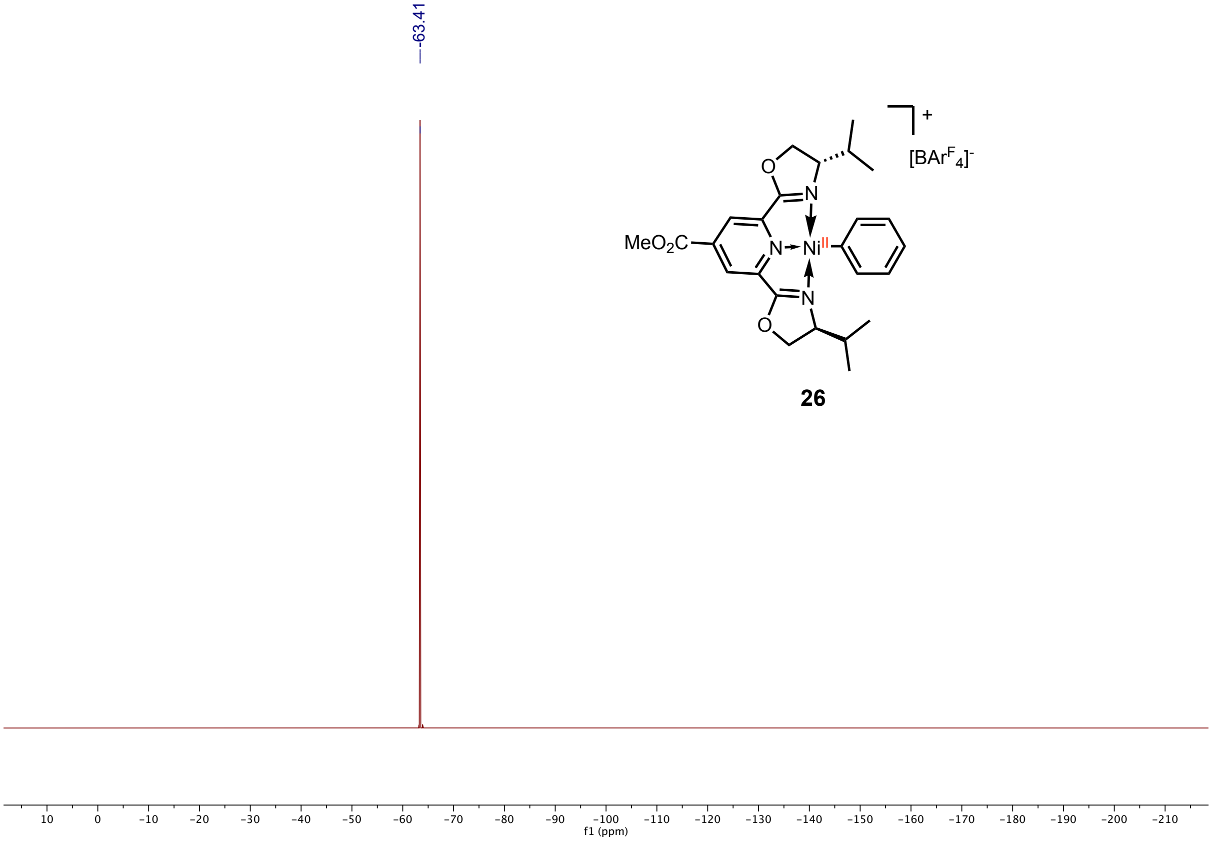

26

Figure S118. ^{19}F NMR (377 MHz, THF-*d*, 25 °C) of [(4-CO_2Me-^{iPr}pybox)Ni(Ph)]BAr^F_4 **26.**

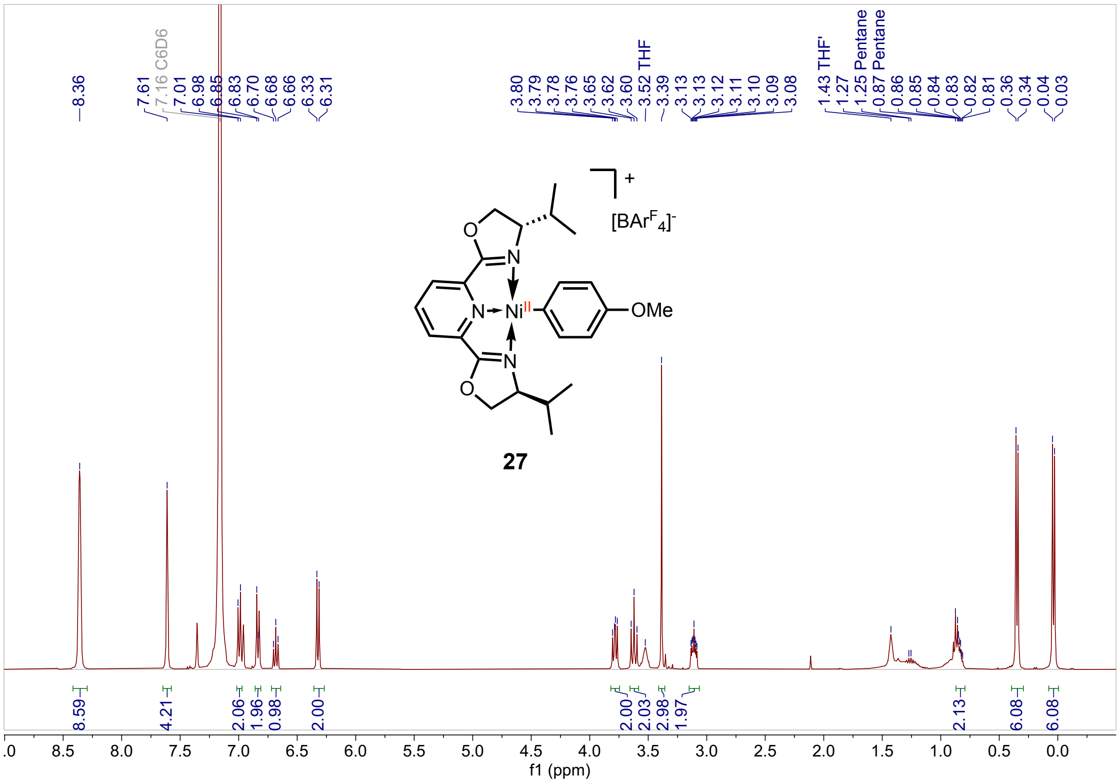

Figure S119. ^{1}H NMR (400 MHz, benzene-*d*, 25 °C) of [(^{iPr}pybox)Ni(*p*-MeO-C_6H_4)]BAr^F_4 **27.**

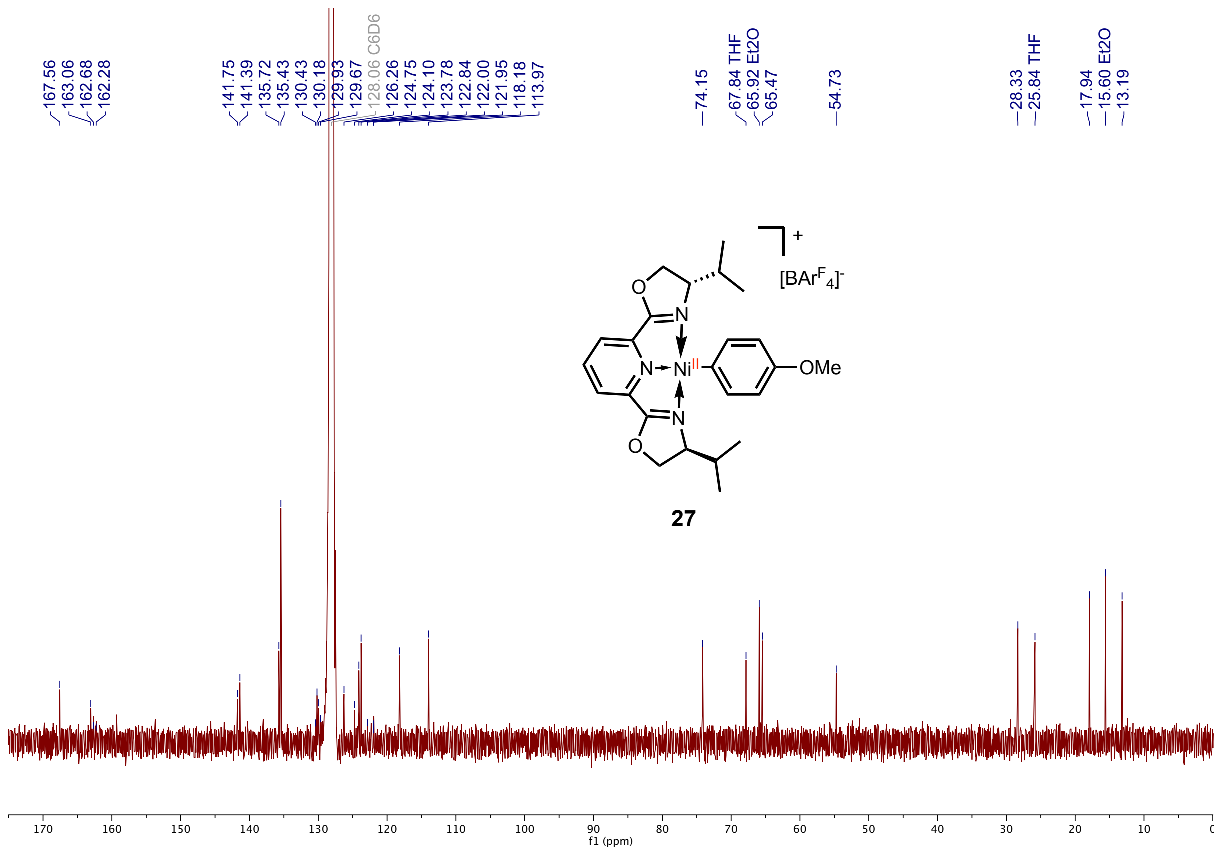

Figure S120. ^{13}C NMR (126 MHz, benzene-*d*, 25 °C) of [(^{iPr}pybox)Ni(*p*-MeO-C_6H_4)]BAr^F_4 **27.**

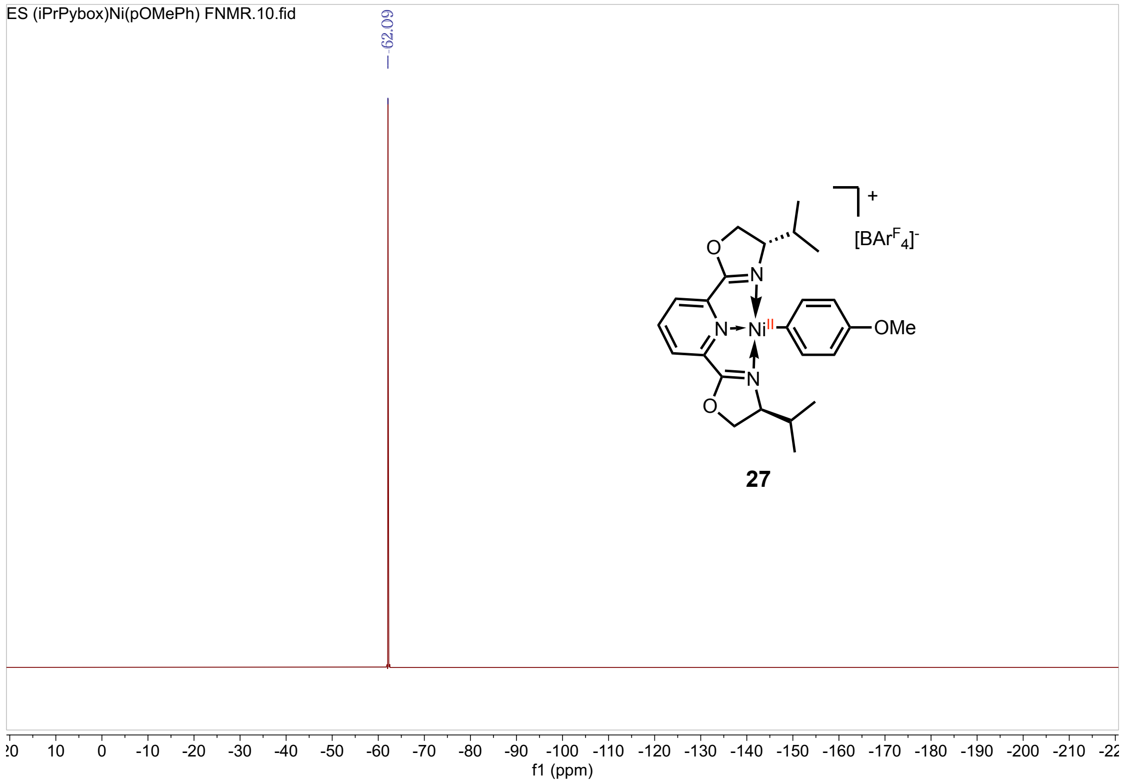

Figure S121. ^{19}F NMR (471 MHz, benzene-*d*, 25 °C) of [($^{i\text{Pr}}$pybox)Ni(*p*-MeO-C_6H_4)]BAr^F_4 **27.**

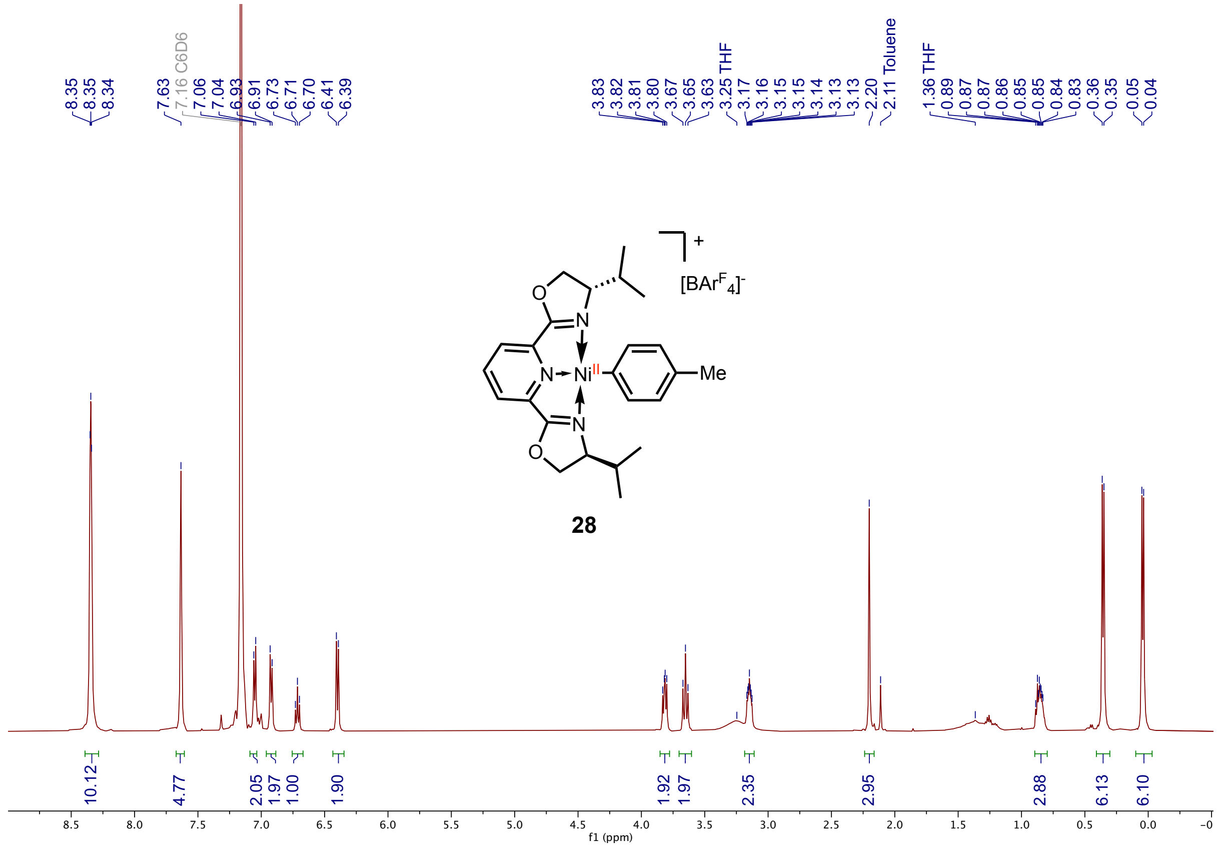

Figure S122. ^{1}H NMR (400 MHz, benzene-*d*, 25 °C) of [(^{iPr}pybox)Ni(*p*-tol)]BAr$^F_4$ **28.**

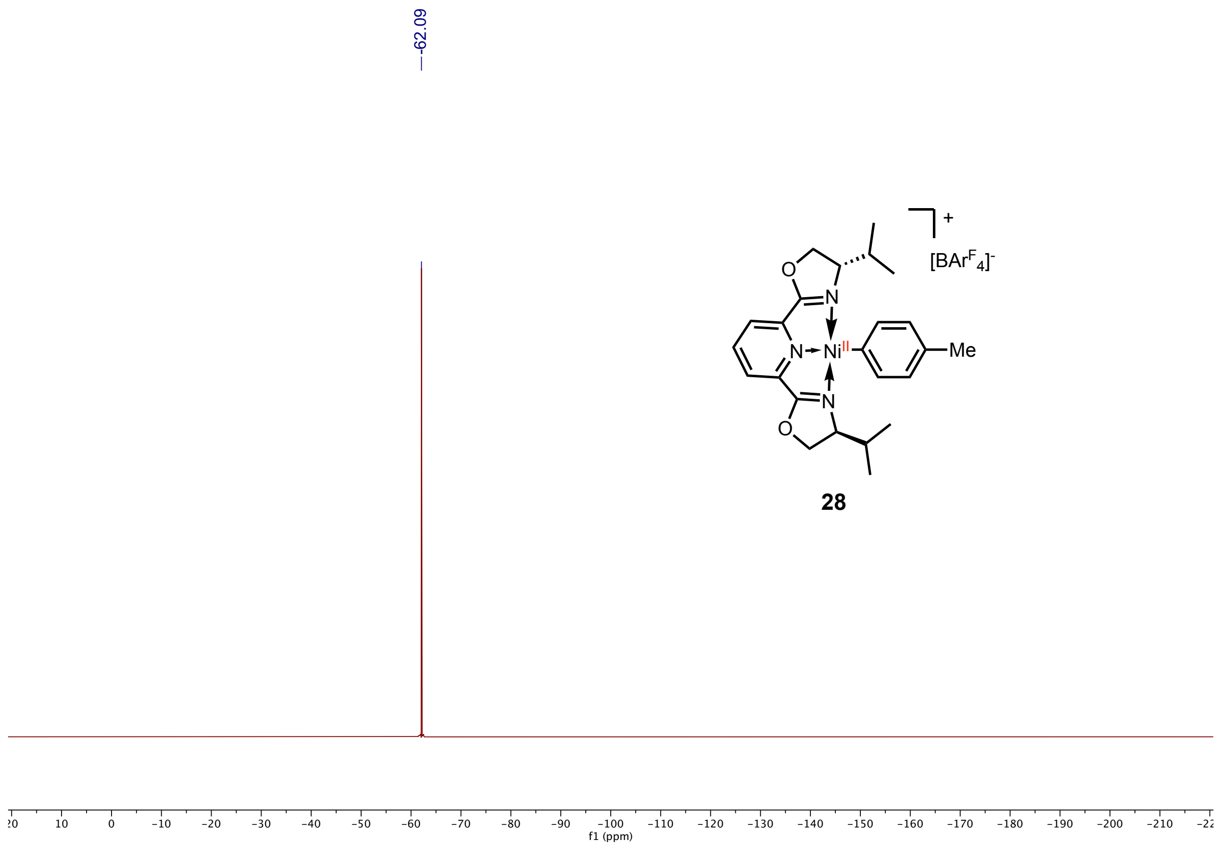

Figure S123. ^{19}F NMR (471 MHz, benzene-*d*, 25 °C) of [($^{i\text{Pr}}$pybox)Ni(*p*-tol)]BAr$^{F}_{4}$ **28.**

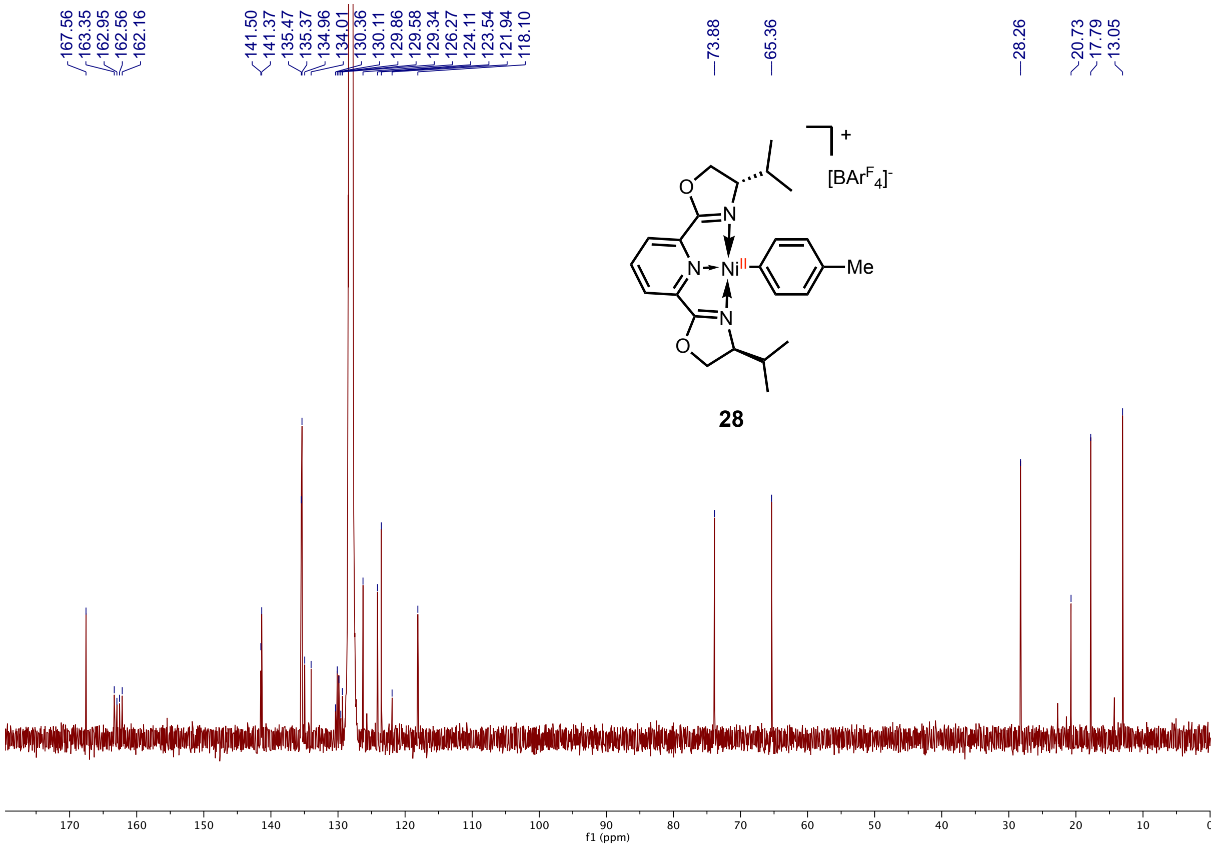

Figure S124. ^{13}C NMR (126 MHz, d-benzene, 25 °C) of [(^{iPr}pybox)Ni(*p*-tol)]BAr$^F_4$ **28.**

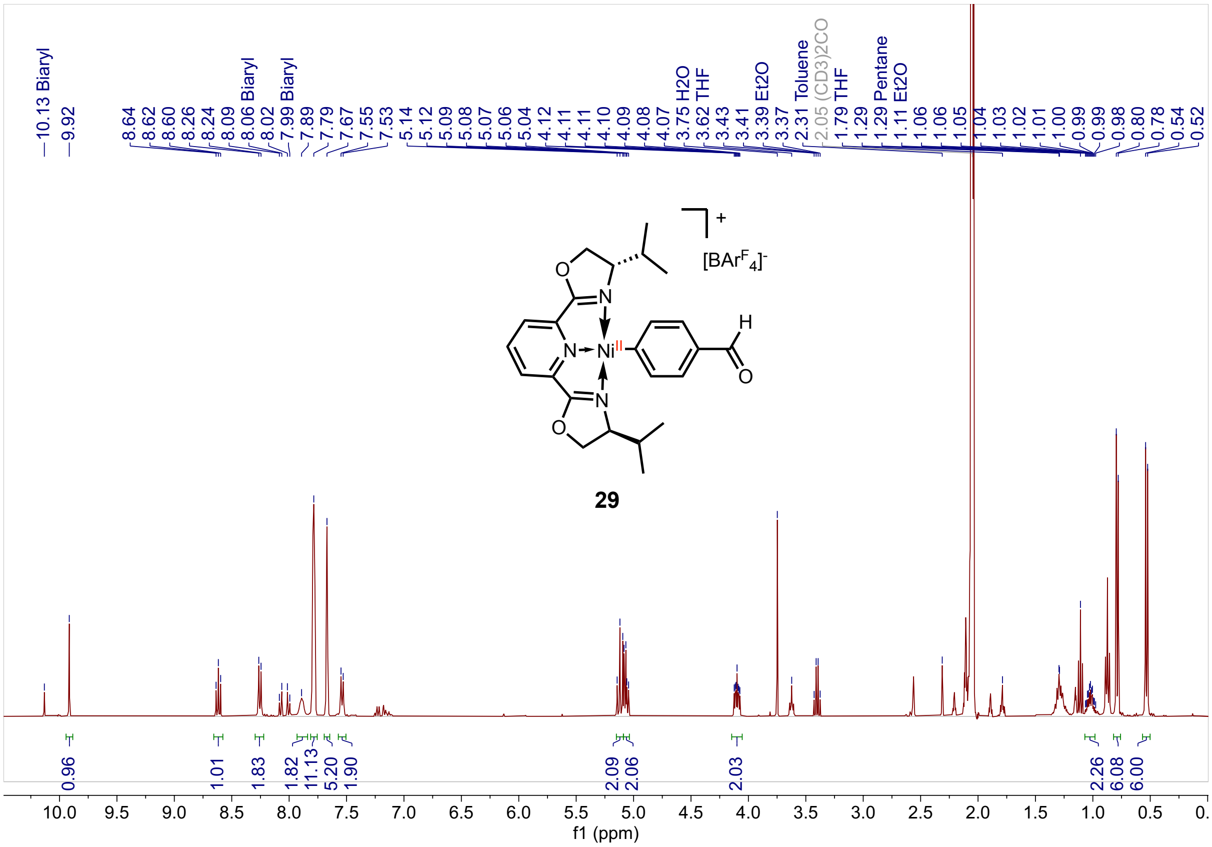

Figure S125. ^{1}H NMR (400 MHz, acetone-*d*, 25 °C) of [($^{i\text{Pr}}$pybox)Ni(*p*-CHO-C_6H_4)]BAr^F_4 **29.**

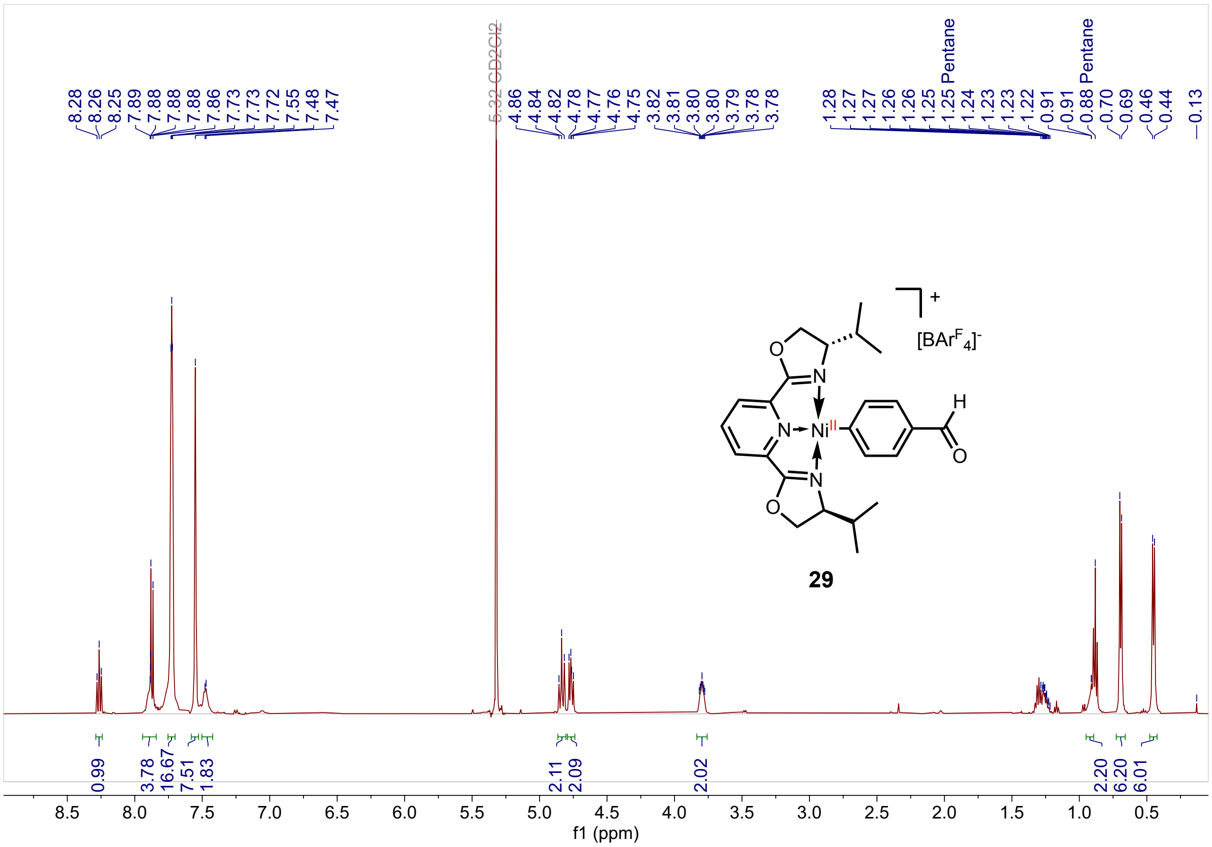

Figure S126. ^{1}H NMR (500 MHz, CD_2Cl_2, 25 °C) of [(^{iPr}pybox)Ni(*p*-CHO-C_6H_4)]BAr^F_4 **29.**

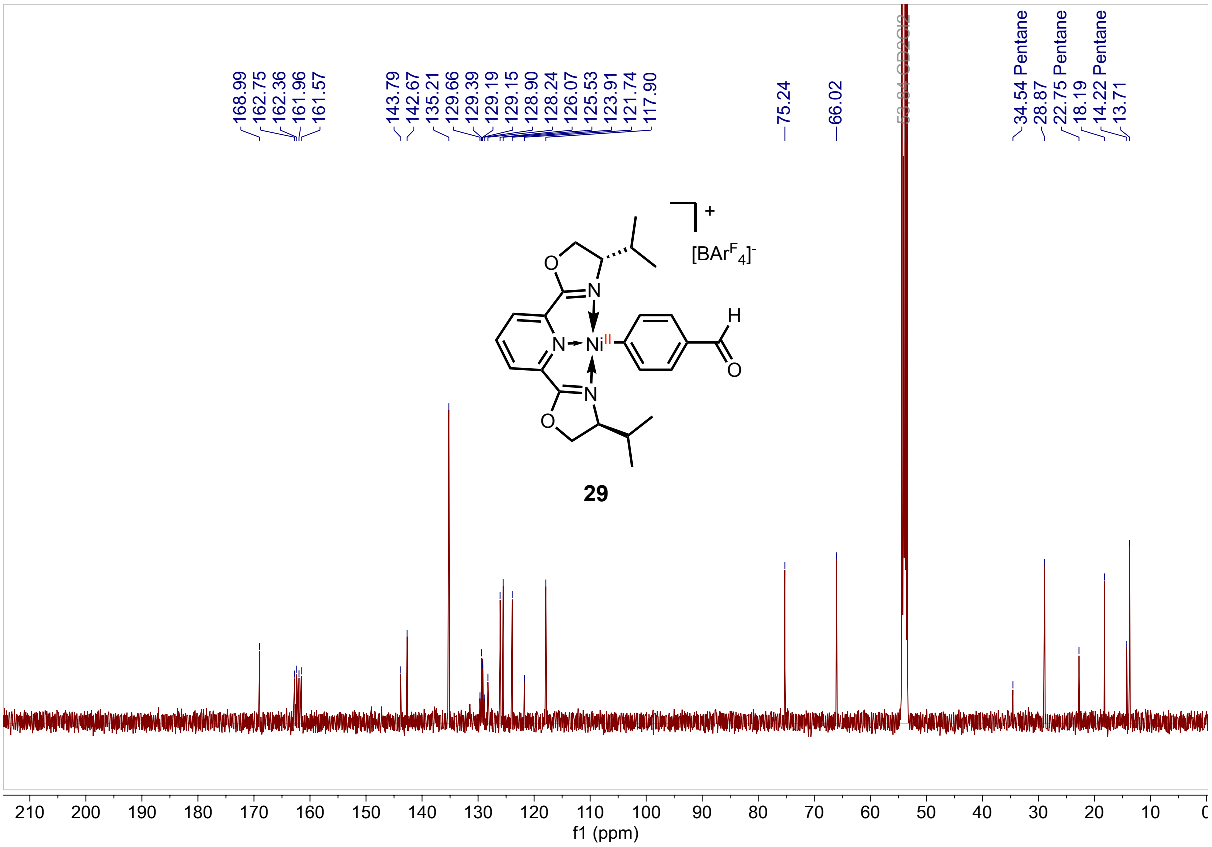

Figure S127. ^{13}C NMR (126 MHz, CD_2Cl_2, 25 °C) of [(^{iPr}pybox)Ni(p-CHO-C_6H_4)]BAr$^F_4$ **29.**

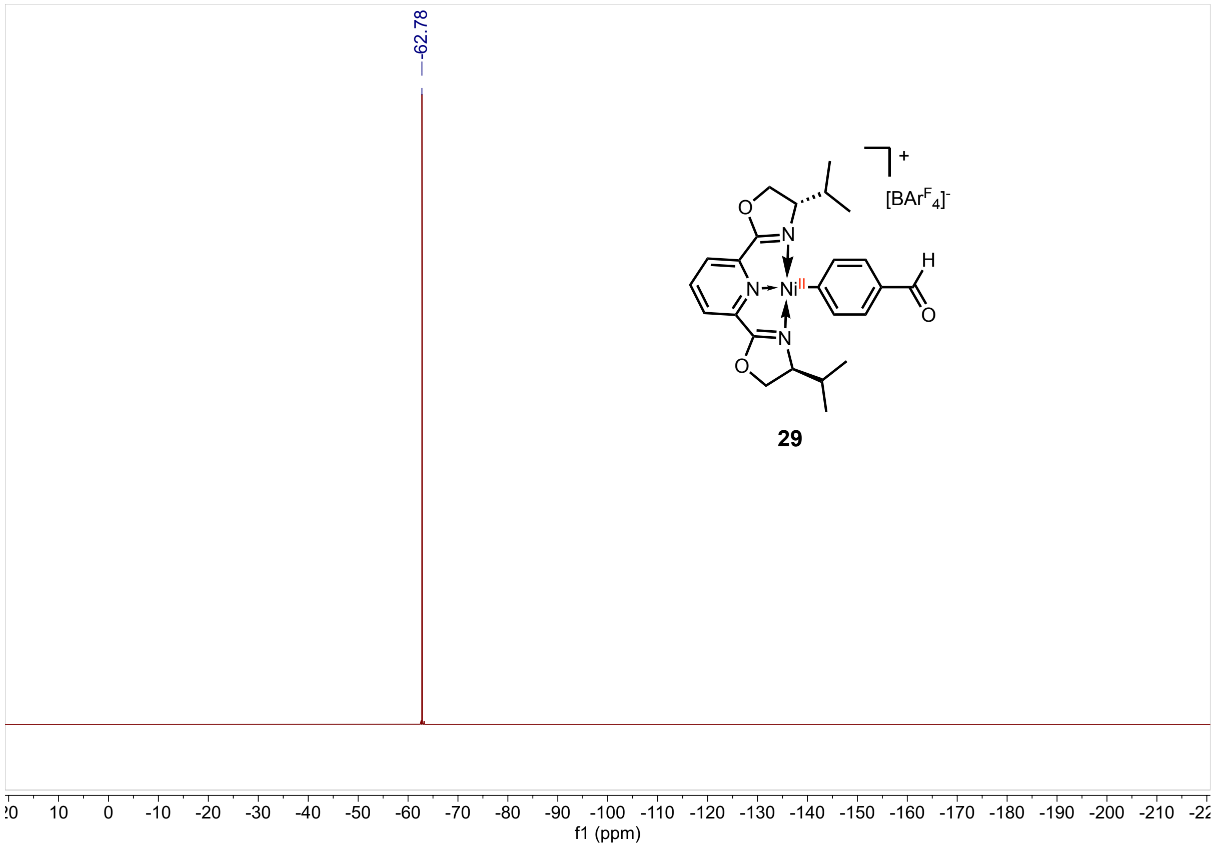

Figure S128. ^{19}F NMR (471 MHz, CD_2Cl_2, 25 °C) of [(^{iPr}pybox)Ni(*p*-CHO-C_6H_4)]BAr^F_4 **29.**

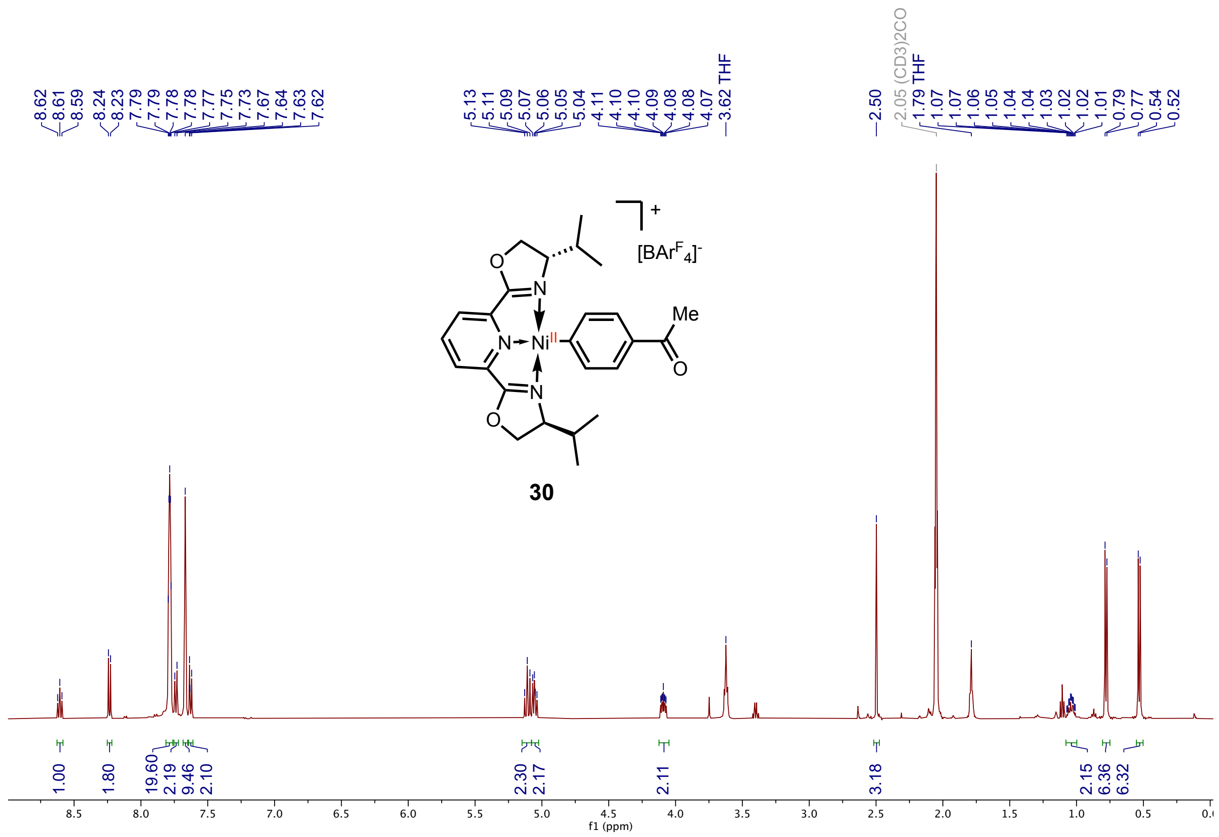

Figure S129. ^{1}H NMR (500 MHz, acetone-*d*, 25 °C) of [($^{i\text{Pr}}$pybox)Ni(*p*-COMe-C_6H_4)]BAr^F_4 **30.**

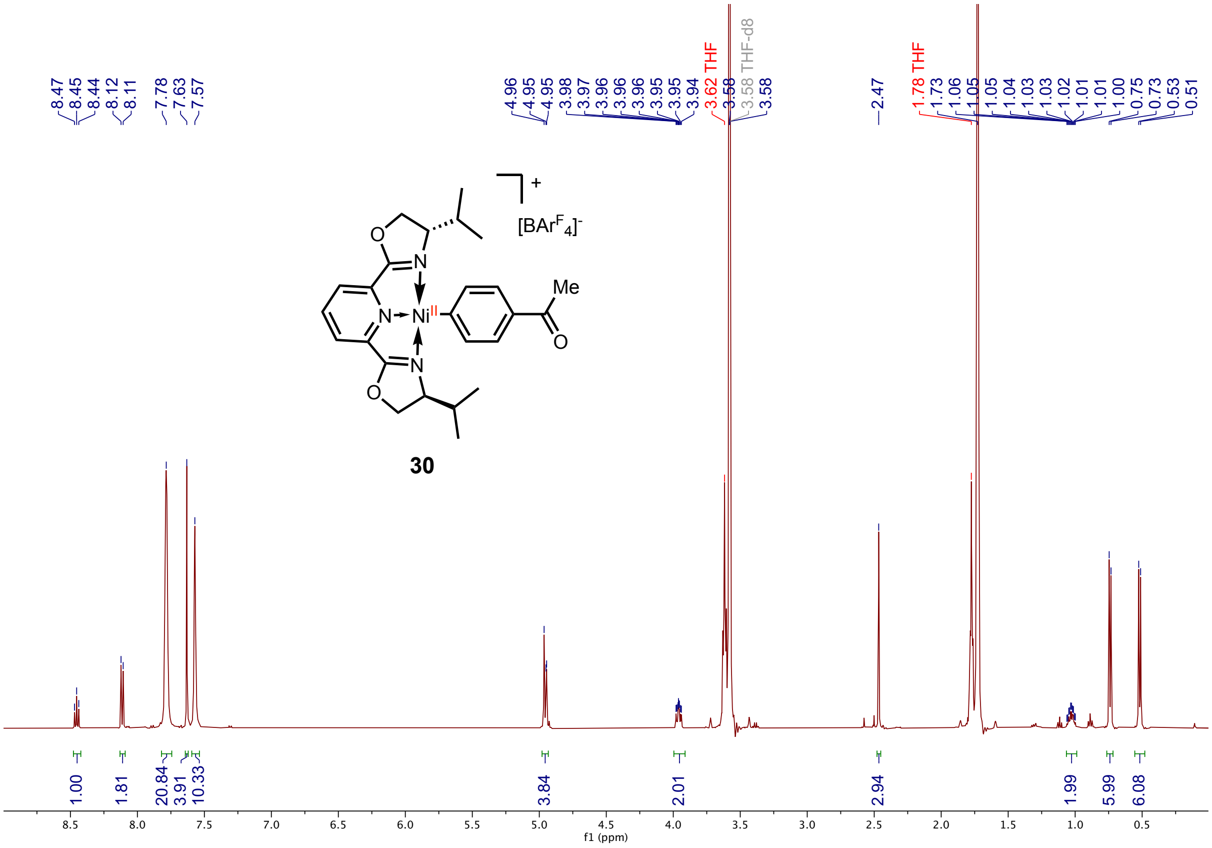

Figure S130. ^{1}H NMR (500 MHz, THF-*d*, 25 °C) of [(^{iPr}pybox)Ni(*p*-COMe-C_6H_4)]$BAr^F{}_4$ **30.**

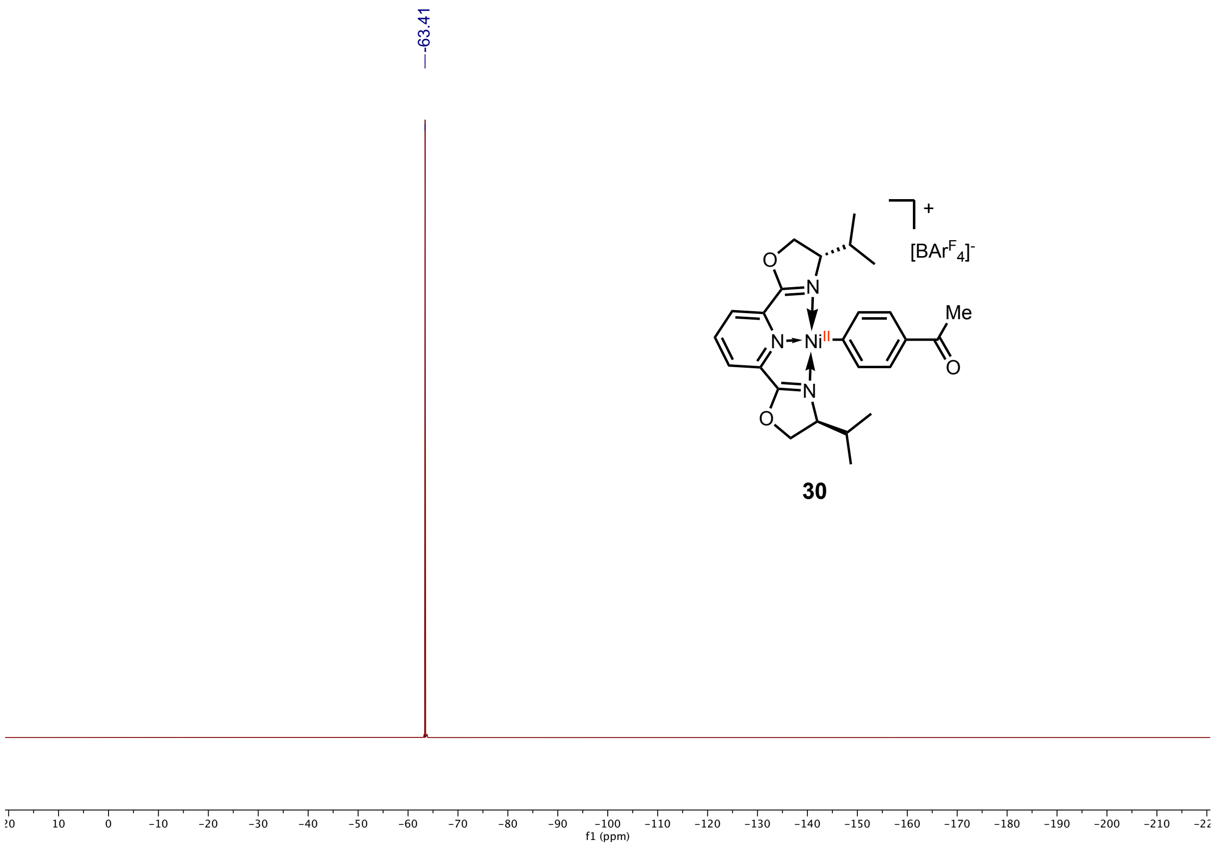

Figure S131. ^{19}F NMR (471 MHz, THF-*d*, 25 °C) of [($^{i\mathrm{Pr}}$pybox)Ni(*p*-COMe-C_6H_4)]BAr$^F{}_4$ **30.**

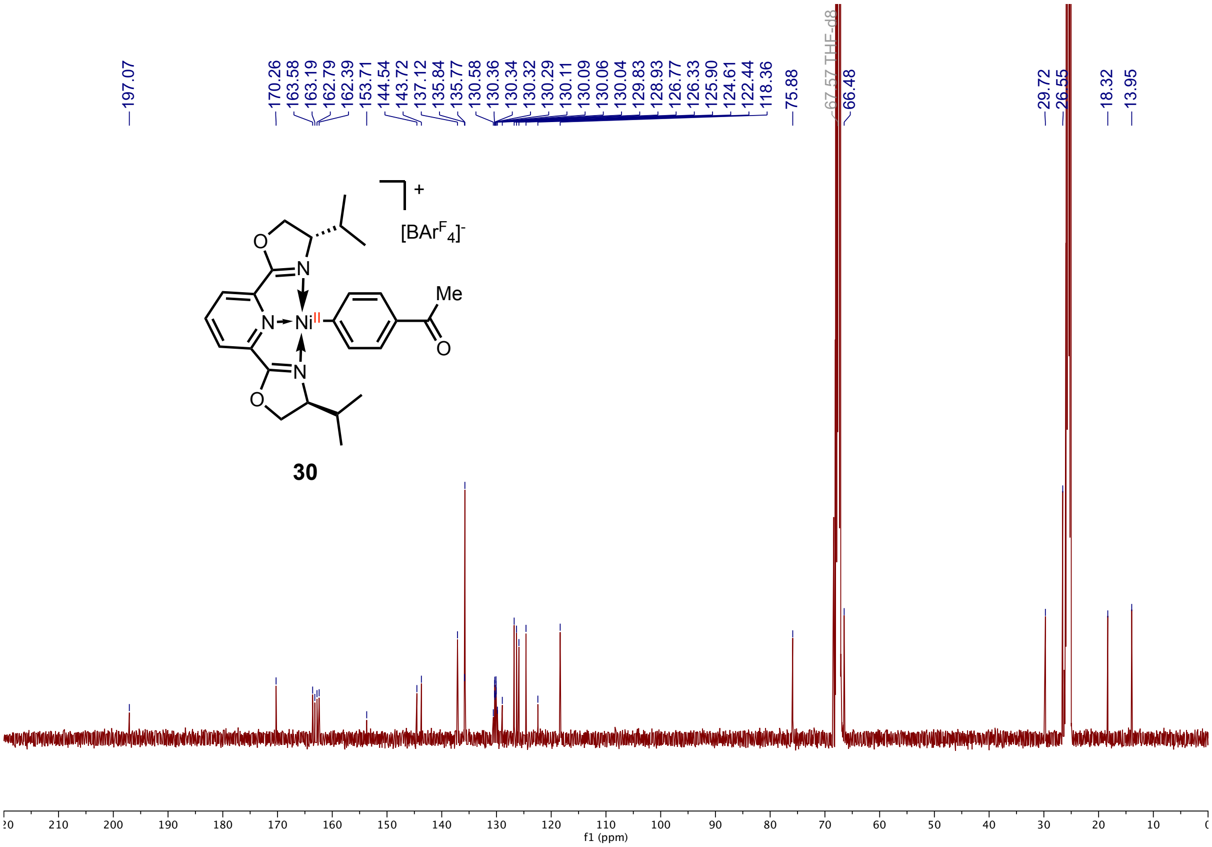

Figure S132. ^{13}C NMR (126 MHz, d-THF, 25 °C) of [(^{iPr}pybox)Ni(p-COMe-C_6H_4)]BAr^F_4 **30.**

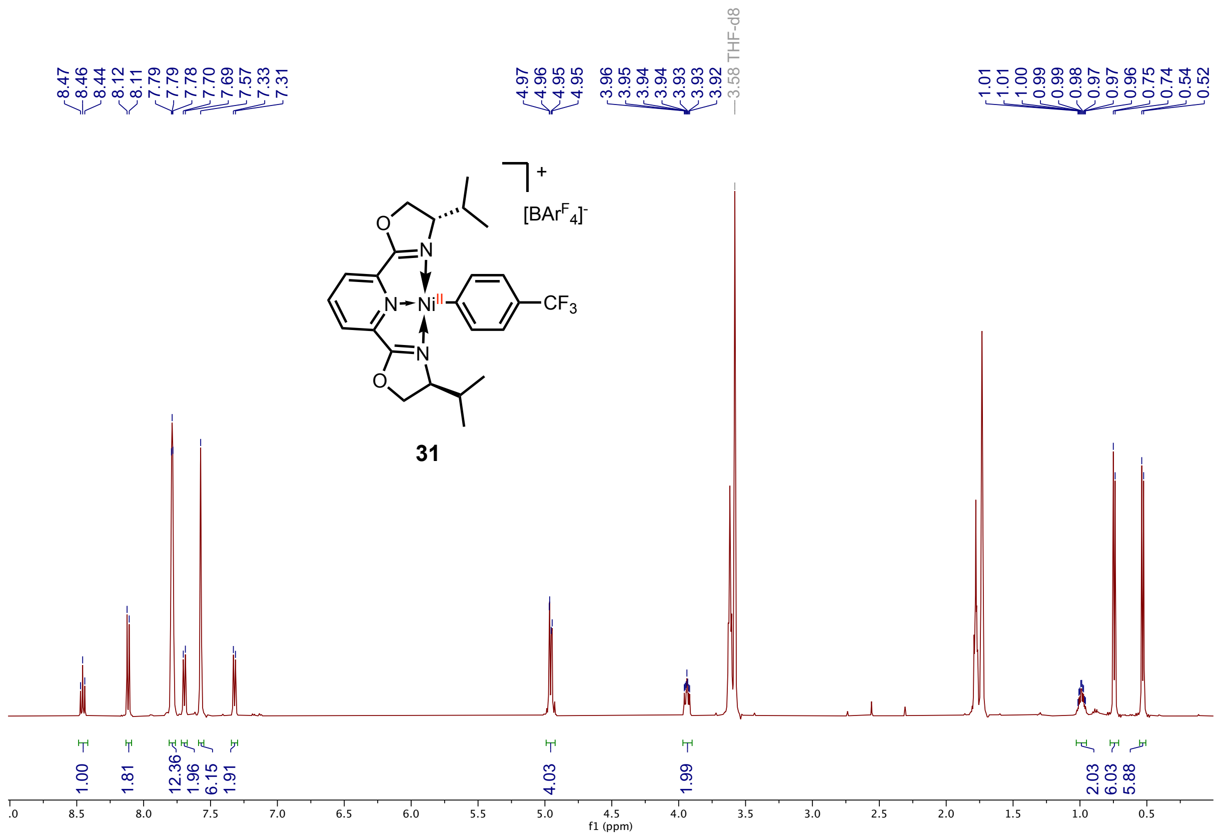

Figure S133. ^{1}H NMR (500 MHz, THF-*d*, 25 °C) of [($^{i\text{Pr}}$pybox)Ni(*p*-CF_3-C_6H_4)]BAr^F_4 **31.**

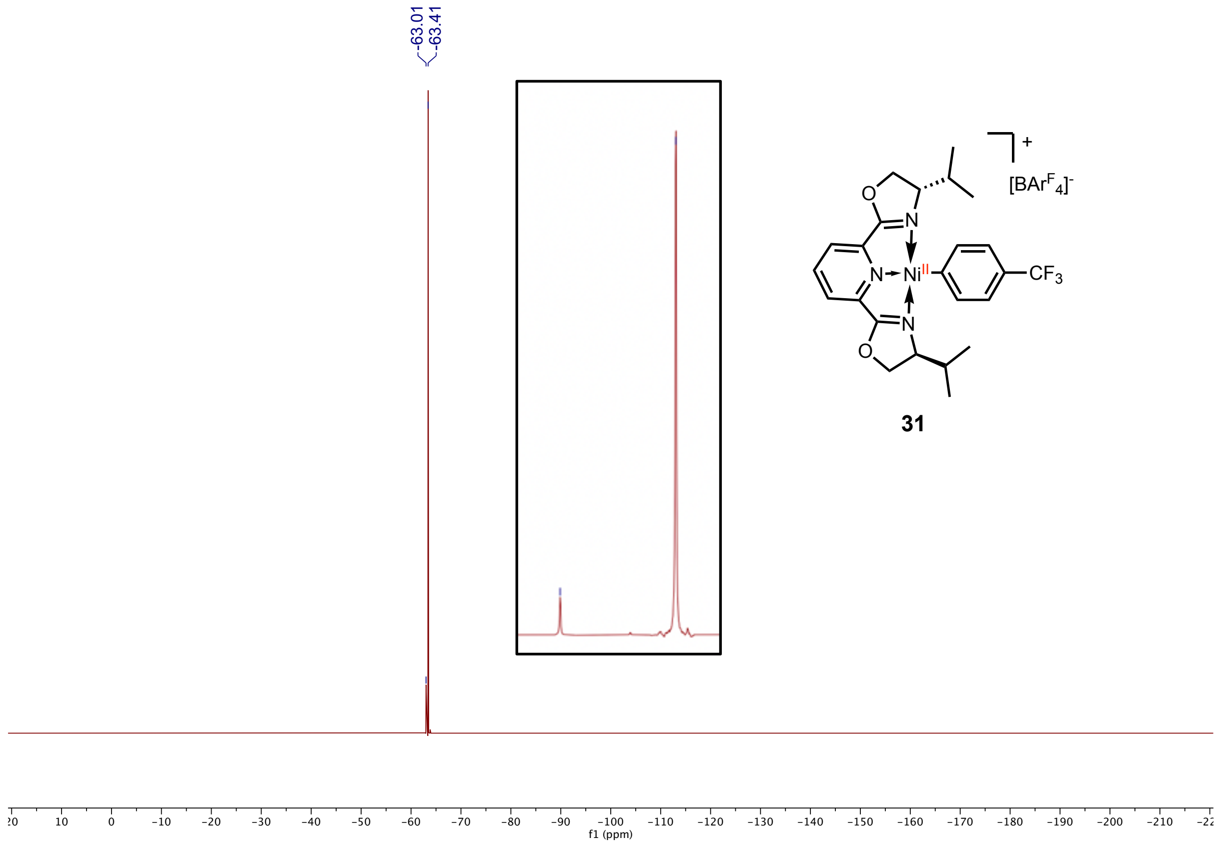

Figure S134. ^{19}F NMR (471 MHz, THF-*d*, 25 °C) of [($^{i\text{Pr}}$pybox)Ni(*p*-CF$_3$-C$_6$H$_4$)]BAr$^F_4$ **31.**

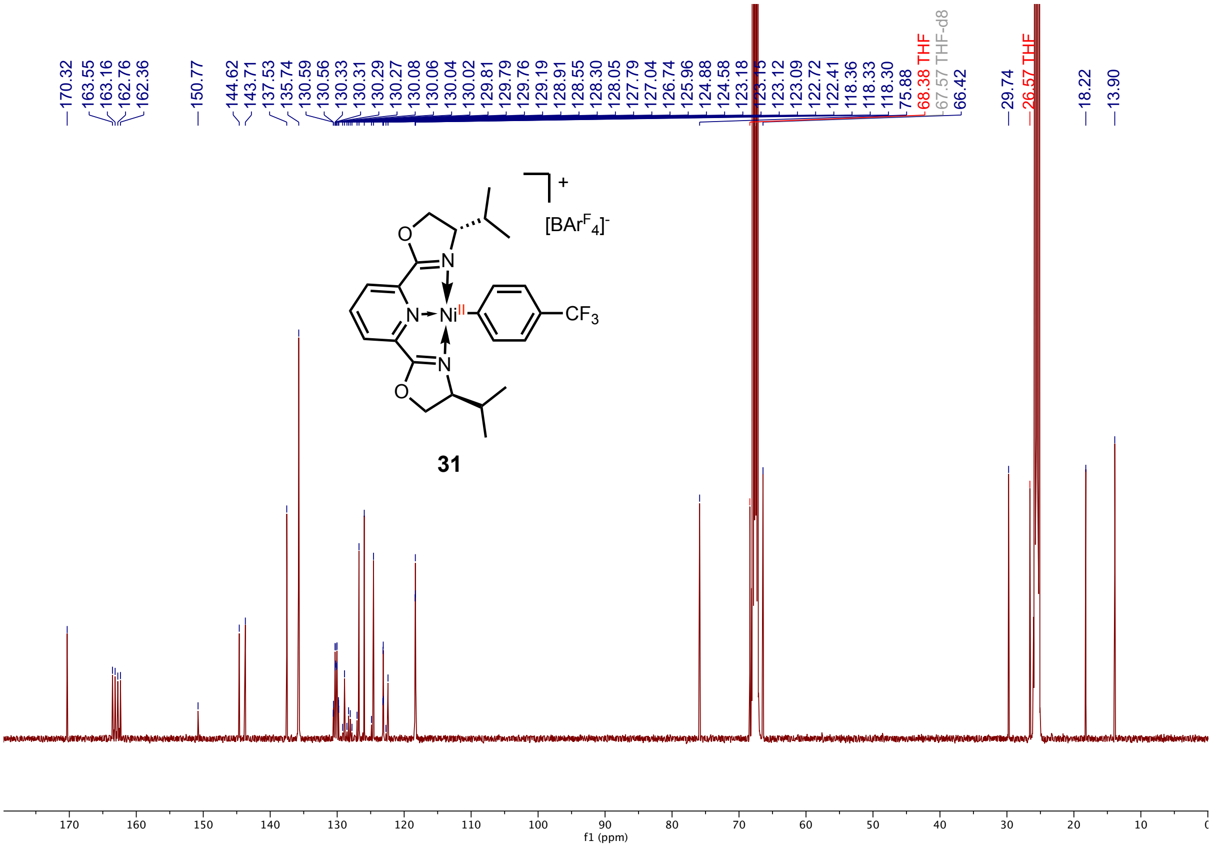

Figure S135. ^{13}C NMR (126 MHz, THF-*d*, 25 °C) of [(^{iPr}pybox)Ni(*p*-CF_3-C_6H_4)]BAr^F_4 **31.**

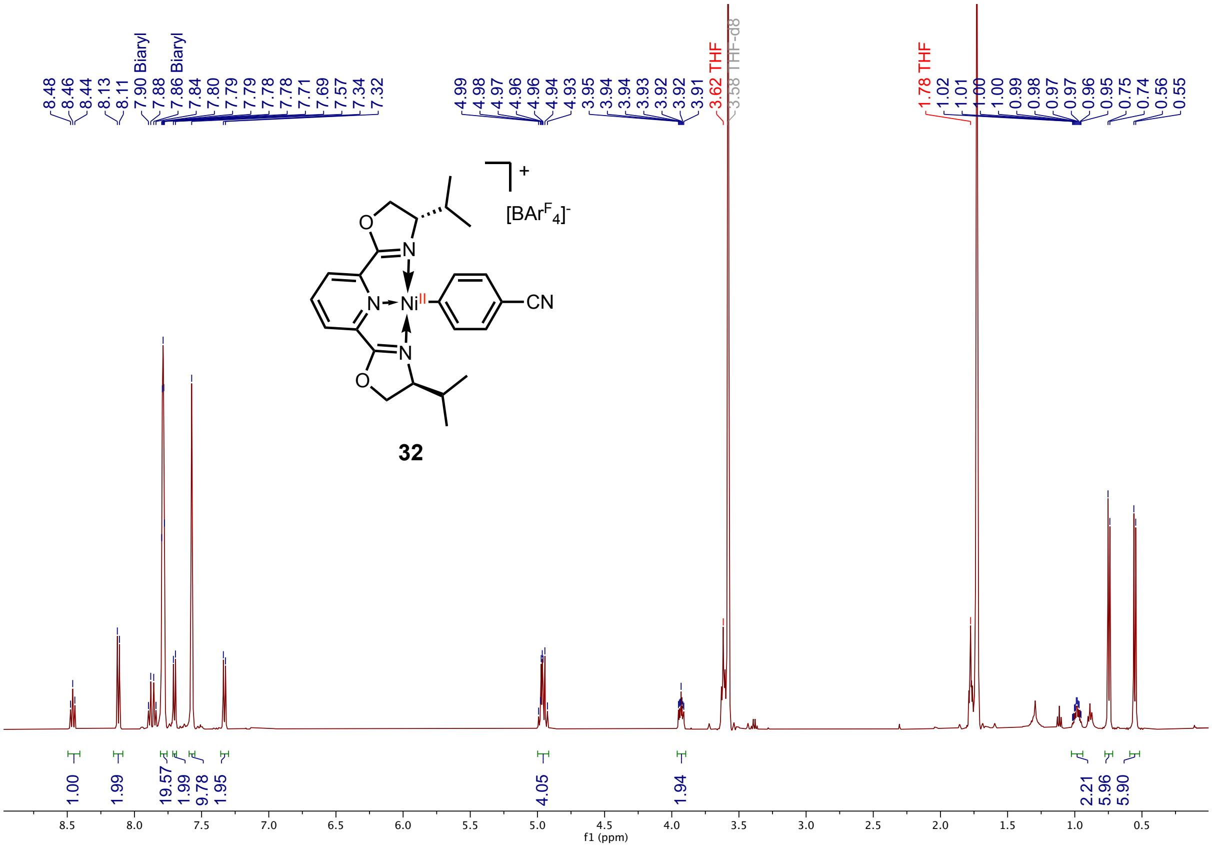

Figure S136. ^{1}H NMR (500 MHz, THF-*d*, 25 °C) of [($^{i\text{Pr}}$pybox)Ni(*p*-CN-C_6H_4)]BAr^F_4 **32.**

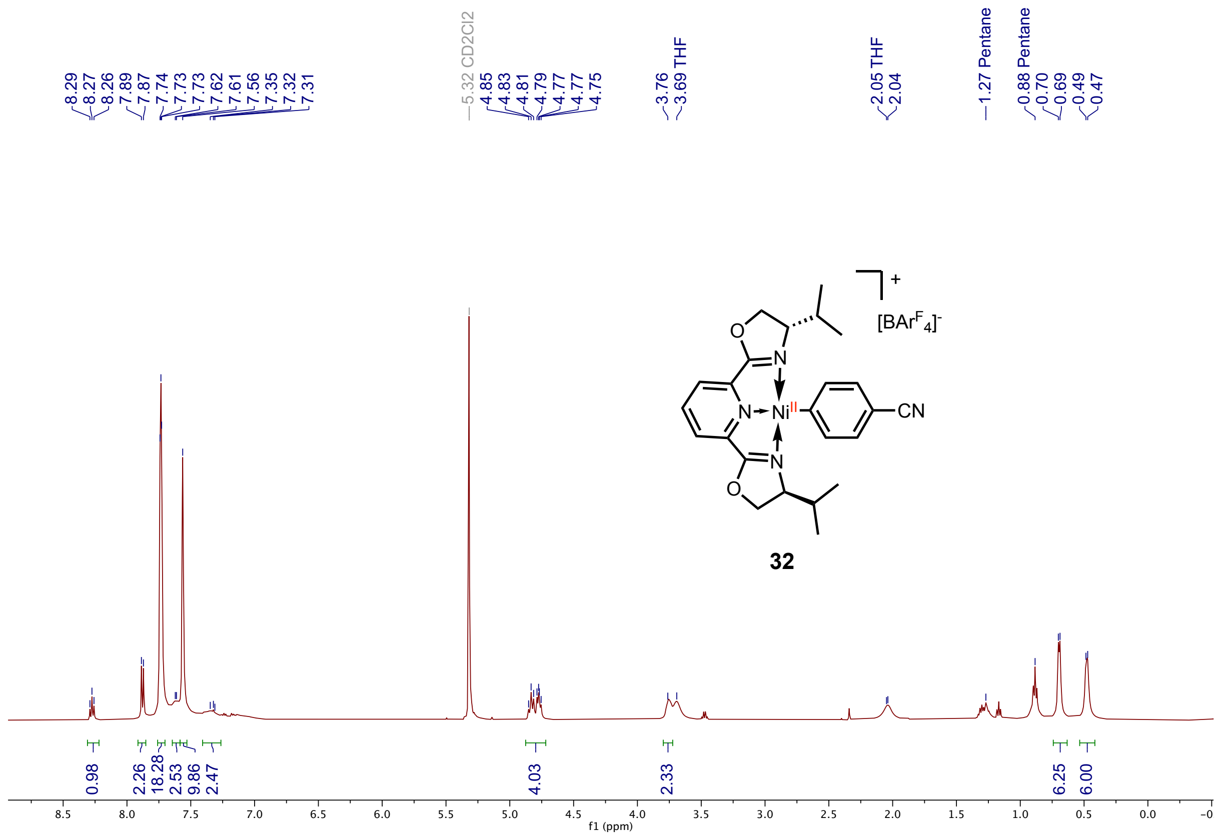

Figure S137. ^{1}H NMR (500 MHz, CD_2Cl_2, 25 °C) of [(^{iPr}pybox)Ni(*p*-CN-C_6H_4)]BAr^F_4 **32.**

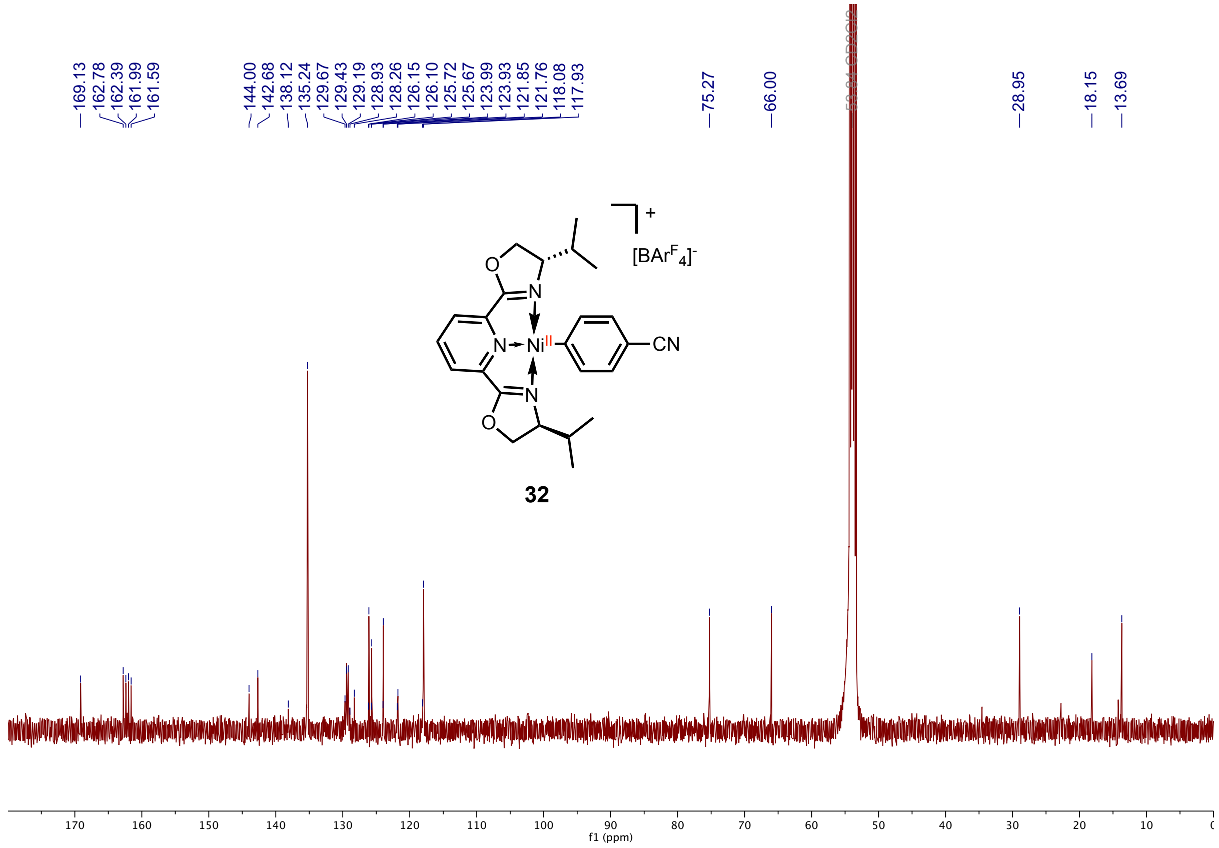

Figure S138. ^{13}C NMR (126 MHz, CD_2Cl_2, 25 °C) of [($^{i\text{Pr}}$pybox)Ni(*p*-CN-C_6H_4)]BAr$^F{}_4$ **32.**

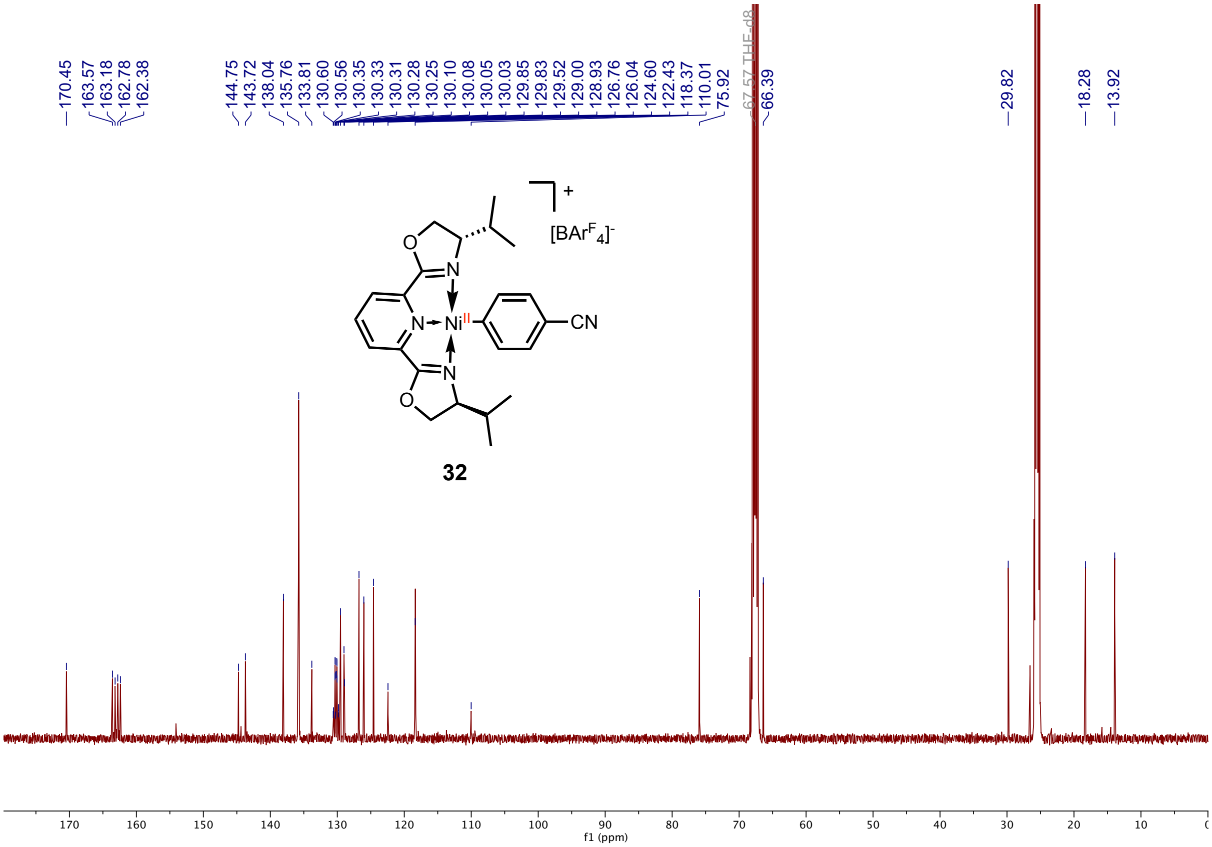

Figure S139. ^{13}C NMR (126 MHz, THF-*d*, 25 °C) of [(^{iPr}pybox)Ni(*p*-CN-C_6H_4)]BAr^F_4 **32.**

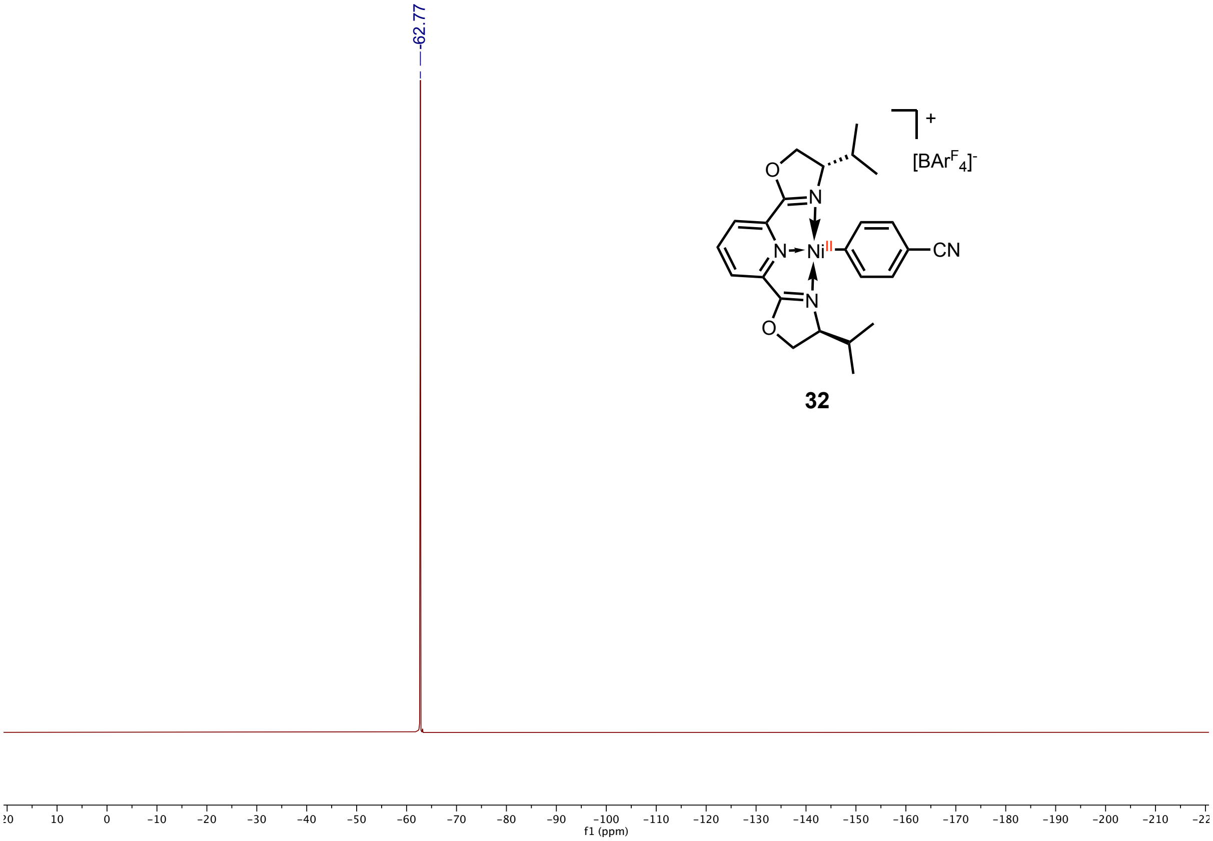

Figure S140. ^{19}F NMR (471 MHz, CD_2Cl_2, 25 °C) [(^{iPr}pybox)Ni(*p*-CN-C_6H_4)]BAr$^F_4$ **32.**

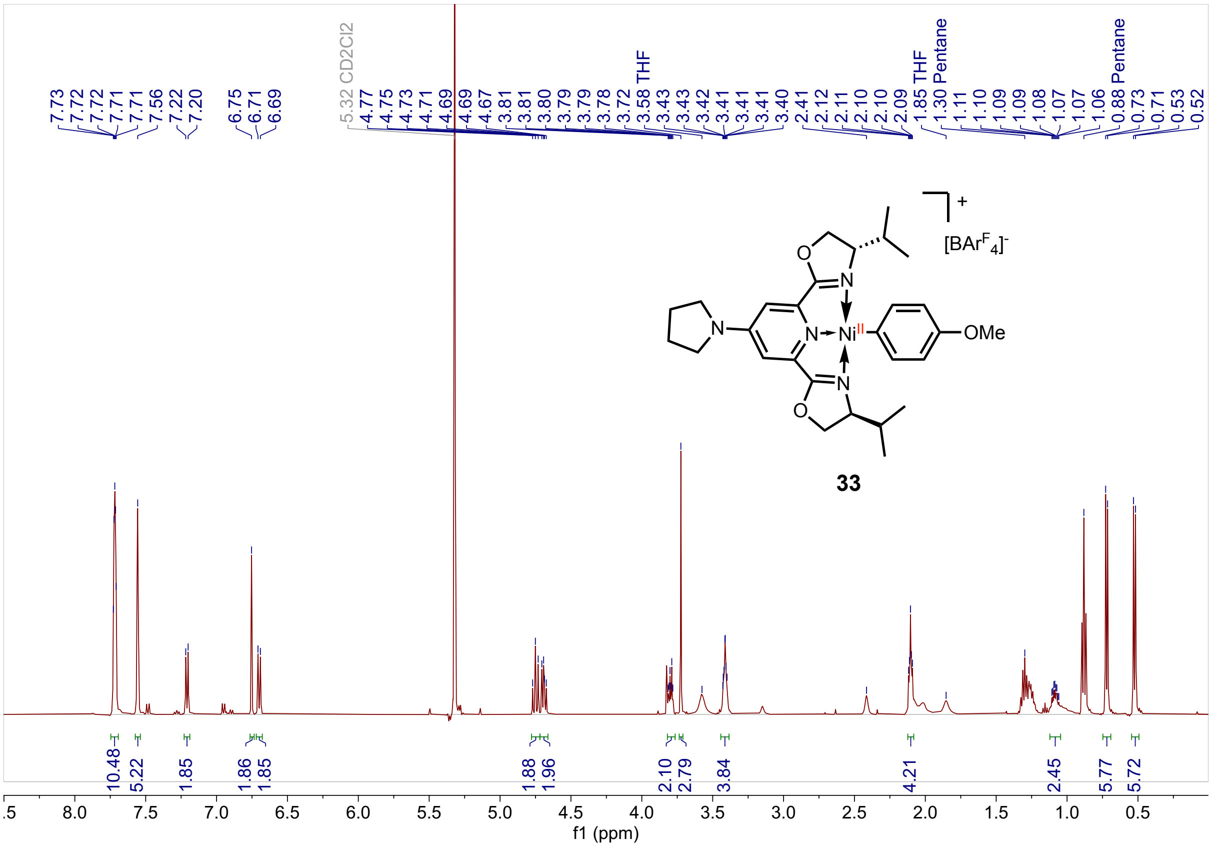

Figure S141. ^{1}H NMR (500 MHz, CD_2Cl_2, 25 °C) of [(4-Pyrrolidinyl-^{iPr}pybox)Ni(*p*-MeO-C_6H_4)]BAr^F_4 **33.**

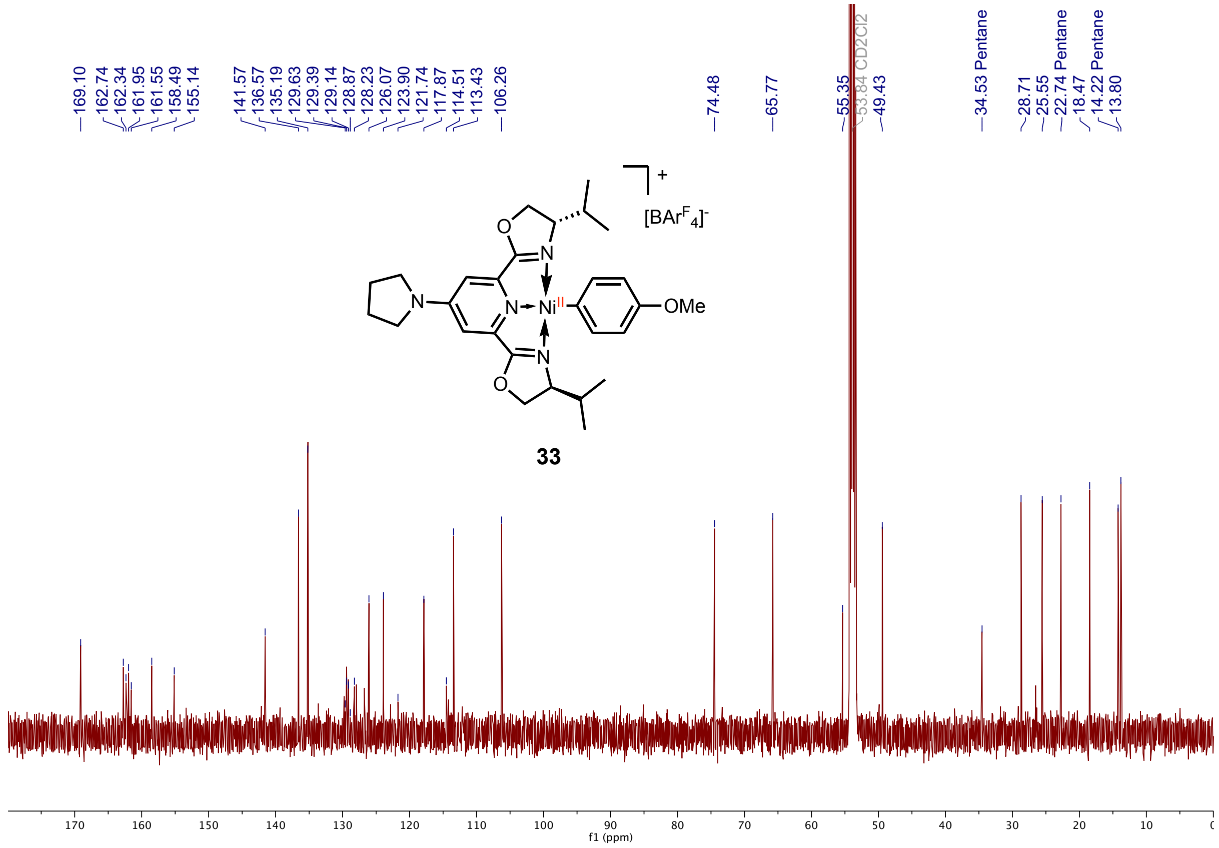

Figure S142. ^{13}C NMR (126 MHz, CD_2Cl_2, 25 °C) of [(4-Pyrrolidinyl-^{iPr}pybox)Ni(*p*-MeO-C_6H_4)]BAr^F$_4$ **33.**

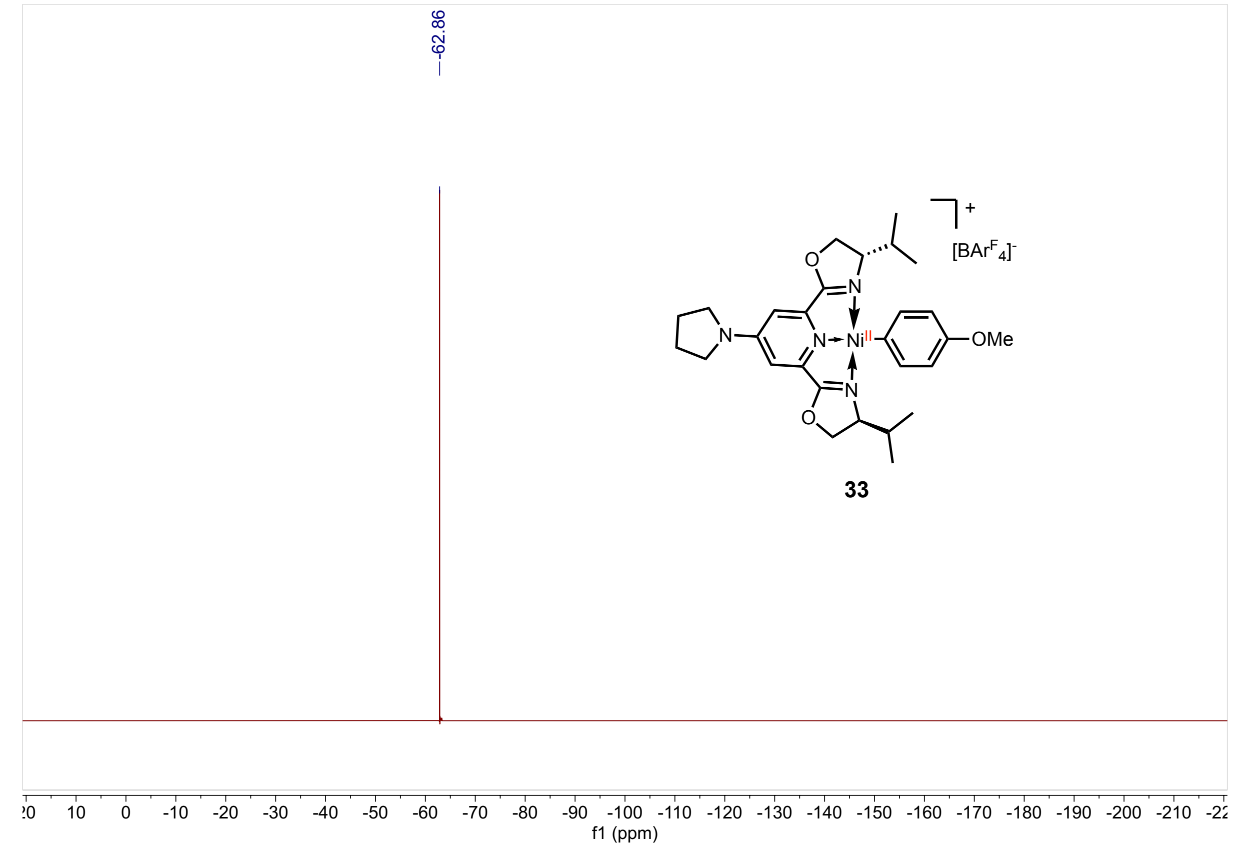

Figure S143. ^{19}F NMR (471 MHz, CD_2Cl_2, 25 °C) of [(4-Pyrrolidinyl-^{iPr}pybox)Ni(p-MeO-C_6H_4)]BAr^F_4 **33.**

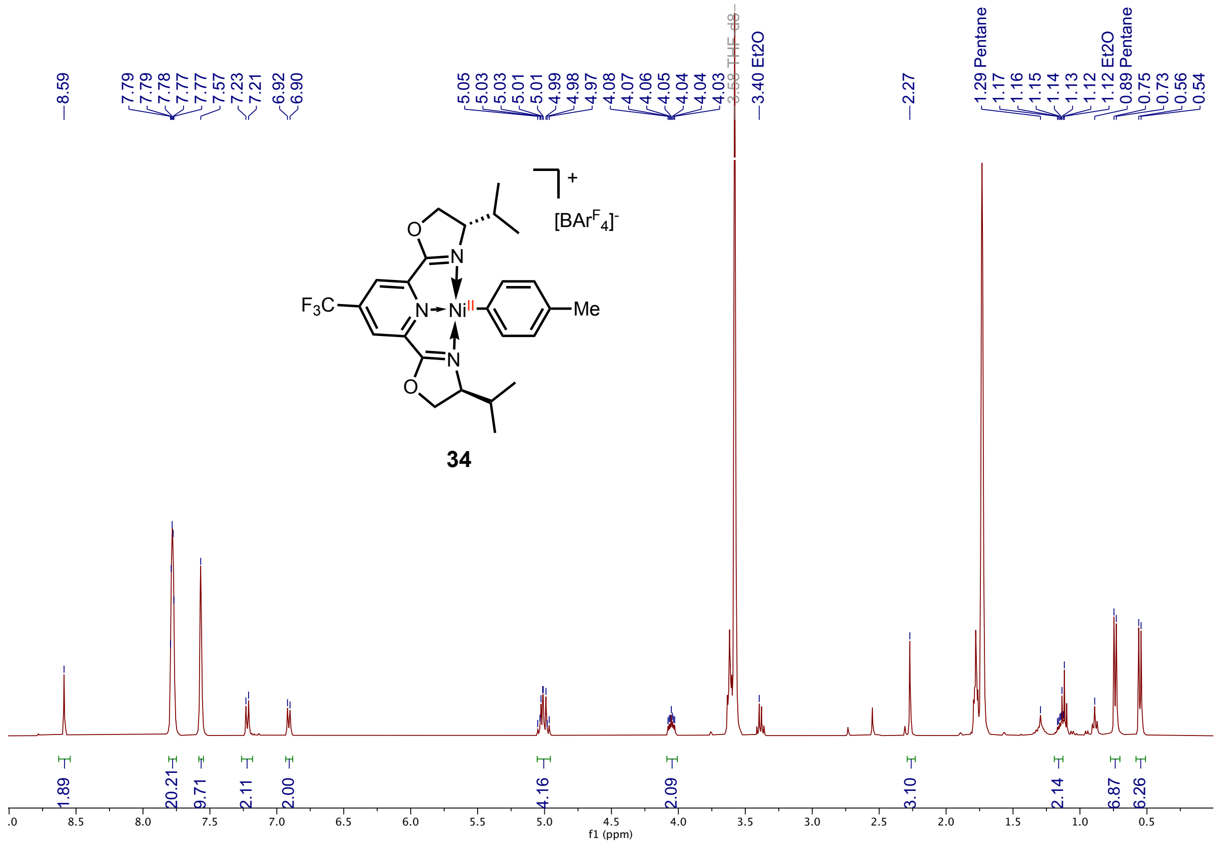

Figure S144. ^{1}H NMR (400 MHz, THF-*d*, 25 °C) of [(4-CF$_3$-$^{i\text{Pr}}$pybox)Ni(*p*-tol)]BAr^F$_4$ **34.**

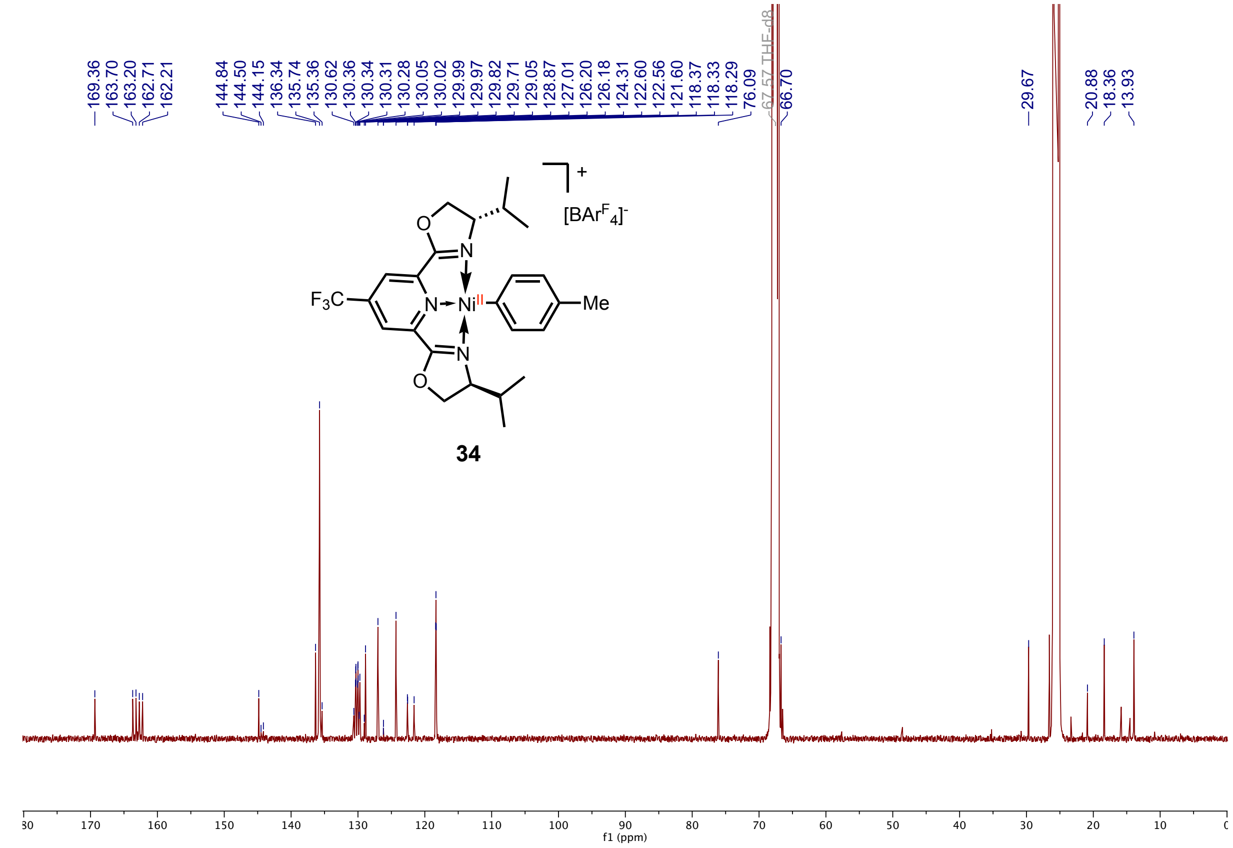

Figure S145. ^{13}C NMR (126 MHz, THF-*d*, 25 °C) of [(4-CF$_3$-^{iPr}pybox)Ni(*p*-tol)]BAr^F$_4$ **34.**

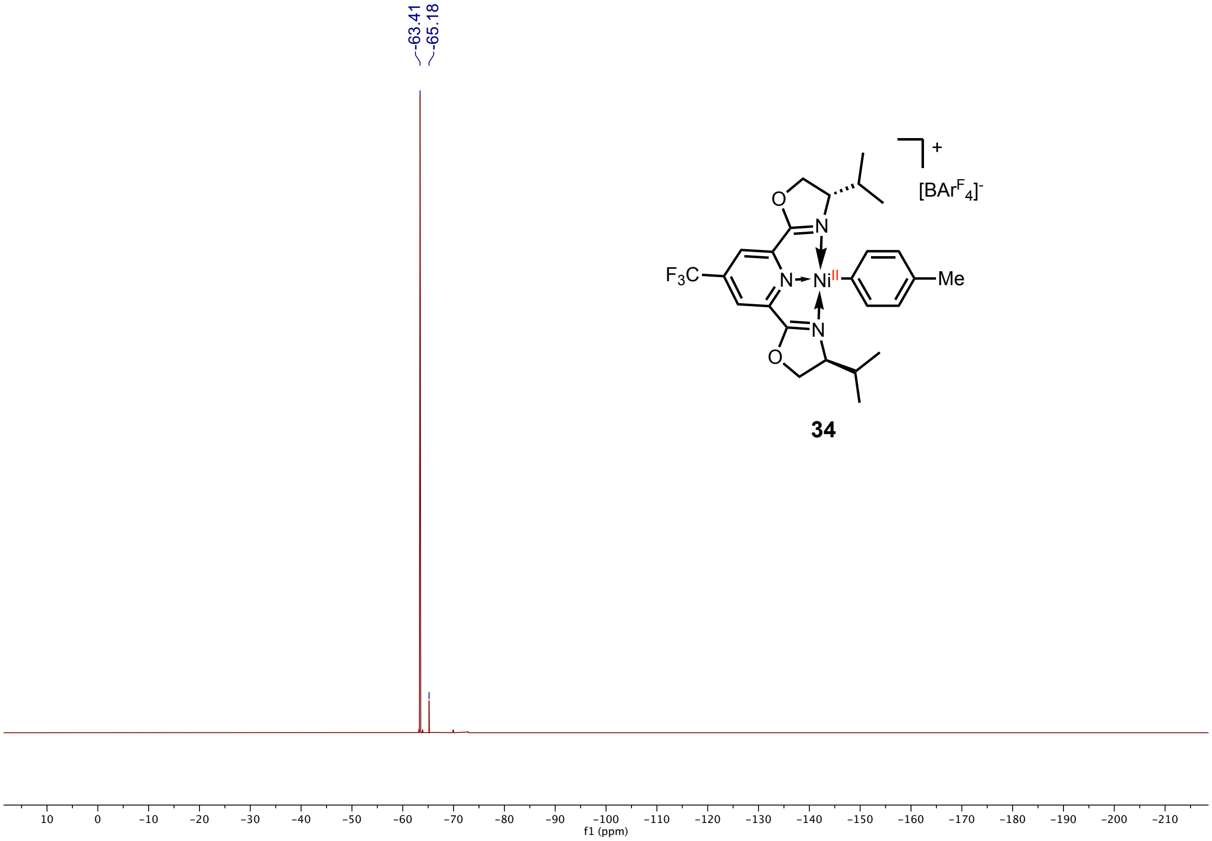

Figure S146. ^{19}F NMR (377 MHz, THF-*d*, 25 °C) of [(4-CF_3-$^{i\text{Pr}}$pybox)Ni(*p*-tol)]BAr^F_4 **34.**

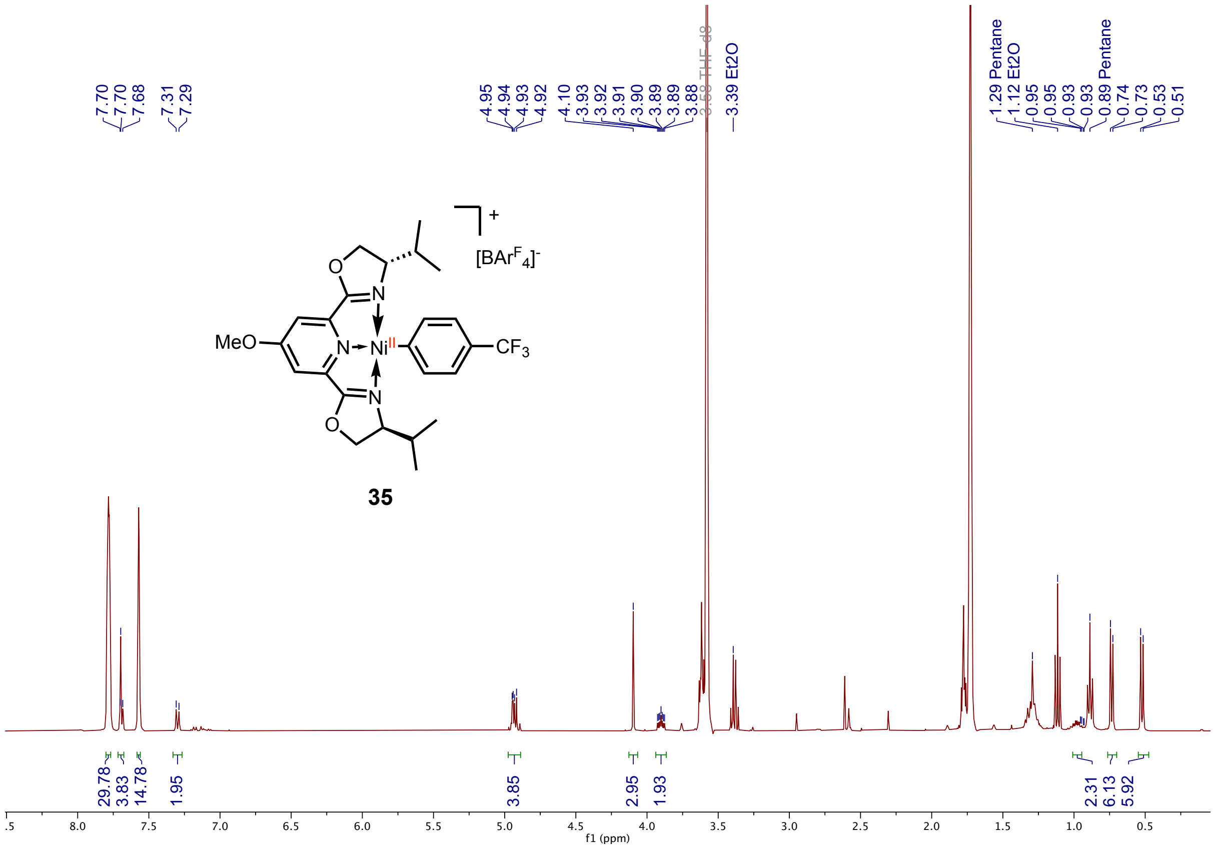

Figure S147. ^{1}H NMR (400 MHz, THF-*d*, 25 °C) of [(4-OMe-^{iPr}pybox)Ni(*p*-CF$_3$-C$_6$H$_4$)]BAr$^F_4$ **35.**

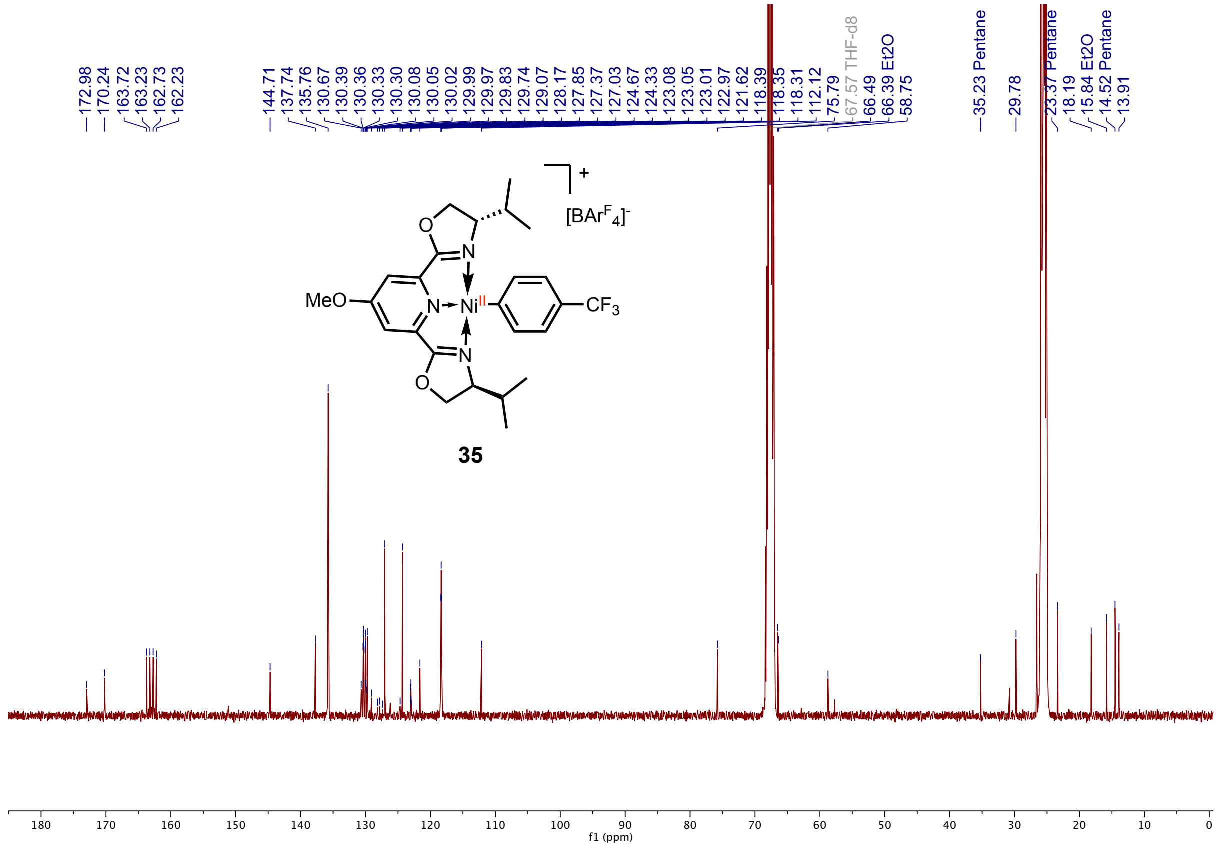

Figure S148. ^{13}C NMR (126 MHz, THF-*d*, 25 °C) of [(4-OMe-^{iPr}pybox)Ni(*p*-CF_3-C_6H_4)]BAr^F_4 **35.**

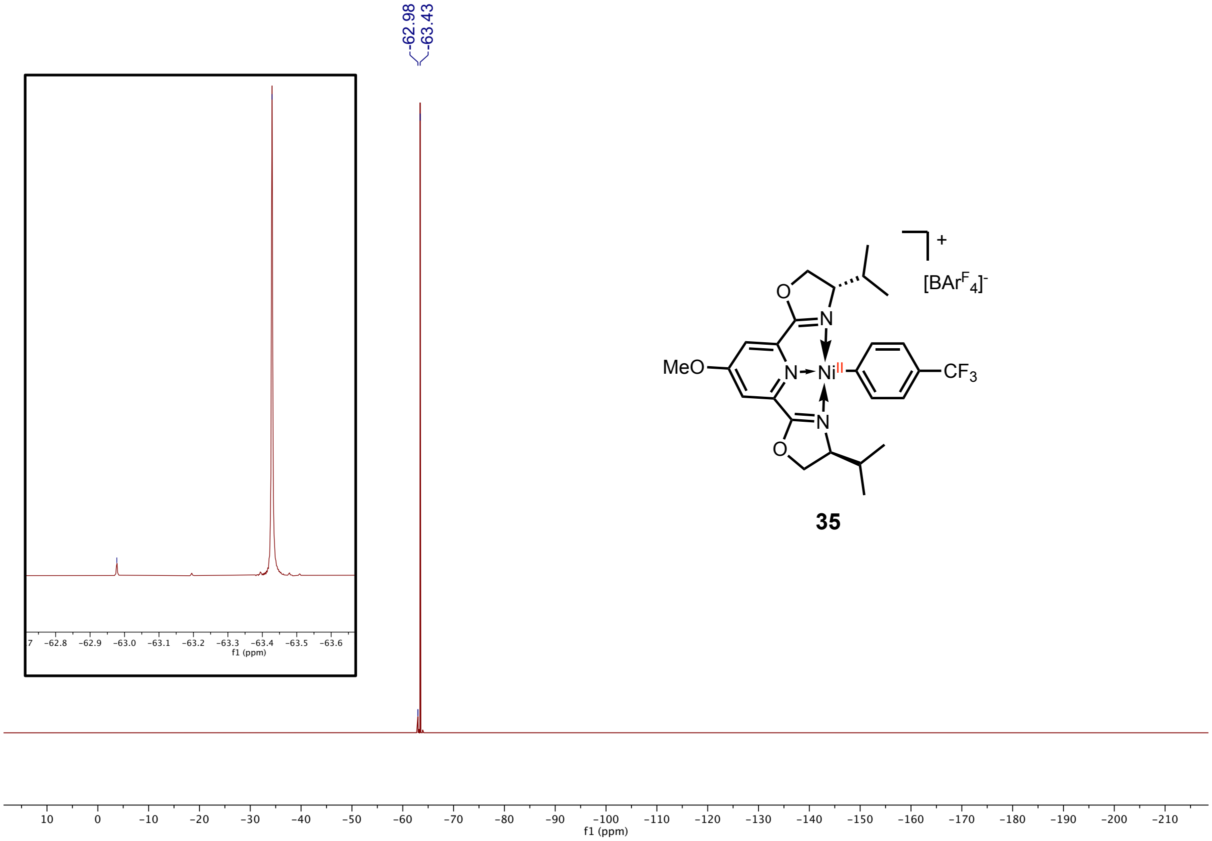

Figure S149. ^{19}F NMR (377 MHz, THF-*d*, 25 °C) of [(4-OMe-$^{i\text{Pr}}$pybox)Ni(*p*-CF_3-C_6H_4)]BAr^F_4 **35.**

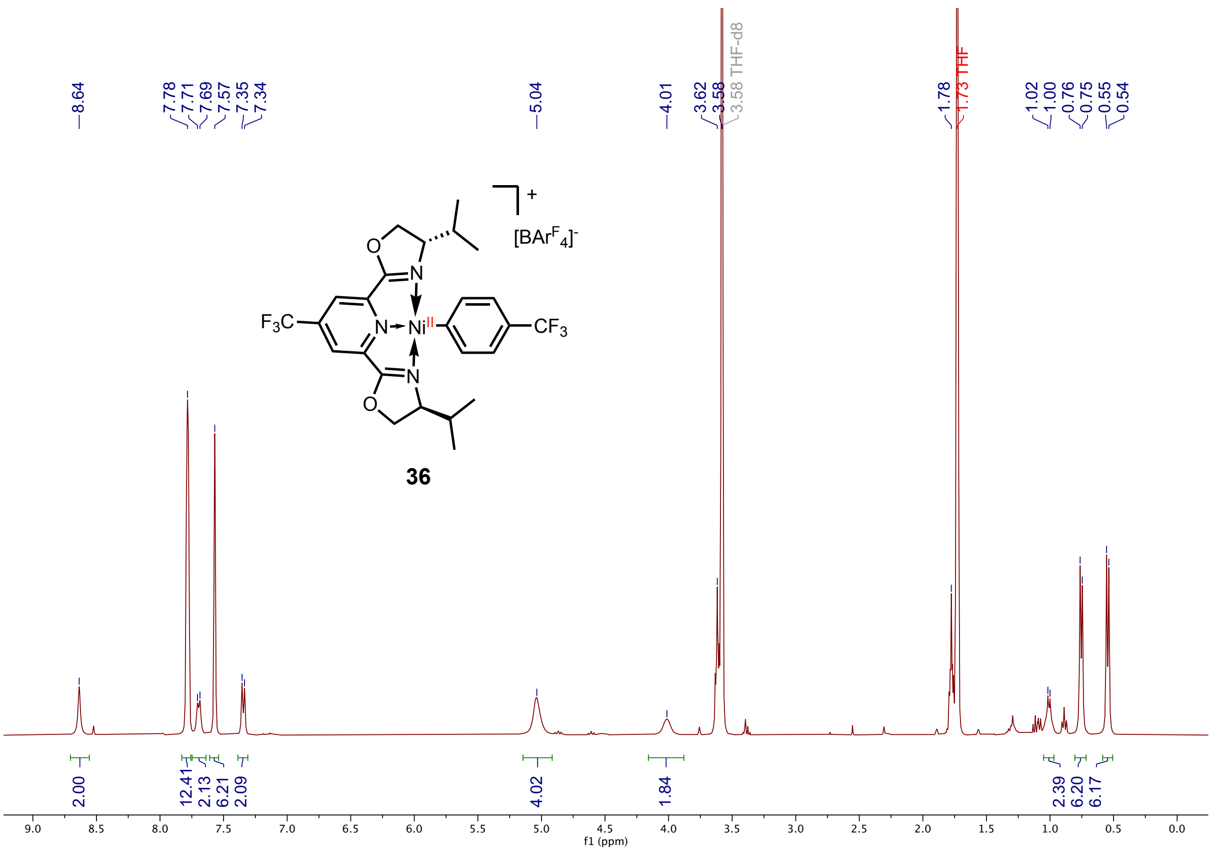

Figure S150. ^1H NMR (400 MHz, THF-*d*, 25 °C) of [(4-CF_3-^{iPr}pybox)Ni(*p*-CF_3-C_6H_4)]BAr$^F_4$ **36.**

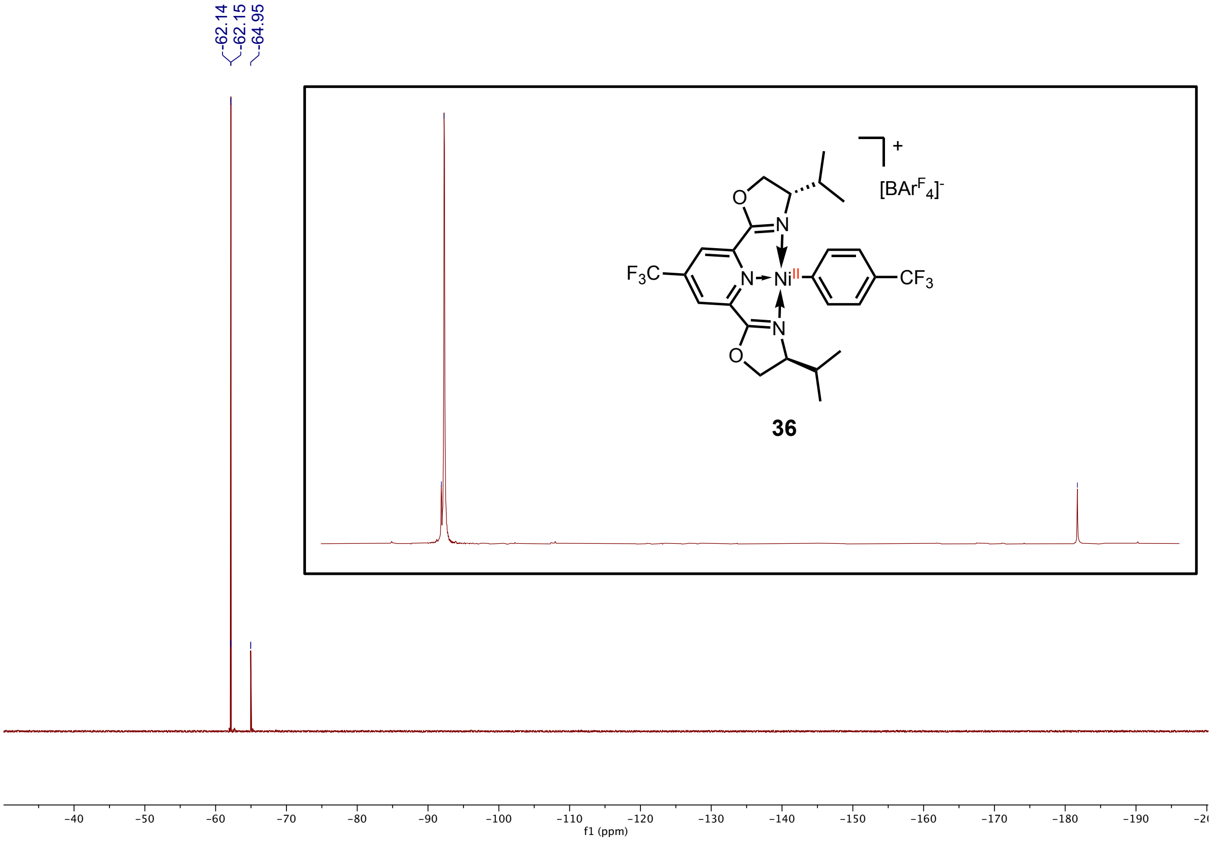

Figure S151. ^{19}F NMR (377 MHz, benzene-*d*, 25 °C) of [(4-CF_3-^{iPr}pybox)Ni(*p*-CF_3-C_6H_4)]BAr$^F{}_4$ **36.**

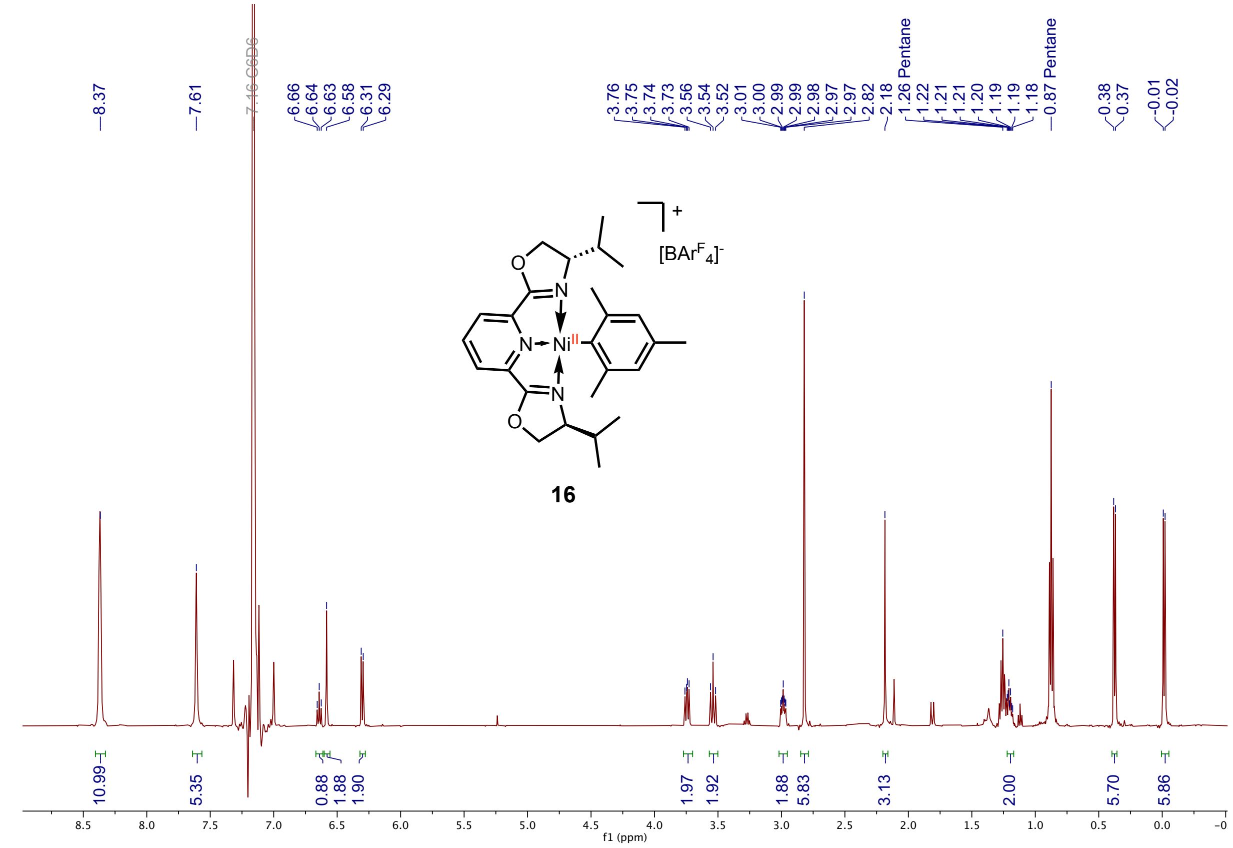

Figure S152. ^{1}H NMR (500 MHz, benzene-*d*, 25 °C) of [(^{iPr}pybox)Ni(Mes)]BAr$^{F}_{4}$ **16.**

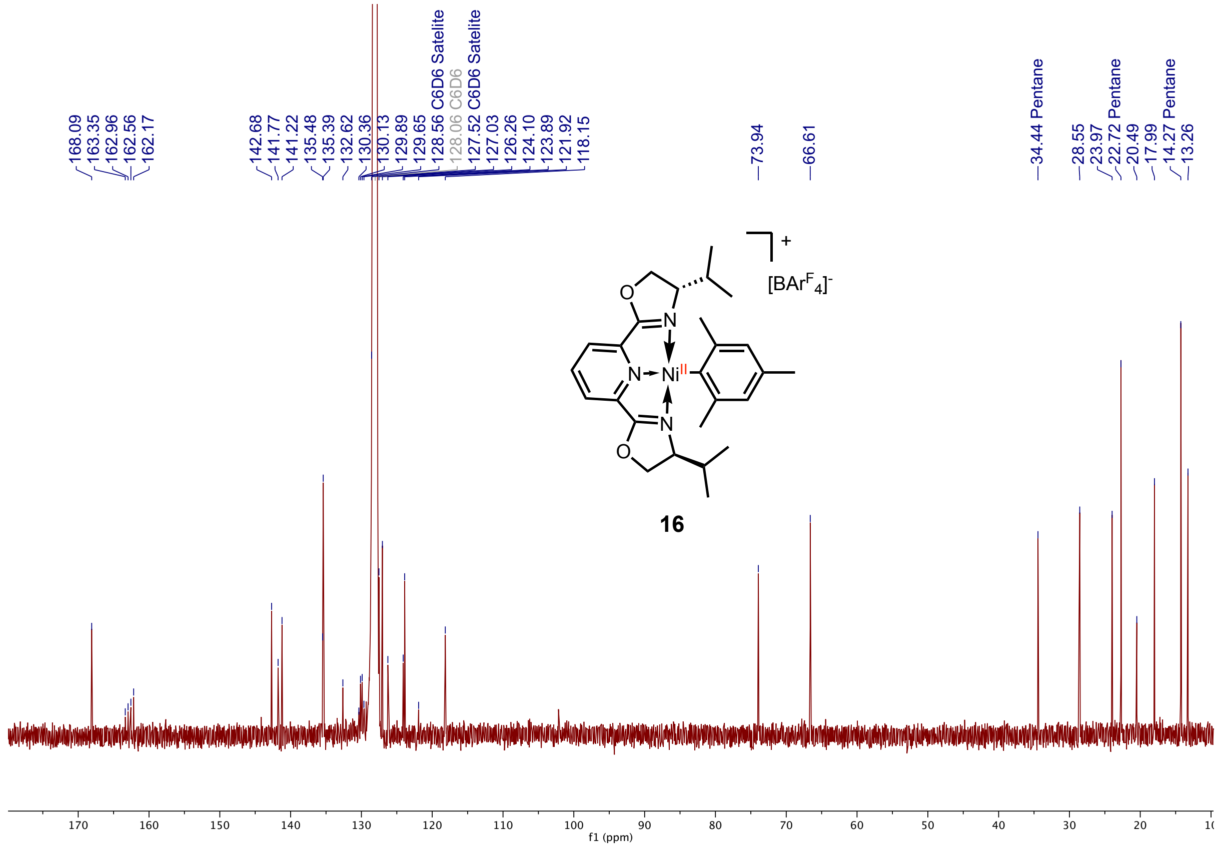

Figure S153. ^{13}C NMR (126 MHz, benzene-*d*, 25 °C) of [(^{iPr}pybox)Ni(Mes)]BAr^F$_4$ **16.**

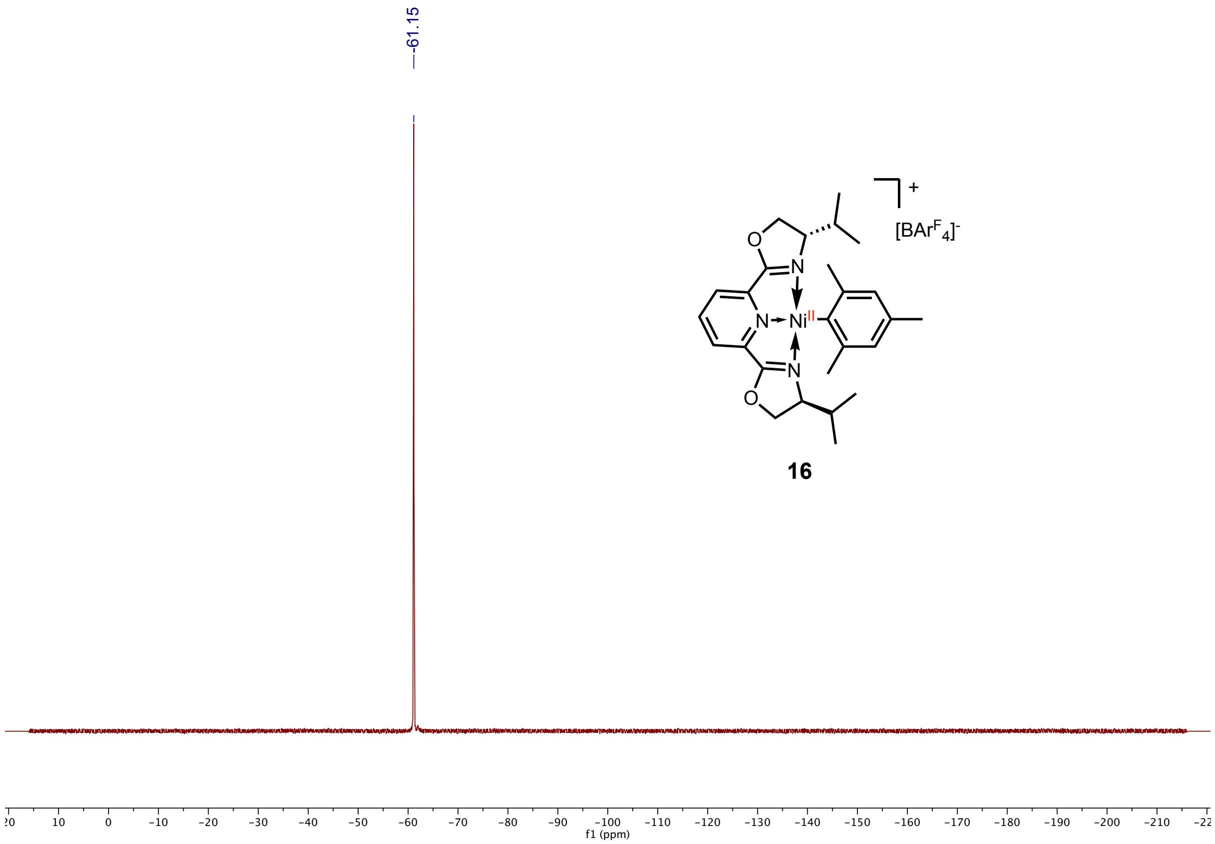

Figure S154. ^{19}F NMR (471 MHz, benzene-*d*, 25 °C) of [($^{i\text{Pr}}$pybox)Ni(Mes)]BAr$^{F}_{4}$ **16.**

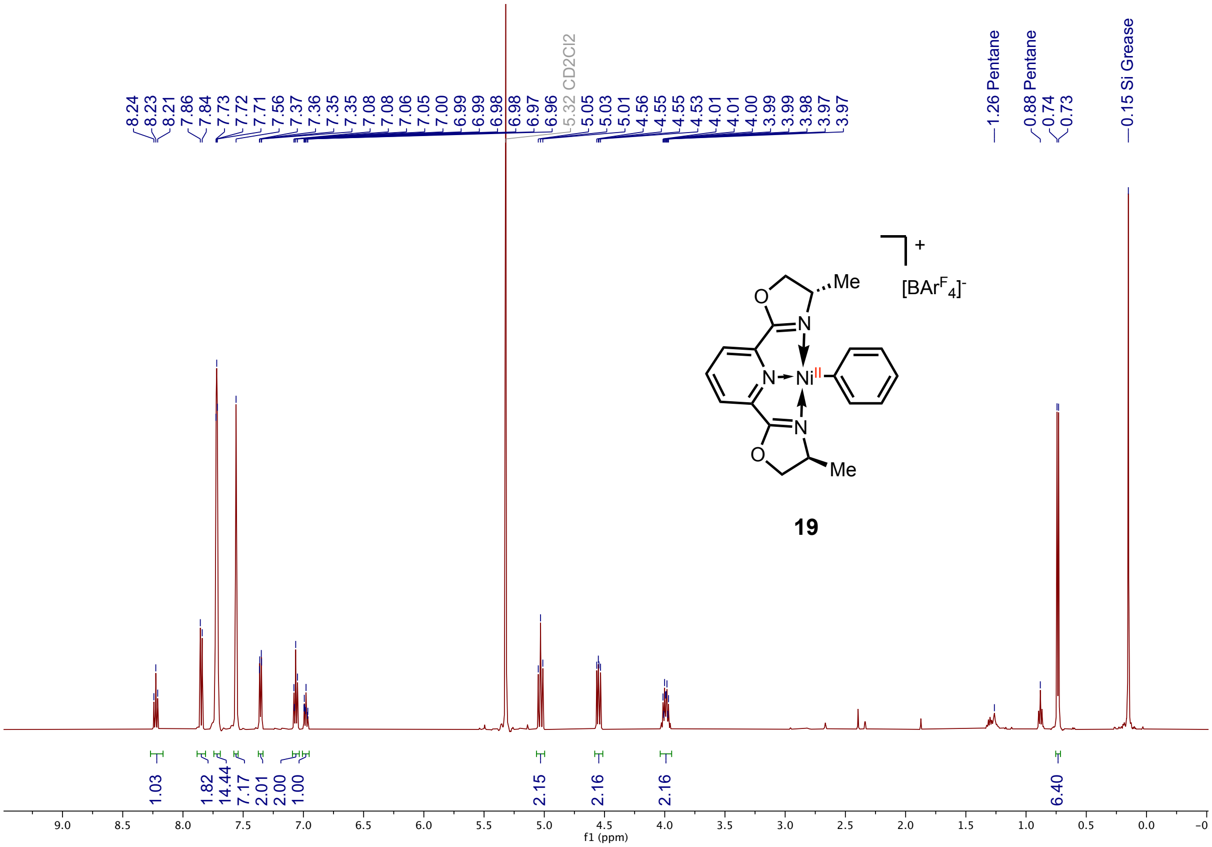

Figure S155. ^{1}H NMR (500 MHz, CD_2Cl_2, 25 °C) of [(^{Me}pybox)Ni(Ph)]BAr^F_4 **19.**

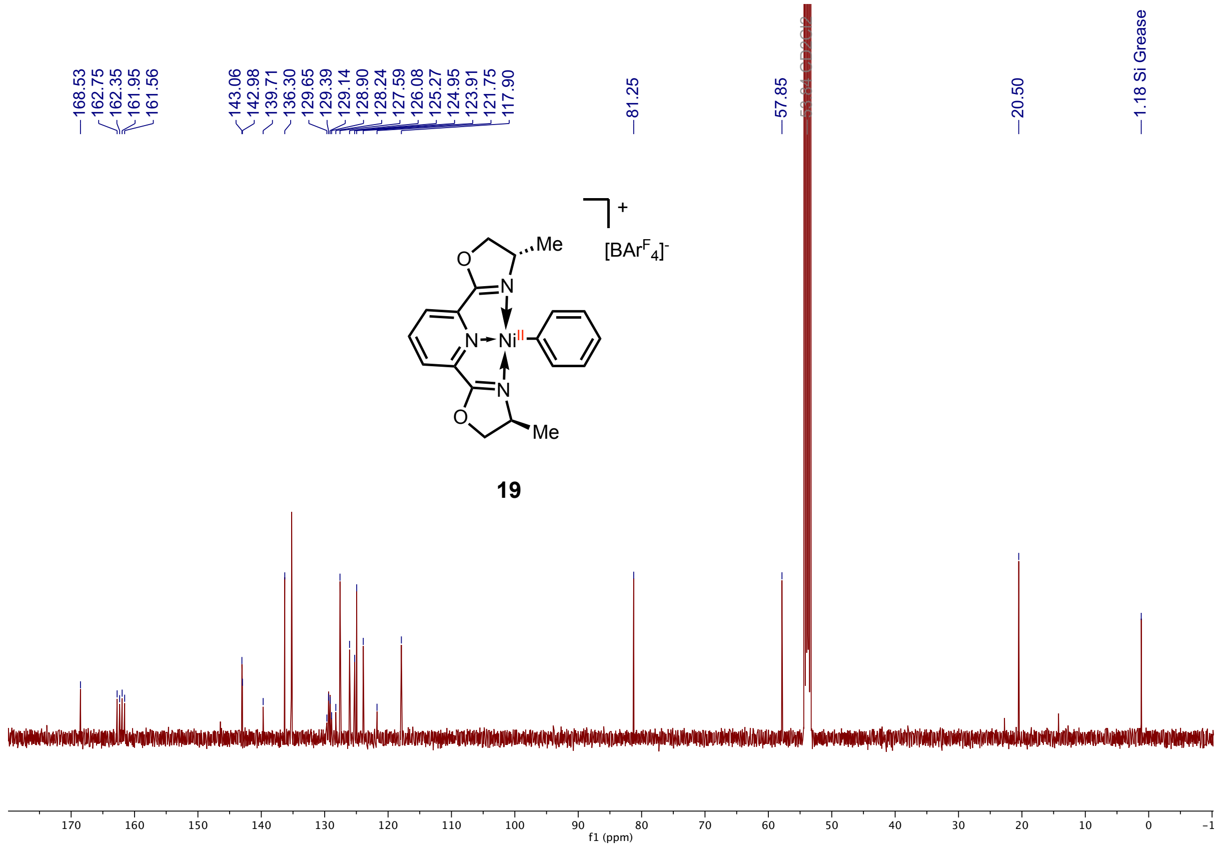

Figure S156. ^{13}C NMR (126 MHz, CD_2Cl_2, 25 °C) of $[(^{Me}pybox)Ni(Ph)]BAr^F_4$ **19.**

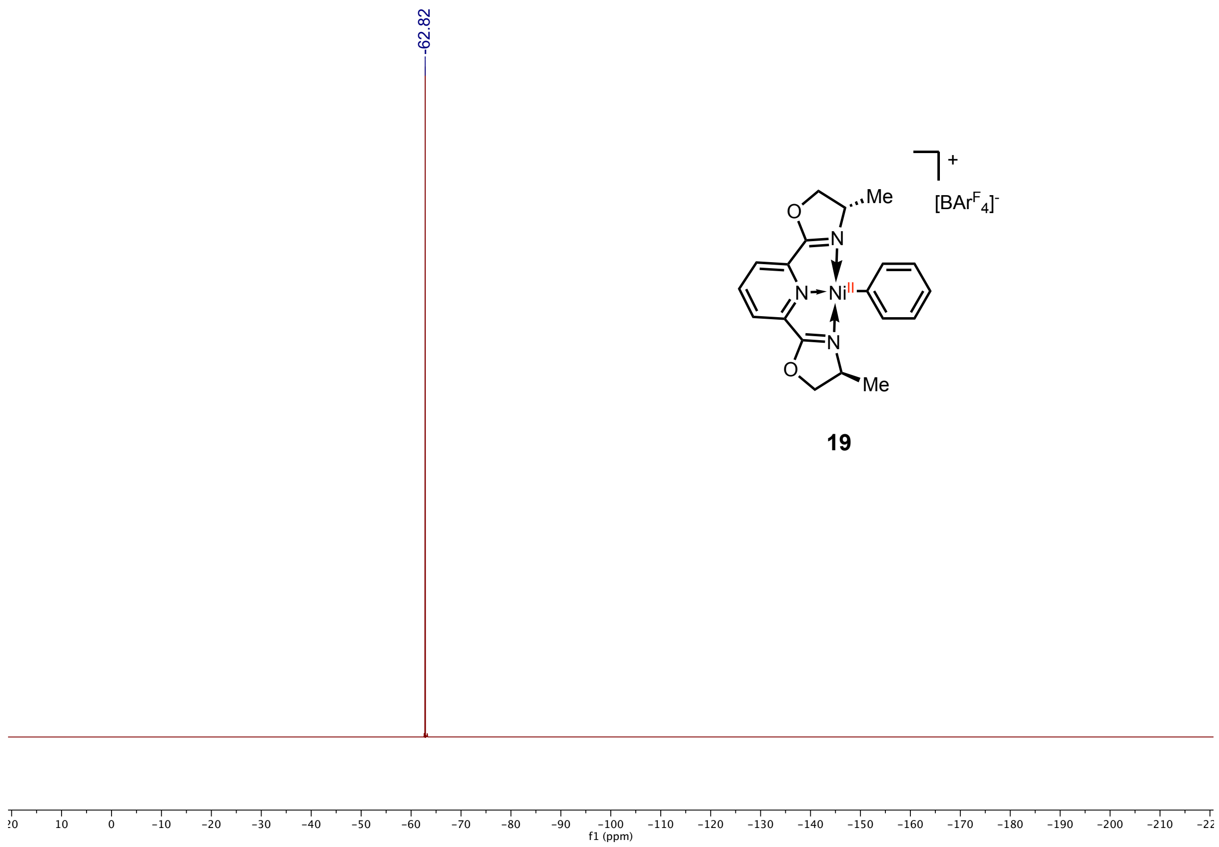

Figure S157. ^{19}F NMR (471 MHz, CD_2Cl_2, 25 °C) of [(^{Me}pybox)Ni(Ph)]BAr^F_4 **19.**

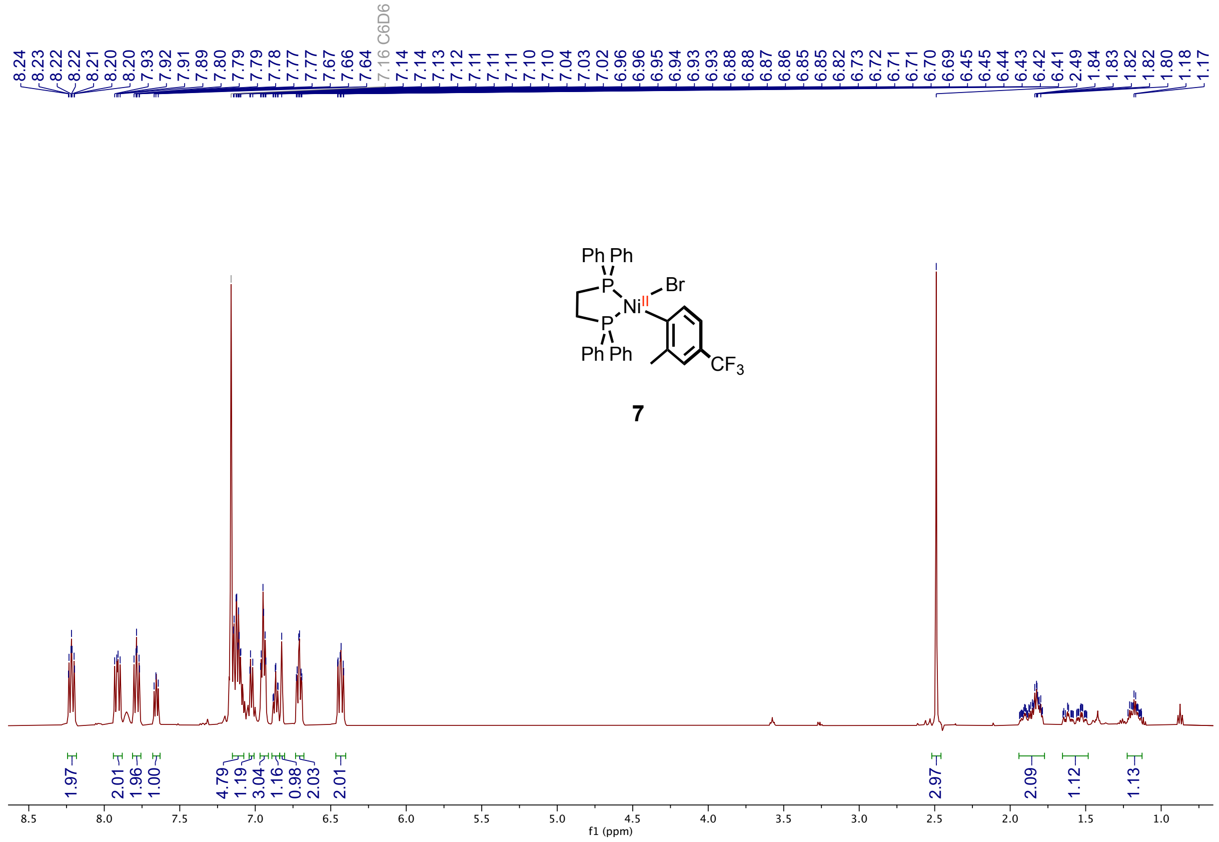

Figure S158. ^{1}H NMR (500 MHz, benzene-*d*, 25 °C) of (dppe)Ni(*p*-CF_3-*o*-tol)Br **7.**

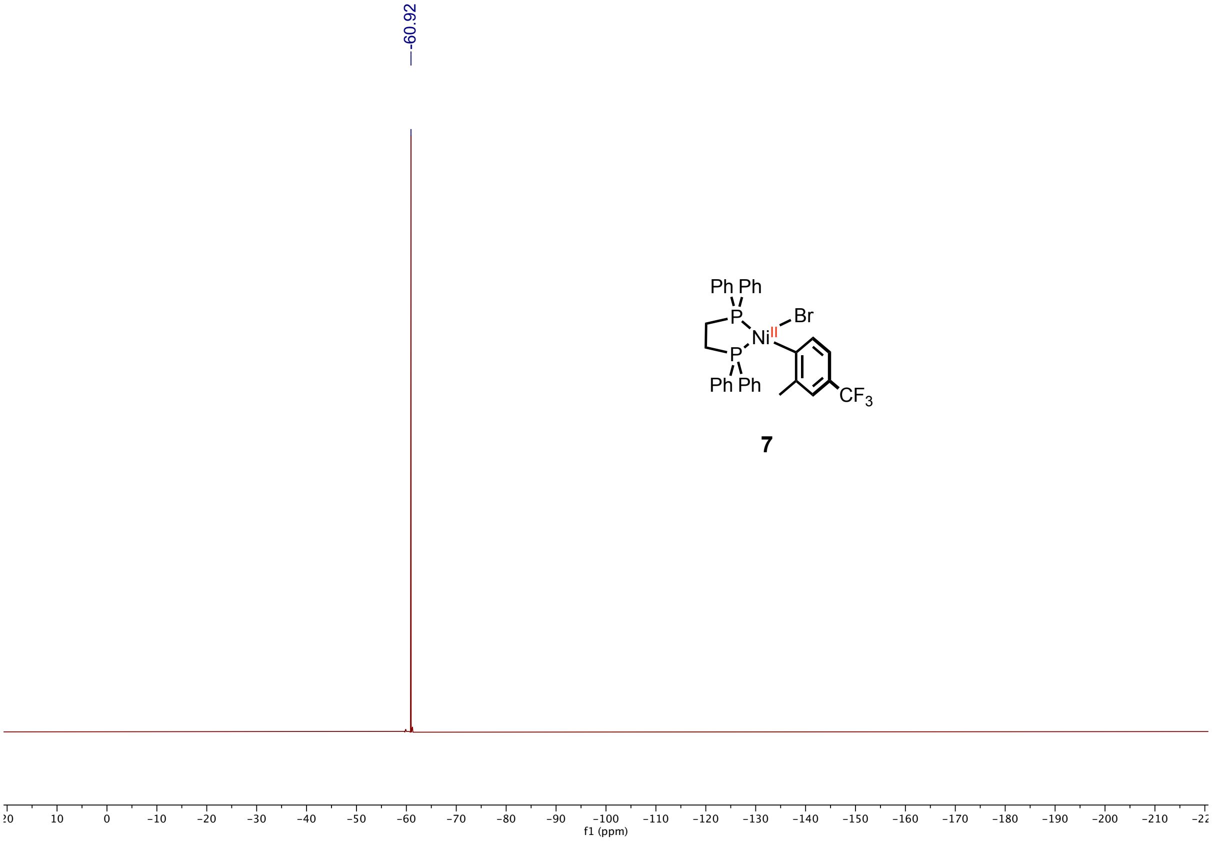

Figure S159. ^{19}F NMR (471 MHz, benzene-*d*, 25 °C) of (dppe)Ni(*p*-CF_3-*o*-tol)Br **7.**

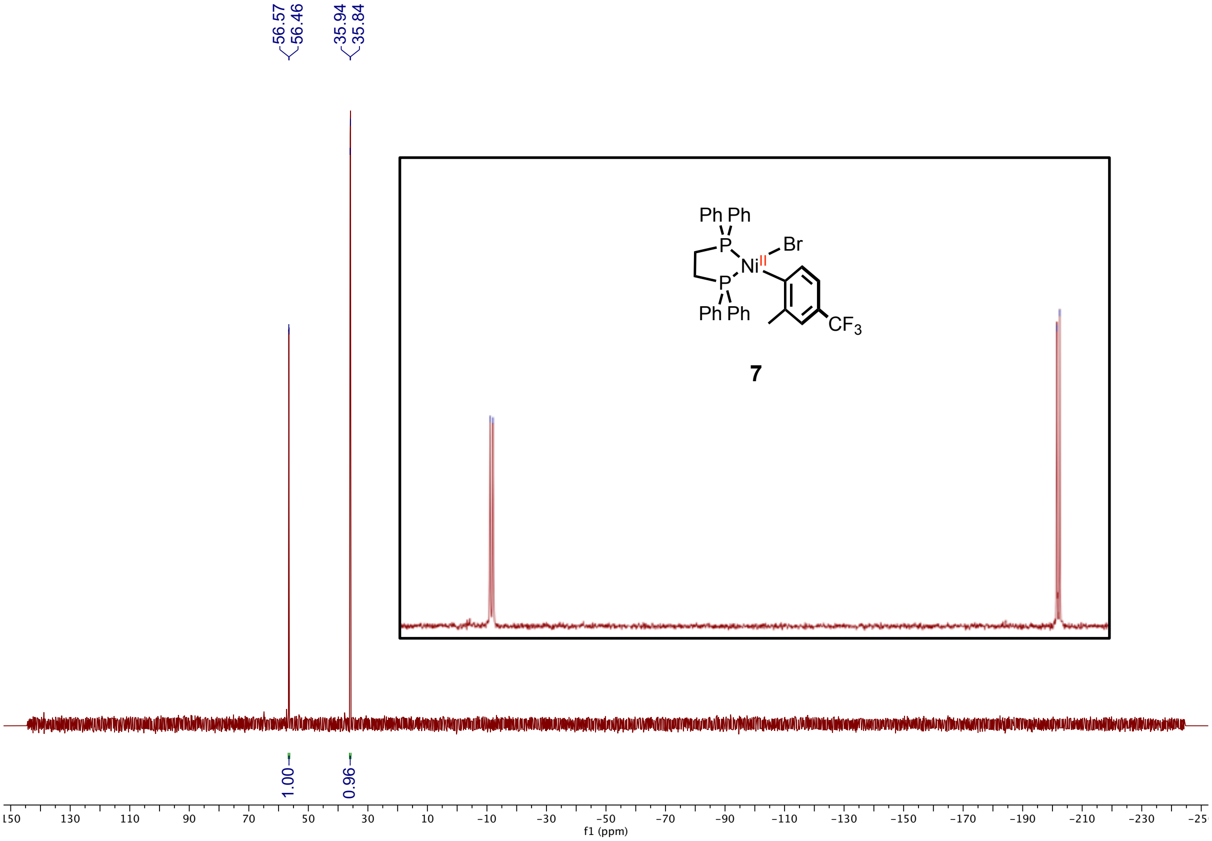

Figure S160. ^{31}P NMR (202 MHz, benzene-*d*, 25 °C) of (dppe)Ni(*p*-CF_3-*o*-tol)Br **7.**

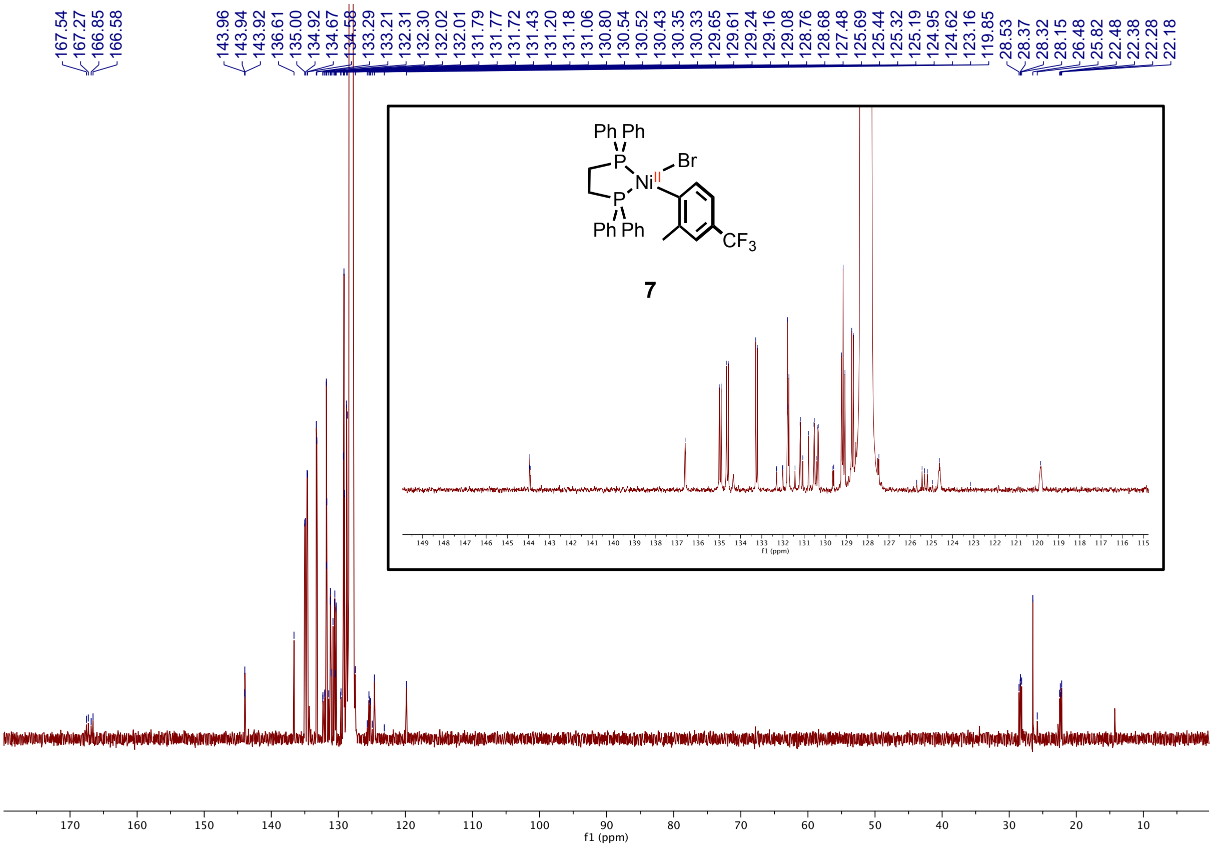

Figure S161. ^{13}C NMR (126 MHz, benzene-*d*, 25 °C) of (dppe)Ni(*p*-CF_3-*o*-tol)Br **7.**

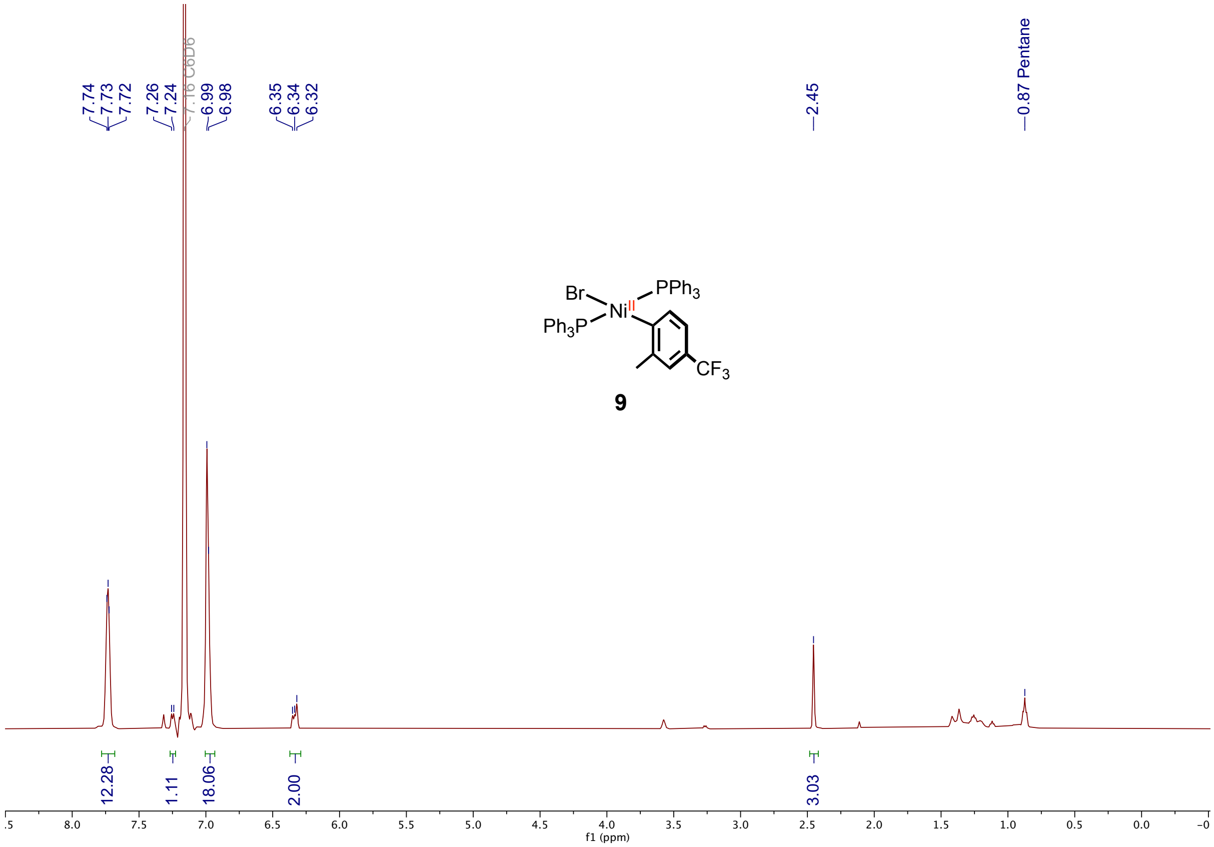

Figure S162. ^{1}H NMR (500 MHz, benzene-*d*, 25 °C) of $(PPh_3)_2Ni$(*p*-CF_3-*o*-tol)Br **9.**

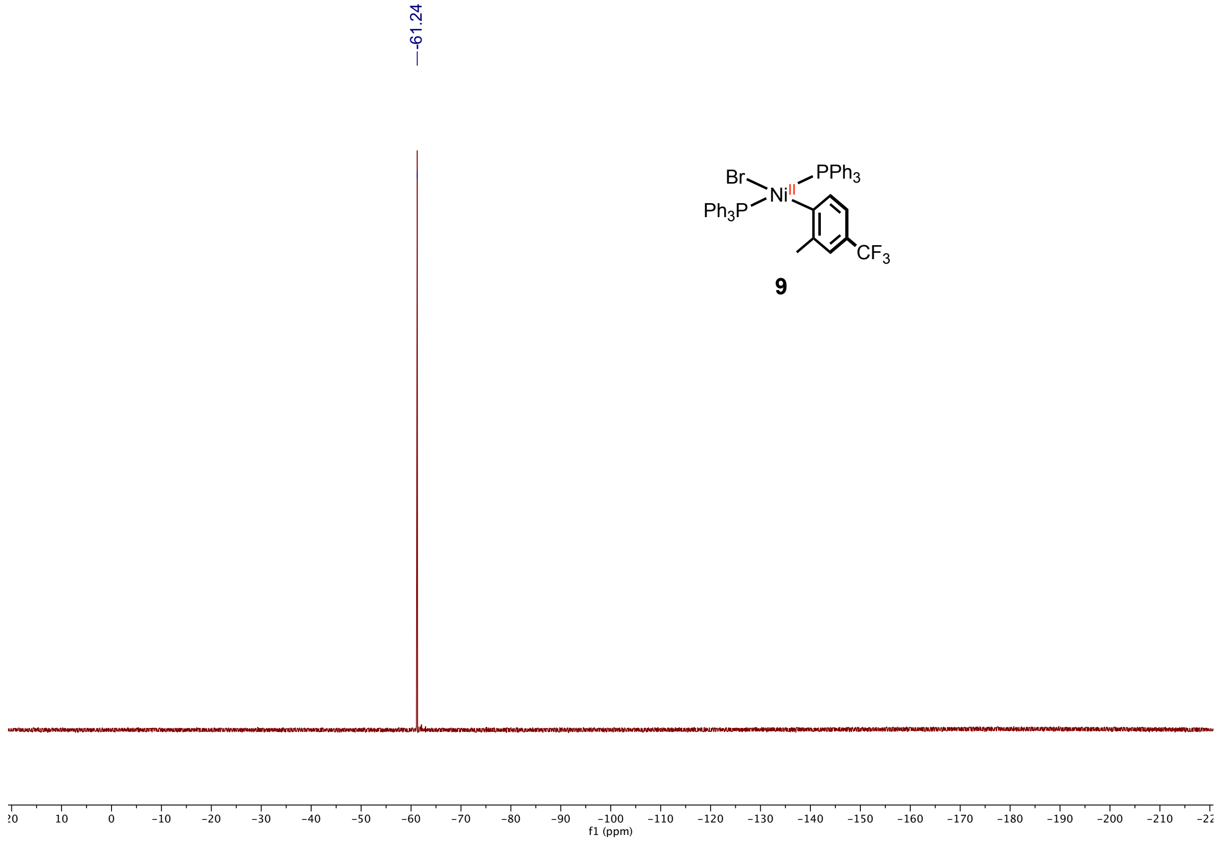

Figure S163. ^{19}F NMR (471 MHz, benzene-*d*, 25 °C) of $(PPh_3)_2Ni$(*p*-CF_3-*o*-tol)Br **9.**

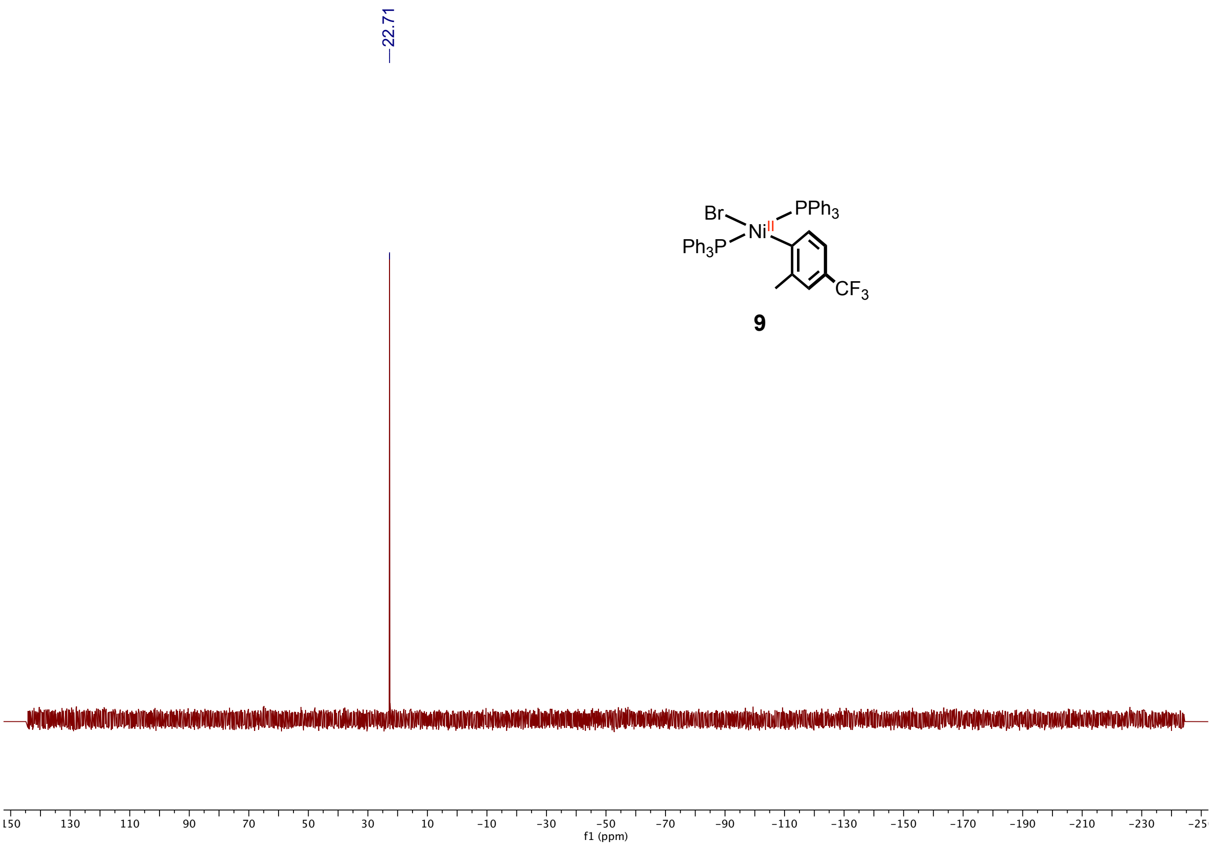

Figure S164. ^{31}P NMR (202 MHz, benzene-*d*, 25 °C) of $(PPh_3)_2Ni$(*p*-CF_3-*o*-tol)Br **9.**

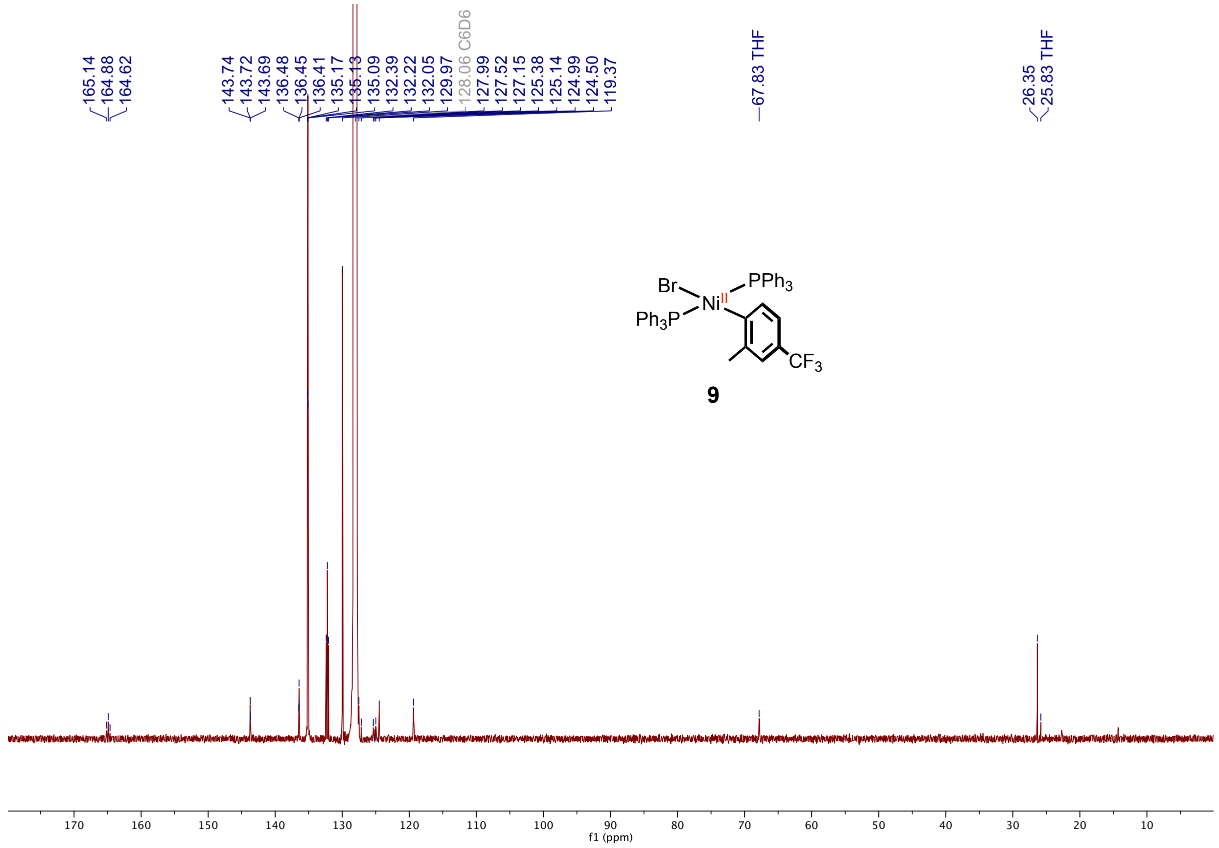

Figure S165. ^{13}C NMR (126 MHz, benzene-*d*, 25 °C) of $(PPh_3)_2Ni$(*p*-CF_3-*o*-tol)Br **9.**

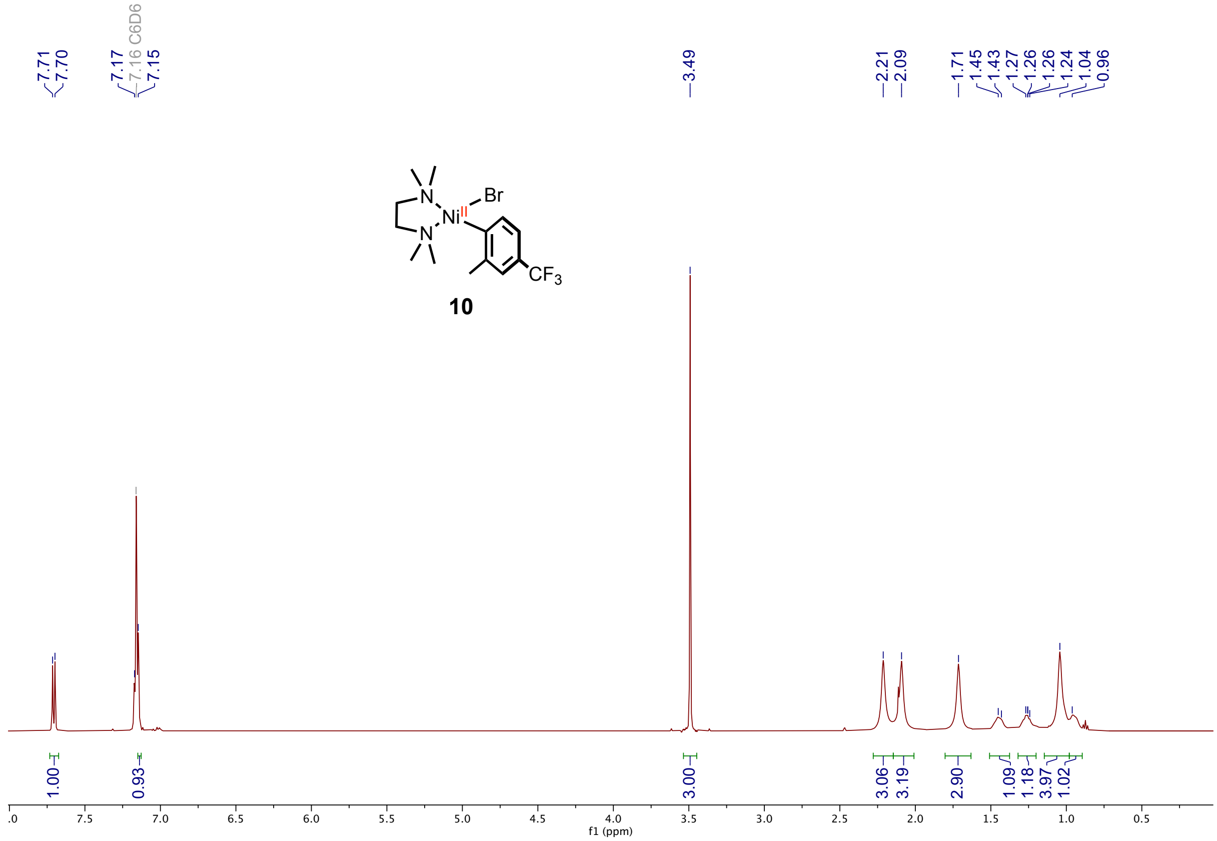

Figure S166. ^{1}H NMR (500 MHz, benzene-*d*, 25 °C) of (TMEDA)Ni(*p*-CF_3-*o*-tol)Br **10.**

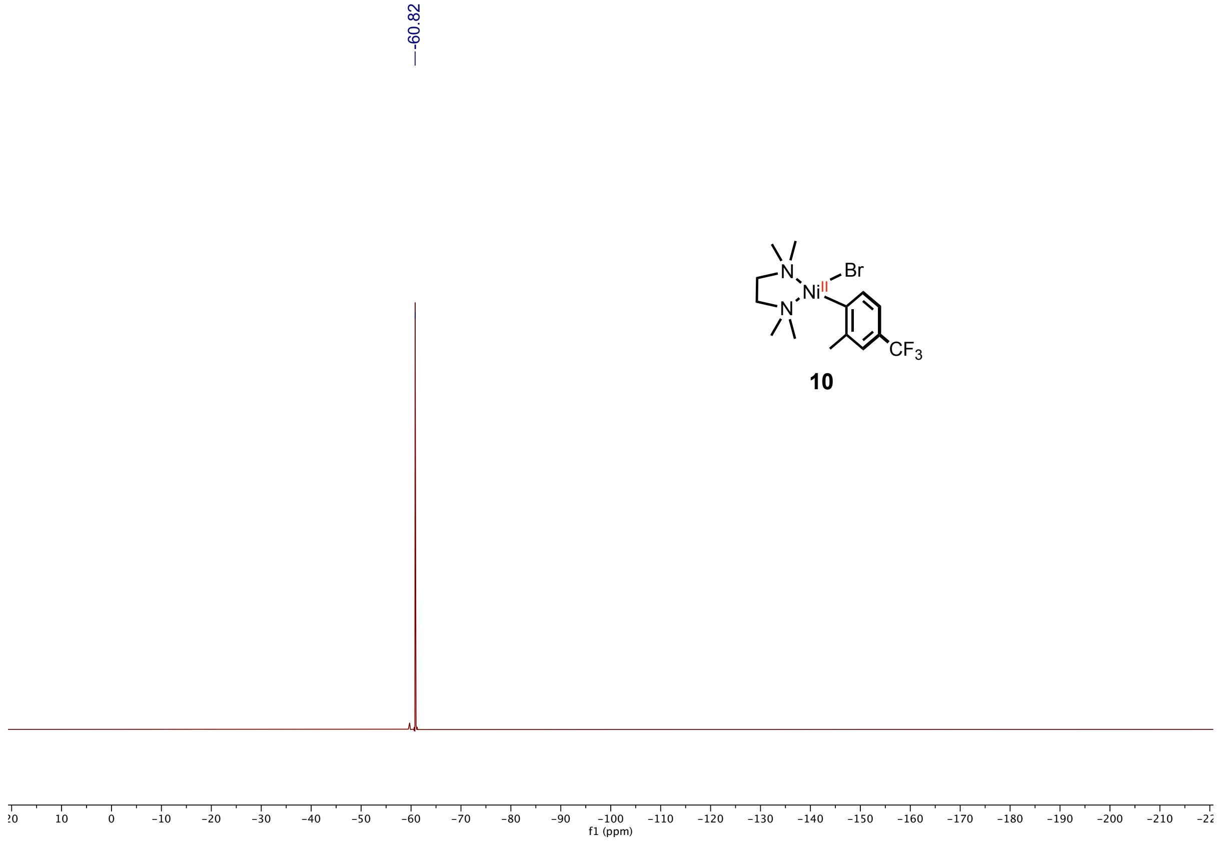

Figure S167. ^{19}F NMR (471 MHz, benzene-*d*, 25 °C) of (TMEDA)Ni(*p*-CF_3-*o*-tol)Br **10.**

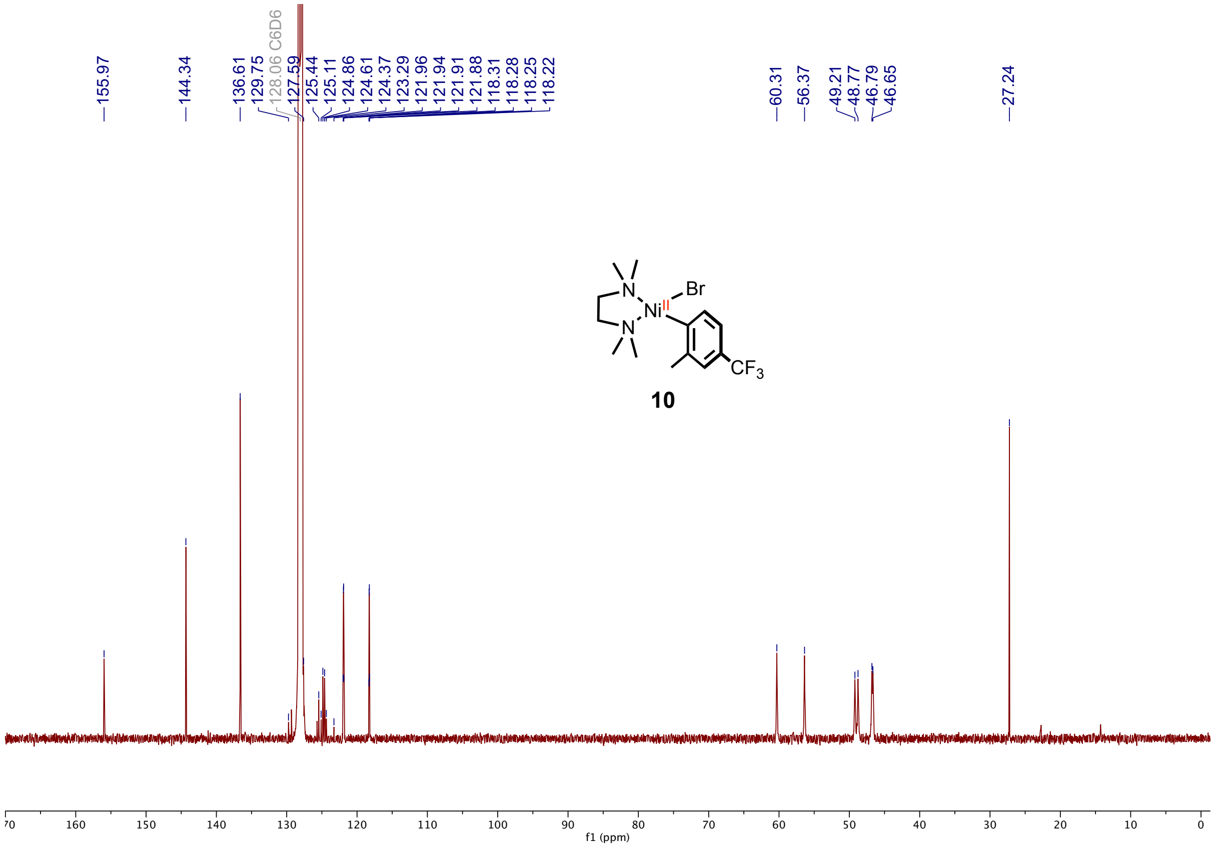

Figure S168. ^{13}C NMR (126 MHz, benzene-*d*, 25 °C) of (TMEDA)Ni(*p*-CF_3-*o*-tol)Br **10.**

24. References

[1] Lin, Q.; Spielvogel, E. H.; Diao, T. Carbon-centered radical capture at nickel(II) complexes: Spectroscopic evidence, rates, and selectivity. *Chem* **2023**, *9*, 1295-1308.

[2] Newcomb, M. Competition methods and scales for alkyl radical reaction kinetics. *Tetrahedron* **1993,** *49*, 1151–1176.

[3] Farrance, I.; Frenkel, R. Uncertainty of Measurement: A Review of the Rules for Calculating Uncertainty Components through Functional Relationships *Clin. Biochem. Rev.* **2012**, *33*, 49–75.

[4] Yan, Y.; Sun, J.; Li, G.; Yang, L.; Zhang, W.; Cao, R.; Wang, C.; Xiao, J.; Xue, D. Photochemically Enabled, Ni-Catalyzed Cyanation of Aryl Halides. *Org. Lett.* **2022**, *24*, 2271-2275.

[5] Speckmeier, E.; Fischer, T. G.; Zeitler, K. A Toolbox Approach To Construct Broadly Applicable Metal-Free Catalysts for Photoredox Chemistry: Deliberate Tuning of Redox Potentials and Importance of Halogens in Donor–Acceptor Cyanoarenes. *J. Am. Chem. Soc.* **2018**, *140*, 15353-15365.

[6] Leifert, D.; Studer, A. The Persistent Radical Effect in Organic Synthesis. *Angew. Chem. Int. Edit.* **2019**, *59*, 74-108.

[7] Nishiyama, H.; Yamaguchi, S.; Kondo, M.; Itoh, K. Electronic substituent effect of nitrogen ligands in catalytic asymmetric hydrosilylation of ketones: chiral 4-substituted bis(oxazolinyl)pyridines. *J. Org. Chem.* **1992**, *57*, 4306-4309.

[8] Poh, J. S.; Makai, S.; von Keutz, T.; Tran, D. N.; Battilocchio, C.; Pasau, P.; Ley, S. V. Rapid Asymmetric Synthesis of Disubstituted Allenes by Coupling of Flow-Generated Diazo Compounds and Propargylated Amines. *Angew. Chem. Int. Ed.* **2017**, *56*, 1864-1868.

[9] Park, S. B.; Murata, K.; Matsumoto, H.; Nishiyama, H. Remote electronic control in asymmetric

cyclopropanation with chiral Ru-pybox catalysts. *Tetrahedron* **1995**, *6*, 2487-2494.
[10] Gutiérrez-Bonet, Á.; Tellis, J. C.; Matsui, J. K.; Vara, B. A.; Molander, G. A. 1,4-Dihydropyridines as Alkyl Radical Precursors: Introducing the Aldehyde Feedstock to Nickel/Photoredox Dual Catalysis. *ACS Catal.* **2016**, *6*, 8004-8008.
[11] Dunsford, J. J.; Clark, E. R.; Ingleson, M. J. Direct C(sp^2)—C(sp^3) Cross-Coupling of Diaryl Zinc Reagents with Benzylic, Primary, Secondary, and Tertiary Alkyl Halides. *Angew. Chem. Int. Ed.* **2015**, *54*, 5688-5692.
[12] Johnson, J.; Dennis, J.; Calyore, C.; Sjoholm, J.; Lutz, J.; Gair, J. Nickel-Catalyzed Direct Addition of Diorganozinc Reagents to Phthalimides: Selective Formation of Gamma-Hydroxylactams. *Synlett* **2013**, *24*, 2567-2570.
[13] Chisholm, M. H.; Gallucci, J. C.; Yin, H.; Zhen, H. Arylzinc Alkoxides: $[ArZnOCHPr^{i}_2]_2$ and $Ar_2Zn_3(OCHPr^{i}_2)_4$ When Ar = C_6H_5, *p*-$CF_3C_6H_4$, 2,4,6-$Me_3C_6H_2$, and C_6F_5. *Inorg. Chem.* **2005**, *44*, 4777-4785.
[14] Ting, S. I.; Garakyaraghi, S.; Taliaferro, C. M.; Shields, B. J.; Scholes, G. D.; Castellano, F. N.; Doyle, A. G. ^{3}d-d Excited States of Ni(II) Complexes Relevant to Photoredox Catalysis: Spectroscopic Identification and Mechanistic Implications. *J. Am. Chem. Soc.* **2020**, *142*, 5800-5810.
[15] Schley, N. D.; Fu, G. C. Nickel-Catalyzed Negishi Arylations of Propargylic Bromides: A Mechanistic Investigation. *J. Am. Chem. Soc.* **2014**, *136*, 16588-16593.
[16] Reuther, J. F.; Bhatt, M. P.; Tian, G.; Batchelor, B. L.; Campos, R.; Novak, B. M. Controlled Living Polymerization of Carbodiimides Using Versatile, Air-Stable Nickel(II) Initiators: Facile Incorporation of Helical, Rod-like Materials. *Macromolecules* **2014**, *47*, 4587-4595.
[17] Hansch, C.; Leo, A.; Taft, R. W. A survey of Hammett substituent constants and resonance and field parameters. *Chem. Rev.* **1991**, *91*, 165-195.
[18] Jiang, X.; Ji, G. A Self-consistent and Cross-checked Scale of Spin-delocalization Substituent Constants, the $\sigma^{\bullet}_{JJ}$ Scale. *J. Org. Chem.* **1992**, *57*, 6051-6056.
[19] (a) Neese, F. *Wiley Interdiscip. Rev.: Comput. Mol. Sci.* **2011**, *2*, 73–78 (b) Neese, F. *Wiley Interdiscip. Rev.: Comput. Mol. Sci.* **2017**, *8*, e1327.
[20] (a) Becke, A. D. Density-Functional Thermochemistry. III. The Role of Exact Exchange. *J. Chem. Phys.* **1993**, *98*, 5648–5652. (b) Lee, C.; Yang, W.; Parr, R. G. Development of the Colle-Salvetti Correlation-Energy Formula into a Functional of the Electron Density. *Phys. Rev. B* **1988**, *37*, 785–789.
[21] Grimme, S. Semiempirical GGA-Type Density Functional Constructed with a Long-Range Dispersion Correction. *J. Comput. Chem.* **2006**, *27*, 1787–1799.
[22] Chai, J.-D.; Head-Gordon, M. Long-Range Corrected Hybrid Density Functionals with Damped Atom–Atom Dispersion Corrections. *Phys. Chem. Chem. Phys.* **2008**, *10*, 6615–6620.
[23] (a) Becke, A. D. Density-functional exchange-energy approximation with correct asymptotic behavior. *Phys. Rev. A* **1988**, *38*, 3098–3100. (b) Perdew, J. P. Density-functional approximation for the correlation energy of the inhomogeneous electron gas. *Phys. Rev. B* **1986**, *33*, 8822–8824.
[24] (a) Hehre, W. J.; Ditchfield, R.; Pople, J.A.; Self—Consistent Molecular Orbital Methods. XII. Further Extensions of Gaussian–Type Basis Sets for Use in Molecular Orbital Studies of Organic Molecules. *J. Chem. Phys.* **1972,** *56*, 2257. (b) Rassolov, V. A.; Pople, J. A.; Ratner, M. A.; Windus, T. L. 6-31G* basis set for atoms K through Zn. *J. Chem. Phys.* **1998**, *109*, 1223. (c) Mitin, A. V.; Baker, J.; Pulay, P. An improved 6-31G* basis set for first-row transition metals. *J. Chem. Phys.* **2003**, *118*, 7775-7782.
[25] (a) Weigend, F.; Ahlrichs, R. Balanced basis sets of split valence, triple zeta valence and quadruple zeta valence quality for H to Rn: Design and assessment of accuracy. *Phys. Chem. Chem. Phys.* **2005**, *7*, 3297. (b) Weigend, F. Accurate Coulomb-fitting basis sets for H to Rn. *Phys. Chem. Chem. Phys.* **2006**, *8*, 1057-1065.
[26] (a) Grimme, S.; Ehrlich, S.; Goerigk, L. Effect of the damping function in dispersion corrected density functional theory. *J. Comput. Chem.* **2011**, *32*, 1456–1465. (b) Grimme, S.; Antony, J.; Ehrlich, S.; Krieg, H. A consistent and accurate ab initio parametrization of density functional dispersion correction (DFT-D) for the 94 elements H-Pu. *J. Chem. Phys.* **2010**, *132*, 154104
[27] Luchini, G.; Paton, R. S. Bottom-Up Atomistic Descriptions of Top-Down Macroscopic Measurements: Computational Benchmarks for Hammett Electronic Parameters. *ACS Phys. Chem Au* **2024**, *4*, 259-267.

[28] Montgomery, J. A., Jr; Frisch, M. J.; Ochterski, J. W.; Petersson,G. A. A complete basis set model chemistry. VII. Use of the minimum population localization method. *J. Chem. Phys.* **2000**, *112*, 6532−6542.
[29] Glendening, E. D.; Badenhoop, J. K.; Reed, A. E.; Carpenter, J. E.; Bohmann, J. A.; Morales, C. M.; Karafiloglou, P.; Landis, C. R.; Weinhold, F. NBO 7.0., **2018**.
